# Supplementary material for: Precision design of stable genetic circuits carried in highly‐insulated E. coli genomic landing pads
Source: Mol Syst Biol. 2020 Aug 19;16(8):e9584. doi: 10.15252/msb.20209584 (PMC7436927; doi:10.15252/msb.20209584)
Supplement: Supplementary file 1 — Appendix [file MSB-16-e9584-s001.pdf]

Appendix for:

Precision design of stable genetic circuits carried in highly-insulated *E. coli* genomic landing pads

Yongjin Park[1], Amin Espah Borujeni[1], Thomas E. Gorochowski[1,2], Jonghyeon Shin[1] and Christopher A. Voigt[1,2,*]

[1] Synthetic Biology Center, Department of Biological Engineering, Massachusetts Institute of Technology, Cambridge, MA 02139, USA

[2] Broad Institute of MIT and Harvard, Cambridge, MA 02139, USA

Figure 1. Terminator characterization assay.
Figure 2. Comparison of expression at different integration sites between *E. coli* strains.
Figure 3. Comparison of expression levels between landing pads.
Figure 4. Comparison between plasmid RPU and genome RPU_G fluorescence levels.
Figure 5. Characterization of the sensor array at Landing Pad #1.
Figure 6. Genetic schematics for the genome-encoded NOT gates.
Figure 7. Optimization of NOT gates.
Figure 8. Growth impact of repressor expression from Landing Pad #1.
Figure 9. Example of a circuit designed using "tandem" NOR gates.
Figure 10. Replicate measurements are shown for the genetic circuits.
Figure 11. Cello-designed plasmid circuit for the 0xF1 logic operation.
Figure 12. Conversion of x-axis of response functions from [IPTG] to RPU_G
Figure 13. Performance of genome- and plasmid- encoded circuits when passaged for two weeks.
Figure 14. Plasmid and genome constructs used in this study.
Note 1. Detailed protocol for inserting payloads into genome landing pads
Table 1. Double terminator sequences and strengths
Table 2. Transposon library results for *E. coli* DH10β
Table 3. ON/OFF levels for genome-encoded sensors
Table 4. Sequence of landing pads used in this study
Table 5. Sequence of genetic parts used in this study
Table 6. Sequence of plasmids used in this study
Table 7. Sequence of genome integrated constructs used In this study
Table 8. Strains used in this study

References

a

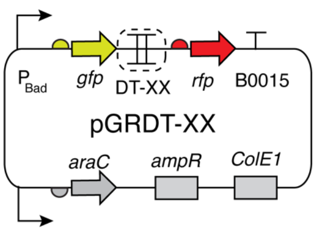

b

	Forward Terminator Strength						Reverse Terminator Strength					
Name	Day 1		Day 2		Day 3		Day 1		Day 2		Day 3	
	GFP	RFP	GFP	RFP	GFP	RFP	GFP	RFP	GFP	RFP	GFP	RFP
DT3	4100	0.4	3200	0.8	3100	0.8	3200	8.4	2900	10	3600	14
DT5	3100	0.9	4600	0.4	3500	0.5	3600	34	4700	51	3300	27
DT19	3400	2.0	2700	1.8	2200	1.5	2900	1300	3200	1500	4100	1400
DT34	4400	3.6	3300	2.4	3200	3.2	2600	950	3200	900	3800	1400
DT36	3400	2.5	3800	2.2	4500	3.8	3000	390	2900	470	3300	510
DT42	3200	0.4	3900	1.4	3900	1.0	6700	130	700	160	800	200
DT54	3100	0.6	3700	1.0	7700	2.7	3200	55	3500	60	4200	52
DT56	2700	5.4	3400	6.5	3300	7.1	540	20	560	26	660	41
DT60	3400	16	3200	14	4200	21	2900	48	3600	53	3800	79
DT65	2400	2.6	1500	1.4	3000	5.4	2200	1200	3000	1400	3100	1900
DT82	2200	6.3	1800	3.9	3000	11	2900	500	3300	570	3500	720
DT83	1800	1.7	4200	5.9	9100	2.9	2100	210	2700	310	3000	400
DT86	4800	11	2500	4.4	4000	12	3000	3100	4000	4700	4100	5200
DT100	5400	0.7	4500	0.5	9500	1.7	2700	120	3200	100	4100	180
DT101	4100	0.4	4600	0.7	3200	0.5	2900	10	5000	13	5200	20
DT103	3500	2.2	3000	1.6	3500	3.0	2400	260	3400	410	3200	500
DT104	3200	3.1	4400	4.9	3000	2.1	2400	11	3700	17	3300	19

Appendix Figure 1: **Terminator characterization assay. (a)** The plasmid to measure terminator strength is shown, where XX refers to the terminator name. The terminator to be measured is inserted between *gfp* and *rfp* genes in an operon and the measured fluorescences are used to calculate terminator strength T_S (Methods). **(b)** The raw fluorescence measurements for each terminator are shown.

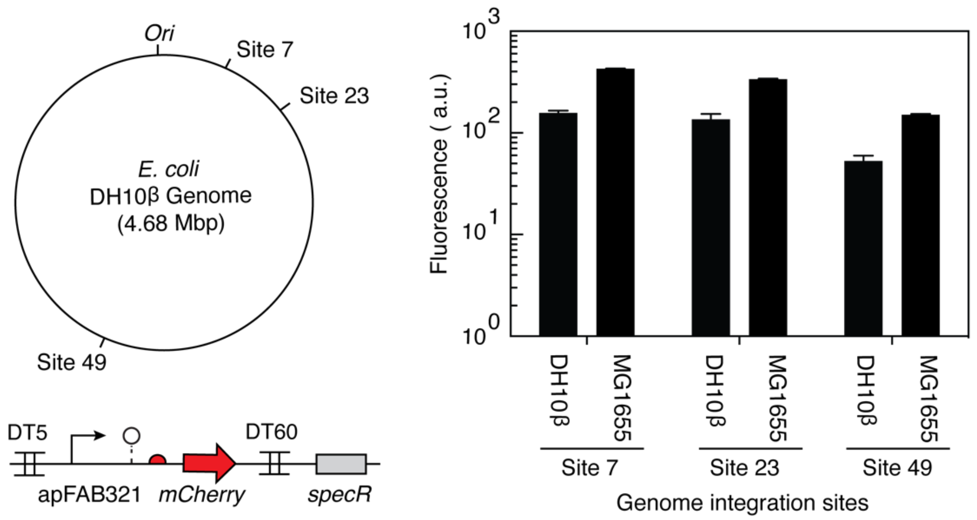

<u>Appendix Figure 2:</u> **Comparison of expression at different integration sites between *E. coli* strains.** Three sites are shown, selected from the Tn5 library (Appendix Table 2) (corresponding to nt positions #4294255, #4614289, #1983293 in the genome sequence NCBI CP000948.1). The construct integrated at these sites is shown. The means of three experiments performed on different days are shown and the error bars are the standard deviations of these measurements.

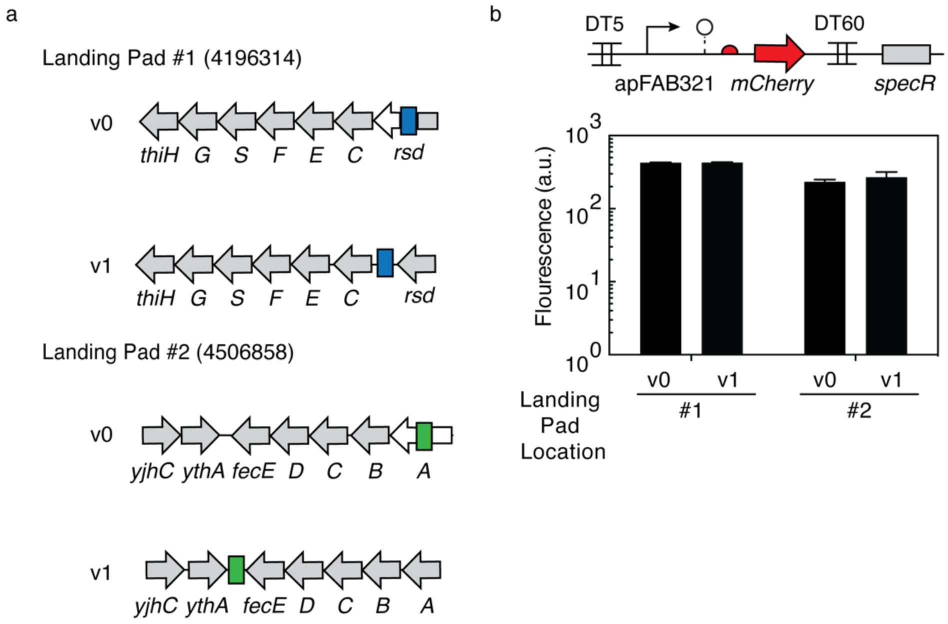

<u>Appendix Figure 3:</u> **Comparison of expression levels between landing pads. (a)** The locations of the landing pads in the genome are shown. **(b)** Experiments were performed where a constitutive expression cassette was evaluated at these positions (but not containing the context of the double terminators and integration sites) (Methods). The means are shown for three experiments performed on different days and the error bars are the standard deviations of these measurements.

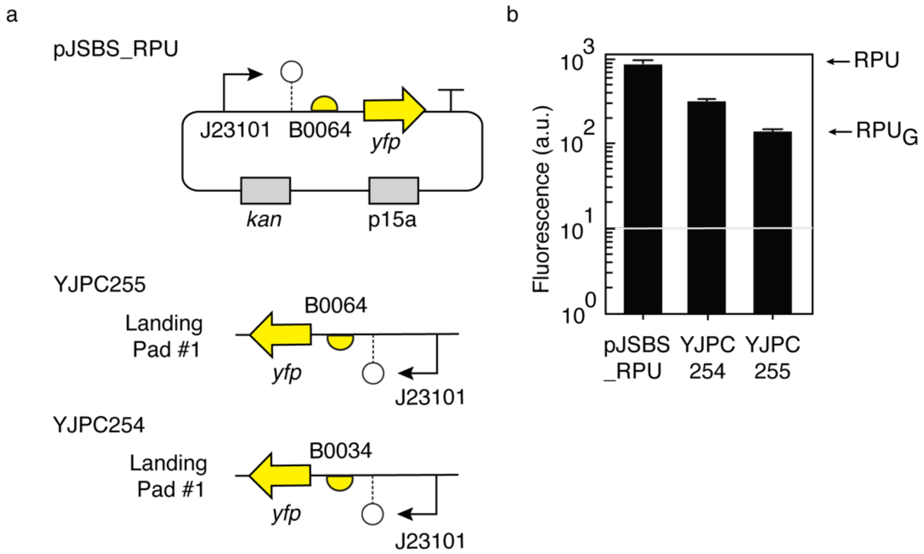

<u>Appendix Figure 4:</u> **Comparison between plasmid RPU and genome RPU_G fluorescence levels.** (a) The constructs used for evaluating the reference promoter are shown, with the plasmid names on the left[1]. **(b)** Fluorescence measurements of cells carrying the constructs are shown (Methods). The horizontal line shows the background fluorescence of cells. By comparing expression levels between the YJPC255 and pJSBS_RPU plasmids, the conversion factor between RPU and RPU_G was calculated (1 RPU = 6.3 RPU_G). Part sequences are provided in Supplementary Table 5. The means are shown for three experiments performed on different days and the error bars are the standard deviations of these measurements.

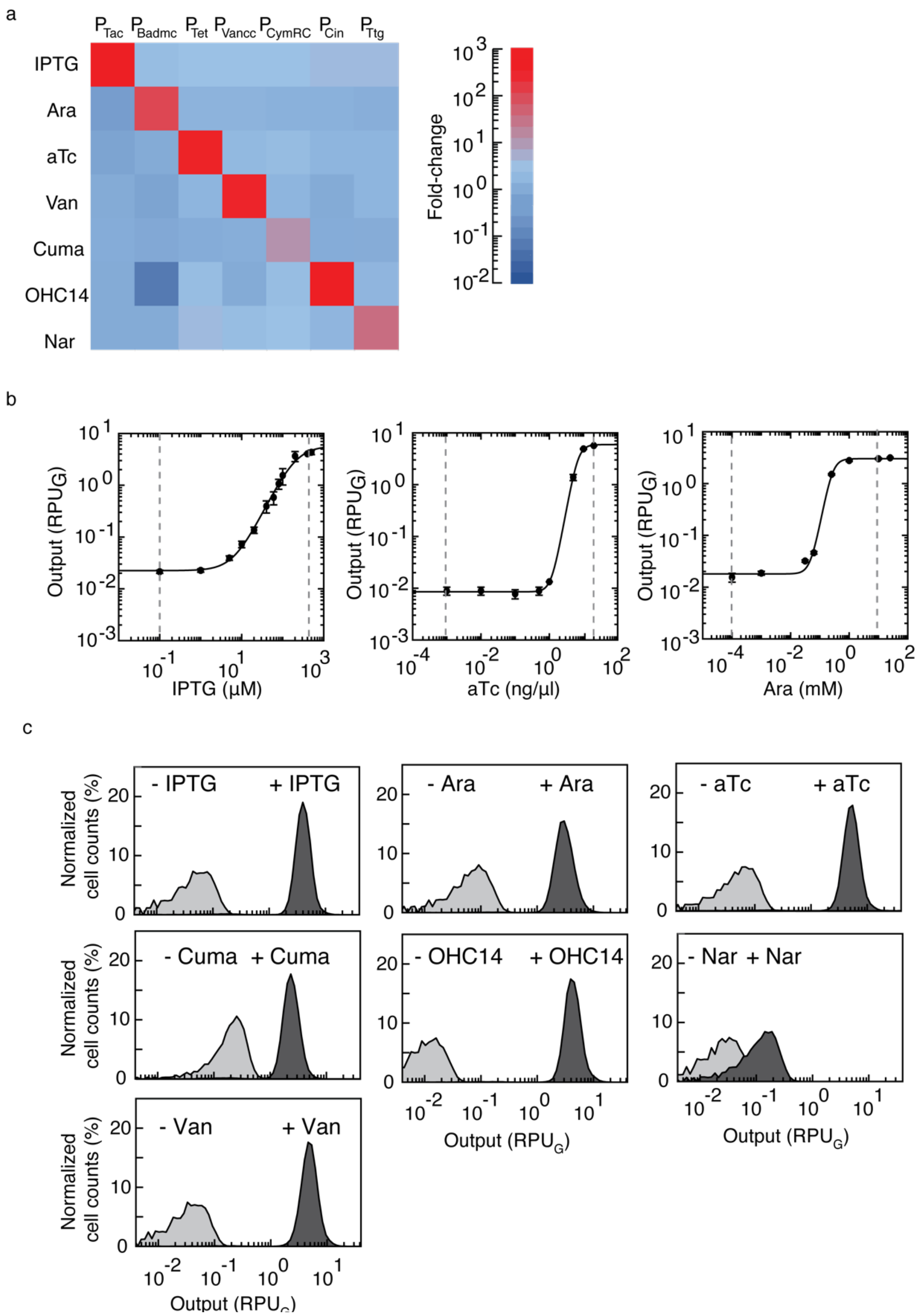

<u>Appendix Figure 5:</u> **Characterization of the sensor array at Landing Pad #1**. The genetic construct showing the sensors is shown in Figure 2c. **(a)** Sensor orthogonality. For these experiments, the output promoter of each sensor is transcriptionally fused to *yfp* and inserted into Landing pad #1 (Appendix Figure S18). The cross reactivity of the sensors is shown when the following inducer concentrations were used: 12.5 mM Ara, 1 mM IPTG, 20 ng/µl aTc, 500 µM Cuma, 200 µM Van, 10 µM OHC14, and 1 mM Nar.

The fold-change is calculated from the median fluorescence in the presence of inducer divided by that measured in its absence. Growth and measurement conditions are provided in the Methods. The data represents the mean from three replicates measured on different days. **(b)** Full response functions for the three sensors used in the circuit designs. The P_{Tac} response function shown was used to convert the x-axis of the NOT gate response functions from concentration to RPU_G (Appendix Figure S12). The means are shown for three experiments performed on different days are and the error bars are the standard deviation of these measurements. Two gray dotted lines indicate inducer concentrations used for Cello prediction and circuit design. **(c)** The fluorescence distribution measured from flow cytometry and was converted into RPU_G (Methods). For each output promoter, both the OFF (without inducer, light gray) and ON (with inducer, darker gray) states are shown in the same plot. The experiments were repeated three times on different days with similar results. The mean of three replicates are shown in Figure 2C.

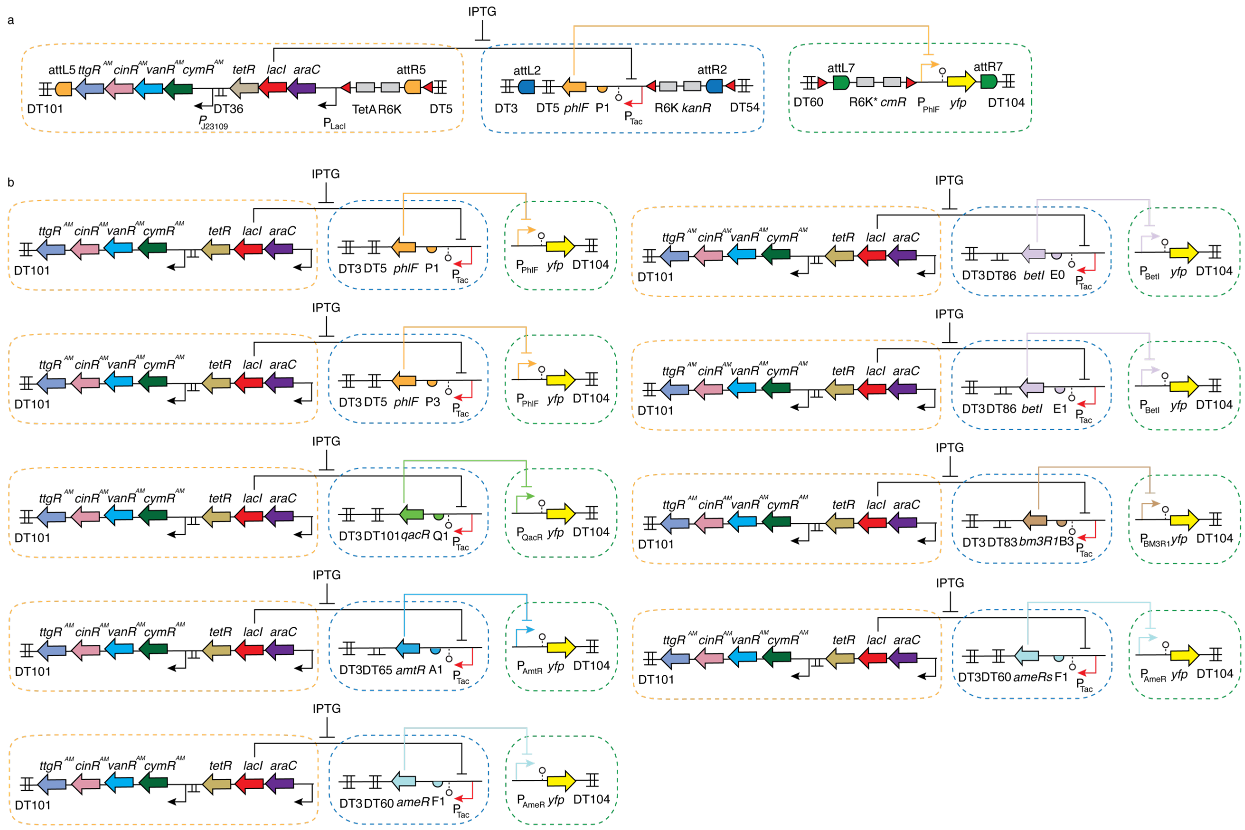

<u>Appendix Figure 6:</u> Genetic schematics for the genome-encoded NOT gates. (a) Schematic representation of the P1-PhlF-NOT gate on the genome. This construct is shown with the additional parts in the landing pad (*e.g.,* antibiotic resistance genes). Genetic parts and the integration sequences are provided in Appendix Tables 4, 5 and 6. **(b)** Design of 9 NOT gates implemented on the genome (genomic parts not shown for clarity). The constructs inserted into each landing pad are: landing pad #1 (blue), landing pad #2 (green) and landing pad #3(orange).

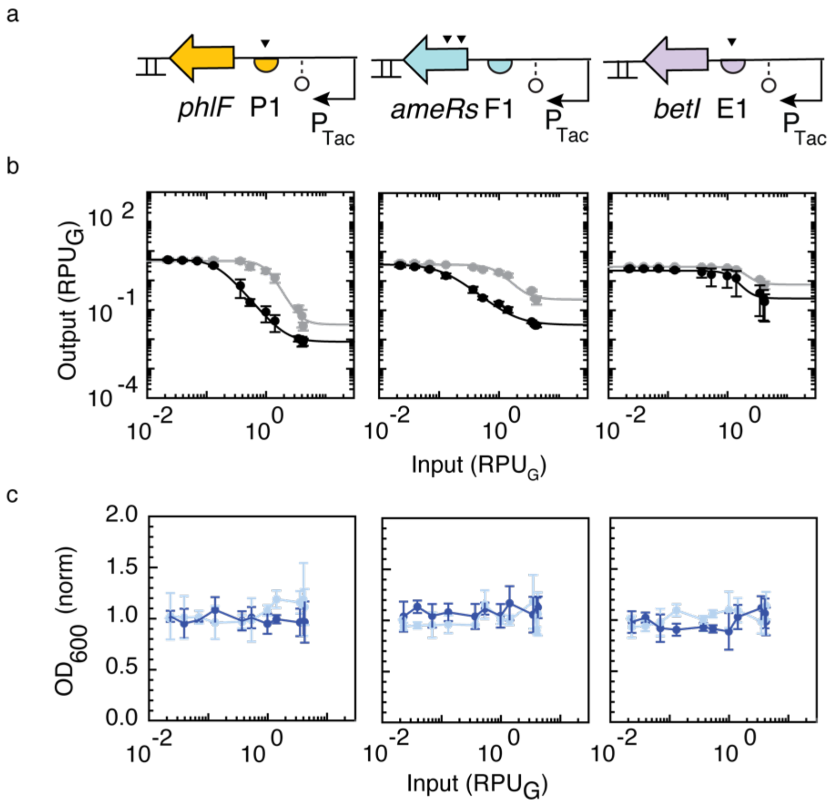

Appendix Figure 7: **Optimization of the NOT gates.** (**a**) Schematics of each gate; the changes made during optimization are indicated by the triangles. For the PhlF gate, the RBS was changed from P3 (CTTTACGAGGGCGATCCT) to P1 (CTATGGACTATGTTTGAAAGGGAGAAATACTAG). Two mutations were made in *ameR* (R25H and R43S) to create *ameRs*. For the BetI gate, the RBS was changed from E0 (CCCCCCGAGGAGTAGCAC) to E1 (CCTTCCGAGGAGGAGCACA). **(b)** The response functions before (gray) and after (black) optimization. **(c)** The impact on the growth of cells is shown before (light blue) and after (dark blue) optimization. The means are shown for three experiments performed on different days and the error bars are the standard deviations of these measurements.

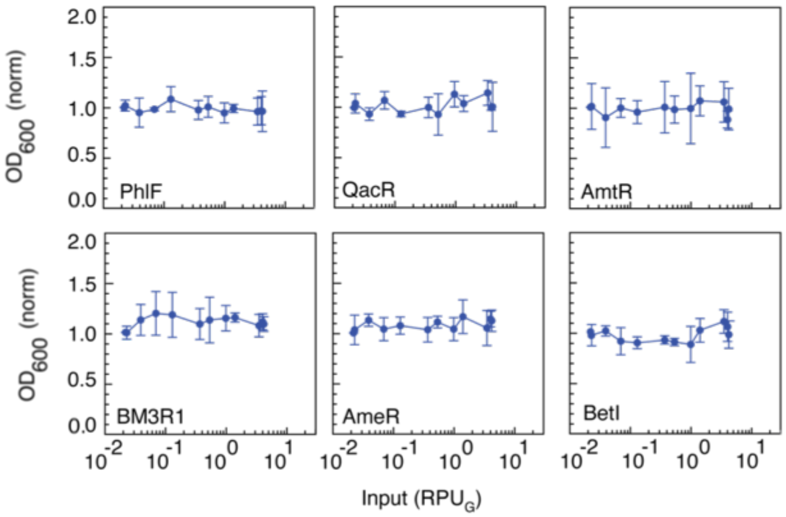

<u>Appendix Figure 8:</u> **Growth impact of repressor expression from Landing Pad #1.** Changes in OD_{600} upon the expression of repressors are shown (Methods). These data were used to create the blue lines shown in Figure 2D. The means are shown for three experiments performed on different days and the error bars are the standard deviations of these measurements. Note that the data used to plot the growth impact of PhiF, BetI, AmeRs gates were also used in Appendix Figure S7.

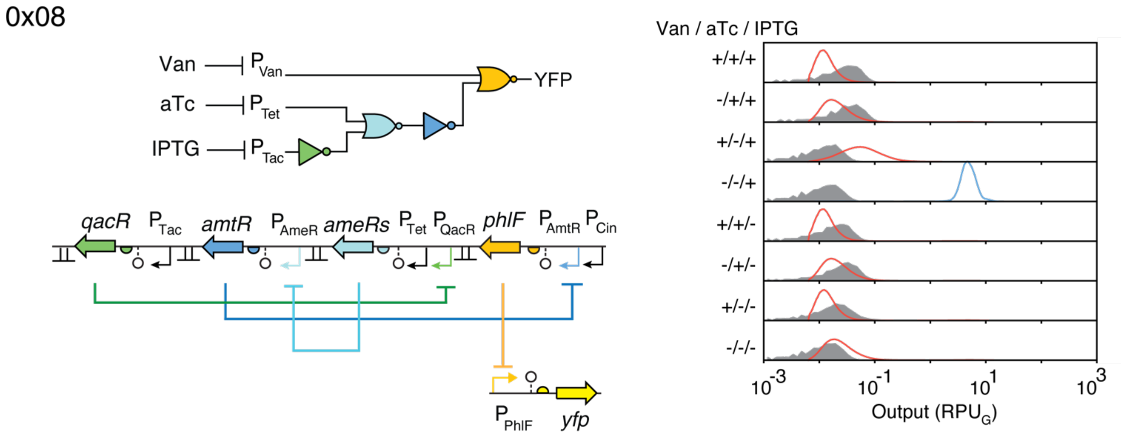

Appendix Figure 9: **Example of a circuit designed using "tandem" NOR gates.** The gates are based on two promoters in series driving the expression of the repressor (Figure 3F). The wiring diagram, repressor assignment, and genetic construct designed by Cello is shown. The predicted outputs are shown as blue (on) or red (off) distributions and the experimental data as gray distributions (Methods). The concentrations of inducers used are: 200 mM Van, 20 ng/µl aTc, and 1mM IPTG. The experiments were repeated three times on different days with similar results.

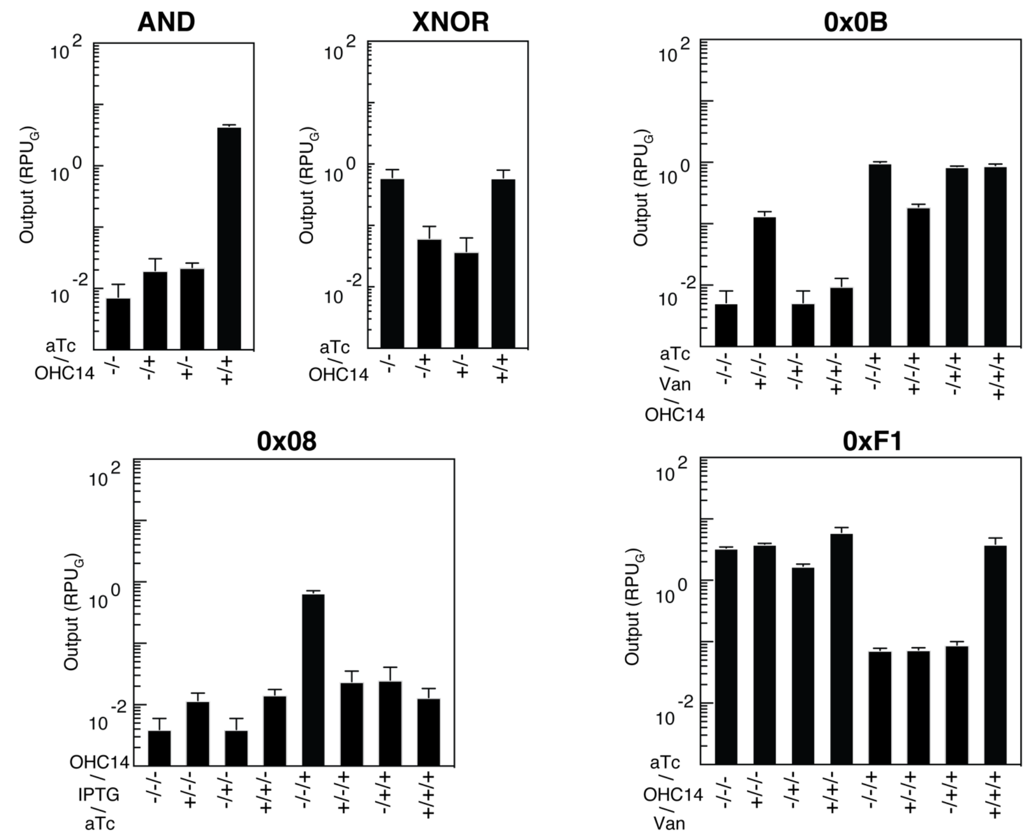

<u>Appendix Figure 10:</u> **Replicate measurements are shown for the genetic circuits.** This figure corresponds to the cytometry distributions in Figure 4. The concentrations of inducers used are: 1mM IPTG, 20 ng/µl aTc, 200 mM Van, and 10 µM of OHC14. These experiments were performed three times on different days and the means and standard deviations of the measurements are shown.

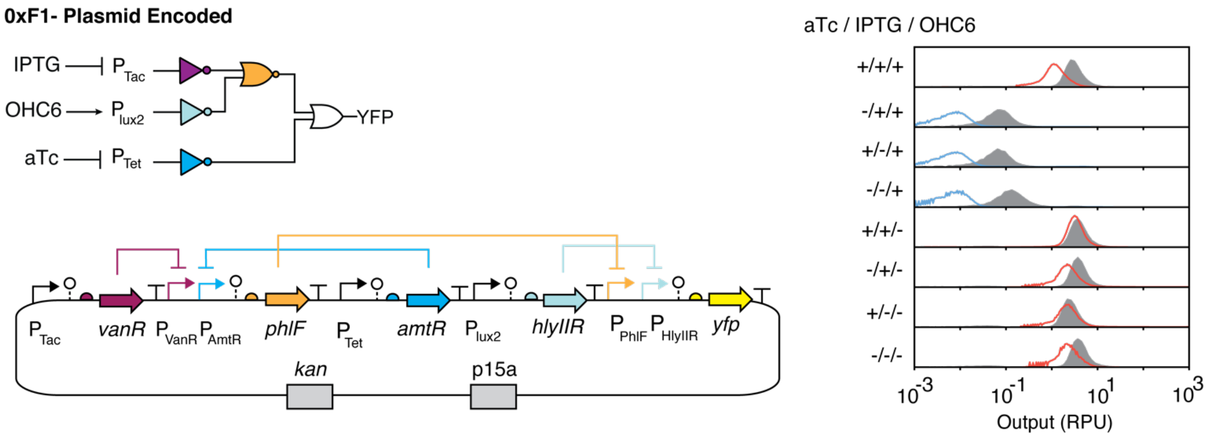

<u>Appendix Figure 11:</u> **Cello-designed plasmid circuit for the 0xF1 logic operation**. This logic operation corresponds to the genome-encoded circuit shown in Figure 4 and used for the evolutionary stability experiments in Figure 5. The following inducer concentrations are used: 2 ng/µl aTc, 0.2 mM IPTG and 10^{-4} ng/µl OHC6. The distributions predicted by Cello are shown in blue (ON states) or red (OFF states) and the grey distributions are the experimental measurements. The experiments were repeated three times on different days with similar results.

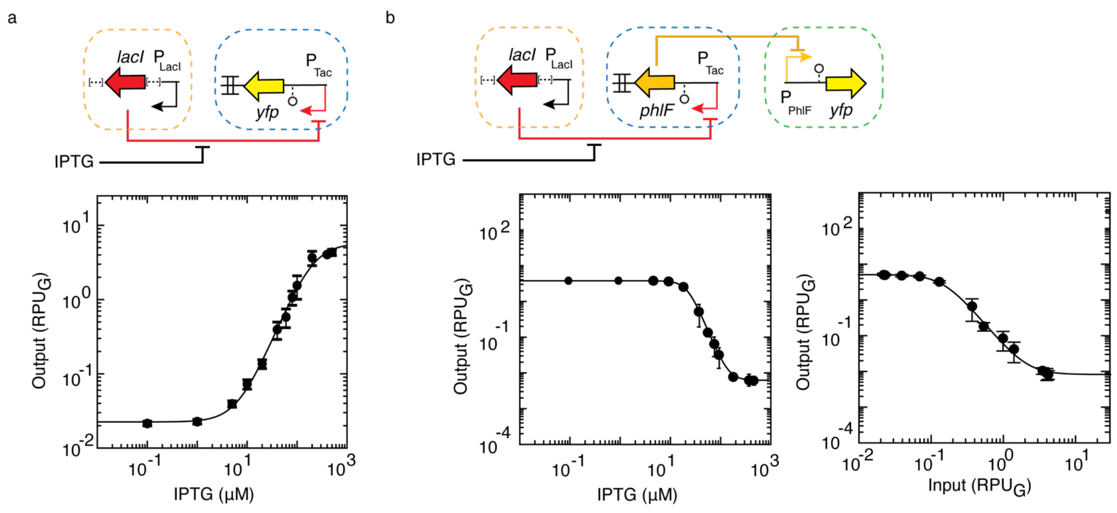

<u>Appendix Figure 12:</u> **Conversion of x-axis of response functions from [IPTG] to $\mathbf{RPU_G}$.** The constructs at each landing pad are shown: blue (LP #1), green (LP #2) and orange (LP #3). In all subfigures, the concentrations of IPTG used were 0.1, 1, 5, 10, 20, 40, 60, 80, 100, 200, 400, 500 μM. **(a)** The construct and response function from the IPTG sensor were first used to obtain the activity of P_{Tac}. (The […] indicates the remainder of the sensor array, removed from the schematic for clarity). **(b)** The response function of the PhlF gate was first obtained as a function of IPTG (top). Then, the P_{Tac} activity at each IPTG concentration (part a) is used to convert the x-axis (bottom), which is used to fit to the response function to parameterize the gate. The data represent the means of three experiments performed on different days and the error bars are the standard deviations.

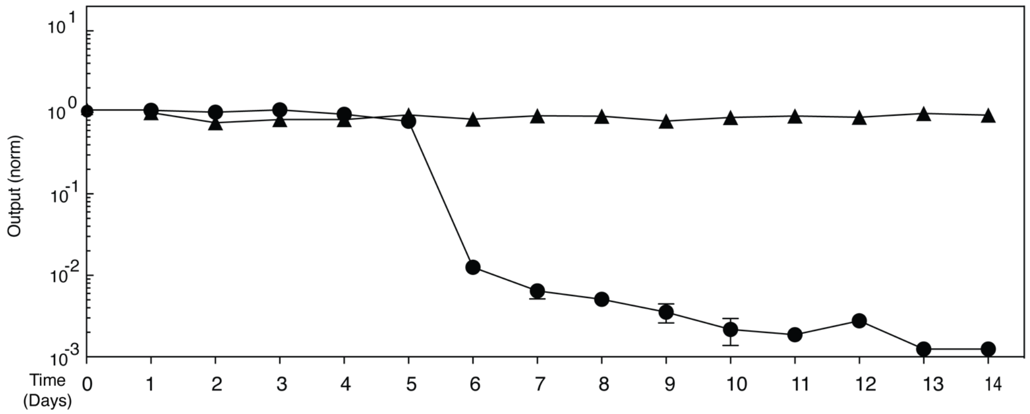

Appendix Figure 13: **Performance of genome- and plasmid- encoded circuits when passaged for two weeks.** The constructs tested are: plasmid (0xF1, YJP_DHC54, circle) and genome (YJP_MKC267, triangle). YJP_MKC267 was streaked on LB agar (2%) plates with Kan (50 µg/ml) and Cm (35 µg/ml) antibiotics and grown overnight. YJP_DHC54 was streaked on LB agar (2%) plates containing Kan (50 µg/ml) and grown overnight. Three individual colonies were inoculated in the M9 media without antibiotics. The circuits were tested under the presence of + 200 µM vanillic acid (genome) or + 2 ng/µl aTc (plasmid) (the other inducers are not present) in M9 media without antibiotics. Each day, cells were diluted 10,000-fold into 500 µl of fresh M9 media containing the same inducer noted above and were grown for 8 hours at 37 °C. After the incubation at 37 °C and 900 rpm in a Multitron Pro incubator shaker (In Vitro Technologies, VIC, Australia), 30 µl of cells were added to 200 µl 1x PBS with 2 mg/ml Kan for flow cytometry analysis and 100 µl of cells were mixed with 80% autoclaved glycerol (VWR chemical BDH1172-1LP) and stored at -80 °C. Another aliquot was diluted 100-fold into fresh media with the same inducer and incubated overnight. The cycle continued for 14 days by repeating this protocol. The means of three replicates are shown and the error bars are the standard deviations of these measurements. The error bars are often smaller than the data points. The output of the 0xF1 circuit was measured and normalized by the output at t = 0 days. Wild type E. coli MG1655 autofluorescence was measured as the output value of 0.005 a.u. (dotted line).

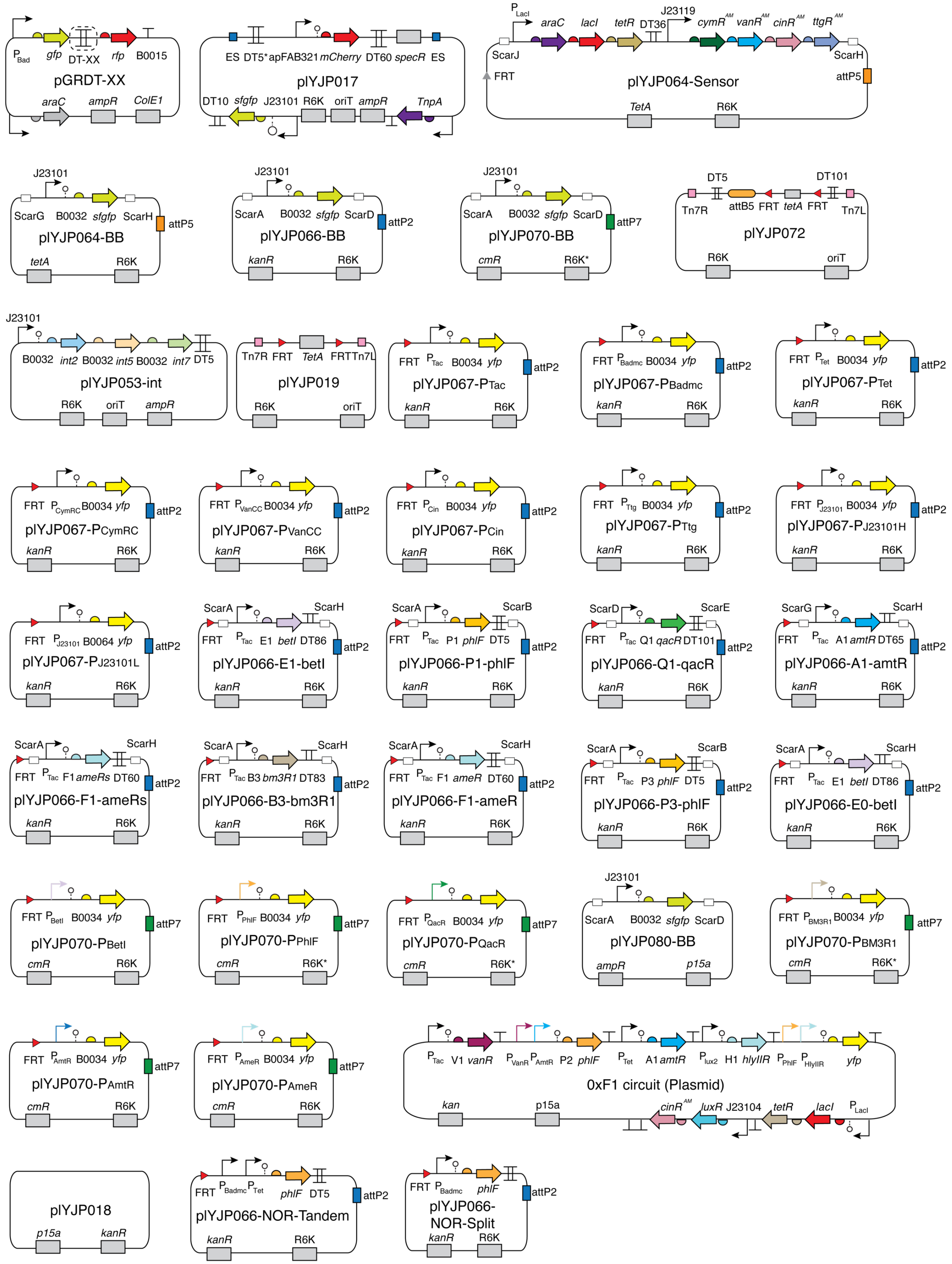
pGRDT-XX
PBad gfp DT-XX rfp B0015
araC ampR ColE1
pIYJP017
ES DT5* apFAB321 mCherry DT60 specR ES
DT10 sfgfp J23101 R6K oriT ampR TnpA
pIYJP064-Sensor
PLacI araC lacI tetR DT36 J23119 cymR AM vanR AM cinR AM ttgR AM
ScarJ FRT ScarH attP5
TetA R6K
pIYJP064-BB
J23101 ScarG B0032 sfgfp ScarH attP5
tetA R6K
pIYJP066-BB
J23101 ScarA B0032 sfgfp ScarD attP2
kanR R6K
pIYJP070-BB
J23101 ScarA B0032 sfgfp ScarD attP7
cmR R6K*
pIYJP072
DT5 DT101 Tn7R attB5 FRT tetA FRT Tn7L
R6K oriT
pIYJP053-int
J23101 B0032 int2 B0032 int5 B0032 int7 DT5
R6K oriT ampR
pIYJP019
Tn7R FRT TetA FRT Tn7L
R6K oriT
pIYJP067-PTac
FRT PTac B0034 yfp attP2 kanR R6K
pIYJP067-PBadmc
FRT PBadmc B0034 yfp attP2 kanR R6K
pIYJP067-PTet
FRT PTet B0034 yfp attP2 kanR R6K
pIYJP067-PCymRC
FRT PCymRC B0034 yfp attP2 kanR R6K
pIYJP067-PVanCC
FRT PVanCC B0034 yfp attP2 kanR R6K
pIYJP067-PCin
FRT PCin B0034 yfp attP2 kanR R6K
pIYJP067-PTtg
FRT PTtg B0034 yfp attP2 kanR R6K
pIYJP067-PJ23101H
FRT PJ23101 B0034 yfp attP2 kanR R6K
pIYJP067-PJ23101L
FRT PJ23101 B0064 yfp attP2 kanR R6K
pIYJP066-E1-betI
ScarA ScarH FRT PTac E1 betI DT86 attP2 kanR R6K
pIYJP066-P1-phlF
ScarA ScarB FRT PTac P1 phlF DT5 attP2 kanR R6K
pIYJP066-Q1-qacR
ScarD ScarE FRT PTac Q1 qacR DT101 attP2 kanR R6K
pIYJP066-A1-amtR
ScarG ScarH FRT PTac A1 amtR DT65 attP2 kanR R6K
pIYJP066-F1-ameRs
ScarA ScarH FRT PTac F1 ameRs DT60 attP2 kanR R6K
pIYJP066-B3-bm3R1
ScarA ScarH FRT PTac B3 bm3R1 DT83 attP2 kanR R6K
pIYJP066-F1-ameR
ScarA ScarH FRT PTac F1 ameR DT60 attP2 kanR R6K
pIYJP066-P3-phlF
ScarA ScarB FRT PTac P3 phlF DT5 attP2 kanR R6K
pIYJP066-E0-betI
ScarA ScarH FRT PTac E1 betI DT86 attP2 kanR R6K
pIYJP070-PBetI
FRT PBetI B0034 yfp attP7 cmR R6K
pIYJP070-PPhlF
FRT PPhlF B0034 yfp attP7 cmR R6K*
pIYJP070-PQacR
FRT PQacR B0034 yfp attP7 cmR R6K*
pIYJP080-BB
J23101 ScarA B0032 sfgfp ScarD ampR p15a
pIYJP070-PBM3R1
FRT PBM3R1 B0034 yfp attP7 cmR R6K*
pIYJP070-PAmtR
FRT PAmtR B0034 yfp attP7 cmR R6K
pIYJP070-PAmeR
FRT PAmeR B0034 yfp attP7 cmR R6K
0xF1 circuit (Plasmid)
PTac V1 vanR PVanR PAmtR P2 phlF PTet A1 amtR Plux2 H1 hlyIIR PPhlF PHlyIIR yfp
kan p15a cinR AM luxR J23104 tetR lacI PLacI
pIYJP018
p15a kanR
pIYJP066-NOR-Tandem
FRT PBadmc PTet phlF DT5 attP2 kanR R6K
pIYJP066-NOR-Split
FRT PBadmc phlF attP2 kanR R6K

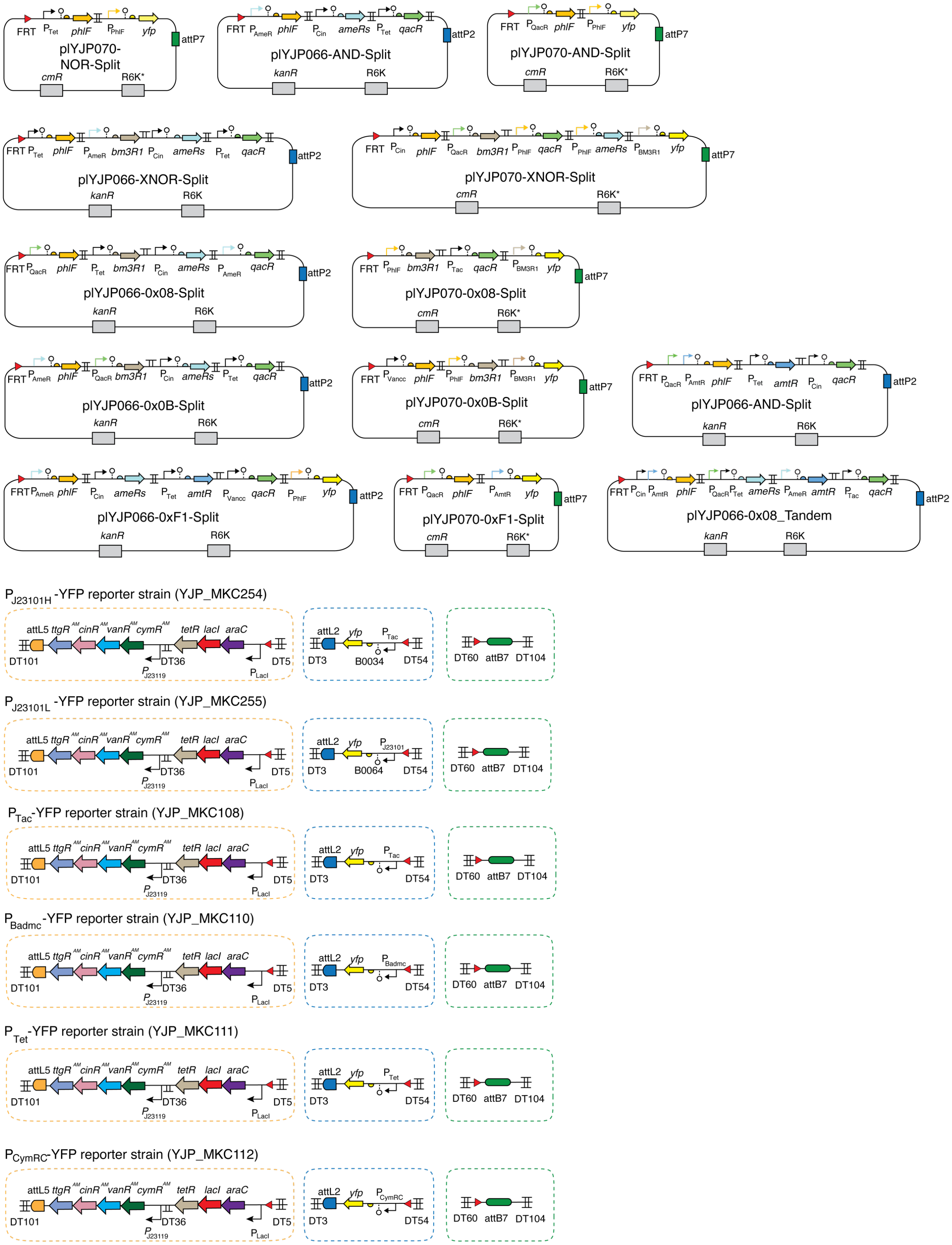
pIYJP070-NOR-Split
pIYJP066-AND-Split
pIYJP070-AND-Split
pIYJP066-XNOR-Split
pIYJP070-XNOR-Split
pIYJP066-0x08-Split
pIYJP070-0x08-Split
pIYJP066-0x0B-Split
pIYJP070-0x0B-Split
pIYJP066-AND-Split
pIYJP066-0xF1-Split
pIYJP070-0xF1-Split
pIYJP066-0x08_Tandem
FRT
attP7
attP2
kanR
cmR
R6K
R6K*
PJ23101H-YFP reporter strain (YJP_MKC254)
PJ23101L -YFP reporter strain (YJP_MKC255)
PTac-YFP reporter strain (YJP_MKC108)
PBadmc-YFP reporter strain (YJP_MKC110)
PTet-YFP reporter strain (YJP_MKC111)
PCymRC-YFP reporter strain (YJP_MKC112)
attL5 ttgR cinR vanR cymR tetR lacI araC
DT101
DT36
DT5
PJ23119
PLacI
attL2 yfp
DT3
B0034
B0064
DT54
DT60 attB7 DT104

P_{VanCC}-YFP reporter strain (YJP_MKC113)

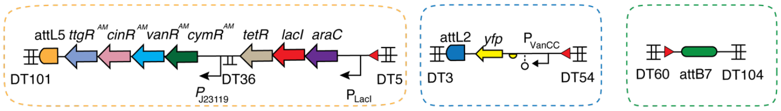

P_{Cin}-YFP reporter strain (YJP_MKC114)

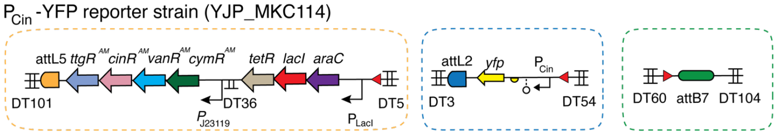

P_{Ttg}-YFP reporter strain (YJP_MKC115)

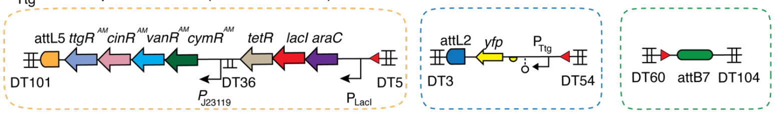

PhlF-NOR gate- Tandem design (YJP_MKC258)

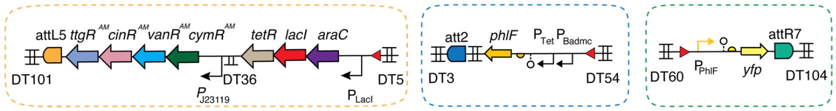

PhlF-NOR gate- Split design (YJP_MKC259)

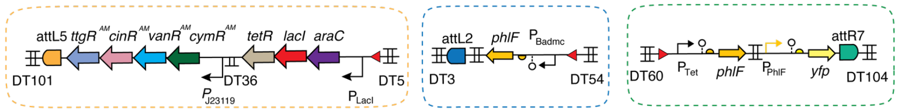

AND circuit- Split- (YJP_MKC262)

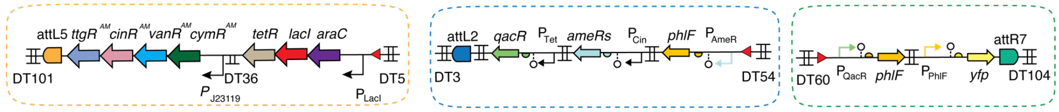

XNOR circuit (YJP_MKC263)

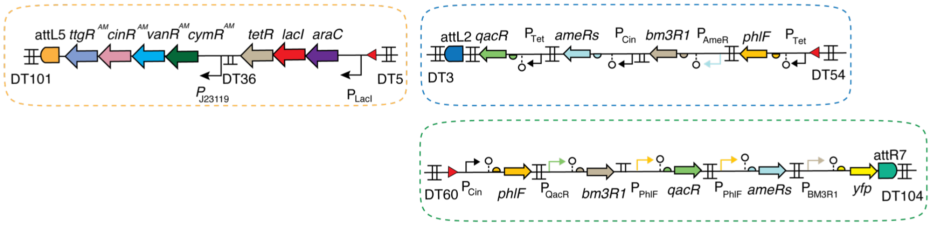

0x08 circuit (YJP_MKC264)

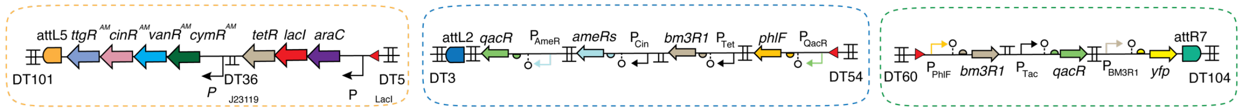

0x0B circuit (YJP_MKC265)

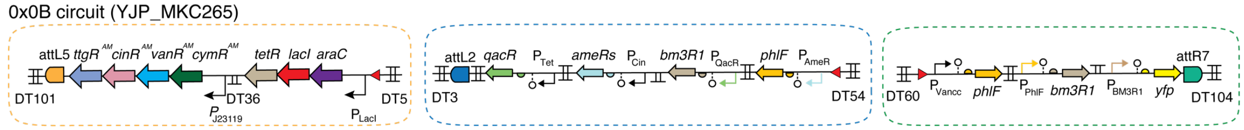

0xF1 circuit (YJP_MKC266)

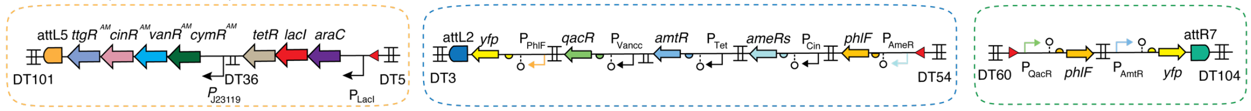

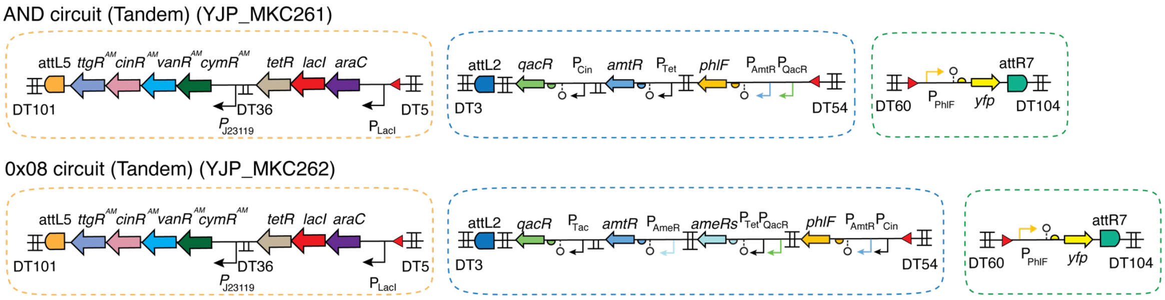

<u>Appendix Figure 14:</u> Plasmid and genome constructs used in this study. All genetic parts are provided in Appendix Table 5. Plasmid sequences are provided in Appendix Table 6.

Appendix Note 1: **Detailed protocol for inserting payloads into genome landing pads**

PREPARATION OF LANDING PAD CONSTRUCTS

Construct plasmids containing the payloads to be inserted in landing pads 1, 2, 3.

1. Clone payloads to one of pIYJP066-KanR, pIYJP070-CmR or pIYJP064-TetR plasmids to target landing pad 1,2, and 3, respectively.
2. Transform the cloned product into *E. coli* EC100D pir+ competent cells and sequence verify the construct
3. Miniprep plasmids (~250 ng/µl).

PREPARATION OF ELECTROCOMPETANT CELLS CONTAINING LANDING PADS

1. Streak cells harboring empty landing pads (*E. coli* MG1655 YJPC173) on a 2% LB-agar plate without antibiotics and incubate overnight at 37 °C.
2. Pick a single colony and inoculate into 2 ml LB no salt media (water + 10 g/L tryptone + 5 g/L yeast extract, autoclaved) without antibiotics and incubate overnight at 37 °C, shaking at 250 rpm.
3. Dilute cells 50-fold 1 ml into 50 ml LB no salt medium in a 250-ml Erlenmeyer flask.
4. Grow 1-2 hours until reaching OD_{600} = 0.55-0.65.
5. Pre-chill 50-ml Falcon tubes, electroporation cuvettes and 10% glycerol solution while cells are growing.
6. Transfer cells to pre-chilled 50-ml Falcon tubes.
7. Centrifuge cells at 4000g and 4°C for 10 minutes.
8. Remove the supernatant.
9. Resuspend cell pellet in 4 ml of ice-chilled 10% glycerol.
10. Centrifuge cells at 4000g and 4°C for 10 minutes.
11. Remove the supernatant.
12. Repeat Steps 9-11 twice more for a total of three glycerol washes.
13. Resuspend cell pellet in 175 µl of ice-chilled 10% glycerol, yielding electrocompetent cells.
14. If making frozen aliquots, which we have found decreases transformation efficiency, aliquot 100 µl cells into pre-chilled 200-µl PCR strip tubes. Each 100 µl aliquot will yield a single transformation.

TRANSFORMATION WITH PAYLOAD DNA

15. Aliquot 100 µl cells into pre-chilled electroporation cuvettes.
16. Add 1500 ng of the integrase plasmid (pIYJP053) and 500-1000 ng of the DNA containing the plasmid with payload.
    1. In order to ensure that the electroporation does not arc, it is important to ensure minimal salts. Salts in prepped plasmids can be removed using plasmid dialysis
    2. For plasmid dialysis:
        a. Fill appropriate number of wells in a 24-well plate with autoclaved Milli-Q purified water (one per transformation).
        b. Place DNA dialysis disc (Millipore Sigma, USA, VSWP01300) floating on top of water.
        c. Spot plasmids to be transformed on disc and leave for 30 minutes.
        d. Carefully retrieve plasmids from discs using pipette without submerging disc.
    3. For higher throughput dialysis method, we have also used Pierce micro-dialysis plates (ThermoFisher Scientific, 88260).
17. Electroporate the mixture (Using the Eppendorf Electroporator 2510, we found that 2500 mA and a time constant after the transformation >4.0 worked best).
18. Immediately after electroporation, add 500 µl of SOC recovery media to the cells.

19. Incubate the cells at 30 °C for 1-2 hours and then spread on a 2% LB agar plate with appropriate antibiotic (Insertion in landing pad #1, #2 and #3 was selected with 50 µg/ml Kan, 35 µg/ml Cm or 5 ng/ml Tet, respectively).
20. Incubate overnight at 37 °C.
21. Pick colony and inoculate in 200 µl LB with appropriate antibiotic for 3 hours. Typically, we pick three colonies for a higher likelihood of identifying a successful integration.

PCR SCREENING FOR SUCCESSFUL INTEGRATION

22. Primers that can amplify the junction between integrated constructs and the adjacent genomic DNA were used to validate the integration of the payload. The following primers are used at concentrations of 10 µM.
    a. LP #1: oYJP2164 (AATAAACAAATAGGCATGGTCTAAGAAACCATT)
       oYJP3436 (CCTGATCAGGTTCCGCGGATCCCGAATAAACGGTC)
    b. LP #2: oYJP2164 (AATAAACAAATAGGCATGGTCTAAGAAACCATT)
       oYJP3526 (TGCCAAAGGCGATAGGTGAAATAATGTCGGCGACAGCGG)
    c. LP #3: oYJP2166 (CACAAAACGGTTTACAAGCATAAAATCTCTG)
       oYJP2826(AGAGATGACAGAAAAATTTTCATTCTGTGACAGAGAAAAAGTAGCCGAAG ATG)

23. Prepare an aliquot of cells for the PCR reaction. Take 30 µl of the cultures from Step 21 and incubate them at 98 °C for 10 minutes.
24. Perform the PCR reaction. Our preference is NEB Hi-Fi Phusion Master Mix (NEB M0531) with the following volumes: 12.5 µl Phusion Master Mix 2X, 8.5 µl water, 1.25 µl, 1.25 µl Primer #1, 1.25 µl Primer #2, 1 µl template (the cell mixture from Step 23) and 0.5 µl DMSO. The thermocycler parameters are: 90s initial denaturation (98 °C), 20s denaturation (98 °C), 30 s anneal (60°C), 1 min/kb extension (72 °C), repeat denaturation/anneal/extension steps 29 times, 1 kb/min final extension (72 °C).
25. Confirm the amplicon size using gel electrophoresis by adding 5 µl of PCR product into 1% agarose gel. NEB 1kb ladder was run together to confirm the size.

REMOVAL OF ANTIBIOTIC RESISTANCE MARKERS

26. Inoculate the 170 µl culture from Step 21 to 4 ml SOB without antibiotics for 3 hours at 37 °C.
27. Once cells reach OD_{600}=0.4, Centrifuge cells at 4700g and 4 °C for 10 minutes.
28. Remove the supernatant.
29. Add 1 ml of ice-chilled 10% glycerol to the cell pellet.
30. Transfer the resuspended cells into a 1.5 ml Eppendorf tube and centrifuge at 21000g and 4 °C for 30 seconds.
31. Remove the supernatant.
32. Repeat steps 30 to 31.
33. Add 20 ng of the FLP encoding plasmid (pE-FLP)
34. Electroporate the mixture using the Eppendorf Electroporator 2510 at 2500 mA with time constant after the transformation >4.0.
35. Immediately after electroporation, add 1 ml of SOC recovery media to the cells.
36. Incubate the cells at 30 °C for 1 hour and then spread on a 2% LB agar plate with 100 µg/ml.
37. Incubate the cells overnight at 30 °C
38. Pick colony and streak the colony on 2% LB agar plate with no antibiotics.
39. Pick 5-6 colonies from the streak after 14 hours of incubation and resuspend each to 30 µl LB without antibiotics.

40. Inoculate the resuspended cells into 5 different media by adding 5 µl of resuspended cells. The five media compositions are: LB without antibiotics, with Amp 100 µg/ml, Kan 50 µg/ml, Cm 35 µg/ml, Tet 5 ng/ml. Incubate at 37 °C overnight.
41. Find colonies that only grew on LB without antibiotics.
42. Take 30 µl of the cultures from Step 41 and incubate them at 98 °C for 10 minutes.
43. PCR amplify using following oligos to confirm the removal of antibiotic markers from landing pads.
44. Primers that can amplify the junction between integrated constructs and the adjacent genomic DNA were used to validate the integration of the payload. The following primers are used at concentrations of 10 µM.
    a. LP #1: oYJP3436 (CCTGATCAGGTTCCGCGGATCCCGAATAAACGGTC)
       oYJP3437 (AGGCGCTGGAAGCGCGCTTTGTGCTGGAAGATAAG)
    b. LP #2: oYJP3525 (ACCAATTGGCGCGCGCTTCGCAATAAAATCTCCCTTCG)
       oYJP3526 (TGCCAAAGGCGATAGGTGAAATAATGTCGGCGACAGCGG)
    c. LP #3: oYJP2826
       (AGAGATGACAGAAAAATTTTCATTCTGTGACAGAGAAAAAGTAGCCGAAGATG)
       oYJP2827(CCGCGTAACCTGGCAAAATCGGTTACGGTTGAGTAA)
45. Perform the PCR reaction. Our preference is NEB Hi-Fi Phusion Master Mix (NEB M0531) with the following volumes: 12.5 µl Phusion Master Mix 2X, 8.5 µl water, 1.25 µl, 1.25 µl Primer #1, 1.25 µl Primer #2, 1 µl template (the cell mixture from Step 42) and 0.5 µl DMSO. The thermocycler parameters are: 90s initial denaturation (98 °C), 20s denaturation (98 °C), 30 s anneal (66°C), 1 min/kb extension (72 °C), repeat denaturation/anneal/extension steps 29 times, 1 kb/min final extension (72 °C).
46. Confirm the amplicon size using gel electrophoresis by adding 5 µl of PCR product into 1% agarose gel. NEB 1kb ladder was run together to confirm the size.
47. Once the size is confirmed, add 25% autoclaved glycerol (v/v) to create glycerol stock.
48. Store glycerol stock at -80 °C.

Appendix Table 1: Double terminator sequences and strengths

Name	Sequence	T_S (forward)[a]	T_S (reverse)[a]
DT3	CCGGCTTATCGGTCAGTTTCACCTGATTTACGTAAAAACCCGCTTCGGCGGGTTTTTGCTTTTGGAGGGGCAGAAAGATGAATGACT<br>GTCCACGACGCTATACCCAAAAGAAAAAAAAAAAAACCCCGCCCCTGACAGGGCGGGGTTTTTTTT	3000	120
DT5	TCCGGCAATTAAAAAAGCGGCTAACCACGCCGCTTTTTTTACGTCTGCACTCGGTACCAAATTCCAGAAAAGAGGCCTCCCGAAAG<br>GGGGGCCTTTTTTCGTTTTGGTCC	4700	50
DT19	TTCAGCCAAAAAACTTAAGACCGCCGGTCTTGTCCACTACCTTGCAGTAATGCGGTGGACAGGATCGGCGGTTTTCTTTTCTCTTCTC<br>AACTCGGTACCAAAGACGAACAATAAGACGCTGAAAAGCGTCTTTTTTCGTTTTGGTCC	770	1.2
DT34	GCTGATGCCAGAAAGGGTCCTGAATTTCAGGGCCCTTTTTTTACATGGATTGCTCGGTACCAAATTCCAGAAAAGAGACGCTTTCGA<br>GCGTCTTTTTTCGTTTTGGTCC	570	1.4
DT36	GATCTAACTAAAAAGGCCGCTCTGCGGCCTTTTTTCTTTTCACTGTAACAACGGAAACCGGCCATTGCGCCGGTTTTTTTTGGCCT	680	3.2
DT42	AGTTAACCAAAAAGGGGGGATTTTATCTCCCCTTTAATTTTTCCTCGCAGATAGCAAAAAAGCGCCTTTAGGGCGCTTTTTTACATTG<br>GTGG	2500	2.2
DT54	GGAAACACAGAAAAAAGCCCGCACCTGACAGTGCGGGCTTTTTTTTTCGACCAAAGGCTCGGTACCAAATTCCAGAAAAGACACCC<br>GAAAGGGTGTTTTTTCGTTTTGGTCC	1800	30
DT56	TACCACCGTCAAAAAAAACGGCGCTTTTTAGCGCCGTTTTTATTTTTCAACCTTCCAGGCATCAAATAAAACGAAAGGCTCAGTCGA<br>AAGACTGGGCCTTTCGTTTTATCTGTTGTTTGTCGGTGAACGCTCTC	240	11
DT60	ACATTTAATAAAAAAAGGGCGGTCGCAAGATCGCCCTTTTTTACGTATGACACAGTGAAAAATGGCGCCCATCGGCGCCATTTTTTT<br>ATG	110	29
DT65	TGCTCGTACCAGGCCCCTGCAATTTCAACAGGGGCCTTTTTTTATCCAATTCCATCGGGTCCGAATTTTCGGACCTTTTCTCCGC	400	1.0
DT82	CTTATTCCATAACAAAGCCGGGTAATTCCCGGCTTTGTTGTATCTGAACAATAAATGGATGCCCTGCGTAAGCGGGGCATTTTTCTTC<br>CT	170	2.8
DT83	AGCGTCAAAAGGCCGGATTTTCCGGCCTTTTTTATTAGGCAGCATGCTGCCAGGTGATCCCCCTGGCCACCTCTTTT	600	4.4
DT86	TAATCATTCTTAGCGTGACCGGGAAGTCGGTCACGCTACCTCTTCTGAAGAAACAGCAAACAATCCAAAACGCCGCGTTCAGCGGC<br>GTTTTTTCTGCTTTTCT	210	0.4
DT100	GTGAAGTGAAAAATGGCGCACATTGTGCGCCATTTTTTTTGTCTGCCGTTTACCGCTTCTCTGAAAATCAACGGGCAGGTCACTGAC<br>TTGCCCGTTTTTTTATCCCTTCTCCACACCG	4700	12
DT101	TCTTTAAAAAGAAACCTCCGCATTGCGGAGGTTTCGCCTTTTGATACTCTGTCTGAAGTAATTCTTGCCGCAGTGAAAAATGGCGCC<br>CATCGGCGCCATTTTTTTATGCTTCCATTAGAAAGCAAAAAGCCTGCTAGAAAGCAGGCTTTTTTGAATTTGGCTCCTCTGAC	2800	160
DT103	AAAGTTCTGAAAAAGGGTCACTTCGGTGGCCCTTTTTTATCGCCACGGTTTGAGCAGTGCACTTGCTTAAAATCCCGCCAGCGGCGG<br>GATTTTTTATTGTCCGGTTTAAGACA	790	4.0
DT104	GCAGACAAAAAAAATGGCGCACAATGTGCGCCATTTTTCACTTCACAGGTACTATTGTTTTGAATTGAAAAGGGCGCTTCGGCGCCC<br>TTTTTGCATTTGTTGACGGCATATATTTGTATATCGAAGCGCCCTGATGGGCGCTTTTTTTATTTAATCGATAACCAGA	580	101

a. The means of three replicates performed on different days are shown. The conditions and calculation of T_S are provided in the Methods and Appendix Figure 1.

Appendix Table 2: Transposon library results for *E. coli* DH10β

Number	Position[a]	Strand	Expression[b,c]	Relative OD_{600}[b,d]
1	3986119	-	160	1.5
2	239205	+	140	1.3
3	1703536	+	50	1.1
4	4403940	-	110	0.9
5	2354832	-	90	1.1
6	3821587	+	120	1.3
7 (LP#1)	4294255	-	160	1.1
8	3636005	-	130	1.1
9	216090	+	110	1.2
10	130791	+	80	1.0
11	1974358	-	60	1.2
12	953620	+	80	1.4
13	3942414	+	180	1.1
14	4577260	+	110	1.0
15	4185554	-	130	1.2
16	3635939	+	160	1.0
17	3293003	-	130	1.2
18	970929	-	80	1.2
19	2968336	-	110	1.0
20	3280886	-	60	1.2
21	3839522	-	120	1.1
22	3007855	+	90	1.4
23 (LP#3)	4000978	-	150	1.2
24	4160155	+	140	1.0
25	311535	+	80	1.3
26	4158775	+	130	1.1
27	2612824	-	80	1.1
28	3119521	-	110	1.2
29	268509	+	90	0.9
30	3286750	+	100	1.0
31	4575120	-	110	0.9
32	4648089	+	90	1.4
33	4525992	+	60	1.1
34	3149195	-	70	0.8
35	3851188	-	140	1.1
36	3792231	+	150	1.0
37	2691678	-	100	1.2
38	4078450	-	190	1.0
39	3008076	-	90	0.9
40	3357859	-	90	1.3
41	124347	-	90	0.8
42	4161116	+	100	0.9
43	6673	-	140	0.8
44	1434023	-	60	1.0
45	3268733	+	110	0.9
46	2763676	-	100	1.0
47	391229	+	120	1.5
48 (LP#2)	4614289	+	140	1.3
49	1983293	+	50	0.7
50	4519171	-	80	1.0

a. The number is based on the nucleotide numbering of the E. coli DH10β genome (NCBI CP000948.1)
b. The means of three experiments performed on different days are shown.
c. The expression is measured as mCherry fluorescence (Methods). The construct is shown in Figure 1b
d. The relative OD_{600} *is calculated by dividing the* OD_{600} *by that of wild-type E. coli* DH10β (Methods)

Appendix Table 3: ON/OFF levels for genome-encoded sensors

Inducer	Output Promoter	Strain	Y_{min} (RPU_G)	Y_{max} (RPU_G)
Ara	P_{Badmc}	YJP_MKC110	0.04	3.33
IPTG	P_{Tac}	YJP_MKC108	0.02	4.20
aTc	P_{Tet}	YJP_MKC111	0.02	5.41
Cuma	P_{CymRC}	YJP_MKC112	0.19	2.39
Van	P_{VanCC}	YJP_MKC113	0.02	3.79
OHC14	P_{Cin}	YJP_MKC114	0.01	4.38
Nar	P_{Ttg}	YJP_MKC115	0.01	0.22

Appendix Table 4: Sequence of landing pads used in this study

Landing Pad[a]	Position	DNA Sequence[c]
#1	4196314	GGAAACACAGAAAAAAGCCCGCACCTGACAGTGCGGGCTTTTTTTTTCGACCAAAGGCTCGGTACCAAATTCCAGAAAAGACACCCGAAAGGGTGTTTTTT<br>CGTTTTGGTCCGAAGTTCCTATTCTCTAGAAAGTATAGGAACTTCGGGTCCCCAATAATTACGATTTAAATTGGCGAAAATGAGACGTTGATCGGCACGTA<br>AGAGGTTCCAACTTTCACCATAATGAAATAAGATCACTACCGGGCGTATTTTTTGAGTTATCGAGATTTTCAGGAGCTAAGGAAGCTAAAATGGAGAAAAA<br>AATCACTGGATATACCACCGTTGATATATCCCAATGGCATCGTAAAGAACATTTTGAGGCATTTCAGTCAGTTGCTCAATGTACCTATAACCAGACCGTTC<br>AGCTGGATATTACGGCCTTTTTAAAGACCGTAAAGAAAAATAAGCACAAGTTTTATCCGGCCTTTATTCACATTCTTGCCCGCCTGATGAATGCTCATCCG<br>GAATTTCGTATGGCAATGAAAGACGGTGAGCTGGTGATATGGGATAGTGTTCACCCTTGTTACACCGTTTTCCATGAGCAAACTGAAACGTTTTCATCGCT<br>CTGGAGTGAATACCACGACGATTTCCGGCAGTTTCTACACATATATTCGCAAGATGTGGCGTGTTACGGTGAAAACCTGGCCTATTTCCCTAAAGGGTTTA<br>TTGAGAATATGTTTTTCGTCTCAGCCAATCCCTGGGTGAGTTTCACCAGTTTTGATTTAAACGTGGCCAATATGGACAACTTCTTCGCCCCCGTTTTCACC<br>ATGGGCAAATATTATACGCAAGGCGACAAGGTGCTGATGCCGCTGGCGATTCAGGTTCATCATGCCGTTTGTGATGGCTTCCATGTCGGCAGAATGCTTAA<br>TGAATTACAACAGTACTGCGATGAGTGGCAGGGCGGGGCGTAAGAAGTTCCTATTCTCTAGAAAGTATAGGAACTTCGGACGGCGCAGAAGGGGAGTAGCT<br>CTTCGCCGGACCGTCGACATACTGCTCAGCTCGTCCCGGCTTATCGGTCAGTTTCACCTGATTTACGTAAAAACCCGCTTCGGCGGGTTTTTGCTTTTGGA<br>GGGGCAGAAAGATGAATGACTGTCCACGACGCTATACCCAAAAGAAAAAAAAAAAACCCCGCCCCTGACAGGGCGGGGTTTTTTTTGCTTCAAATGCGGAT<br>TTGACAGCTAGCTCAGTCCTAGGTATTGTGCTAGC
#2	4506858	ACATTTAATAAAAAAAGGGCGGTCGCAAGATCGCCCTTTTTTACGTATGACACAGTGAAAAATGGCGCCCATCGGCGCCATTTTTTTATGGAAGTTCCTAT<br>TCTCTAGAAAGTATAGGAACTTCCACGCTCACCGGCTCCAGATTTATCAGCAATAAACCAGCCAGCCGGAAGCTCGAGTCCCGTCAAGTCAGCGTAATGCT<br>CTGCCAGTGTTACAACCAATTAACCAATTCTGATTAGAAAAACTCATCGAGCATCAAATGAAACTGCAATTTATTCATATCAGGATTATCAATACCATATT<br>TTTGAAAAAGCCGTTTCTGTAATGAAGGAGAAAACTCACCGAGGCAGTTCCATAGGATGGCAAGATCCTGGTATCGGTCTGCGATTCCGACTCGTCCAACA<br>TCAATACAACCTATTAATTTCCCCTCGTCAAAAATAAGGTTATCAAGTGAGAAATCACCATGAGTGACGACTGAATCCGGTGAGAATGGCAAAAGCTTATG<br>CATTTCTTTCCAGACTTGTTCAACAGGCCAGCCATTACGCTCGTCATCAAAATCACTCGCATCAACCAAACCGTTATTCATTCGTGATTGCGCCTGAGCGA<br>GACGAAATACGCGATCGCTGTTAAAAGGACAATTACAAACAGGAATCGAATGCAACCGGCGCAGGAACACTGCCAGCGCATCAACAATATTTTCACCTGAA<br>TCAGGATATTCTTCTAATACCTGGAATGCTGTTTTCCCGGGGATCGCAGTGGTGAGTAACCATGCATCATCAGGAGTACGGATAAAATGCTTGATGGTCGG<br>AAGAGGCATAAATTCCGTCAGCCAGTTTAGTCTGACCATCTCATCTGTAACATCATTGGCAACGCTACCTTTGCCATGTTTCAGAAACAACTCTGGCGCAT<br>CGGGCTTCCCATACAATCGATAGATTGTCGCACCTGATTGCCCGACATTATCGCGAGCCCATTTATACCCATATAAATCAGCATCCATGTTGGAATTTAAT<br>CGCGGCCTCGAGCAAGACGTTTCCCGTTGAATATGGCTCATAACACCCCTTGTATTACTGTTTATGTAAGCAGACAGTTTTATTGTTCATGATGATATATT<br>TTTATCTTGTGCAATGTAACATCAGAGATTTTGAGACACAACGTGGCTTTGTTGAGAAGTTCCTATTCTCTAGAAAGTATAGGAACTTCAGACGAGAAACG<br>TTCCGTCCGTCTGGGTCAGTTGGGCAAAGTTGATGACCGGGTCGTCCGTTGCAGACAAAAAAAATGGCGCACAATGTGCGCCATTTTTCACTTCACAGGTA<br>CTATTGTTTTGAATTGAAAAGGGCGCTTCGGCGCCCTTTTTGCATTTGTTGACGGCATATATTTGTATATCGAAGCGCCCTGATGGGCGCTTTTTTTATTT<br>AATCGATAACCAGA
#3	3911814	TGTGGGCGGACAATAAAGTCTTAAACTGAACAAAATAGATCTAAACTATGACAATAAAGTCTTAAACTAGACAGAATAGTTGTAAACTGAAATCAGTCCAG<br>TTATGCTGTGAAAAAGCATACTGGACTTTTGTTATGGCTAAAGCAAACTCTTCATTTTCTGAAGTGCAAATTGCCCGTCGTATTAAAGAGGGGCGTGGGGT<br>TCGAGGTCGACGGTATCGATAAGCTAGCTTAATTAGCTGAGCTTGTCCGGCAATTAAAAAAGCGGCTAACCACGCCGCTTTTTTTACGTCTGCACTCGGTA<br>CCAAATTCCAGAAAAGAGGCCTCCCGAAAGGGGGGCCTTTTTTCGTTTTGGTCCAGAAGTTCCTATTCTCTAGAAAGTATAGGAACTTCAGCGGAAAAGGA<br>CAATTGTCTCAGGTCGAGGTGGCCCGGCTCCATGCACCGCGACGCAACGCGGGGAGGCAGACAAGGTATAGGGCGGCGCCTACAATCCATGCCAACCCGTT<br>CCATGTGCTCGCCGAGGCGGCATAAATCGCCGTGACGATCAGCGGTCCAGTGATCGAAGTTAGGCTGGTAAGAGCCGCGAGCGATCCTTGAAGCTGTCCCT<br>GATGGTCGTCATCTACCTGCCTGGACAGCATGGCCTGCAACGCGGGCATCCCGATGCCGCCGGAAGCGAGAAGAATCATAATGGGGAAGGCCATCCAGCCT<br>CGCGTCGCGAACGCCAGCAAGACGTAGCCCAGCGCGTCGGCCGCCATGCCGGCGATAATGGCCTGCTTCTCGCCGAAACGTTTGGTGGCGGGACCAGTGAC<br>GAAGGCTTGAGCGAGGGCGTGCAAGATTCCGAATACCGCAAGCGACAGGCCGATCATCGTCGCGCTCCAGCGAAAGCGGTCCTCGCCGAAAATGACCCAGA<br>GCGCTGCCGGCACCTGTCCTACGAGTTGCATGATAAAGAAGACAGTCATAAGTGCGGCCACAATGGTCATGCCCCGCGCCCACCGGAAGGAGCTGACTGGG<br>TTGAAGGCTCTCAAGGGCATCGGACGGCGCTCTCCCTTATGCGACTCCTGCATTAGGAAGCAGCCCAGTAGTAGGTTGAGGCCGTTGAGCACCGCCGCCGC<br>AAGGAATGGTGCGTGCAGGGAGATGGCGCCCAACAGTCCCCCGGCCACGGGGCCTGCCACCATACCCACGCCGAAACAAGCGCTCATGAGCCCGAAGTGGC<br>GAGCCCGATCTTCCCCATCGGTGATGTCGGCGATATAGGCGCCAGCAACCGCACCTGTGGCGCCGGTGATGCCGGCCACGATGCGTCCGGCGTAGAGAATC<br>CACAGGACGGGTGTGGTCGCCATGATCGCGTAGTCGATAGTGGCTCCAAGTAGCGAAGCGAGCAGGACTGGGCGGCGGCCAAAGCGGTCGGACAGTGCTCC<br>GAGAACGGGTGCGCATAGAAATTGCATCAACGCATATAGCGCTAGCAGCACGCCATAGTGACTGGCGATGCTGTCGGAATGGACGATATCCCGCAAGAGGC<br>CCGGCAGTACCGGCATAACCAAGCCTATGCCTACAGCATCCAGGGTGACGGTGCCGAGGATGACGATGAGCGCATTGTTAGATTTCATACACGGTGCCTGA<br>CTGCGTTAGCAATTTAACTGTGATAAACTACCGCATTACAGTTTATCGATGATAAGCTGTCAAGAAGTTCCTATTCCGAAGTTCCTATTCTCTAGAAAGTA<br>TAGGAACTTCGAGCGCCGGATCAGGGAGTGGACGGCCTGGGAGCGCTACACGCTGTGGCTGCGGTCGGTGCTCTTTAAAAAGAAACCTCCGCATTGCGGAG<br>GTTTCGCCTTTTGATACTCTGTCTGAAGTAATTCTTGCCGCAGTGAAAAATGGCGCCCATCGGCGCCATTTTTTTATGCTTCCATTAGAAAGCAAAAAGCC<br>TGCTAGAAAGCAGGCTTTTTTGAATTTGGCTCCTCTGACTGCATTTACGTTGACACCATCGAATGCCAAGCTAGCTTGGCGAGATCCTTGCAGCACATCCC<br>CCTTTCGCCAGCTGGCGTAATAGCGAAGAGGCCCGCACCGATCGCAGGCCAACCAGATAAGTGAAATCTAGTTCCAAACTATTTTGTCATTTTTAATTTTC<br>GTATTAGCTTACGACGCTACACCCAGTTCCCATCTATTTTGTCACTCTTCCCTAAATAATCCTTAAAAACTCCATTTCCACCCCTCCCAGTTCCCAACTAT<br>TTTGTCCGCCCACA

a. *All landing pad sequences contain the antibiotic resistance marker.*

b. *The nucleotide numbering is based on E. coli MG1655 K-12* (NCBI U00096.3)

c. *The following colors are used for annotations. Blue: Promoters; Purple: Ribozymes; Green: Repressors, YFP and Sensor ORFs; Red: Terminators; Orange: FRT sites and att sites; yellow highlight: RBSs; Gray highlight: antibiotic resistance gene, and origin of rep; Green highlight: Transposase (Tn5, Tn7) recognition sites*

Appendix Table 5: Sequence of genetic parts used in this study

Part Name	Part Class	Part Sequence
P_{Badmc}	Promoter	AACGATCGTTGGCTGTAGCATTTTTATCCATAAGATTAGCGGATCCTACCTGACGCTTTTTATCGCAACTCTCTATATTTTTCTCCATACCCG
P_{Tet}	Promoter	AACGATCGTTGGCTGTCCCTATCAGTGATAGAGATTGACATCCCTATCAGTGATAGATATAATGAGCAC
P_{Cin}	Promoter	TGGTAGCACAAAAGTCCCTTTGTGCGTCCAAACGGACGCACGGCGCTCTAAAGCGGGTCGCGATCTTTCAGATTCGCTCCTCGCGCTTTCAGTCTTTGT<br>TTTGGCGCATGTCGTTATCGCAAAACCGCTGCACACTTTTGCGCGACATGCTCTGATCCCCCTCATCTGGGGGGGCCTATCTGAGGGAATTTCCGATCC<br>GGCTCGCCTGAACCATTCTGCT
P_{CymRC}	Promoter	TTCCGATGTAGGAGTAACAAACAGACAATCTGGTCTGTTTGTATTATGGAAAATTTTTCTGTATAATAGATTC
P_{Ttg}	Promoter	TACGCTGCCACGTGTCACCCAGCAGTATTTACAAACAACCATGAATGTAAGTATATTCCTTAGCAA
P_{Tac}	Promoter	AACGATCGTTGGCTGTGTTGACAATTAATCATCGGCTCGTATAATGTGTGGAATTGTGAGCGCTCACAATT
P_{VanCC}	Promoter	GAGCCTACCTGCCTTATTGGATCCAATTGACAGCTAGCTCAGTCCTAGGTACCATTGGATCCAAT
P_{AmtR}	Promoter	CTTGTCCAACCAAATGATTCGTTACCCTTTGACAGTTTCTATCGATCTATAGATAATGCTAGC
P_{AmeR}	Promoter	TCGTCACTAGAGGGCGATAGTGACAAACTTGACAACTCATCACTTCCTACGTAGGCTGCTAGC
P_{PhlF}	Promoter	CGACGTACGGTGGAATCTGATTCGTTACCAATTGACATGATACGAAACGTACCGTATCGTTAAGGT
P_{QacR}	Promoter	GGTATGGAAGCTATACGTTACCAATTGACAGCTAGCTCAGTCCTACTTTAGTATATAGACCGTGCGATCGGTCTATA
P_{BetI}	Promoter	AGCGCGGGTGAGAGGGATTCGTTACCAATAGACAATTGATTGGACGTTCAATATAATGCTAGC
P_{BM3R1}	Promoter	AACGAATGGCTCATGTCTGATTCGTTACCAATTGACGGAATGAACGTTCATTCCGATAATGCTAGC
P_{HlyIIR}	Promoter	ACCAGGAATCTGAACGATTCGTTACCAATTGCCATATTTAAAATTCTTGTTTAAAATGCTAGC
P_{LacI}	Promoter	TGGTGCAAAACCTTTCGCGGTATGGCATGATAGCGCCCGGAAGAGAGTCAATTCAGG
P_{Lux2}	Promoter	ATAGCTTCTTACCGGACCTGTAGGATCGTACAGGTTTACGCAAGAAAATGGTTTGTTATTTTCGAATAAA
BBa_J23105	Promoter	GAAGACGCGGAGGGAGACCGGCGCGCCTTTACGGCTAGCTCAGTCCTAGGTACTATGCTAGCAAGGT
BBa_J23104	Promoter	GCTTCAAATGCGGATTTGACAGCTAGCTCAGTCCTAGGTATTGTGCTAGC
BBa_J23119	Promoter	GAATCGCACCAAGACAGGTTTGTCCATTGACAGCTAGCTCAGTCCTAGGTATAATGCTAGC
BBa_J23101	Promoter	GATAAGTCCCTAACTTTTACAGCTAGCTCAGTCCTAGGTATTATGCTAGC
Tn7L	att sites	AACCAGATAAGTGAAATCTAGTTCCAAACTATTTTGTCATTTTTAATTTTCGTATTAGCTTACGACGCTACACCCAGTTCCCATCTATTTTGTCACTCT<br>TCCCTAAATAATCCTTAAAAACTCCATTTCCACCCCTCCCAGTTCCCAACTATTTTGTCCGCCCACA
Tn7R	att sites	TGTGGGCGGACAATAAAGTCTTAAACTGAACAAAATAGATCTAAACTATGACAATAAAGTCTTAAACTAGACAGAATAGTTGTAAACTGAAATCAGTCC<br>AGTTATGCTGTGAAAAAGCATACTGGACTTTTGTTATGGCTAAAGCAAACTCTTCATTTTCTGAAGTGCAAATTGCCCGTCGTATTAAAGAGGGGCGTG<br>G
attR2	att sites	GGACGGCGCAGAAGGGGAGTAGCTCTTCGCCCGAGAACTTCTGCAAGGCACTGCTCTTGGCT
attR5	att sites	GCACCGACCGCAGCCACAGCGTGTAGCGCTCCCAGGCCGTCCACTCCCTGATCCGGCGCTC
attR7	att sites	GTGTTATAAACCTGTGTGAGAGTTAAGTTTACATGGGCAAAGTTGATGACCGGGTCGTCCGTT
attL2	att sites	GCTCATGTATGTGTCTACGCGAGATTCTCGCCGGACCGTCGACATACTGCTCAGCTCGTC
attL5	att sites	GCACCGACCGCAGCCACAGCGTGTAGCGCTCCCAGGAGAGTTATCGACTTGCGTATTAGGG
attL7	att sites	AGACGAGAAACGTTCCGTCCGTCTGGGTCAGTTGCCTAACCTTAACTTTTACGCAGGTTCAGCTT
attB2	att sites	GACGAGCTGAGCAGTATGTCGACGGTCCGGCGAAGAGCTACTCCCCTTCTGCGCCGTCC
attP2	att sites	GCTCATGTATGTGTCTACGCGAGATTCTCGCCCGAGAACTTCTGCAAGGCACTGCTCTTGGCT
attB5	att sites	GAGCGCCGGATCAGGGAGTGGACGGCCTGGGAGCGCTACACGCTGTGGCTGCGGTCGGTGC
attP5	att sites	CCCTAATACGCAAGTCGATAACTCTCCTGGGAGCGTTGACAACTTGCGCACCCTGATCTG
attB7	att sites	AGACGAGAAACGTTCCGTCCGTCTGGGTCAGTTGGGCAAAGTTGATGACCGGGTCGTCCGTT
attP7	att sites	GTGTTATAAACCTGTGTGAGAGTTAAGTTTACATGCCTAACCTTAACTTTTACGCAGGTTCAGCTT
FRT	FRT	GAAGTTCCTATTCTCTAGAAAGTATAGGAACTTC
A1	RBS	AATGTTCCCTAATAATCAGCAAAGAGGTTACTAG
F2	RBS	ACGCTATGGACTATGTTTCAAACAGGAGCTAATAG
B3	RBS	CCAAACGAGGCCGGGAGG
E0	RBS	CCCCCCGAGGAGTAGCAC
E1	RBS	CCTTCCGAGGAGGAGCACA
P1	RBS	CTATGGACTATGTTTGAAAGGGAGAAATACTAG
P2	RBS	GGAGCTATGGACTATGTTTGAAAGGCTGAAATACTAG
P3	RBS	CTTTACGAGGGCGATCCT
H1	RBS	ACCCCCGAG
V1	RBS	AAGACCATTATAAGGTTTGAACT
BBa_B0032	RBS	TCACACAGGAAAGTACTAG
BBa_B0064	RBS	TACTAGAGAAAGAGGGGAAATACTAG
BBa_B0034	RBS	TACTAGAGAAAGAGGAGAAATACTAG
AraJ	Ribozyme	AGTGGTCGTGATCTGAAACTCGATCACCTGATGAGCTCAAGGCAGAGCGAAACCACCTCTACAAATAATTTTGTTTAA
SarJ	Ribozyme	AGACTGTCGCCGGATGTGTATCCGACCTGACGATGGCCCAAAAGGGCCGAAACAGTCCTCTACAAATAATTTTGTTTAA
RiboJ00	Ribozyme	AGCTGTCACCGGATGTGCTTTCCGGTCTGATGAGTCCGTGAGGACGAAACAGCCTCTACAAATAATTTTGTTTAA

RiboJ53	Ribozyme	AGCGGTCAACGCATGTGCTTTGCGTTCTGATGAGACAGTGATGTCGAAACCGCCTCTACAAATAATTTTGTTTAA
RiboJ54	Ribozyme	AGGGGTCAGTTGATGTGCTTTCAACTCTGATGAGTCAGTGATGACGAAACCCCCTCTACAAATAATTTTGTTTAA
RiboJ57	Ribozyme	AGAAGTCAATTAATGTGCTTTTAATTCTGATGAGTCGGTGACGACGAAACTTCCTCTACAAATAATTTTGTTTAA
RiboJ60	Ribozyme	AGTCGTCAAGTGCTGTGCTTGCACTTCTGATGAGGCAGTGATGCCGAAACGACCTCTACAAATAATTTTGTTTAA
BydvJ	Ribozyme	AGGGTGTCTCAAGGTGCGTACCTTGACTGATGAGTCCGAAAGGACGAAACACCCCTCTACAAATAATTTTGTTTAA
araC	Gene	ATGGCTGAAGCGCAAAATGATCCCCTGCTGCCGGGATACTCGTTTAATGCCCATCTGGTGGCGGGTTTAACGCCGATTGAGGCCAACGGTTATCTCGAT<br>TTTTTTATCGACCGACCGCTGGGAATGAAAGGTTATATTCTCAATCTCACCATTCGCGGTCAGGGGGTGGTGAAAAATCAGGGACGAGAATTTGTTTGC<br>CGACCGGGTGATATTTTGCTGTTCCCGCCAGGAGAGATTCATCACTACGGTCGTCATCCGGAGGCTCGCGAATGGTATCACCAGTGGGTTTACTTTCGT<br>CCGCGCGCCTACTGGCATGAATGGCTTAACTGGCCGTCAATATTTGCCAATACGGGGTTCTTTCGCCCGGATGAAGCGCACCAGCCGCATTTCAGCGAC<br>CTGTTTGGGCAAATCATTAACGCCGGGCAAGGGGAAGGGCGCTATTCGGAGCTGCTGGCGATAAATCTGCTTGAGCAATTGTTACTGCGGCGCATGGAA<br>GCGATTAACGAGTCGCTCCATCCACCGATGGATAATCGGGTACGCGAGGCTTGTCAGTACATCAGCGATCACCTGGCAGACAGCAATTTTGATATCGCC<br>AGCGTCGCACAGCATGTTTGCTTGTCGCCGTCGCGTCTGTCACATCTTTTCCGCCAGCAGTTAGGGATTAGCGTCTTAAGCTGGCGCGAGGACCAACGT<br>ATCAGCCAGGCGAAGCTGCTTTTGAGCACCACCCGGATGCCTATCGCCACCGTCGGTCGCAATGTTGGTTTTGACGATCAACTCTATTTCTCGCGGGTA<br>TTTAAAAAATGCACCGGGGCCAGCCCGAGCGAGTTCCGTGCCGGTTAAGGAAGAGAGTCAATTCAGGGTGGTGAATATGAAACCAGTAACGTTATACGA<br>TGTCGCAGAGTATGCCGGTGTCTCTTATCAGACCGTTTCCCGCGTGGTGAACCAGGCCAGCCACGTTTCTGCGAAAACGCGGGAAAAAGTGGAAGCGGC<br>GATGGCGGAGCTGAATTACATTCCCAACCGCGTGGCACAACAACTGGCGGGCAAACAGTCGTTGCTGATTGGCGTTGCCACCTCCAGTCTGGCCCTGCA<br>CGCGCCGTCGCAAATTGTCGCGGCGATTAAATCTCGCGCCGATCAACTGGGTGCCAGCGTGGTGGTGTCGATGGTAGAACGAAGCGGCGTCGAAGCCTG<br>TAAAGCGGCGGTGCACAATCTTCTCGCGCAACGCGTCAGTGGGCTGATCATTAACTATCCGCTGGATGACCAGGATGCCATTGCTGTGGAAGCTGCCTG<br>CACTAATGTTCCGGCGTTATTTCTTGATGTCTCTGACCAGACACCCATCAACAGTATTATTTTCTCCCATGAGGACGGTACGCGACTGGGCGTGGAGCA<br>TCTGGTCGCATTGGGTCACCAGCAAATCGCGCTGTTAGCG
lacI	Gene	ATGAAACCAGTAACGTTATACGATGTCGCAGAGTATGCCGGTGTCTCTTATCAGACCGTTTCCCGCGTGGTGAACCAGGCCAGCCACGTTTCTGCGAAA<br>ACGCGGGAAAAAGTGGAAGCGGCGATGGCGGAGCTGAATTACATTCCCAACCGCGTGGCACAACAACTGGCGGGCAAACAGTCGTTGCTGATTGGCGTT<br>GCCACCTCCAGTCTGGCCCTGCACGCGCCGTCGCAAATTGTCGCGGCGATTAAATCTCGCGCCGATCAACTGGGTGCCAGCGTGGTGGTGTCGATGGTA<br>GAACGAAGCGGCGTCGAAGCCTGTAAAGCGGCGGTGCACAATCTTCTCGCGCAACGCGTCAGTGGGCTGATCATTAACTATCCGCTGGATGACCAGGAT<br>GCCATTGCTGTGGAAGCTGCCTGCACTAATGTTCCGGCGTTATTTCTTGATGTCTCTGACCAGACACCCATCAACAGTATTATTTTCTCCCATGAGGAC<br>GGTACGCGACTGGGCGTGGAGCATCTGGTCGCATTGGGTCACCAGCAAATCGCGCTGTTAGCGGGCCCATTAAGTTCTGTCTCGGCGCGTCTGCGTCTG<br>GCTGGCTGGCATAAATATCTCACTCGCAATCAAATTCAGCCGATAGCGGAACGGGAAGGCGACTGGAGTGCCATGTCCGGTTTTCAACAAACCATGCAA<br>ATGCTGAATGAGGGCATCGTTCCCACTGCGATGCTGGTTGCCAACGATCAGATGGCGCTGGGCGCAATGCGCGCCATTACCGAGTCCGGGCTGCGCGTT<br>GGTGCGGATATCTCGGTAGTGGGATACGACGATACCGAAGATAGCTCATGTTATATCCCGCCGTTAACCACCATCAAACAGGATTTTCGCCTGCTGGGG<br>CAAACCAGCGTGGACCGCTTGCTGCAACTCTCTCAGGGCCAGGCGGTGAAGGGCAATCAGCTGTTGCCAGTCTCACTGGTTAAAAGAAAAACCACCCTG<br>GCGCCCAATACGCAAACCGCCTCTCCCCGCGCGTTGGCCGATTCATTAATGCAGCTGGCACGACAGGTTTCCCGACTGGAAAGCGGGCAGTGATAA
tetR	Gene	ATGTCCAGATTAGATAAAAGTAAAGTGATTAACAGCGCATTAGAGCTGCTTAATGAGGTCGGAATCGAAGGTTTAACAACCCGTAAACTCGCCCAGAAG<br>CTAGGTGTAGAGCAGCCTACATTGTATTGGCATGTAAAAAATAAGCGGGCTTTGCTCGACGCCTTAGCCATTGAGATGTTAGATAGGCACCATACTCAC<br>TTTTGCCCTTTAGAAGGGGAAAGCTGGCAAGATTTTTTACGTAATAACGCTAAAAGTTTTAGATGTGCTTTACTAAGTCATCGCGATGGAGCAAAAGTA<br>CATTTAGGTACACGGCCTACAGAAAAACAGTATGAAACTCTCGAAAATCAATTAGCCTTTTTATGCCAACAAGGTTTTTCACTAGAGAATGCATTATAT<br>GCACTCAGCGCTGTGGGGCATTTTACTTTAGGTTGCGTATTGGAAGATCAAGAGCATCAAGTCGCTAAAGAAGAAAGGGAAACACCTACTACTGATAGT<br>ATGCCGCCATTATTACGACAAGCTATCGAATTATTTGATCACCAAGGTGCAGAGCCAGCCTTCTTATTCGGCCTTGAATTGATCATATGCGGATTAGAA<br>AAACAACTTAAATGTGAAAGTGGGTCCTAA
cinR^{AM}	Gene	ATGATTGAGAATACCTATAGCGAAAAGTTCGAGTCCGCGTTCGAACAGATCAAAGCGGCGGCCAACGTGGATGCCGCCATCCGTATTCTCCAGGCGGAA<br>TATAACCTCGATTTCGTCACCTACCATCTCGCCCAGACAATCGCGAGCAAGATCGATTCGCCCTTCGTGCGCACCACCTATCCGGATGCCTGGGTTTCC<br>CGTTACCTCCTCAACTGCTATGTGAAGGTCGATCCGATCATCAAGCAGGGCTTCGAACGCCAGCTGCCCTTCGACTGGAGCGAGGTCGAACCGACGCCG<br>GAGGCCTATGCCATGCTGGTCGACGCCCAGAAACACGGCATCGATGACAATGGCTACTCCATCCCCGTCGCCGACAAGGCGCAGCGCCGCGCCCTGCTG<br>TCGCTGAATGCCCATATACCGGCCGACGAATGGACCGAGCTCGTGCGCCGCTGCCGCAATGAGTGGATCGAGATCGCCCATCTGATCCACCGCAAGGCC<br>GTATATGAGCTGCATGGCGAAAACGATCCGGTGCCGGCATTGTCGCCGCGCGAGATCGAGTGTCTGCACTGGACCGCCCTCGGCAAGGATTACAAGGAT<br>ATTTCGGTCATCCTGGGCATATCAGAGCATACCACACGCGATTACCTGAAAACCGCCCGCTTCAGGCTCGGCTGCACCACGATCTCGGCCGCCGCGTCG<br>CGGGCTGTTCAATTGCGCATCATCAATCCCTATAGGATCCGCATGACGCGACGTAATTGGTAATAG
vanR^{AM}	Gene	ATGGACATGCCTCGTATTAAACCGGGTCAGCGTGTTATGATGGCACTGCGTAAAATGATTGCAAGCGGTGAAATCAAAAGTGGTGAACGTATTGCAGAA<br>ATTCCGACCGCAGCAGCACTGGGTGTTAGCCGTATGCCGGTTCGTATCGCACTGCGTTCACTGGAACAAGAAGGTCTGGTTGTTCGTCTGGGTGCACGT<br>GGTTATGCAGCCCGTGGTGTTAGCAGCGATCAGATTCGTGATGCAATTGAAGTTCGTGGTGTTCTGGAAGGTTTTGCAGCACGTCGTCTGGCAGAACGT<br>GGTATGACCGCAGAAACCCATGCACGTTTTGTTGTACTGATTGCAGAAGGTGAAGCACTGTTTGCAGCCGGTCGCCTGAATGGTGAAGATCTGGATCGT<br>TATGCCGCATATAATCAGGCATTTCATGATACCCTGGTTAGCGCAGCAGGTAATGGTGCAGTTGAAAGCGCACTGGCACGTAATGGTTTTGAACCGTTT<br>GCAGCAGCCGGTGCACTGGCCCTGGATCTGATGGACCTGTCTGCCGAATATGAACATCTGCTGGCAGCACATCGTCAGCATCAGGCAGTTCTGGATGCA<br>GTTAGCTGTGGTGATGCCGAAGGTGCAGAACGTATTATGCGTGATCATGCACTGGCAGCAATTCGTAATGCAAAAGTTTTTGAAGCAGCAGCAAGCGCA<br>GGCGCACCGCTGGGTGCAGCATGGTCAATTCGTGCAGATTGA
ttgR^{AM}	Gene	ATGGTTCGTCGTACCAAAGAAGAGGCACAAGAAACCCGTGCACAGATTATTGAAGCAGCAGAACGTGCATTCTATAAACGTGGTGTTGCACGTACCACC<br>CTGGCAGATATTGCAGAACTGGCAGGCGTTACCCGTGGTGCAATTTATTGGCATTTTAACAACAAAGCCGAACTGGTTCAGGCACTGCTGGATAGCCTG<br>CATGAAACCCATGATCATCTGGCACGTGCAAGCGAAAGCGAAGATGAAGTTGATCCGCTGGGTTGTATGCGTAAACTGCTGCTGCAGGTTTTTAATGAA<br>CTGGTTCTGGATGCACGTACCCGTCGTATTAATGAAATCCTGCATCACAAATGCGAGTTCACCGATGATATGTGTGAAATTCGTCAGCAGCATCAGAGC<br>GCAGTTCTGGATTGTCATAAAGGTATTACCCTGACACTGGCAAATGTAGTTCGTCGTGGTCAGCTGCCTGGTGAACTGGATGCAGAACGTGCCGCAGTT<br>GCAATGTTTGCCTATGTTGATGGTCTGATTCGTCGTTGGCTGCTGCTGCCGGATAGCGTTGATCTGCTGGGTGATGTTGAAAAATGGGTTGATACCGGT<br>CTGGATATGCTGCGTCTGAGTCCGGCACTGCGTAAATAA
cymR^{AM}	Gene	ATGAGCCCGAAACGTCGTACCCAGGCAGAACGTGCAATGGAAACCCAGGGTAAACTGATTGCAGCAGCACTGGGTGTTCTGCGTGAAAAAGGTTATGCA<br>GGTTTTCGTATTGCAGATGTTCCGGGTGCAGCCGGTGTTAGCCGTGGTGCACAGAGCCATCATTTTCCGACCAAACTGGAACTGCTGCTGGCAACCTTT<br>GAATGGCTGTATGAGCAGATTACCGAACGTAGCCGTGCACGTCTGGCAAAACTGAAACCGGAAGATGATGTTATTCAGCAGATGCTGGATGATGCAGCA<br>GAATTTTTTCTGGATGATGATTTTAGCATCGGCCTGGATCTGATTGTTGCAGCAGATCGTGATCCGGCACTGCGTGAAGGTATTCAGCGTACCGTTGAA<br>CGTAATCGTTTTGTTGTTGAAGATATGTGGCTGGGTGTGCTGGTGAGCCGTGGTCTGAGCCGTGATGATGCCGAAGATATTCTGTGGCTGATTTTTAAC<br>AGCGTTCGTGGTCTGGTAGTTCGTAGCCTGTGGCAGAAAGATAAAGAACGTTTTGAACGTGTGCGTAATAGCACCCTGGAAATTGCACGTGAACGTTAT<br>GCAAAATTCAAACGTTGA
luxR	Gene	ATGAAAAACATAAATGCCGACGACACATACAGAATAATTAATAAAATTAAAGCTTGTAGAAGCAATAATGATATTAATCAATGCTTATCTGATATGACT<br>AAAATGGTACATTGTGAATATTATTTACTCGCAATCATTTATCCTCATTATATGGTTAAATCCGATATTTCAATCCTAGATAATTACCCTAAAAAATGG<br>AGGCAATATTATGATGACGCTAATTTAATAAAATATGATCCTATAGTAGATTATTCTAACTCCAATCATTCACCAATTAATTGGAATATATTTGAAAAC<br>AATGCTGTAAATAAAAAATCTCCAAATGTAATTAAAGAAGCGAAAACATCAGGTCTTATCACTGGGTTTAGTTTCCCTATTCATACGGCTAACAATGGC<br>TTCGGAATGCTTAGTTTTGCACATTCAGAAAAAGACAACTATATAGATAGTTTATTTTTACATGCGTGTATGAACATACCATTAATTGTTCCTTCTCTA<br>GTTGATAATTATCGAAAAATAAATATAGCAAATAATAAATCAAACAACGATTTAACCAAAAGAGAAAAAGAATGTTTAGCGTGGGCATGCGAAGGAAAA<br>AGCTCTTGGGATATTTCAAAAATATTAGGTTGCAGTGAGCGTACTGTCACTTTCCATTTAACCAATGCGCAAATGAAACTCAATACAACAAACCGCTGC<br>CAAAGTATTTCTAAAGCAATTTTAACAGGAGCAATTGATTGCCCATACTTTAAAAATTAA
tnpA	Gene	ATGGTAACGTTCATGATTACCAGTGCACTGCATCGTGCGGCGGATTGGGCGAAAAGCGTGTTTTCTAGTGCTGCGCTGGGTGATCCGCGTCGTACCGCG<br>CGTCTGGTGAATGTTGCGGCGCAACTGGCCAAATATAGCGGCAAAAGCATTACCATTAGCAGCGAAGGCAGCAAAGCCATGCAGGAAGGCGCGTATCGT<br>TTTATTCGTAATCCGAACGTGAGCGCGGAAGCGATTCGTAAAGCGGGTGCCATGCAGACCGTGAAACTGGCCCAGGAATTTCCGGAACTGCTGGCAATT<br>GAAGATACCACCTCTCTGAGCTATCGTCATCAGGTGGCGGAAGAACTGGGCAAACTGGGTAGCATTCAGGATAAAAGCCGTGGTTGGTGGGTGCATAGC<br>GTGCTGCTGCTGGAAGCGACCACCTTTCGTACCGTGGGCCTGCTGCATCAAGAATGGTGGATGCGTCCGGATGATCCGGCGGATGCGGATGAAAAAGAA<br>AGCGGCAAATGGCTGGCCGCTGCTGCAACTTCGCGTCTGAGAATGGGCAGCATGATGAGCAACGTGATTGCGGTGTGCGATCGTGAAGCGGATATTCAT<br>GCGTATCTGCAAGATAAACTGGCCCATAACGAACGTTTTGTGGTGCGTAGCAAACATCCGCGTAAAGATGTGGAAAGCGGCCTGTATCTGTATGATCAC<br>CTGAAAAACCAGCCGGAACTGGGCGGCTATCAGATTAGCATTCCGCAGAAAGGCGTGGTGGATAAACGTGGCAAACGTAAAAACCGTCCGGCGCGTAAA<br>GCGAGCCTGAGCCTGCGTAGCGGCCGTATTACCCTGAAACAGGGCAACATTACCCTGAACGCGGTGCTGGCCGAAGAAATTAATCCGCCGAAAGGCGAA<br>ACCCCGCTGAAATGGCTGCTGCTGACCAGCGAGCCGGTGGAAAGTCTGGCCCAAGCGCTGCGTGTGATTGATATTTATACCCATCGTTGGCGCATTGAA<br>GAATTTCACAAAGCGTGGAAAACGGGTGCGGGTGCGGAACGTCAGCGTATGGAAGAACCGGATAACCTGGAACGTATGGTGAGCATTCTGAGCTTTGTG<br>GCGGTGCGTCTGCTGCAACTGCGTGAATCTTTTACTCCGCCGCAAGCACTGCGTGCGCAGGGCCTGCTGAAAGAAGCGGAACACGTTGAAAGCCAGAGC<br>GCGGAAACCGTGCTGACCCCGGATGAATGCCAACTGCTGGGCTATCTGGATAAAGGCAAACGCAAACGCAAAGAAAAAGCGGGCAGCCTGCAATGGGCG<br>TATATGGCGATTGCGCGTCTGGGCGGCTTTATGGATAGCAAACGTACCGGCATTGCGAGCTGGGGTGCGCTGTGGGAAGGTTGGGAAGCGCTGCAAAGC<br>AAACTGGATGGCTTTCTGGCCGCGAAAGACCTGATGGCGCAGGGCATTAAAATCTAA

specR	Gene	ATGGCTTGTTATGACTGTTTTTTTGGGGTACAGTCTATGCCTCGGGCATCCAAGCAGCAAGCGCGTTACGCCGTGGGTCGATGTTTGATGTTATGGAGC<br>AGCAACGATGTTACGCAGCAGGGCAGTCGCCCTAAAACAAAGTTAAACATCATGAGGGAAGCGGTGATCGCCGAAGTATCGACTCAACTATCAGAGGTA<br>GTTGGCGTCATCGAGCGCCATCTCGAACCGACGTTGCTGGCCGTACATTTGTACGGCTCCGCAGTGGATGGCGGCCTGAAGCCACACAGTGATATTGAT<br>TTGCTGGTTACGGTGACCGTAAGGCTTGATGAAACAACGCGGCGAGCTTTGATCAACGACCTTTTGGAAACTTCGGCTTCCCCTGGAGAGAGCGAGATT<br>CTCCGCGCTGTAGAAGTCACCATTGTTGTGCACGACGACATCATTCCGTGGCGTTATCCAGCTAAGCGCGAACTGCAATTTGGAGAATGGCAGCGCAAT<br>GACATTCTTGCAGGTATCTTCGAGCCAGCCACGATCGACATTGATCTGGCTATCTTGCTGACAAAAGCAAGAGAACATAGCGTTGCCTTGGTAGGTCCA<br>GCGGCGGAGGAACTCTTTGATCCGGTTCCTGAACAGGATCTATTTGAGGCGCTAAATGAAACCTTAACGCTATGGAACTCGCCGCCCGACTGGGCTGGC<br>GATGAGCGAAATGTAGTGCTTACGTTGTCCCGCATTTGGTACAGCGCAGTAACCGGCAAAATCGCGCCGAAGGATGTCGCTGCCGACTGGGCAATGGAG<br>CGCCTGCCGGCCCAGTATCAGCCCGTCATACTTGAAGCTAGACAGGCTTATCTTGGACAAGAAGAAGATCGCTTGGCCTCGCGCGCAGATCAGTTGGAA<br>GAATTTGTCCACTACGTGAAAGGCGAGATCACCAAGGTAGTCGGCAAATAA
kanR	Gene	ATGAGCCATATTCAACGGGAAACGTCTTGCTCTAGGCCGCGATTAAATTCCAACATGGATGCTGATTTATATGGGTATAAATGGGCTCGCGATAATGTC<br>GGGCAATCAGGTGCGACAATCTATCGATTGTATGGGAAGCCCGATGCGCCAGAGTTGTTTCTGAAACATGGCAAAGGTAGCGTTGCCAATGATGTTACA<br>GATGAGATGGTCAGACTAAACTGGCTGACGGAATTTATGCCTCTTCCGACCATCAAGCATTTTATCCGTACTCCTGATGATGCATGGTTACTCACCACT<br>GCGATCCCCGGGAAAACAGCATTCCAGGTATTAGAAGAATATCCTGATTCAGGTGAAAATATTGTTGATGCGCTGGCAGTGTTCCTGCGCCGGTTGCAT<br>TCGATTCCTGTTTGTAATTGTCCTTTTAACAGCGACCGCGTATTTCGTCTCGCTCAGGCGCAATCACGAATGAATAACGGTTTGGTTGATGCGAGTGAT<br>TTTGATGACGAGCGTAATGGCTGGCCTGTTGAACAAGTCTGGAAAGAAATGCATAAACTTTTGCCATTCTCACCGGATTCAGTCGTCACTCATGGTGAT<br>TTCTCACTTGATAACCTTATTTTTGACGAGGGGAAATTAATAGGTTGTATTGATGTTGGACGAGTCGGAATCGCAGACCGATACCAGGATCTTGCCATC<br>CTATGGAACTGCCTCGGTGAGTTTTCTCCTTCATTACAGAAACGGCTTTTTCAAAAATATGGTATTGATAATCCTGATATGAATAAATTGCAGTTTCAT<br>TTGATGCTCGATGAGTTTTTCTAA
cmR	Gene	ATGGAGAAAAAAATCACTGGATATACCACCGTTGATATATCCCAATGGCATCGTAAAGAACATTTTGAGGCATTTCAGTCAGTTGCTCAATGTACCTAT<br>AACCAGACCGTTCAGCTGGATATTACGGCCTTTTTAAAGACCGTAAAGAAAAATAAGCACAAGTTTTATCCGGCCTTTATTCACATTCTTGCCCGCCTG<br>ATGAATGCTCATCCGGAATTTCGTATGGCAATGAAAGACGGTGAGCTGGTGATATGGGATAGTGTTCACCCTTGTTACACCGTTTTCCATGAGCAAACT<br>GAAACGTTTTCATCGCTCTGGAGTGAATACCACGACGATTTCCGGCAGTTTCTACACATATATTCGCAAGATGTGGCGTGTTACGGTGAAAACCTGGCC<br>TATTTCCCTAAAGGGTTTATTGAGAATATGTTTTTCGTCTCAGCCAATCCCTGGGTGAGTTTCACCAGTTTTGATTTAAACGTGGCCAATATGGACAAC<br>TTCTTCGCCCCCGTTTTCACCATGGGCAAATATTATACGCAAGGCGACAAGGTGCTGATGCCGCTGGCGATTCAGGTTCATCATGCCGTTTGTGATGGC<br>TTCCATGTCGGCAGAATGCTTAATGAATTACAACAGTACTGCGATGAGTGGCAGGGCGGGGCGTAA
tetA	Gene	ATGCCGGTACTGCCGGGCCTCTTGCGGGATATCGTCCATTCCGACAGCATCGCCAGTCACTATGGCGTGCTGCTAGCGCTATATGCGTTGATGCAATTT<br>CTATGCGCACCCGTTCTCGGAGCACTGTCCGACCGCTTTGGCCGCCGCCCAGTCCTGCTCGCTTCGCTACTTGGAGCCACTATCGACTACGCGATCATG<br>GCGACCACACCCGTCCTGTGGATTCTCTACGCCGGACGCATCGTGGCCGGCATCACCGGCGCCACAGGTGCGGTTGCTGGCGCCTATATCGCCGACATC<br>ACCGATGGGGAAGATCGGGCTCGCCACTTCGGGCTCATGAGCGCTTGTTTCGGCGTGGGTATGGTGGCAGGCCCCGTGGCCGGGGGACTGTTGGGCGCC<br>ATCTCCCTGCACGCACCATTCCTTGCGGCGGCGGTGCTCAACGGCCTCAACCTACTACTGGGCTGCTTCCTAATGCAGGAGTCGCATAAGGGAGAGCGC<br>CGTCCGATGCCCTTGAGAGCCTTCAACCCAGTCAGCTCCTTCCGGTGGGCGCGGGGCATGACCATTGTGGCCGCACTTATGACTGTCTTCTTTATCATG<br>CAACTCGTAGGACAGGTGCCGGCAGCGCTCTGGGTCATTTTCGGCGAGGACCGCTTTCGCTGGAGCGCGACGATGATCGGCCTGTCGCTTGCGGTATTC<br>GGAATCTTGCACGCCCTCGCTCAAGCCTTCGTCACTGGTCCCGCCACCAAACGTTTCGGCGAGAAGCAGGCCATTATCGCCGGCATGGCGGCCGACGCG<br>CTGGGCTACGTCTTGCTGGCGTTCGCGACGCGAGGCTGGATGGCCTTCCCCATTATGATTCTTCTCGCTTCCGGCGGCATCGGGATGCCCGCGTTGCAG<br>GCCATGCTGTCCAGGCAGGTAGATGACGACCATCAGGGACAGCTTCAAGGATCGCTCGCGGCTCTTACCAGCCTAACTTCGATCACTGGACCGCTGATC<br>GTCACGGCGATTTATGCCGCCTCGGCGAGCACATGGAACGGGTTGGCATGGATTGTAGGCGCCGCCCTATACCTTGTCTGCCTCCCCGCGTTGCGTCGC<br>GGTGCATGGAGCCGGGCCACCTCGACCTGA
ampR	Gene	ATGAGCACTTTTAAAGTTCTGCTATGTGGCGCGGTATTATCCCGTATTGACGCCGGGCAAGAGCAACTCGGTCGCCGCATACACTATTCTCAGAATGAC<br>TTGGTTGAGTACTCACCAGTCACAGAAAAGCATCTTACGGATGGCATGACAGTAAGAGAATTATGCAGTGCTGCCATAACCATGAGTGATAACACTGCG<br>GCCAACTTACTTCTGACAACGATCGGAGGACCGAAGGAGCTAACCGCTTTTTTGCACAACATGGGGGATCATGTAACTCGCCTTGATCGTTGGGAACCG<br>GAGCTGAATGAAGCCATACCAAACGACGAGCGTGACACCACGATGCCTGTAGCAATGGCAACAACGTTGCGCAAACTATTAACTGGCGAACTACTTACT<br>CTAGCTTCCCGGCAACAATTAATAGACTGGATGGAGGCGGATAAAGTTGCAGGACCACTTCTGCGCTCGGCCCTTCCGGCTGGCTGGTTTATTGCTGAT<br>AAATCTGGAGCCGGTGAGCGTGGGTCTCGCGGTATCATTGCAGCACTGGGGCCAGATGGTAAGCCCTCCCGTATCGTAGTTATCTACACGACGGGGAGT<br>CAGGCAACTATGGATGAACGAAATAGACAGATCGCTGAGATAGGTGCCTCACTGATTAAGCATTGGTAA
gfp	Gene	ATGCGTAAAGGAGAAGAACTTTTCACTGGAGTTGTCCCAATTCTTGTTGAATTAGATGGTGATGTTAATGGGCACAAATTTTCTGTCAGTGGAGAGGGT<br>GAAGGTGATGCAACATACGGAAAACTTACCCTTAAATTTATTTGCACTACTGGAAAACTACCTGTTCCATGGCCAACACTTGTCACTACTTTCGGTTAT<br>GGTGTTCAATGCTTTGCGAGATACCCAGATCATATGAAACAGCATGACTTTTTCAAGAGTGCCATGCCCGAAGGTTATGTACAGGAAAGAACTATATTT<br>TTCAAAGATGACGGGAACTACAAGACACGTGCTGAAGTCAAGTTTGAAGGTGATACCCTTGTTAATAGAATCGAGTTAAAAGGTATTGATTTTAAAGAA<br>GATGGAAACATTCTTGGACACAAATTGGAATACAACTATAACTCACACAATGTATACATCATGGCAGACAAACAAAAGAATGGAATCAAAGTTAACTTC<br>AAAATTAGACACAACATTGAAGATGGAAGCGTTCAACTAGCAGACCATTATCAACAAAATACTCCAATTGGCGATGGCCCTGTCCTTTTACCAGACAAC<br>CATTACCTGTCCACACAATCTGCCCTTTCGAAAGATCCCAACGAAAAGAGAGACCACATGGTCCTTCTTGAGTTTGTAACAGCTGCTGGGATTACACAT<br>GGCATGGATGAACTATACAAATAA
yfp	Gene	ATGGTGAGCAAGGGCGAGGAGCTGTTCACCGGGGTGGTGCCCATCCTGGTCGAGCTGGACGGCGACGTAAACGGCCACAAGTTCAGCGTGTCCGGCGAG<br>GGCGAGGGCGATGCCACCTACGGCAAGCTGACCCTGAAGTTCATCTGCACCACAGGCAAGCTGCCCGTGCCCTGGCCCACCCTCGTGACCACCTTCGGC<br>TACGGCCTGCAATGCTTCGCCCGCTACCCCGACCACATGAAGCTGCACGACTTCTTCAAGTCCGCCATGCCCGAAGGCTACGTCCAGGAGCGCACCATC<br>TTCTTCAAGGACGACGGCAACTACAAGACCCGCGCCGAGGTGAAGTTCGAGGGCGACACCCTGGTGAACCGCATCGAGCTGAAGGGCATCGACTTCAAG<br>GAGGACGGCAACATCCTGGGGCACAAGCTGGAGTACAACTACAACAGCCACAACGTCTATATCATGGCCGACAAGCAGAAGAACGGCATCAAGGTGAAC<br>TTCAAGATCCGCCACAACATCGAGGACGGCAGCGTGCAGCTCGCCGACCACTACCAGCAGAACACCCCCAATCGGCGACGGCCCCGTGCTGCTGCCCGAC<br>AACCACTACCTTAGCTACCAGTCCGCCCTGAGCAAAGACCCCAACGAGAAGCGCGATCACATGGTCCTGCTGGAGTTCGTGACCGCCGCCGGGATCACT<br>CTCGGCATGGACGAGCTGTACAAGTAA
rfp	Gene	ATGGCTTCCTCCGAAGACGTTATCAAAGAGTTCATGCGTTTCAAAGTTCGTATGGAAGGTTCCGTTAACGGTCACGAGTTCGAAATCGAAGGTGAAGGT<br>GAAGGTCGTCCGTACGAAGGTACCCAGACCGCTAAACTGAAAGTTACCAAAGGTGGTCCGCTGCCGTTCGCTTGGGACATCCTGTCCCCGCAGTTCCAG<br>TACGGTTCCAAAGCTTACGTTAAACACCCGGCTGACATCCCGGACTACCTGAAACTGTCCTTCCCGGAAGGTTTCAAATGGGAACGTGTTATGAACTTC<br>GAAGACGGTGGTGTTGTTACCGTTACCCAGGACTCCTCCCTGCAAGACGGTGAGTTCATCTACAAAGTTAAACTGCGTGGTACCAACTTCCCGTCCGAC<br>GGTCCGGTTATGCAGAAAAAAACCATGGGTTGGGAAGCTTCCACCGAACGTATGTACCCGGAAGACGGTGCTCTGAAAGGTGAAATCAAAATGCGTCTG<br>AAACTGAAAGACGGTGGTCACTACGACGCTGAAGTTAAAACCACCTACATGGCTAAAAAACCGGTTCAGCTGCCGGGTGCTTACAAAACCGACATCAAA<br>CTGGACATCACCTCCCACAACGAAGACTACACCATCGTTGAACAGTACGAACGTGCTGAAGGTCGTCACTCCACCGGTGCTTAA
mCherry	Gene	ATGGTTTCTAAGGGCGAAGAAGATAATATGGCCATTATCAAGGAGTTTATGCGCTTTAAAGTGCACATGGAGGGCTCAGTAAACGGTCACGAATTTGAA<br>ATCGAAGGAGAAGGCGAGGGCCGTCCGTACGAGGGGACTCAGACGGCGAAGCTCAAAGTCACAAAAGGTGGCCCATTGCCATTCGCCTGGGATATTCTG<br>TCGCCGCAGTTCATGTATGGCAGCAAAGCGTACGTAAAACACCCGGCAGATATTCCGGATTATCTGAAATTATCTTTCCCGGAAGGTTTCAAATGGGAA<br>CGCGTTATGAATTTCGAGGACGGGGGCGTAGTGACGGTCACGCAGGATAGTAGCCTGCAGGACGGCGAATTCATTTACAAAGTCAAGCTGCGTGGCACA<br>AATCTTCCGTCTGATGGCCCGGTGATGCAGAAGAAAACCATGGGTTGGGAGGCTTCCAGCGAACGCATGTATCCCGAAGATGGAGCATTGAAAGGCGAA<br>ATCAAACAGCGCCTGAAATTAAAAGACGGCGGACATTACGACGCCGAGGTCAAGACCACTTACAAAGCGAAAAAACCAGTTCAGCTGCCAGGCGCCTAC<br>AACGTTAATATCAAGCTGGATATTACCTCGCATAATGAAGATTATACTATCGTGGAACAATACGAGCGCGCCGAAGGCCGCCACTCCACTGGTGGTATG<br>GATGAATTATATAAA
amtR	Gene	ATGGCAGGCGCAGTTGGTCGTCCGCGTCGTAGTGCACCGCGTCGTGCAGGTAAAAATCCGCGTGAAGAAATTCTGGATGCAAGCGCAGAACTGTTTACC<br>CGTCAGGGTTTTGCAACCACCAGTACCCATCAGATTGCAGATGCAGTTGGTATTCGTCAGGCAAGCCTGTATTATCATTTTCCGAGCAAAACCGAAATC<br>TTTCTGACCCTGCTGAAAAGCACCGTTGAACCGAGCACCGTTCTGGCAGAAGATCTGAGCACCCTGGATGCAGGTCCGGAAATGCGTCTGTGGGCAATT<br>GTTGCAAGCGAAGTTCGTCTGCTGCTGAGCACCAAATGGAATGTTGGTCGTCTGTATCAGCTGCCGATTGTTGGTAGCGAAGAATTTGCAGAATATCAT<br>AGCCAGCGTGAAGCACTGACCAATGTTTTTCGTGATCTGGCAACCGAAATTGTTGGTGATGATCCGCGTGCAGAACTGCCGTTTCATATTACCATGAGC<br>GTTATTGAAATGCGTCGCAATGATGGTAAAATTCCGAGTCCGCTGAGCGCAGATAGCCTGCCGGAAACCGCAATTATGCTGGCAGATGCAAGCCTGGCA<br>GTTCTGGGTGCACCGCTGCCTGCAGATCGTGTTGAAAAAACCCTGGAACTGATTAAACAGGCAGATGCAAAATAA
ameR	Gene	ATGAACAAAACCATTGATCAGGTGCGTAAAGGTGATCGTAAAAGCGATCTGCCGGTTCGTCGTCGTCCGCGTCGTAGTGCCGAAGAAACCCGTCGTGAT<br>ATTCTGGCAAAAGCCGAAGAACTGTTTCGTGAACGTGGTTTTAATGCAGTTGCCATTGCAGATATTGCAAGCGCACTGAATATGAGTCCGGCAAATGTG<br>TTTAAACATTTTAGCAGCAAAAACGCACTGGTTGATGCAATTGGTTTTGGTCAGATTGGTGTTTTTGAACGTCAGATTTGTCCGCTGGATAAAAGCCAT<br>GCACCGCTGGATCGTCTGCGTCATCTGGCACGTAATCTGATGGAACAGCATCATCAGGATCATTTCAAACACATACGGGTTTTTATTCAGATCCTGATG<br>ACCGCCAAACAGGATATGAAATGTGGCGATTATTACAAAAGCGTGATTGCAAAACTGCTGGCCGAAATTATTCGTGATGGTGTTGAAGCAGGTCTGTAT<br>ATTGCAACCGATATTCCGGTTCTGGCAGAAACCGTTCTGCATGCACTGACCAGCGTTATTCATCCGGTTCTGATTGCACAAGAAGATATTGGTAATCTG<br>GCAACCCGTTGTGATCAGCTGGTTGATCTGATTGATGCAGGTCTGCGTAATCCGCTGGCAAAATAA
ameRs	Gene	ATGAACAAAACCATTGATCAGGTGCGTAAAGGTGATCGTAAAAGCGATCTGCCGGTTCGTCGTCGTCCGCGTCATAGTGCCGAAGAAACCCGTCGTGAT<br>ATTCTGGCAAAAGCCGAAGAACTGTTTAGTGAACGTGGTTTTAATGCAGTTGCCATTGCAGATATTGCAAGCGCACTGAATATGAGTCCGGCAAATGTG<br>TTTAAACATTTTAGCAGCAAAAACGCACTGGTTGATGCAATTGGTTTTGGTCAGATTGGTGTTTTTGAACGTCAGATTTGTCCGCTGGATAAAAGCCAT<br>GCACCGCTGGATCGTCTGCGTCATCTGGCACGTAATCTGATGGAACAGCATCATCAGGATCATTTCAAACACATACGGGTTTTTATTCAGATCCTGATG

		ACCGCCAAACAGGATATGAAATGTGGCGATTATTACAAAAGCGTGATTGCAAAACTGCTGGCCGAAATTATTCGTGATGGTGTTGAAGCAGGTCTGTAT<br>ATTGCAACCGATATTCCGGTTCTGGCAGAAACCGTTCTGCATGCACTGACCAGCGTTATTCATCCGGTTCTGATTGCACAAGAAGATATTGGTAATCTG<br>GCAACCCGTTGTGATCAGCTGGTTGATCTGATTGATGCAGGTCTGCGTAATCCGCTGGCAAAATAA
phlF	Gene	ATGGCACGTACCCCGAGCCGTAGCAGCATTGGTAGCCTGCGTAGTCCGCATACCCATAAAGCAATTCTGACCAGCACCATTGAAATCCTGAAAGAATGT<br>GGTTATAGCGGTCTGAGCATTGAAAGCGTTGCACGTCGTGCCGGTGCAAGCAAACCGACCATTTATCGTTGGTGGACCAATAAAGCAGCACTGATTGCC<br>GAAGTGTATGAAAATGAAAGCGAACAGGTGCGTAAATTTCCGGATCTGGGTAGCTTTAAAGCCGATCTGGATTTTCTGCTGCGTAATCTGTGGAAAGTT<br>TGGCGTGAAACCATTTGTGGTGAAGCATTTCGTTGTGTTATTGCAGAAGCACAGCTGGACCCTGCAACCCTGACCCAGCTGAAAGATCAGTTTATGGAA<br>CGTCGTCGTGAGATGCCGAAAAAACTGGTTGAAAATGCCATTAGCAATGGTGAACTGCCGAAAGATACCAATCGTGAACTGCTGCTGGATATGATTTTT<br>GGTTTTTGTTGGTATCGCCTGCTGACCGAACAGCTGACCGTTGAACAGGATATTGAAGAATTTACCTTCCTGCTGATTAATGGTGTTTGTCCGGGTACA<br>CAGCGTTAA
qacR	Gene	ATGAACCTGAAAGATAAAATTCTGGGCGTTGCCAAAGAACTGTTTATCAAAAATGGCTATAACGCAACCACCACCGGTGAAATTGTTAAACTGAGCGAA<br>AGCAGCAAAGGCAATCTGTATTATCACTTTAAAACCAAAGAGAACCTGTTTCTGGAAATCCTGAACATCGAAGAAAGCAAATGGCAAGAGCAGTGGAAA<br>AAAGAACAAATCAAATGCAAAACCAACCGCGAGAAATTCTATCTGTATAATGAACTGAGCCTGACCACCGAATATTACTATCCGCTGCAGAATGCCATC<br>ATCGAGTTTTATACCGAGTACTATAAAACCAACAGCATCAACGAGAAAATGAACAAACTGGAAAACAAATACATCGATGCCTACCACGTGATCTTTAAA<br>GAAGGTAATCTGAACGGCGAATGGTGCATTAATGATGTTAATGCCGTGAGCAAAATTGCAGCAAATGCCGTTAATGGCATTGTTACCTTTACCCATGAG<br>CAGAATATCAACGAACGCATTAAACTGATGAACAAATTCAGCCAGATCTTTCTGAATGGCCTGAGCAAATAA
betI	Gene	ATGCCGAAACTGGGTATGCAGAGCATTCGTCGTCGTCAGCTGATTGATGCAACCCTGGAAGCAATTAATGAAGTTGGTATGCATGATGCAACCATTGCA<br>CAGATTGCACGTCGTGCCGGTGTTAGCACCGGTATTATTAGCCATTATTTCCGCGATAAAAACGGTCTGCTGGAAGCAACCATGCGTGATATTACCAGC<br>CAGCTGCGTGATGCAGTTCTGAATCGTCTGCATGCACTGCCGCAGGGTAGCGCAGAACAGCGTCTGCAGGCAATTGTTGGTGGTAATTTTGATGAAACC<br>CAGGTTAGCAGCGCAGCAATGAAAGCATGGCTGGCATTTTGGGCAAGCAGCATGCATCAGCCGATGCTGTATCGTCTGCAGCAGGTTAGCAGTCGTCGT<br>CTGCTGAGCAATCTGGTTAGCGAATTTCGTCGTGAACTGCCTCGTGAACAGGCACAAGAGGCAGGTTATGGTCTGGCAGCACTGATTGATGGTCTGTGG<br>CTGCGTGCAGCACTGAGCGGTAAACCGCTGGATAAAACCCGTGCAAATAGCCTGACCCGTCATTTTATCACCCAGCATCTGCCGACCGATTAA
hlyIIR	Gene	ATGAAATACATCCTGTTTGAGGTGTGCGAAATGGGTAAAAGCCGTGAACAGACCATGGAAAATATTCTGAAAGCAGCCAAAAAGAAATTCGGCGAACGT<br>GGTTATGAAGGCACCAGCATTCAAGAAATTACCAAAGAAGCCAAAGTTAACGTTGCAATGGCCAGCTATTACTTTAATGGCAAAGAGAACCTGTACTAC<br>GAGGTGTTCAAAAAATACGGTCTGGCAAATGAACTGCCGAACTTTCTGGAAAAAAACCAGTTTAATCCGATTAATGCCCTGCGTGAATATCTGACCGTT<br>TTTACCACCCACATTAAAGAAAATCCGGAAATTGGCACCCTGGCCTATGAAGAAATTATCAAAGAAAGCGCACGCCTGGAAAAAATCAAACCGTATTTT<br>ATCGGCAGCTTCGAACAGCTGAAAGAAATTCTGCAAGAGGGTGAAAAACAGGGTGTGTTTCACTTTTTTAGCATCAACCATACCATCCATTGGATTACC<br>AGCATTGTTCTGTTTCCGAAATTCAAAAAATTCATCGATAGCCTGGGTCCGAATGAAACCAATGATACCAATCATGAATGGATGCCGGAAGATCTGGTT<br>AGCCGTATTATTAGCGCACTGACCGATAAACCGAACATTTAA
int2	Gene	ATGCCGATTGCACCGGAATTTCTGAGCCTGGCATATCCGGGTCAAGAATTTCCGGCATATCTGTATGGTCGTGCAAGCCGTGATCCGAAACGTAAAGGT<br>CGTAGCGTTCAGAGCCAGCTGGATGAAGGTCGTGCCACCTGTCTGGATGCAGGTTGGCCGATTGCCGGTGAATTTAAAGATGTTGATCGTAGCGCAAGC<br>GCATATGCACGTCGTACCCGTGATGAATTTGAAGAAATGATTGCAGGTATTCAGGCAGGCGAATGTCGTATTCTGGTTGCATTTGAAGCAAGCCGTTAT<br>TATCGTGATCTGGAAGCATATGTTCGTCTGCGTCGTGTTTGTCGTGAAGCCGGTGTTCTGCTGTGTTATAATGGTCAGGTTTATGATCTGAGCAAAAGC<br>GCAGATCGTAAAGCAACCGCACAGGATGCAGTTAATGCCGAAGGTGAAGCAGATGATATTCGTGAACGTAATCTGCGTACCACCCGTCTGAATGCAAAA<br>CGTGGTGGTGCACATGGTCCGGTTCCGGATGGTTATAAACGTCGTTATGATCCGGATAGCGGTGATCTGGTTGATCAGATTCCGCATCCTGATCGTGCA<br>GGTCTGATTACCGAAATTTTTCGTCGTGCAGCAGCAGCCGAACCGCTGGCAGCAATTTGCCGTGATCTGAATGAACGTGGTGAAACCACCCATCGTGGT<br>AAAGCATGGCAGCGTCATCATCTGCATGCAATTCTGCGTAATCCTGCATATATTGGTCATCGTCGTCATCTGGGTGTTGATACCGGTAAAGGTATGTGG<br>GCACCGATTTGTGATGATGAAGATTTTGCAGAAACCTTTCAGGCCGTTCAAGAAATTCTGAGTCTGCCTGGTCGTCAGCTGAGTCCGGGTCCGGAAGCA<br>CAGCATCTGCAGACCGGTATTGCACTGTGTGGTGAACATCCTGATGAACCGCCTCTGCGTAGCGTTACCGTTCGTGGTCGTACCAATTATAACTGTAGC<br>ACCCGTTATGATGTTGCCATGCGTGAAGATCGTATGGATGCATTTGTTGAAGAAAGCGTGATTACCTGGCTGGCAAGTGATGAAGCAGTTGCAGCCTTT<br>GAAGATAACACCGATGATGAACGTACCCGTAAAGCACGTATTCGTCTGAAAGTTCTGGAAGAACAGCTGGAAGCAGCACAGAAACAGGCACGTACCCTG<br>CGTCCGGATGGCATGGGTATGCTGCTGAGCATTGATAGCCTGGCAGGTCTGGAAGCCGAACTGACACCGCAGATTGATAAAGCCCGTCAAGAAAGCCGT<br>AGCCTGCATGTTCCGGCACTGCTGCGTGATCTGCTGGGTAAACCGCGTGCAGATGTGGATCGTGCATGGAATGAAGCACTGACCCTGCCGCAGCGTCGC<br>ATGATTCTGCGCATGGTTGTTACCATTCGTCTGTTTAAAGCAGGTAGCCGTGGTGTTCGTGCAATTGAACCGGGTCGTATTACCCTGAGCTATGTTGGC<br>GAACCGGGTTTTAAACCGGTTGGTGGTAATCGTGCAAAACAGTAA
int5	Gene	ATGCCTGGTATGACCACCGAAACCGGTCCGGATCCTGCAGGTCTGATTGACCTGTTTTGTCGTAAAAGCAAAGCAGTTAAAAGCCGTGCAAATGGTGCA<br>GGTCAGCGTCGTAAACAAGAAATTAGCATTGCAGCACAAGAAACCCTGGGTCGTAAAGTTGCAGCACTGCTGGGTATGCAGGTTCGTCATGTTTGGAAA<br>GAAGTTGGTAGCGCAAGCCGTTTTCGTAAAGGTAAAGCACGTGATGATCAGAGCAAAGCACTGAAAGCCCTGGAAAGCGGTGAAGTTGGTGCACTGTGG<br>TGTTATCGTCTGGATCGTTGGGATCGTGGTGGTGCCGGTGCAATTCTGAAAATTATCGAACCGGAAGATGGTATGCCTCGTCGTCTGCTGTTTGGTTGG<br>GATGAAGATACCGGTCGTCCGGTTCTGGATAGCACCAATAAACGTGATCGCGGTGAACTGATTCGTCGTGCAGAAGAAGCACGCGAAGAAGCAGAAAAA<br>CTGAGCGAACGTGTTCGTGATACCAAAGCACATCAGCGTGAAAATGGTGAATGGGTTAATGCCCGTGCACCGTATGGTCTGCGTGTTGTTCTGGTTACC<br>GTTAGTGATGAAGAGGGTGATGAATATGATGAACGTAAACTGGCAGCAGATGATGAAGATGCGGGTGGTCCTGATGGTCTGACCAAAGCAGAAGCAGCC<br>CGTCTGGTTTTTACCCTGCCGGTTACCGATCGTCTGAGCTATGCAGGCACCGCACATGCAATGAATACCCGTGAAATTCCGAGCCCGACCGGTGGTCCG<br>TGGATTGCAGTTACCGTGCGTGATATGATTCAGAATCCGGCATATGCGGGTTGGCAGACCACAGGTCGTCAGGATGGTAAACAGCGTCGTCTGACCTTT<br>TATAACGGTGAAGGTAAACGTGTTAGCGTTATGCATGGTCCTCCGCTGGTGACCGATGAAGAACAAGAAGCCGCAAAAGCAGCCGTTAAAGGTGAAGAT<br>GGTGTTGGTGTTCCGCTGGATGGTAGCGATCATGATACCCGTCGCAAACATCTGCTGAGCGGTCGTATGCGTTGTCCGGGTTGTGGTGGTAGCTGTAGC<br>TATAGCGGTAATGGTTATCGTTGTTGGCGTAGCAGTGTGAAAGGTGGTTGTCCGGCACCGACCTATGTTGCACGTAAAAGCGTTGAAGAATATGTTGCA<br>TTTCGTTGGGCAGCAAAATTAGCAGCAAGCGAACCGGATGATCCGTTTGTTATTGCAGTTGCAGATCGCTGGGCAGCACTGACCCATCCGCAGGCAAGC<br>GAAGATGAAAAGTATGCAAAAGCCGCAGTTCGTGAAGCCGAAAAAAATCTGGGTCGCCTGCTGCGTGATCGTCAGAATGGTGTTTATGATGGTCCGGCA<br>GAACAGTTTTTTGCCCCTGCATATCAAGAAGCACTGAGCACCCTGCAGGCAGCCAAAGATGCAGTTAGCGAAAGCAGCGCAAGCGCAGCAGTTGATGTT<br>AGCTGGATTGTTGATAGCAGCGATTATGAAGAACTGTGGCTGCGTGCAACCCCGACCATGCGTAATGCAATTATTGATACCTGCATCGATGAAATTTGG<br>GTTGCAAAAGGCCAGCGTGGTCGTCCGTTTGATGGTGATGAACGCGTTAAAATCAAATGGGCAGCCCGTACCTAA
int7	Gene	ATGAAAGTGGCCATTTATGTTCGTGTTAGCACCGATGAACAGGCCAAAGAAGGTTTTAGCATTCCGGCACAGCGTGAACGTCTGCGTGCATTTTGTGCA<br>AGCCAGGGTTGGGAAATTGTGCAAGAATATATTGAAGAAGGTTGGAGCGCAAAAGATCTGGATCGTCCGCAGATGCAGCGTCTGCTGAAAGATATCAAA<br>AAAGGCAACATTGATATTGTGCTGGTGTATCGTCTGGATCGCCTGACCCGTAGCGTTCTGGATCTGTATCTGCTGCTGCAGACCTTTGAAAAATACAAT<br>GTGGCATTTCGTAGCGCCACCGAAGTTTATGATACCAGCACCGCAATGGGTCGTCTGTTTATTACCCTGGTTGCAGCACTGGCACAGTGGGAACGTGAA<br>AATCTGGCAGAACGTGTTAAATTTGGTATCGAGCAGATGATCGATGAAGGTAAAAAACCGGGTGGTCATAGCCCGTATGGTTACAAATTTGATAAAGAC<br>TTCAATTGCACCATTATTGAGGAAGAAGCAGACGTTGTTCGTATGATCTATCGCATGTATTGTGATGGTTATGGCTATCGTAGCATTGCAGATCGTCTG<br>AATGAACTGATGGTTAAACCGCGTATTGCCAAAGAATGGAATCATAATAGCGTGCGTGATATCCTGACCAACGATATCTATATTGGCACCTATCGTTGG<br>GGTGATAAAGTTGTTCCGAATAATCATCCGCCTATTATTAGCGAAACCCTGTTCAAAAAAGCCCAGAAAGAAAAAGAAAAACGTGGCGTTGATCGTAAA<br>CGCGTTGGTAAATTTCTGTTTACCGGTCTGCTGCAGTGTGGTAATTGTGGTGGCCATAAAATGCAGGGCCATTTTGATAAACGTGAGCAGAAAACCTAT<br>TACCGTTGTACCAAATGTCACCGCATTACCAACGAAAAAACATTCTGGAACCGCTGCTGGATGAAATTCAGCTGCTGATTACCAGCAAAGAATACTTT<br>ATGAGCAAATTCAGCGACCGCTATGATCAGCAAGAGGTTGTTGATGTTAGCGCACTGACAAAAGAACTGGAAAAAATCAAACGCCAGAAAGAGAAATGG<br>TACGATCTGTATATGGATGATCGTAACCCGATTCCGAAAGAAGAACTGTTTGCCAAAATTAACGAACTGAACAAAAAAGAAGAAGAAATCTATAGCAAG<br>CTGAGCGAAGTGGAAGAAGATAAAGAACCGGTTGAAGAGAAATATAACCGCCTGAGCAAAATGATCGATTTTAAACAGCAGTTTGAGCAGGCCAACGAC<br>TTTACCAAAAAAGAGCTGCTGTTCAGCATCTTCGAAAAGATTGTGATTTATCGCGAGAAAGGCAAGCTGAAAAAAATCACCCTGGATTACACCCTGAAA<br>TAA
R6K	Ori	TGATTCGCACGGGCCCATGGCTAATTCCCATGTCAGCCGTTAAGTGTTCCTGTGTCACTCAAAATTGCTTTGAGAGGCTCTAAGGGCTTCTCAGTGCGT<br>TACATCCCTGGCTTGTTGTCCACAACCGTTAAACCTTAAAAGCTTTAAAAGCCTTATATATTCTTTTTTTTCTTATAAAACTTAAAACCTTAGAGGCTA<br>TTTAAGTTGCTGATTTATATTAATTTTATTGTTCAAACATGAGAGCTTAGTACGTGAAACATGAGAGCTTAGTACGTTAGCCATGAGAGCTTAGTACGT<br>TAGCCATGAGGGTTTAGTTCGTTAAACATGAGAGCTTAGTACGTTAAACATGAGAGCTTAGTACGTGAAACATGAGAGCTTAGTACGTACTATCAACAG<br>GTTGAACTGCTGATCTTCAGATCCTCTACGCCGGACGCATCGTGGCCGGATCTT
*R6K**	Ori	CCCATGTCAGCCGTTAAGTGTTCCTGTGTCACTCAAAATTGCTTTGAGAGGCTCTAAGGGCTTCTCAGTGCGTTACATCCCTGGCTTGTTGTCCACAAC<br>CGTTAAACCTTAAAAGCTTTAAAAGCCTTATATATTCTTTTTTTTCTTATAAAACTTAAAACCTTAGAGGCTATTTAAGTTGCTGATTTATATTAATTT<br>TATTGTTCAAACATGAGAGCTTAGTACGTGAAACATGAGAGCTTAGTACGTTAGCCATGAGAGCTTAGTACGTTAGCCATGAGGGTTTAGTTCGTTAAA<br>CATGAGAGCTTAGTACGTTAAACATGAGAGCTTAGTACGTGAAACATGAGAGCTTAGTACGTACTATCAACAGGTTGAACTGCTGATCTTCAGATCCTC<br>TACGCCGGACGCATCGTGGCCGGATCTT
p15a	Ori	ACTAACTCCTCTAAATCAATTACCAGTGGCTGCTGCCAGTGGTGCTTTTGCATGTCTTTCCGGGTTGGACTCAAGACGATAGTTACCGGATAAGGCGCA<br>GCGGTCGGACTGAACGGGGGGTTCGTGCATACAGTCCAGCTTGGAGCGAACTGCCTACCCGGAACTGAGTGTCAGGCGTGGAATGAGACAAACGCGGCC<br>ATAACAGCGGAATGACACCGGTAAACCGAAAGGCAGGAACAGGAGAGCGCACGAGGGAGCCGCCAGGGGGAAACGCCTGGTATCTTTATAGTCCTGTCG<br>GGTTTCGCCACCACTGATTTGAGCGTCAGATTTCGTGATGCTTGTCAGGGGGGCGGAGCCTACGGAAAAACGGCTTTGCCGCGGCCCTCTCACTTCCCT<br>GTTAAGTATCTTCCTGGCATCTTCCAGGAAATCTCCGCCCCGTTCGTAAGCCATTTCCGCTCGCCGCAGTCGAACGACCGAGCGTAGCGAGTCTGTGAG<br>CGAGGAAGCGGAATATATCCTGTATCACATATTCTGCTGACGCACCGGTGCTGCCTTTTTTCTCCTGCCACATGAAGCACTTCACTGACACCCTCATCA<br>GTGCCAACATAGTAAGCCAGTATACACTCCGCTA

oriT	Conjugation element	CTTTTCCGCTGCATAACCCTGCTTCGGGGTCATTATAGCGATTTTTTCGGTATATCCATCCTTTTTCGCACGATATACAGGATTTTGCCAAAGGGTTCGTGTAGACTTTCCTTGGTGTATCCAACGGCGTCAGCCGGGCAGGATAGGTGAAGTAGGCCCACCCGCGAGCGGGTGTTCCTTCTTCACTGTCCCTTATTCGCACCTGGCGGTGCTCAACGGGAATCCTGCTCT

Appendix Table 6: Sequence of plasmids used in this study

Name	Function	DNA Sequence[a]
pIYJP080	Intermediate cloning backbone	TGCCACCTGACGTCTAAGAAACCATTATTATCATGACATTAACCTATAAAAATAGGCGTATCACGAGGCAGAATTTCAGATAAAAAAAT<br>CCTTAGCTTTCGCTAAGGATGATTTCTGGAATTCGCGGCCGCTTCTAGAGGAAGACGCGGAGGGAGACCGGCGCGCCTTTACGGCTAGCT<br>CAGTCCTAGGTACTATGCTAGCAAGGTAGCTGTCACCGGATGTGCTTTCCGGTCTGATGAGTCCGTGAGGACGAAACAGCCTCTACAAAT<br>AATTTTGTTTAATTCACACAGGAAAGTACTAGATGAGCAAAGGAGAAGAACTTTTCACTGGAGTTGTCCCAATTCTTGTTGAATTAGATGG<br>TGATGTTAATGGGCACAAATTTTCTGTCCGTGGAGAGGGTGAAGGTGATGCTACAAACGGAAAACTCACCCTTAAATTTATTTGCACTAC<br>TGGAAAACTACCTGTTCCGTGGCCAACACTTGTCACTACTCTGACCTATGGTGTTCAATGCTTTTCCCGTTATCCGGATCACATGAAACG<br>GCATGACTTTTTCAAGAGTGCCATGCCCGAAGGTTATGTACAGGAACGCACTATATCTTTCAAAGATGACGGGACCTACAAGACGCGTGC<br>TGAAGTCAAGTTTGAAGGTGATACCCTTGTTAATCGTATCGAGTTAAAGGGTATTGATTTTAAAGAAGATGGAAACATTCTTGGACACAA<br>ACTCGAGTACAACTTTAACTCACACAATGTATACATCACGGCAGACAAACAAAAGAATGGAATCAAAGCTAACTTCAAAATTCGCCACAA<br>CGTTGAAGATGGTTCCGTTCAACTAGCAGACCATTATCAACAAAATACTCCAATTGGCGATGGCCCTGTCCTTTTACCAGACAACCATTA<br>CCTGTCGACACAATCTGTCCTTTCGAAAGATCCCAACGAAAAGCGTGACCACATGGTCCTTCTTGAGTTTGTAACTGCTGCTGGGATTAC<br>ACATGGCATGGATGAGCTCTACAAATAATACTAGAGCCAGGCATCAAATAAAACGAAAGGCTCAGTCGAAAGACTGGGCCTTTCGTTTTA<br>TCTGTTGTTTGTCGGTGAACGCTCTCTACTAGAGTCACACTGGCTCACCTTCGGGTGGGCCTTTCTGCGTTTATAGGTCTCATACTATGT<br>CTTCTACTAGTAGCGGCCGCTGCAGTCCGGCAAAAAAGGGCAAGGTGTCACCACCCTGCCCTTTCTTTAAAACCGAAAAGATTACTTCGC<br>GTTATGCAGGCTTCCTCGCTCACTGACTCGCTGCGCTCGGTCGTTCGGCTGCGGCGAGCGGTATCAGCTCACTCAAAGGCGGTAATTTAA<br>TAAGATGATCTTCTTGAGATCGTTTTGGTCTGCGCGTAATCTCTTGCTCTGAAAACGAAAAAACCGCCTTGCAGGGCGGTTTTTCGAAGG<br>TTCTCTGAGCTACCAACTCTTTGAACCGAGGTAACTGGCTTGGAGGAGCGCAGTCACCAAAACTTGTCCTTTCAGTTTAGCCTTAACCGG<br>CGCATGACTTCAAGACTAACTCCTCTAAATCAATTACCAGTGGCTGCTGCCAGTGGTGCTTTTGCATGTCTTTCCGGGTTGGACTCAAGA<br>CGATAGTTACCGGATAAGGCGCAGCGGTCGGACTGAACGGGGGGTTCGTGCATACAGTCCAGCTTGGAGCGAACTGCCTACCCGGAACTG<br>AGTGTCAGGCGTGGAATGAGACAAACGCGGCCATAACAGCGGAATGACACCGGTAAACCGAAAGGCAGGAACAGGAGAGCGCACGAGGGA<br>GCCGCCAGGGGGAAACGCCTGGTATCTTTATAGTCCTGTCGGGTTTCGCCACCACTGATTTGAGCGTCAGATTTCGTGATGCTTGTCAGG<br>GGGGCGGAGCCTACGGAAAAACGGCTTTGCCGCGGCCCTCTCACTTCCCTGTTAAGTATCTTCCTGGCATCTTCCAGGAAATCTCCGCCC<br>CGTTCGTAAGCCATTTCCGCTCGCCGCAGTCGAACGACCGAGCGTAGCGAGTCTGTGAGCGAGGAAGCGGAATATATCCTGTATCACATA<br>TTCTGCTGACGCACCGGTGCTGCCTTTTTTCTCCTGCCACATGAAGCACTTCACTGACACCCTCATCAGTGCCAACATAGTAAGCCAGTA<br>TACACTCCGCTACGATTATCAAAAAGGATCTTCACCTAGATCCTTTTAAATTAAAAATGAAGTTTTAAATCAATCTAAAGTATATATGAG<br>TAAACTTGGTCTGACAGTTACCAATGCTTAATCAGTGAGGCACCTATCTCAGCGATCTGTCTATTTCGTTCATCCATAGTTGCCTGACTC<br>CCCGTCGTGTAGATAACTACGATACGGGAGGGCTTACCATCTGGCCCCAGTGCTGCAATGATACCGCGAGAACCACGCTCACCGGCTCCA<br>GATTTATCAGCAATAAACCAGCCAGCCGGAAGGGCCGAGCGCAGAAGTGGTCCTGCAACTTTATCCGCCTCCATCCAGTCTATTAATTGT<br>TGCCGGGAAGCTAGAGTAAGTAGTTCGCCAGTTAATAGTTTGCGCAACGTTGTTGCCATTGCTACAGGCATCGTGGTGTCACGCTCGTCG<br>TTTGGTATGGCTTCATTCAGCTCCGGTTCCCAACGATCAAGGCGAGTTACATGATCCCCCATGTTGTGCAAAAAAGCGGTTAGCTCCTTC<br>GGTCCTCCGATCGTTGTCAGAAGTAAGTTGGCCGCAGTGTTATCACTCATGGTTATGGCAGCACTGCATAATTCTCTTACTGTCATGCCA<br>TCCGTAAGATGCTTTTCTGTGACTGGTGAGTACTCAACCAAGTCATTCTGAGAATAGTGTATGCGGCGACCGAGTTGCTCTTGCCCGGCG<br>TCAATACGGGATAATACCGCGCCACATAGCAGAACTTTAAAAGTGCTCATCATTGGAAAACGTTCTTCGGGGCGAAAACTCTCAAGGATC<br>TTACCGCTGTTGAGATCCAGTTCGATATAACCCACTCGTGCACCCAACTGATCTTCAGCATCTTTTACTTTCACCAGCGTTTCTGGGTGA<br>GCAAAAACAGGAAGGCAAAATGCCGCAAAAAAGGGAATAAGGGCGACACGGAAATGTTGAATACTCATACTCTTCCTTTTTCAATATTAT<br>TGAAGCATTTATCAGGGTTATTGTCTCATGAGCGGATACATATTTGAATGTATTTAGAAAAATAAACAAATAGGGGTTCCGCGCACATTT<br>CCCCGAAAAG
pYJP053	Integrases	GACGAAAGGGCCTCGTGATACGCCTATTTTTATAGGTTAATGTCATGATAATAATGGTTTCTTAGACGTCAGGTGGCACTTTTCGGGGAA<br>ATGTGCGCGGAACCCCTATTTGTTTATTTTTCTAAATACATTCAAATATGTATCCGCTCATGAGACAATAACCCTGATAAATGCTTCAAT<br>AATATTGAAAAAGGAAGAGTATGAGTATTCAACATTTCCGTGTCGCCCTTATTCCCTTTTTTGCGGCATTTTGCCTTCCTGTTTTTGCTC<br>ACCCAGAAACGCTGGTGAAAGTAAAAGATGCTGAAGATCAGTTGGGTGCACGAGTGGGTTACATCGAACTGGATCTCAACAGCGGTAAGA<br>TCCTTGAGAGTTTTCGCCCCGAAGAACGTTTTCCAATGATGAGCACTTTTAAAGTTCTGCTATGTGGCGCGGTATTATCCCGTATTGACG<br>CCGGGCAAGAGCAACTCGGTCGCCGCATACACTATTCTCAGAATGACTTGGTTGAGTACTCACCAGTCACAGAAAAGCATCTTACGGATG<br>GCATGACAGTAAGAGAATTATGCAGTGCTGCCATAACCATGAGTGATAACACTGCGGCCAACTTACTTCTGACAACGATCGGAGGACCGA<br>AGGAGCTAACCGCTTTTTTGCACAACATGGGGGATCATGTAACTCGCCTTGATCGTTGGGAACCGGAGCTGAATGAAGCCATACCAAACG<br>ACGAGCGTGACACCACGATGCCTGTAGCAATGGCAACAACGTTGCGCAAACTATTAACTGGCGAACTACTTACTCTAGCTTCCCGGCAAC<br>AATTAATAGACTGGATGGAGGCGGATAAAGTTGCAGGACCACTTCTGCGCTCGGCCCTTCCGGCTGGCTGGTTTATTGCTGATAAATCTG<br>GAGCCGGTGAGCGTGGGTCTCGCGGTATCATTGCAGCACTGGGGCCAGATGGTAAGCCCTCCCGTATCGTAGTTATCTACACGACGGGGA<br>GTCAGGCAACTATGGATGAACGAAATAGACAGATCGCTGAGATAGGTGCCTCACTGATTAAGCATTGGTAACTGTCAGACCAAGTTTACT<br>CATATATACTTTAGATTGATTTAAAACTTCATTTTTAATTTAAAAGGATCTAGGTGAAGATCCTTTTTGATAATCTCATGACCAAAATCC<br>CTTAACGTGAGTTTTCGTTCCACTGAGCGTCAGACCCCGTAGAAAAGATCAAAGGATCTTCTTGAGATCCTTTTTTTCTGCGCGTAATCT<br>GCTGCTTGCAAACAAAAAAACCACCGCTACCAGCGGTGGTTTGTTTGCCGGATCAAGAGCTACCAACTCTTTTTCCGAAGGTAACTGGCT<br>TCAGCAGAGCGCAGATACCAAATACTGTCCTTCTAGTGTAGCCGTAGTTAGGCCACCACTTCAAGAACTCTGTAGCACCGCCTACATACC<br>TCGCTCTGCTAATCCTGTTACCAGCCGGTTGTCAGCCGTTAAGTGTTCCTGTGTCACTCAAAATTGCTTTGAGAGGCTCTAAGGGCTTCT<br>CAGTGCGTTACATCCCTGGCTTGTTGTCCACAACCGTTAAACCTTAAAAGCTTTAAAAGCCTTATATATTCTTTTTTTTCTTATAAAACT<br>TAAAACCTTAGAGGCTATTTAAGTTGCTGATTTATATTAATTTTATTGTTCAAACATGAGAGCTTAGTACGTGAAACATGAGAGCTTAGT<br>ACGTTAGCCATGAGAGCTTAGTACGTTAGCCATGAGGGTTTAGTTCGTTAAACATGAGAGCTTAGTACGTTAAACTTGAGAGCTTAGTAC<br>GTGAAACATGAGAGCTTAGTACGTACTATCAACAGGTTGAACTGCCCATGTTCTTTCCTGCGTTATCAGAGCTTATCGGCCAGCCTCGCA<br>GAGCAGGATTCCCGTTGAGCACCGCCAGGTGCGAATAAGGGACAGTGAAGAAGGAACACCCGCTCGCGGGTGGGCCTACTTCACCTATCC<br>TGCCCGGCTGACGCCGTTGGATACACCAAGGAAAGTCTACACGAACCCTTTGGCAAAATCCTGTATATCGTGCGAAAAAGGATGGATATA<br>CCGAAAAAATCGCTATAATGACCCCGAAGCAGGGTTATGCAGCGGAAAGTATACCTTAACATGTTCTTTCCTGCGTTATCCCCTGATTCT<br>GTGGATAACCGTATTACCGCCTTTGAGTGAGCTGATACCGCTCGCCGCAGCCGAACGACCGAGCGCAGCGAGTCAGTGAGCGAGGAAGCG<br>GAAGAGCGCCCAATACGCAAACCGCCTCTCCCCGCGCGTTGGCCGATTCATTAATGCAGCTGGCACGACAGGTTTCCCGACTGGAAAGCG<br>GGCAGTGAGCGCAACGCAATTAATGTGAGTTAGCTCACTCATTAGGCACCCCAGGCTTTACACTTTATGCTTCCGGCTCGTATGTTGTGT<br>GGAATTGTGAGCGGATAACAATTTCACACAGGAAACAGCTATGACCATGATTACGAATTCGGCTTGCGGCCGCTTCTAGAGGGCGCGCCT<br>TTACAGCTAGCTCAGTCCTAGGTATTATGCTAGCCAGTTAGCTGTCACCGGATGTGCTTTCCGGTCTGATGAGTCCGTGAGGACGAAACA<br>GCCTCTACAAATAATTTTGTTTAATCACACAGGAAAGTACTAGATGCCGATTGCACCGGAATTTCTGAGCCTGGCATATCCGGGTCAAGA<br>ATTTCCGGCATATCTGTATGGTCGTGCAAGCCGTGATCCGAAACGTAAAGGTCGTAGCGTTCAGAGCCAGCTGGATGAAGGTCGTGCCAC<br>CTGTCTGGATGCAGGTTGGCCGATTGCCGGTGAATTTAAAGATGTTGATCGTAGCGCAAGCGCATATGCACGTCGTACCCGTGATGAATT<br>TGAAGAAATGATTGCAGGTATTCAGGCAGGCGAATGTCGTATTCTGGTTGCATTTGAAGCAAGCCGTTATTATCGTGATCTGGAAGCATA<br>TGTTCGTCTGCGTCGTGTTTGTCGTGAAGCCGGTGTTCTGCTGTGTTATAATGGTCAGGTTTATGATCTGAGCAAAAGCGCAGATCGTAA<br>AGCAACCGCACAGGATGCAGTTAATGCCGAAGGTGAAGCAGATGATATTCGTGAACGTAATCTGCGTACCACCCGTCTGAATGCAAAACG<br>TGGTGGTGCACATGGTCCGGTTCCGGATGGTTATAAACGTCGTTATGATCCGGATAGCGGTGATCTGGTTGATCAGATTCCGCATCCTGA<br>TCGTGCAGGTCTGATTACCGAAATTTTTCGTCGTGCAGCAGCAGCCGAACCGCTGGCAGCAATTTGCCGTGATCTGAATGAACGTGGTGA<br>AACCACCCATCGTGGTAAAGCATGGCAGCGTCATCATCTGCATGCAATTCTGCGTAATCCTGCATATATTGGTCATCGTCGTCATCTGGG<br>TGTTGATACCGGTAAAGGTATGTGGGCACCGATTTGTGATGATGAAGATTTTGCAGAAACCTTTCAGGCCGTTCAAGAAATTCTGAGTCT<br>GCCTGGTCGTCAGCTGAGTCCGGGTCCGGAAGCACAGCATCTGCAGACCGGTATTGCACTGTGTGGTGAACATCCTGATGAACCGCCTCT<br>GCGTAGCGTTACCGTTCGTGGTCGTACCAATTATAACTGTAGCACCCGTTATGATGTTGCCATGCGTGAAGATCGTATGGATGCATTTGT<br>TGAAGAAAGCGTGATTACCTGGCTGGCAAGTGATGAAGCAGTTGCAGCCTTTGAAGATAACACCGATGATGAACGTACCCGTAAAGCACG<br>TATTCGTCTGAAAGTTCTGGAAGAACAGCTGGAAGCAGCACAGAAACAGGCACGTACCCTGCGTCCGGATGGCATGGGTATGCTGCTGAG<br>CATTGATAGCCTGGCAGGTCTGGAAGCCGAACTGACACCGCAGATTGATAAAGCCCGTCAAGAAAGCCGTAGCCTGCATGTTCCGGCACT<br>GCTGCGTGATCTGCTGGGTAAACCGCGTGCAGATGTGGATCGTGCATGGAATGAAGCACTGACCCTGCCGCAGCGTCGCATGATTCTGCG<br>CATGGTTGTTACCATTCGTCTGTTTAAAGCAGGTAGCCGTGGTGTTCGTGCAATTGAACCGGGTCGTATTACCCTGAGCTATGTTGGCGA<br>ACCGGGTTTTAAACCGGTTGGTGGTAATCGTGCAAAACAGTAATCACACAGGAAAGTACTAGATGCCTGGTATGACCACCGAAACCGGTC<br>CGGATCCTGCAGGTCTGATTGACCTGTTTTGTCGTAAAAGCAAAGCAGTTAAAAGCCGTGCAAATGGTGCAGGTCAGCGTCGTAAACAAG<br>AAATTAGCATTGCAGCACAAGAAACCCTGGGTCGTAAAGTTGCAGCACTGCTGGGTATGCAGGTTCGTCATGTTTGGAAAGAAGTTGGTA<br>GCGCAAGCCGTTTTCGTAAAGGTAAAGCACGTGATGATCAGAGCAAAGCACTGAAAGCCCTGGAAAGCGGTGAAGTTGGTGCACTGTGGT<br>GTTATCGTCTGGATCGTTGGGATCGTGGTGGTGCCGGTGCAATTCTGAAAATTATCGAACCGGAAGATGGTATGCCTCGTCGTCTGCTGT<br>TTGGTTGGGATGAAGATACCGGTCGTCCGGTTCTGGATAGCACCAATAAACGTGATCGCGGTGAACTGATTCGTCGTGCAGAAGAAGCAC<br>GCGAAGAAGCAGAAAAACTGAGCGAACGTGTTCGTGATACCAAAGCACATCAGCGTGAAAATGGTGAATGGGTTAATGCCCGTGCACCGT<br>ATGGTCTGCGTGTTGTTCTGGTTACCGTTAGTGATGAAGAGGGTGATGAATATGATGAACGTAAACTGGCAGCAGATGATGAAGATGCGG

		GTGGTCCTGATGGTCTGACCAAAGCAGAAGCAGCCCGTCTGGTTTTTACCCTGCCGGTTACCGATCGTCTGAGCTATGCAGGCACCGCAC<br>ATGCAATGAATACCCGTGAAATTCCGAGCCCGACCGGTGGTCCGTGGATTGCAGTTACCGTGCGTGATATGATTCAGAATCCGGCATATG<br>CGGGTTGGCAGACCACAGGTCGTCAGGATGGTAAACAGCGTCGTCTGACCTTTTATAACGGTGAAGGTAAACGTGTTAGCGTTATGCATG<br>GTCCTCCGCTGGTGACCGATGAAGAACAAGAAGCCGCAAAAGCAGCCGTTAAAGGTGAAGATGGTGTTGGTGTTCCGCTGGATGGTAGCG<br>ATCATGATACCCGTCGCAAACATCTGCTGAGCGGTCGTATGCGTTGTCCGGGTTGTGGTGGTAGCTGTAGCTATAGCGGTAATGGTTATC<br>GTTGTTGGCGTAGCAGTGTGAAAGGTGGTTGTCCGGCACCGACCTATGTTGCACGTAAAAGCGTTGAAGAATATGTTGCATTTCGTTGGG<br>CAGCAAAATTAGCAGCAAGCGAACCGGATGATCCGTTTGTTATTGCAGTTGCAGATCGCTGGGCAGCACTGACCCATCCGCAGGCAAGCG<br>AAGATGAAAAGTATGCAAAAGCCGCAGTTCGTGAAGCCGAAAAAAATCTGGGTCGCCTGCTGCGTGATCGTCAGAATGGTGTTTATGATG<br>GTCCGGCAGAACAGTTTTTTGCCCCTGCATATCAAGAAGCACTGAGCACCCTGCAGGCAGCCAAAGATGCAGTTAGCGAAAGCAGCGCAA<br>GCGCAGCAGTTGATGTTAGCTGGATTGTTGATAGCAGCGATTATGAAGAACTGTGGCTGCGTGCAACCCCGACCATGCGTAATGCAATTA<br>TTGATACCTGCATCGATGAAATTTGGGTTGCAAAAGGCCAGCGTGGTCGTCCGTTTGATGGTGATGAACGCGTTAAAATCAAATGGGCAG<br>CCCGTACCTAATCACACAGGAAAGTACTAGATGAAAGTGGCCATTTATGTTCGTGTTAGCACCGATGAACAGGCCAAAGAAGGTTTTAGC<br>ATTCCGGCACAGCGTGAACGTCTGCGTGCATTTTGTGCAAGCCAGGGTTGGGAAATTGTGCAAGAATATATTGAAGAAGGTTGGAGCGCA<br>AAAGATCTGGATCGTCCGCAGATGCAGCGTCTGCTGAAAGATATCAAAAAAGGCAACATTGATATTGTGCTGGTGTATCGTCTGGATCGC<br>CTGACCCGTAGCGTTCTGGATCTGTATCTGCTGCTGCAGACCTTTGAAAAATACAATGTGGCATTTCGTAGCGCCACCGAAGTTTATGAT<br>ACCAGCACCGCAATGGGTCGTCTGTTTATTACCCTGGTTGCAGCACTGGCACAGTGGGAACGTGAAAATCTGGCAGAACGTGTTAAATTT<br>GGTATCGAGCAGATGATCGATGAAGGTAAAAAACCGGGTGGTCATAGCCCGTATGGTTACAAATTTGATAAAGACTTCAATTGCACCATT<br>ATTGAGGAAGAAGCAGACGTTGTTCGTATGATCTATCGCATGTATTGTGATGGTTATGGCTATCGTAGCATTGCAGATCGTCTGAATGAA<br>CTGATGGTTAAACCGCGTATTGCCAAAGAATGGAATCATAATAGCGTGCGTGATATCCTGACCAACGATATCTATATTGGCACCTATCGT<br>TGGGGTGATAAAGTTGTTCCGAATAATCATCCGCCTATTATTAGCGAAACCCTGTTCAAAAAAGCCCAGAAAGAAAAAGAAAAACGTGGC<br>GTTGATCGTAAACGCGTTGGTAAATTTCTGTTTACCGGTCTGCTGCAGTGTGGTAATTGTGGTGGCCATAAAATGCAGGGCCATTTTGAT<br>AAACGTGAGCAGAAAACCTATTACCGTTGTACCAAATGTCACCGCATTACCAACGAAAAAAACATTCTGGAACCGCTGCTGGATGAAATT<br>CAGCTGCTGATTACCAGCAAAGAATACTTTATGAGCAAATTCAGCGACCGCTATGATCAGCAAGAGGTTGTTGATGTTAGCGCACTGACA<br>AAAGAACTGGAAAAAATCAAACGCCAGAAAGAGAAATGGTACGATCTGTATATGGATGATCGTAACCCGATTCCGAAAGAAGAACTGTTT<br>GCCAAAATTAACGAACTGAACAAAAAAGAAGAAGAAATCTATAGCAAGCTGAGCGAAGTGGAAGAAGATAAAGAACCGGTTGAAGAGAAA<br>TATAACCGCCTGAGCAAAATGATCGATTTTAAACAGCAGTTTGAGCAGGCCAACGACTTTACCAAAAAAGAGCTGCTGTTCAGCATCTTC<br>GAAAAGATTGTGATTTATCGCGAGAAAGGCAAGCTGAAAAAAATCACCCTGGATTACACCCTGAAATAATCCGGCAATTAAAAAAGCGGC<br>TAACCACGCCGCTTTTTTTACGTCTGCACTCGGTACCAAATTCCAGAAAAGAGGCCTCCCGAAAGGGGGGCCTTTTTTCGTTTTGGTCCG<br>TGGTTAGGCTAGCTACATTTAATGACAATGTGCAGGTTGTACATATTGGTCATTTATTCCGTAACTCGGGTCATAAGGAGTGGCGTATTT<br>TTGTTTGGTTTAATCCAATGCAAGAACGGAAATGGACTCGATTTACTCATTTGCCTTTATTAAGTCGAGCTAAGGTGGTTAACAGTACAA<br>CAAAGCAAATAAATAAGGCGGATCGTGTGATTGAGTTTGAAGCATCGGATCTTCAACGAGCCAAAATAATCGGGGGATCCTCTAGAGTCG<br>ACCTGCAGGCATGCAAGCTTGGCACTGGCCGTCGTTTTACAACGTCGTGACTGGGAAAACCCTGGCGTTACCCAACTTAATCGCCTTGCA<br>GCACATCCCCCTTTCGCCAGCTGGCGTAATAGCGAAGAGGCCCGCACCGATCGCCCTTCCCAACAGTTGCGCAGCCTGAATGGCGAATGG<br>CGCCTGATGCGGTATTTTCTCCTTACGCATCTGTGCGGTATTTCACACCGCATATGGTGCACTCTCAGTACAATCTGCTCTGATGCCGCA<br>TAGTTAAGCCAGCCCCGACACCCGCCAACACCCGCTGACGCGCCCTGACGGGCTTGTCTGCTCCCGGCATCCGCTTACAGACAAGCTGTG<br>ACCGTCTCCGGGAGCTGCATGTGTCAGAGGTTTTCACCGTCATCACCGAAACGCGCGA
pGR-DT5	Terminator strength assay	TACTTTTCATACTCCCGCCATTCAGAGAAGAAACCAATTGTCCATATTGCATCAGACATTGCCGTCACTGCGTCTTTTACTGGCTCTTCT<br>CGCTAACCAAACCGGTAACCCCGCTTATTAAAAGCATTCTGTAACAAAGCGGGACCAAAGCCATGACAAAAACGCGTAACAAAAGTGTCT<br>ATAATCACGGCAGAAAAGTCCACATTGATTATTTGCACGGCGTCACACTTTGCTATGCCATAGCATTTTTATCCATAAGATTAGCGGATC<br>CTACCTGACGCTTTTTATCGCAACTCTCTACTGTTTCTCCATACCCGTTTTTTTGGGCTAGCTACTAGAGAAAGAGGAGAAATACTAGAT<br>GCGTAAAGGAGAAGAACTTTTCACTGGAGTTGTCCCAATTCTTGTTGAATTAGATGGTGATGTTAATGGGCACAAATTTTCTGTCAGTGG<br>AGAGGGTGAAGGTGATGCAACATACGGAAAACTTACCCTTAAATTTATTTGCACTACTGGAAAACTACCTGTTCCATGGCCAACACTTGT<br>CACTACTTTCGGTTATGGTGTTCAATGCTTTGCGAGATACCCAGATCATATGAAACAGCATGACTTTTTCAAGAGTGCCATGCCCGAAGG<br>TTATGTACAGGAAAGAACTATATTTTTCAAAGATGACGGGAACTACAAGACACGTGCTGAAGTCAAGTTTGAAGGTGATACCCTTGTTAA<br>TAGAATCGAGTTAAAAGGTATTGATTTTAAAGAAGATGGAAACATTCTTGGACACAAATTGGAATACAACTATAACTCACACAATGTATA<br>CATCATGGCAGACAAACAAAAGAATGGAATCAAAGTTAACTTCAAAATTAGACACAACATTGAAGATGGAAGCGTTCAACTAGCAGACCA<br>TTATCAACAAAATACTCCAATTGGCGATGGCCCTGTCCTTTTACCAGACAACCATTACCTGTCCACACAATCTGCCCTTTCGAAAGATCC<br>CAACGAAAAGAGAGACCACATGGTCCTTCTTGAGTTTGTAACAGCTGCTGGGATTACACATGGCATGGATGAACTATACAAATAATAAGA<br>ATTCTCCGGCAATTAAAAAAGCGGCTAACCACGCCGCTTTTTTTACGTCTGCACTCGGTACCAAATTCCAGAAAAGAGGCCTCCCGAAAG<br>GGGGGCCTTTTTTCGTTTTGGTCCACTAGTAGCGGCCGCTGCAGAAAGAGGAGAAATACTAGATGGCTTCCTCCGAAGACGTTATCAAAG<br>AGTTCATGCGTTTCAAAGTTCGTATGGAAGGTTCCGTTAACGGTCACGAGTTCGAAATCGAAGGTGAAGGTGAAGGTCGTCCGTACGAAG<br>GTACCCAGACCGCTAAACTGAAAGTTACCAAAGGTGGTCCGCTGCCGTTCGCTTGGGACATCCTGTCCCCGCAGTTCCAGTACGGTTCCA<br>AAGCTTACGTTAAACACCCGGCTGACATCCCGGACTACCTGAAACTGTCCTTCCCGGAAGGTTTCAAATGGGAACGTGTTATGAACTTCG<br>AAGACGGTGGTGTTGTTACCGTTACCCAGGACTCCTCCCTGCAAGACGGTGAGTTCATCTACAAAGTTAAACTGCGTGGTACCAACTTCC<br>CGTCCGACGGTCCGGTTATGCAGAAAAAAACCATGGGTTGGGAAGCTTCCACCGAACGTATGTACCCGGAAGACGGTGCTCTGAAAGGTG<br>AAATCAAAATGCGTCTGAAACTGAAAGACGGTGGTCACTACGACGCTGAAGTTAAAACCACCTACATGGCTAAAAAACCGGTTCAGCTGC<br>CGGGTGCTTACAAAACCGACATCAAACTGGACATCACCTCCCACAACGAAGACTACACCATCGTTGAACAGTACGAACGTGCTGAAGGTC<br>GTCACTCCACCGGTGCTTAATAACGCTGATAGTGCTAGTGTAGATCGCTACTAGAGCCAGGCATCAAATAAAACGAAAGGCTCAGTCGAA<br>AGACTGGGCCTTTCGTTTTATCTGTTGTTTGTCGGTGAACGCTCTCTACTAGAGTCACACTGGCTCACCTTCGGGTGGGCCTTTCTGCGT<br>TTATATACTAGAGTCAATTGAGGTAGAGGTACACACGCGAACTCCGATAGCCAATTCAGAGTAATAAACTGTGATAATCAATGCAGTGCT<br>TCCTCGCTCACTGACTCGCTGCGCTCGGTCGTTCGGCTGCGGCGAGCGGTATCAGCTCACTCAAAGGCGGTAATACGGTTATCCACAGAA<br>TCAGGGGATAACGCAGGAAAGAACATGTGAGCAAAAGGCCAGCAAAAGGCCAGGAACCGTAAAAAGGCCGCGTTGCTGGCGTTTTTCCAT<br>AGGCTCCGCCCCCCTGACGAGCATCACAAAAATCGACGCTCAAGTCAGAGGTGGCGAAACCCGACAGGACTATAAAGATACCAGGCGTTT<br>CCCCCTGGAAGCTCCCTCGTGCGCTCTCCTGTTCCGACCCTGCCGCTTACCGGATACCTGTCCGCCTTTCTCCCTTCGGGAAGCGTGGCG<br>CTTTCTCATAGCTCACGCTGTAGGTATCTCAGTTCGGTGTAGGTCGTTCGCTCCAAGCTGGGCTGTGTGCACGAACCCCCCGTTCAGCCC<br>GACCGCTGCGCCTTATCCGGTAACTATCGTCTTGAGTCCAACCCGGTAAGACACGACTTATCGCCACTGGCAGCAGCCACTGGTAACAGG<br>ATTAGCAGAGCGAGGTATGTAGGCGGTGCTACAGAGTTCTTGAAGTGGTGGCCTAACTACGGCTACACTAGAAGGACAGTATTTGGTATC<br>TGCGCTCTGCTGAAGCCAGTTACCTTCGGAAAAAGAGTTGGTAGCTCTTGATCCGGCAAACAAACCACCGCTGGTAGCGGTGGTTTTTTT<br>GTTTGCAAGCAGCAGATTACGCGCAGAAAAAAAGGATCTCAAGAAGATCCTTTGATCTTTTCTACGGGGTCTGACGCTCAGTGGAACGAA<br>AACTCACGTTAAGGGATTTTGGTCATGAGATTATCAAAAAGGATCTTCACCTAGATCCTTTTAAATTAAAAATGAAGTTTTAAATCAATC<br>TAAAGTATATATGAGTAAACTTGGTCTGACAGTTACCAATGCTTAATCAGTGAGGCACCTATCTCAGCGATCTGTCTATTTCGTTCATCC<br>ATAGTTGCCTGACTCCCCGTCGTGTAGATAACTACGATACGGGAGGGCTTACCATCTGGCCCCAGTGCTGCAATGATACCGCGAGACCCA<br>CGCTCACCGGCTCCAGATTTATCAGCAATAAACCAGCCAGCCGGAAGGGCCGAGCGCAGAAGTGGTCCTGCAACTTTATCCGCCTCCATC<br>CAGTCTATTAATTGTTGCCGGGAAGCTAGAGTAAGTAGTTCGCCAGTTAATAGTTTGCGCAACGTTGTTGCCATTGCTACAGGCATCGTG<br>GTGTCACGCTCGTCGTTTGGTATGGCTTCATTCAGCTCCGGTTCCCAACGATCAAGGCGAGTTACATGATCCCCCATGTTGTGCAAAAAA<br>GCGGTTAGCTCCTTCGGTCCTCCGATCGTTGTCAGAAGTAAGTTGGCCGCAGTGTTATCACTCATGGTTATGGCAGCACTGCATAATTCT<br>CTTACTGTCATGCCATCCGTAAGATGCTTTTCTGTGACTGGTGAGTACTCAACCAAGTCATTCTGAGAATAGTGTATGCGGCGACCGAGT<br>TGCTCTTGCCCGGCGTCAATACGGGATAATACCGCGCCACATAGCAGAACTTTAAAAGTGCTCATCATTGGAAAACGTTCTTCGGGGCGA<br>AAACTCTCAAGGATCTTACCGCTGTTGAGATCCAGTTCGATGTAACCCACTCGTGCACCCAACTGATCTTCAGCATCTTTTACTTTCACC<br>AGCGTTTCTGGGTGAGCAAAAACAGGAAGGCAAAATGCCGCAAAAAAGGGAATAAGGGCGACACGGAAATGTTGAATACTCATACTCTTC<br>CTTTTTCAATATTATTGAAGCATTTATCAGGGTTATTGTCTCATGAGCGGATACATATTTGAATGTATTTAGAAAAATAAACAAATAGGG<br>GTTCCGCGCACATTTCCCCGAAAAGTGCCACCTGACGTCTAAGAAACCATTATTATCATGACATTAACCTATAAAAATAGGCGTATCACG<br>AGGCAGAATTTCAGATAAAAAAAATCCTTAGCTTTCGCTAAGGATGATTTCTGGAATTGAATTGAGGTAGAGGTACACACGCGAACTCCG<br>ATAGCCAATTCAGAGTAATAAACTGTGATAATCAATGCATGTACTAGAGTTATGACAACTTGACGGCTACATCATTCACTTTTTCTTCAC<br>AACCGGCACGGAACTCGCTCGGGCTGGCCCCGGTGCATTTTTTAAATACCCGCGAGAAATAGAGTTGATCGTCAAAACCAACATTGCGAC<br>CGACGGTGGCGATAGGCATCCGGGTGGTGCTCAAAAGCAGCTTCGCCTGGCTGATACGTTGGTCCTCGCGCCAGCTTAAGACGCTAATCC<br>CTAACTGCTGGCGGAAAAGATGTGACAGACGCGACGGCGACAAGCAAACATGCTGTGCGACGCTGGCGATATCAAAATTGCTGTCTGCCA<br>GGTGATCGCTGATGTACTGACAAGCCTCGCGTACCCGATTATCCATCGGTGGATGGAGCGACTCGTTAATCGCTTCCATGCGCCGCAGTA<br>ACAATTGCTCAAGCAGATTTATCGCCAGCAGCTCCGAATAGCGCCCTTCCCCTTGCCCGGCGTTAATGATTTGCCCAAACAGGTCGCTGA<br>AATGCGGCTGGTGCGCTTCATCCGGGCGAAAGAACCCCGTATTGGCAAATATTGACGGCCAGTTAAGCCATTCATGCCAGTAGGCGCGCG<br>GACGAAAGTAAACCCACTGGTGATACCATTCGCGAGCCTCCGGATGACGACCGTAGTGATGAATCTCTCCTGGCGGGAACAGCAAAATAT<br>CACCCGGTCGGCAAACAAATTCTCGTCCCTGATTTTTCACCACCCCCTGACCGCGAATGGTGAGATTGAGAATATAACCTTTCATTCCCA<br>GCGGTCGGTCGATAAAAAAATCGAGATAACCGTTGGCCTCAATCGGCGTTAAACCCGCCACCAGATGGGCATTAAACGAGTATCCCGGCA<br>GCAGGGGATCATTTTGCGCTTCAGCCA

pYJP017	Transposon Insertion of *mCherry* probe	GCGAGGCTGGCCGTAGGCCGGCCGATCTGAAGATCAGCAGTTCAACCTGTTGATAGTACGTACTAAGCTCTCATGTTTCACGTACTAAGC<br>TCTCATGTTTAACGTACTAAGCTCTCATGTTTAACGAACTAAACCCTCATGGCTAACGTACTAAGCTCTCATGGCTAACGTACTAAGCTC<br>TCATGTTTCACGTACTAAGCTCTCATGTTTGAACAATAAAATTAATATAAATCAGCAACTTAAATAGCCTCTAAGGTTTTAAGTTTTATA<br>AGAAAAAAAAGAATATATAAGGCTTTTAAAGCCTTTAAGGTTTAACGGTTGTGGACAACAAGCCAGGGATGTAACGCACTGAGAAGCCCT<br>TAGAGCCTCTCAAAGCAATTTTGAGTGACACAGGAACACTTAACGGCTGACATGGGGCGCGCCCAGCTGTCTAGGGCGGCGGATTTGTCC<br>TACTCAGCAGATAAAAAAAATCCTTAGCTTTCGCTAAGGATGATTTCTGGAATTCGCGGCCGCTTCTAGAGGGCGCGCCTTTACAGCTAG<br>CTCAGTCCTAGGTATTATGCTAGCCAGTTAGCTGTCACCGGATGTGCTTTCCGGTCTGATGAGTCCGTGAGGACGAAACAGCCTCTACAA<br>ATAATTTTGTTTAATCACACAGGAAAGTACTAGATGAGCAAAGGAGAAGAACTTTTCACTGGAGTTGTCCCAATTCTTGTTGAATTAGAT<br>GGTGATGTTAATGGGCACAAATTTTCTGTCCGTGGAGAGGGTGAAGGTGATGCTACAAACGGAAAACTCACCCTTAAATTTATTTGCACT<br>ACTGGAAAACTACCTGTTCCGTGGCCAACACTTGTCACTACTCTGACCTATGGTGTTCAATGCTTTTCCCGTTATCCGGATCACATGAAA<br>CGGCATGACTTTTTCAAGAGTGCCATGCCCGAAGGTTATGTACAGGAACGCACTATATCTTTCAAAGATGACGGGACCTACAAGACGCGT<br>GCTGAAGTCAAGTTTGAAGGTGATACCCTTGTTAATCGTATCGAGTTAAAGGGTATTGATTTTAAAGAAGATGGAAACATTCTTGGACAC<br>AAACTCGAGTACAACTTTAACTCACACAATGTATACATCACGGCAGACAAACAAAAGAATGGAATCAAAGCTAACTTCAAAATTCGCCAC<br>AACGTTGAAGATGGTTCCGTTCAACTAGCAGACCATTATCAACAAAATACTCCAATTGGCGATGGCCCTGTCCTTTTACCAGACAACCAT<br>TACCTGTCGACACAATCTGTCCTTTCGAAAGATCCCAACGAAAAGCGTGACCACATGGTCCTTCTTGAGTTTGTAACTGCTGCTGGGATT<br>ACACATGGCATGGATGAGCTCTACAAATAATTCAGCCAAAAAACTTAAGACCGCCGGTCTTGTCCACTACCTTGCAGTAATGCGGTGGAC<br>AGGATCGGCGGTTTTCTTTTCTCTTCTCAATACAAATGAAAGTACATAGAAATTACTCGGTACCAAATTCCAGAAAAGAGGCCTCCCGAA<br>AGGGGGGCCTTTTTTCGTTTTGGTCCTCATAGGCAATACGATCGCATGTCCGAGAGCGTTCACCGACAAACAACAGATAAAACGAAAGGC<br>CCAGTCTTTCGACTGAGCCTTTCGTTTTATTTGATGCCTTTAATTAACTGTCTCTTATACACATCTTTGTGTCTCAGGCCGCCTAGGCCG<br>CGGCCGCGCGAATTCGAGCTCGGTACCCGGGGATCCTCCGGCAATTAAAAAAGCGGCTAACCACGCCGCTTTTTTTACGTCTGCATCCTT<br>GGCCCTCCATCCTTAGATAGCTCGGTACCAAATTCCAGAAAAGAGGCCTCCCGAAAGGGGGGCCTTTTTTCGTTTTGGTCCTCATAGGCA<br>ATACGATCGCATGTCCTTGACAATTAATCATCCGGCTCGTAACTTATGTGGACTCGGAGCGCTCAACGGGTGTGCTTCCCGTTCTGATGA<br>GTCCGTGAGGACGAAAGCGCCTCTACAAATAATTTTGTTTAAAAATTTAATGATGAGTACAAAGAAGTACTAAGGAAGGAAAATACCCAA<br>TGGTTTCTAAGGGCGAAGAAGATAATATGGCCATTATCAAGGAGTTTATGCGCTTTAAAGTGCACATGGAGGGCTCAGTAAACGGTCACG<br>AATTTGAAATCGAAGGAGAAGGCGAGGGCCGTCCGTACGAGGGGACTCAGACGGCGAAGCTCAAAGTCACAAAAGGTGGCCCATTGCCAT<br>TCGCCTGGGATATTCTGTCGCCGCAGTTCATGTATGGCAGCAAAGCGTACGTAAAACACCCGGCAGATATTCCGGATTATCTGAAATTAT<br>CTTTCCCGGAAGGTTTCAAATGGGAACGCGTTATGAATTTCGAGGACGGGGGCGTAGTGACGGTCACGCAGGATAGTAGCCTGCAGGACG<br>GCGAATTCATTTACAAAGTCAAGCTGCGTGGCACAAATCTTCCGTCTGATGGCCCGGTGATGCAGAAGAAAACCATGGGTTGGGAGGCTT<br>CCAGCGAACGCATGTATCCCGAAGATGGAGCATTGAAAGGCGAAATCAAACAGCGCCTGAAATTAAAAGACGGCGGACATTACGACGCCG<br>AGGTCAAGACCACTTACAAAGCGAAAAAACCAGTTCAGCTGCCAGGCGCCTACAACGTTAATATCAAGCTGGATATTACCTCGCATAATG<br>AAGATTATACTATCGTGGAACAATACGAGCGCGCCGAAGGCCGCCACTCCACTGGTGGTATGGATGAATTATATAAATAGGAAAACCTGT<br>ACTTCCAGGGCCCGGCGAGCGTGCCGCAGGGCATTGATTATAAAGATGATGATGATAAGTAATAAACATTTAATAAAAAAAGGGCGGTCG<br>CAAGATCGCCCTTTTTTACGTATGACACCTCGTCTCAACCAAAGCAATCAACCAGTGAAAAATGGCGCCCATCGGCGCCATTTTTTTATG<br>GTTCCTTATCATCTGGCGAATCGGAAAGCTTGCGGCCGCCAAAGCCCGCCGAAAGGCGGGCTTTTCTGTATTTAAATGAACCTTGACCGA<br>ACGCAGCGGTGGTAACGGCGCAGTGGCGGTTTTCATGGCTTGTTATGACTGTTTTTTTGGGGTACAGTCTATGCCTCGGGCATCCAAGCA<br>GCAAGCGCGTTACGCCGTGGGTCGATGTTTGATGTTATGGAGCAGCAACGATGTTACGCAGCAGGGCAGTCGCCCTAAAACAAAGTTAAA<br>CATCATGAGGGAAGCGGTGATCGCCGAAGTATCGACTCAACTATCAGAGGTAGTTGGCGTCATCGAGCGCCATCTCGAACCGACGTTGCT<br>GGCCGTACATTTGTACGGCTCCGCAGTGGATGGCGGCCTGAAGCCACACAGTGATATTGATTTGCTGGTTACGGTGACCGTAAGGCTTGA<br>TGAAACAACGCGGCGAGCTTTGATCAACGACCTTTTGGAAACTTCGGCTTCCCCTGGAGAGAGCGAGATTCTCCGCGCTGTAGAAGTCAC<br>CATTGTTGTGCACGACGACATCATTCCGTGGCGTTATCCAGCTAAGCGCGAACTGCAATTTGGAGAATGGCAGCGCAATGACATTCTTGC<br>AGGTATCTTCGAGCCAGCCACGATCGACATTGATCTGGCTATCTTGCTGACAAAAGCAAGAGAACATAGCGTTGCCTTGGTAGGTCCAGC<br>GGCGGAGGAACTCTTTGATCCGGTTCCTGAACAGGATCTATTTGAGGCGCTAAATGAAACCTTAACGCTATGGAACTCGCCGCCCGACTG<br>GGCTGGCGATGAGCGAAATGTAGTGCTTACGTTGTCCCGCATTTGGTACAGCGCAGTAACCGGCAAAATCGCGCCGAAGGATGTCGCTGC<br>CGACTGGGCAATGGAGCGCCTGCCGGCCCAGTATCAGCCCGTCATACTTGAAGCTAGACAGGCTTATCTTGGACAAGAAGAAGATCGCTT<br>GGCCTCGCGCGCAGATCAGTTGGAAGAATTTGTCCACTACGTGAAAGGCGAGATCACCAAGGTAGTCGGCAAATAAGACAATTGTCTAAT<br>TAATTGCGGACCCTAGAGGTCCCCTTTTTTATTTTAAAAATTTTTTCACAAAACGGTTTACAAGCATAAAATCTCTGAAGATGTGTATAA<br>GAGACAGACTAGTCTTGGACTCCTGTTGATAGATCCAGTAATGACCTCAGAACTCCATCTGGATTTGTTCAGAACGCTCGGTTGCCGCCG<br>GGCGTTTTTTATTGGTGAGAATCCAGGGGTCCCCTGGTTTAAACTACACAAGTAGCGTCCTGAACGGAACCTTTCCCGTTTTCCAGAATC<br>TGATGTTCCATGTGACCTCCTAACATGGTAACGTTCATGATTACCAGTGCACTGCATCGTGCGGCGGATTGGGCGAAAAGCGTGTTTTCT<br>AGTGCTGCGCTGGGTGATCCGCGTCGTACCGCGCGTCTGGTGAATGTTGCGGCGCAACTGGCCAAATATAGCGGCAAAAGCATTACCATT<br>AGCAGCGAAGGCAGCAAAGCCATGCAGGAAGGCGCGTATCGTTTTATTCGTAATCCGAACGTGAGCGCGGAAGCGATTCGTAAAGCGGGT<br>GCCATGCAGACCGTGAAACTGGCCCAGGAATTTCCGGAACTGCTGGCAATTGAAGATACCACCTCTCTGAGCTATCGTCATCAGGTGGCG<br>GAAGAACTGGGCAAACTGGGTAGCATTCAGGATAAAAGCCGTGGTTGGTGGGTGCATAGCGTGCTGCTGCTGGAAGCGACCACCTTTCGT<br>ACCGTGGGCCTGCTGCATCAAGAATGGTGGATGCGTCCGGATGATCCGGCGGATGCGGATGAAAAAGAAAGCGGCAAATGGCTGGCCGCT<br>GCTGCAACTTCGCGTCTGAGAATGGGCAGCATGATGAGCAACGTGATTGCGGTGTGCGATCGTGAAGCGGATATTCATGCGTATCTGCAA<br>GATAAACTGGCCCATAACGAACGTTTTGTGGTGCGTAGCAAACATCCGCGTAAAGATGTGGAAAGCGGCCTGTATCTGTATGATCACCTG<br>AAAAACCAGCCGGAACTGGGCGGCTATCAGATTAGCATTCCGCAGAAAGGCGTGGTGGATAAACGTGGCAAACGTAAAAACCGTCCGGCG<br>CGTAAAGCGAGCCTGAGCCTGCGTAGCGGCCGTATTACCCTGAAACAGGGCAACATTACCCTGAACGCGGTGCTGGCCGAAGAAATTAAT<br>CCGCCGAAAGGCGAAACCCCGCTGAAATGGCTGCTGCTGACCAGCGAGCCGGTGGAAAGTCTGGCCCAAGCGCTGCGTGTGATTGATATT<br>TATACCCATCGTTGGCGCATTGAAGAATTTCACAAAGCGTGGAAAACGGGTGCGGGTGCGGAACGTCAGCGTATGGAAGAACCGGATAAC<br>CTGGAACGTATGGTGAGCATTCTGAGCTTTGTGGCGGTGCGTCTGCTGCAACTGCGTGAATCTTTTACTCCGCCGCAAGCACTGCGTGCG<br>CAGGGCCTGCTGAAAGAAGCGGAACACGTTGAAAGCCAGAGCGCGGAAACCGTGCTGACCCCGGATGAATGCCAACTGCTGGGCTATCTG<br>GATAAAGGCAAACGCAAACGCAAAGAAAAAGCGGGCAGCCTGCAATGGGCGTATATGGCGATTGCGCGTCTGGGCGGCTTTATGGATAGC<br>AAACGTACCGGCATTGCGAGCTGGGGTGCGCTGTGGGAAGGTTGGGAAGCGCTGCAAAGCAAACTGGATGGCTTTCTGGCCGCGAAAGAC<br>CTGATGGCGCAGGGCATTAAAATCTAATCGGCGATCGCTAGTAGCCCGCCTAATGAGCGGGCTTTTTTTTAATTCCCCTATTTGTTTATT<br>TTTCTAAATACATTCAAATATGTATCCGCTCATGAGACAATAACCCTGATAAATGCTTCAATAATATTGAAAAAGGAAGAGTATGAGCAT<br>TCAGCATTTTCGTGTGGCGCTGATTCCGTTTTTTGCGGCGTTTTGCCTGCCGGTGTTTGCGCATCCGGAAACCCTGGTGAAAGTGAAAGA<br>TGCGGAAGATCAACTGGGTGCGCGCGTGGGCTATATTGAACTGGATCTGAACAGCGGCAAAATTCTGGAATCTTTTCGTCCGGAAGAACG<br>TTTTCCGATGATGAGCACCTTTAAAGTGCTGCTGTGCGGTGCGGTTCTGAGCCGTGTGGATGCGGGCCAGGAACAACTGGGCCGTCGTAT<br>TCATTATAGCCAGAACGATCTGGTGGAATATAGCCCGGTGACCGAAAAACATCTGACCGATGGCATGACCGTGCGTGAACTGTGCAGCGC<br>GGCGATTACCATGAGCGATAACACCGCGGCGAACCTGCTGCTGACGACCATTGGCGGTCCGAAAGAACTGACCGCGTTTCTGCATAACAT<br>GGGCGATCATGTGACCCGTCTGGATCGTTGGGAACCGGAACTGAACGAAGCGATTCCGAACGATGAACGTGATACCACCATGCCGGCAGC<br>AATGGCGACCACCCTGCGTAAACTGCTGACGGGTGAGCTGCTGACCCTGGCAAGCCGCCAGCAACTGATTGATTGGATGGAAGCGGATAA<br>AGTGGCGGGTCCGCTGCTGCGTAGCGCGCTGCCGGCTGGCTGGTTTATTGCGGATAAAAGCGGTGCGGGCGAACGTGGCAGCCGTGGCAT<br>TATTGCGGCGCTGGGCCCGGATGGTAAACCGAGCCGTATTGTGGTGATTTATACCACCGGCAGCCAGGCGACGATGGATGAACGTAACCG<br>TCAGATTGCGGAAATTGGCGCGAGCCTGATTAAACATTGGTAAACCGATACAATTAAAGGCTCCTTTTGGAGCCTTTTTTTTTGGACACG<br>CGTTTGTCCTTTTCCGCTGCATAACCCTGCTTCGGGGTCATTATAGCGATTTTTTCGGTATATCCATCCTTTTTCGCACGATATACAGGA<br>TTTTGCCAAAGGGTTCGTGTAGACTTTCCTTGGTGTATCCAACGGCGTCAGCCGGGCAGGATAGGTGAAGTAGGCCCACCCGCGAGCGGG<br>TGTTCCTTCTTCACTGTCCCTTATTCGCACCTGGCGGTGCTCAACGGGAATCCTGCTCT
pYJP018	Used as p15a-*kanR* control (in figure 5)	TAGCGGAGTGTATACTGGCTTACTATGTTGGCACTGATGAGGGTGTCAGTGAAGTGCTTCATGTGGCAGGAGAAAAAAGGCTGCACCGGT<br>GCGTCAGCAGAATATGTGATACAGGATATATTCCGCTTCCTCGCTCACTGACTCGCTACGCTCGGTCGTTCGACTGCGGCGAGCGGAAAT<br>GGCTTACGAACGGGGCGGAGATTTCCTGGAAGATGCCAGGAAGATACTTAACAGGGAAGTGAGAGGGCCGCGGCAAAGCCGTTTTTCCAT<br>AGGCTCCGCCCCCCTGACAAGCATCACGAAATCTGACGCTCAAATCAGTGGTGGCGAAACCCGACAGGACTATAAAGATACCAGGCGTTT<br>CCCCTGGCGGCTCCCTCGTGCGCTCTCCTGTTCCTGCCTTTCGGTTTACCGGTGTCATTCCGCTGTTATGGCCGCGTTTGTCTCATTCCA<br>CGCCTGACACTCAGTTCCGGGTAGGCAGTTCGCTCCAAGCTGGACTGTATGCACGAACCCCCCGTTCAGTCCGACCGCTGCGCCTTATCC<br>GGTAACTATCGTCTTGAGTCCAACCCGGAAAGACATGCAAAAGCACCACTGGCAGCAGCCACTGGTAATTGATTTAGAGGAGTTAGTCTT<br>GAAGTCATGCGCCGGTTAAGGCTAAACTGAAAGGACAAGTTTTGGTGACTGCGCTCCTCCAAGCCAGTTACCTCGGTTCAAAGAGTTGGT<br>AGCTCAGAGAACCTTCGAAAAACCGCCCTGCAAGGCGGTTTTTTCGTTTTCAGAGCAAGAGATTACGCGCAGACCAAAACGATCTCAAGA<br>AGATCATCTTATTAAGGGGTCTGACGCTCAGTGGAACGAAAACTCACGTTAAGGGATTTTGGTCATGAGATTATCAAAAAGGATCTTCAC<br>CTAGATCCTTTTAAATTAAAAATGAAGTTTTAAATCAATCTAAAGTATATATGAGTAAACTTGGTCTGACAGTTACCAATGCTTAATCAG<br>TGAGGCACCTATCTCAGCGATCTGTCTATTTCGTTCATCCATAGTTGCCTGACTCCCCGTCGTGTAGATAACTACGATACGGGAGGGCTT<br>ACCATCTGGCCCCAGTGCTGCAATGATACCGCGAGACCCACGCTCACCGGCTCCAGATTTATCAGCAATAAACCAGCCAGCCGGAAGCTC<br>GAGTCCCGTCAAGTCAGCGTAATGCTCTGCCAGTGTTACAACCAATTAACCAATTCTGATTAGAAAAACTCATCGAGCATCAAATGAAAC<br>TGCAATTTATTCATATCAGGATTATCAATACCATATTTTTGAAAAAGCCGTTTCTGTAATGAAGGAGAAAACTCACCGAGGCAGTTCCAT<br>AGGATGGCAAGATCCTGGTATCGGTCTGCGATTCCGACTCGTCCAACATCAATACAACCTATTAATTTCCCCTCGTCAAAAATAAGGTTA

		TCAAGTGAGAAATCACCATGAGTGACGACTGAATCCGGTGAGAATGGCAAAAGCTTATGCATTTCTTTCCAGACTTGTTCAACAGGCCAG<br>CCATTACGCTCGTCATCAAAATCACTCGCATCAACCAAACCGTTATTCATTCGTGATTGCGCCTGAGCGAGACGAAATACGCGATCGCTG<br>TTAAAAGGACAATTACAAACAGGAATCGAATGCAACCGGCGCAGGAACACTGCCAGCGCATCAACAATATTTTCACCTGAATCAGGATAT<br>TCTTCTAATACCTGGAATGCTGTTTTCCCGGGGATCGCAGTGGTGAGTAACCATGCATCATCAGGAGTACGGATAAAATGCTTGATGGTC<br>GGAAGAGGCATAAATTCCGTCAGCCAGTTTAGTCTGACCATCTCATCTGTAACATCATTGGCAACGCTACCTTTGCCATGTTTCAGAAAC<br>AACTCTGGCGCATCGGGCTTCCCATACAATCGATAGATTGTCGCACCTGATTGCCCGACATTATCGCGAGCCCATTTATACCCATATAAA<br>TCAGCATCCATGTTGGAATTTAATCGCGGCCTCGAGCAAGACGTTTCCCGTTGAATATGGCTCATAACACCCCTTGTATTACTGTTTATG<br>TAAGCAGACAGTTTTATTGTTCATGATGATATATTTTTATCTTGTGCAATGTAACATCAGAGATTTTGAGACACAACGTGGCTTTGTTGA<br>ATAAATCGAACTTTTGCTGAGTTGAAGGATCAGATCACGCATCTTCCCGACAACGCAGACCGTTCCGTGGCAAAGCAAAAGTTCAAAATC<br>ACCAACTGGTCCACCTACAACAAAGCTCTCATCAACCGTGGCTCCCTCACTTTCTGGCTGGATGATGGGGCGATTCAGGCCTGGTATGAG<br>TCAGCAACACCTTCTTCACGAGGCAGACCTCAGCGC

a. *Annotation colors. Blue: Promoters; Purple: Ribozymes; Green: Repressors, YFP and Sensor ORFs; Red: Terminators; Orange: FRT sites and att sites; yellow highlight: RBSs; Gray highlight: antibiotic resistance gene and origin of rep; Green highlight: Transposase (Tn5, Tn7) recognition sites*

Appendix Table 7: Sequence of genome integrated constructs used In this study

Strain name	Sequence[a]
YJP_DHC404	CCCGCTGTGGGCGGACAAAATAGTTGGGAACTGGGAGGGGTGGAAATGGAGTTTTTAAGGATTATTTAGGGAAGAGTGACAAAATAGATGGGAACTGGGTGTAGC<br>GTCGTAAGCTAATACGAAAATTAAAAATGACAAAATAGTTTGGAACTAGATTTCACTTATCTGGTTGGCCTGCGATCGGTGCGGGCCTCTTCGCTATTACGCCAG<br>CTGGCGAAAGGGGGATGTGCTGCAAGGATCTCGCCAAGCTAGCTTGGCATTCGATGGTGTCAACGTAAATGCAGAAGTTCCTATACTTTCTAGAGAATAGGAACT<br>TCGGAATAGGAACTTCTTGACAGCTTATCATCGATAAACTGTAATGCGGTAGTTTATCACAGTTAAATTGCTAACGCAGTCAGGCACCGTGTATGAAATCTAACA<br>ATGCGCTCATCGTCATCCTCGGCACCGTCACCCTGGATGCTGTAGGCATAGGCTTGGTTATGCCGGTACTGCCGGGCCTCTTGCGGGATATCGTCCATTCCGACA<br>GCATCGCCAGTCACTATGGCGTGCTGCTAGCGCTATATGCGTTGATGCAATTTCTATGCGCACCCGTTCTCGGAGCACTGTCCGACCGCTTTGGCCGCCGCCCAG<br>TCCTGCTCGCTTCGCTACTTGGAGCCACTATCGACTACGCGATCATGGCGACCACACCCGTCCTGTGGATTCTCTACGCCGGACGCATCGTGGCCGGCATCACCG<br>GCGCCACAGGTGCGGTTGCTGGCGCCTATATCGCCGACATCACCGATGGGGAAGATCGGGCTCGCCACTTCGGGCTCATGAGCGCTTGTTTCGGCGTGGGTATGG<br>TGGCAGGCCCCGTGGCCGGGGGACTGTTGGGCGCCATCTCCCTGCACGCACCATTCCTTGCGGCGGCGGTGCTCAACGGCCTCAACCTACTACTGGGCTGCTTCC<br>TAATGCAGGAGTCGCATAAGGGAGAGCGCCGTCCGATGCCCTTGAGAGCCTTCAACCCAGTCAGCTCCTTCCGGTGGGCGCGGGGCATGACCATTGTGGCCGCAC<br>TTATGACTGTCTTCTTTATCATGCAACTCGTAGGACAGGTGCCGGCAGCGCTCTGGGTCATTTTCGGCGAGGACCGCTTTCGCTGGAGCGCGACGATGATCGGCC<br>TGTCGCTTGCGGTATTCGGAATCTTGCACGCCCTCGCTCAAGCCTTCGTCACTGGTCCCGCCACCAAACGTTTCGGCGAGAAGCAGGCCATTATCGCCGGCATGG<br>CGGCCGACGCGCTGGGCTACGTCTTGCTGGCGTTCGCGACGCGAGGCTGGATGGCCTTCCCCATTATGATTCTTCTCGCTTCCGGCGGCATCGGGATGCCCGCGT<br>TGCAGGCCATGCTGTCCAGGCAGGTAGATGACGACCATCAGGGACAGCTTCAAGGATCGCTCGCGGCTCTTACCAGCCTAACTTCGATCACTGGACCGCTGATCG<br>TCACGGCGATTTATGCCGCCTCGGCGAGCACATGGAACGGGTTGGCATGGATTGTAGGCGCCGCCCTATACCTTGTCTGCCTCCCCGCGTTGCGTCGCGGTGCAT<br>GGAGCCGGGCCACCTCGACCTGAGACAATTGTCCTTTTCCGCTGAAGTTCCTATACTTTCTAGAGAATAGGAACTTCGGAATAGGTACTTCCAAGCTCAGCTAAT<br>TAAGCTAGCTTATCGATACCGTCGACCTCGAACCCCACGCCCCTCTTTAATACGACGGGCAATTTGCACTTCAGAAAATGAAGAGTTTGCTTTAGCCATAACAAA<br>AGTCCAGTATGCTTTTTCACAGCATAACTGGACTGATTTCAGTTTACAACTATTCTGTCTAGTTTAAGACTTTATTGTCATAGTTTAGATCTATTTTGTTCAGTT<br>TAAGACTTTATTGTCCGCCCACACCCGC
YJP_MKC174 (without antibiotic marker *tetA*)	CCCGCTGTGGGCGGACAAAATAGTTGGGAACTGGGAGGGGTGGAAATGGAGTTTTTAAGGATTATTTAGGGAAGAGTGACAAAATAGATGGGAACTGGGTGTAGC<br>GTCGTAAGCTAATACGAAAATTAAAAATGACAAAATAGTTTGGAACTAGATTTCACTTATCTGGTTGGCCTGCGATCGGTGCGGGCCTCTTCGCTATTACGCCAG<br>CTGGCGAAAGGGGGATGTGCTGCAAGGATCTCGCCAAGCTAGCTTGGCATTCGATGGTGTCAACGTAAATGCAGTCAGAGGAGCCAAATTCAAAAAAGCCTGCTT<br>TCTAGCAGGCTTTTTGCTTTCTAATGGAAGCATAAAAAAATGGCGCCGATGGGCGCCATTTTTCACTGCGGCAAGAATTACTTCAGACAGAGTATCAAAAGGCGA<br>AACCTCCGCAATGCGGAGGTTTCTTTTTAAAGAGCACCGACCGCAGCCACAGCGTGTAGCGCTCCCAGGAGAGTTATCGACTTGCGTATTAGGGAGCGTCATTAT<br>TTACGCAGTGCCGGACTCAGACGCAGCATATCCAGACCGGTATCAACCCATTTTTCAACATCACCCAGCAGATCAACGCTATCCGGCAGCAGCAGCCAACGACGA<br>ATCAGACCATCAACATAGGCAAACATTGCAACTGCGGCACGTTCTGCATCCAGTTCACCAGGCAGCTGACCACGACGAACTACATTTGCCAGTGTCAGGGTAATA<br>CCTTTATGACAATCCAGAACTGCGCTCTGATGCTGCTGACGAATTTCACACATATCATCGGTGAACTCGCATTTGTGATGCAGGATTTCATTAATACGACGGGTA<br>CGTGCATCCAGAACCAGTTCATTAAAAACCTGCAGCAGCAGTTTACGCATACAACCCAGCGGATCAACTTCATCTTCGCTTTCGCTTGCACGTGCCAGATGATCA<br>TGGGTTTCATGCAGGCTATCCAGCAGTGCCTGAACCAGTTCGGCTTTGTTGTTAAAATGCCAATAAATTGCACCACGGGTAACGCCTGCCAGTTCTGCAATATCT<br>GCCAGGGTGGTACGTGCAACACCACGTTTATAGAATGCACGTTCTGCTGCTTCAATAATCTGTGCACGGGTTTCTTGTGCCTCTTCTTTGGTACGACGAACCATG<br>TTTTACCTACTTATCTAATTCGTAACTATTACCAATTACGTCGCGTCATGCGGATCCTATAGGGATTGATGATGCGCAATTGAACAGCCCGCGACGCGGCGGCCG<br>AGATCGTGGTGCAGCCGAGCCTGAAGCGGGCGGTTTTCAGGTAATCGCGTGTGGTATGCTCTGATATGCCCAGGATGACCGAAATATCCTTGTAATCCTTGCCGA<br>GGGCGGTCCAGTGCAGACACTCGATCTCGCGCGGCGACAATGCCGGCACCGGATCGTTTTCGCCATGCAGCTCATATACGGCCTTGCGGTGGATCAGATGGGCGA<br>TCTCGATCCACTCATTGCGGCAGCGGCGCACGAGCTCGGTCCATTCGTCGGCCGGTATATGGGCATTCAGCGACAGCAGGGCGCGGCGCTGCGCCTTGTCGGCGA<br>CGGGGATGGAGTAGCCATTGTCATCGATGCCGTGTTTCTGGGCGTCGACCAGCATGGCATAGGCCTCCGGCGTCGGTTCGACCTCGCTCCAGTCGAAGGGCAGCT<br>GGCGTTCGAAGCCCTGCTTGATGATCGGATCGACCTTCACATAGCAGTTGAGGAGGTAACGGGAAACCCAGGCATCCGGATAGGTGGTGCGCACGAAGGGCGAAT<br>CGATCTTGCTCGCGATTGTCTGGGCGAGATGGTAGGTGACGAAATCGAGGTTATATTCCGCCTGGAGAATACGGATGGCGGCATCCACGTTGGCCGCCGCTTTGA<br>TCTGTTCGAACGCGGACTCGAACTTTTCGCTATAGGTATTCTCAATCATTCGCCGTTCTCCGTTGACGGGCGTCTCGGTCAATCTGCACGAATTGACCATGCTGC<br>ACCCAGCGGTGCGCCTGCGCTTGCTGCTGCTTCAAAAACTTTTGCATTACGAATTGCTGCCAGTGCATGATCACGCATAATACGTTCTGCACCTTCGGCATCACC<br>ACAGCTAACTGCATCCAGAACTGCCTGATGCTGACGATGTGCTGCCAGCAGATGTTCATATTCGGCAGACAGGTCCATCAGATCCAGGGCCAGTGCACCGGCTGC<br>TGCAAACGGTTCAAAACCATTACGTGCCAGTGCGCTTTCAACTGCACCATTACCTGCTGCGCTAACCAGGGTATCATGAAATGCCTGATTATATGCGGCATAACG<br>ATCCAGATCTTCACCATTCAGGCGACCGGCTGCAAACAGTGCTTCACCTTCTGCAATCAGTACAACAAAACGTGCATGGGTTTCTGCGGTCATACCACGTTCTGC<br>CAGACGACGTGCTGCAAAACCTTCCAGAACACCACGAACTTCAATTGCATCACGAATCTGATCGCTGCTAACACCACGGGCTGCATAACCACGTGCACCCAGACG<br>AACAACCAGACCTTCTTGTTCCAGTGAACGCAGTGCGATACGAACCGGCATACGGCTAACACCCAGTGCTGCTGCGGTCGGAATTTCTGCAATACGTTCACCACT<br>TTTGATTTCACCGCTTGCAATCATTTTACGCAGTGCCATCATAACACGCTGACCCGGTTTAATACGAGGCATGTCCATGTCTATCCTCCTTATTTACGTTCGTTA<br>GTTTTCAACGTTTGAATTTTGCATAACGTTCACGTGCAATTTCCAGGGTGCTATTACGCACACGTTCAAAACGTTCTTTATCTTTCTGCCACAGGCTACGAACTA<br>CCAGACCACGAACGCTGTTAAAAATCAGCCACAGAATATCTTCGGCATCATCACGGCTCAGACCACGGCTCACCAGCACACCCAGCCACATATCTTCAACAACAA<br>AACGATTACGTTCAACGGTACGCTGAATACCTTCACGCAGTGCCGGATCACGATCTGCTGCAACAATCAGATCCAGGCCGATGCTAAAATCATCATCCAGAAAAA<br>ATTCTGCTGCATCATCCAGCATCTGCTGAATAACATCATCTTCCGGTTTCAGTTTTGCCAGACGTGCACGGCTACGTTCGGTAATCTGCTCATACAGCCATTCAA<br>AGGTTGCCAGCAGCAGTTCCAGTTTGGTCGGAAAATGATGGCTCTGTGCACCACGGCTAACACCGGCTGCACCCGGAACATCTGCAATACGAAAACCTGCATAAC<br>CTTTTTCACGCAGAACACCCAGTGCTGCTGCAATCAGTTTACCCTGGGTTTCCATTGCACGTTCTGCCTGGGTACGACGTTTCGGGCTCATAAGAATCTCCGTTT<br>GGTATTTACTATGAATATTCCATTGCTAGCATTATACCTAGGACTGAGCTAGCTGTCAATGGACAAACCTGTCTTGGTGCGATTCAGGCCAAAAAAAACCGGCGC<br>AATGGCCGGTTTCCGTTGTTACAGTGAAAAGAAAAAAGGCCGCAGAGCGGCCTTTTTAGTTAGATCTTATTAGGACCCACTTTCACATTTAAGTTGTTTTTCTAA<br>TCCGCATATGATCAATTCAAGGCCGAATAAGAAGGCTGGCTCTGCACCTTGGTGATCAAATAATTCGATAGCTTGTCGTAATAATGGCGGCATACTATCAGTAGT<br>AGGTGTTTCCCTTTCTTCTTTAGCGACTTGATGCTCTTGATCTTCCAATACGCAACCTAAAGTAAAATGCCCCACAGCGCTGAGTGCATATAATGCATTCTCTAG<br>TGAAAAACCTTGTTGGCATAAAAAGGCTAATTGATTTTCGAGAGTTTCATACTGTTTTTCTGTAGGCCGTGTACCTAAATGTACTTTTGCTCCATCGCGATGACT<br>TAGTAAAGCACATCTAAAACTTTTAGCGTTATTACGTAAAAAATCTTGCCAGCTTTCCCCTTCTAAAGGGCAAAAGTGAGTATGGTGCCTATCTAACATCTCAAT<br>GGCTAAGGCGTCGAGCAAAGCCCGCTTATTTTTTACATGCCAATACAATGTAGGCTGCTCTACACCTAGCTTCTGGGCGAGTTTACGGGTTGTTAAACCTTCGAT<br>TCCGACCTCATTAAGCAGCTCTAATGCGCTGTTAATCACTTTACTTTTATCTAATCTGGACATCTAGTATTTCTCCTCTTTCTCTTCACTGCCCGCTTTCCAGTC<br>GGGAAACCTGTCGTGCCAGCTGCATTAATGAATCGGCCAACGCGCGGGGAGAGGCGGTTTGCGTATTGGGCGCCAGGGTGGTTTTTCTTTTCACCAGTGAGACTG<br>GCAACAGCTGATTGCCCTTCACCGCCTGGCCCTGAGAGAGTTGCAGCAAGCGGTCCACGCTGGTTTGCCCCAGCAGGCGAAAATCCTGTTTGATGGTGGTTAACG<br>GCGGGATATAACATGAGCTATCTTCGGTATCGTCGTATCCCACTACCGAGATATCCGCACCAACGCGCAGCCCGGACTCGGTAATGGCGCGCATTGCGCCCAGCG<br>CCATCTGATCGTTGGCAACCAGCATCGCAGTGGGAACGATGCCCTCATTCAGCATTTGCATGGTTTGTTGAAAACCGGACATGGCACTCCAGTCGCCTTCCCGTT<br>CCGCTATCGGCTGAATTTGATTGCGAGTGAGATATTTATGCCAGCCAGCCAGACGCAGACGCGCCGAGACAGAACTTAATGGGCCCGCTAACAGCGCGATTTGCT<br>GGTGACCCAATGCGACCAGATGCTCCACGCCCAGTCGCGTACCGTCCTCATGGGAGAAAATAATACTGTTGATGGGTGTCTGGTCAGAGACATCAAGAAATAACG<br>CCGGAACATTAGTGCAGGCAGCTTCCACAGCAATGGCATCCTGGTCATCCAGCGGATAGTTAATGATCAGCCCACTGACGCGTTGCGCGAGAAGATTGTGCACCG<br>CCGCTTTACAGGCTTCGACGCCGCTTCGTTCTACCATCGACACCACCACGCTGGCACCCAGTTGATCGGCGCGAGATTTAATCGCCGCGACAATTTGCGACGGCG<br>CGTGCAGGGCCAGACTGGAGGTGGCAACGCCAATCAGCAACGACTGTTTGCCCGCCAGTTGTTGTGCCACGCGGTTGGGAATGTAATTCAGCTCCGCCATCGCCG<br>CTTCCACTTTTTCCCGCGTTTTCGCAGAAACGTGGCTGGCCTGGTTCACCACGCGGGAAACGGTCTGATAAGAGACACCGGCATACTCTGCGACATCGTATAACG<br>TTACTGGTTTCATATTCACCACCCTGAATTGACTCTCTTCCTTAACCGGCACGGAACTCGCTCGGGCTGGCCCCGGTGCATTTTTTAAATACCCGCGAGAAATAG<br>AGTTGATCGTCAAAACCAACATTGCGACCGACGGTGGCGATAGGCATCCGGGTGGTGCTCAAAAGCAGCTTCGCCTGGCTGATACGTTGGTCCTCGCGCCAGCTT<br>AAGACGCTAATCCCTAACTGCTGGCGGAAAAGATGTGACAGACGCGACGGCGACAAGCAAACATGCTGTGCGACGCTGGCGATATCAAAATTGCTGTCTGCCAGG<br>TGATCGCTGATGTACTGACAAGCCTCGCGTACCCGATTATCCATCGGTGGATGGAGCGACTCGTTAATCGCTTCCATGCGCCGCAGTAACAATTGCTCAAGCAGA<br>TTTATCGCCAGCAGCTCCGAATAGCGCCCTTCCCCTTGCCCGGCGTTAATGATTTGCCCAAACAGGTCGCTGAAATGCGGCTGGTGCGCTTCATCCGGGCGAAAG<br>AACCCCGTATTGGCAAATATTGACGGCCAGTTAAGCCATTCATGCCAGTAGGCGCGCGGACGAAAGTAAACCCACTGGTGATACCATTCGCGAGCCTCCGGATGA<br>CGACCGTAGTGATGAATCTCTCCTGGCGGGAACAGCAAAATATCACCCGGTCGGCAAACAAATTCTCGTCCCTGATTTTTCACCACCCCCTGACCGCGAATGGTG<br>AGATTGAGAATATAACCTTTCATTCCCAGCGGTCGGTCGATAAAAAAATCGAGATAACCGTTGGCCTCAATCGGCGTTAAACCCGCCACCAGATGGGCATTAAAC<br>GAGTATCCCGGCAGCAGGGGATCATTTTGCGCTTCAGCCATGTACGTCTCCTCGAATTGCCTGAATTGACTCTCTTCCGGGCGCTATCATGCCATACCGCGAAAG<br>GTTTTGCACCATTCGATGGCGCGCCGCTACCATTTTCTAAACTGGCCTCCCAGGCCTTAATTGGACCTCGAGTGCGGCCGCACTAGTGTCGACGGAGCTCGAATT<br>CGGATCCGAAGTTCCTATACTTTCTAGAGAATAGGAACTTCTGGACCAAAACGAAAAAGGCCCCCCTTTCGGGAGGCCTCTTTTCTGGAATTTGGTACCGAGTG<br>CAGACGTAAAAAAAGCGGCGTGGTTAGCCGCTTTTTTAATTGCCGGACAAGCTCAGCTAATTAAGCTAGCTTATCGATACCGTCGACCTCGAACCCCACGCCCCT<br>CTTTAATACGACGGGCAATTTGCACTTCAGAAAATGAAGAGTTTGCTTTAGCCATAACAAAGTCCAGTATGCTTTTTCACAGCATAACTGGACTGATTTCAGTT<br>TACAACTATTCTGTCTAGTTTAAGACTTTATTGTCATAGTTTAGATCTATTTTGTTCAGTTTAAGACTTTATTGTCCGCCCACACCCGC
YJP_MKC174 (with antibiotic marker *tetA)*	CCCGCTGTGGGCGGACAAAATAGTTGGGAACTGGGAGGGGTGGAAATGGAGTTTTTAAGGATTATTTAGGGAAGAGTGACAAAATAGATGGGAACTGGGTGTAGC<br>GTCGTAAGCTAATACGAAAATTAAAAATGACAAAATAGTTTGGAACTAGATTTCACTTATCTGGTTGGCCTGCGATCGGTGCGGGCCTCTTCGCTATTACGCCAG<br>CTGGCGAAAGGGGGATGTGCTGCAAGGATCTCGCCAAGCTAGCTTGGCATTCGATGGTGTCAACGTAAATGCAGTCAGAGGAGCCAAATTCAAAAAAGCCTGCTT<br>TCTAGCAGGCTTTTTGCTTTCTAATGGAAGCATAAAAAAATGGCGCCGATGGGCGCCATTTTTCACTGCGGCAAGAATTACTTCAGACAGAGTATCAAAAGGCGA<br>AACCTCCGCAATGCGGAGGTTTCTTTTTAAAGAGCACCGACCGCAGCCACAGCGTGTAGCGCTCCCAGGAGAGTTATCGACTTGCGTATTAGGGAGCGTCATTAT<br>TTACGCAGTGCCGGACTCAGACGCAGCATATCCAGACCGGTATCAACCCATTTTTCAACATCACCCAGCAGATCAACGCTATCCGGCAGCAGCAGCCAACGACGA<br>ATCAGACCATCAACATAGGCAAACATTGCAACTGCGGCACGTTCTGCATCCAGTTCACCAGGCAGCTGACCACGACGAACTACATTTGCCAGTGTCAGGGTAATA<br>CCTTTATGACAATCCAGAACTGCGCTCTGATGCTGCTGACGAATTTCACACATATCATCGGTGAACTCGCATTTGTGATGCAGGATTTCATTAATACGACGGGTA

	CGTGCATCCAGAACCAGTTCATTAAAAACCTGCAGCAGCAGTTTACGCATACAACCCAGCGGATCAACTTCATCTTCGCTTTCGCTTGCACGTGCCAGATGATCA<br>TGGGTTTCATGCAGGCTATCCAGCAGTGCCTGAACCAGTTCGGCTTTGTTGTTAAAATGCCAATAAATTGCACCACGGGTAACGCCTGCCAGTTCTGCAATATCT<br>GCCAGGGTGGTACGTGCAACACCACGTTTATAGAATGCACGTTCTGCTGCTTCAATAATCTGTGCACGGGTTTCTTGTGCCTCTTCTTTGGTACGACGAACCATG<br>TTTTACCTACTTATCTAATTCGTAACTATTACCAATTACGTCGCGTCATGCGGATCCTATAGGGATTGATGATGCGCAATTGAACAGCCCGCGACGCGGCGGCCG<br>AGATCGTGGTGCAGCCGAGCCTGAAGCGGGCGGTTTTCAGGTAATCGCGTGTGGTATGCTCTGATATGCCCAGGATGACCGAAATATCCTTGTAATCCTTGCCGA<br>GGGCGGTCCAGTGCAGACACTCGATCTCGCGCGGCGACAATGCCGGCACCGGATCGTTTTCGCCATGCAGCTCATATACGGCCTTGCGGTGGATCAGATGGGCGA<br>TCTCGATCCACTCATTGCGGCAGCGGCGCACGAGCTCGGTCCATTCGTCGGCCGGTATATGGGCATTCAGCGACAGCAGGGCGCGGCGCTGCGCCTTGTCGGCGA<br>CGGGGATGGAGTAGCCATTGTCATCGATGCCGTGTTTCTGGGCGTCGACCAGCATGGCATAGGCCTCCGGCGTCGGTTCGACCTCGCTCCAGTCGAAGGGCAGCT<br>GGCGTTCGAAGCCCTGCTTGATGATCGGATCGACCTTCACATAGCAGTTGAGGAGGTAACGGGAAACCCAGGCATCCGGATAGGTGGTGCGCACGAAGGGCGAAT<br>CGATCTTGCTCGCGATTGTCTGGGCGAGATGGTAGGTGACGAAATCGAGGTTATATTCCGCCTGGAGAATACGGATGGCGGCATCCACGTTGGCCGCCGCTTTGA<br>TCTGTTCGAACGCGGACTCGAACTTTTCGCTATAGGTATTCTCAATCATTCGCCGTTCTCCGTTGACGGGCGTCTCGGTCAATCTGCACGAATTGACCATGCTGC<br>ACCCAGCGGTGCGCCTGCGCTTGCTGCTGCTTCAAAAACTTTTGCATTACGAATTGCTGCCAGTGCATGATCACGCATAATACGTTCTGCACCTTCGGCATCACC<br>ACAGCTAACTGCATCCAGAACTGCCTGATGCTGACGATGTGCTGCCAGCAGATGTTCATATTCGGCAGACAGGTCCATCAGATCCAGGGCCAGTGCACCGGCTGC<br>TGCAAACGGTTCAAAACCATTACGTGCCAGTGCGCTTTCAACTGCACCATTACCTGCTGCGCTAACCAGGGTATCATGAAATGCCTGATTATATGCGGCATAACG<br>ATCCAGATCTTCACCATTCAGGCGACCGGCTGCAAACAGTGCTTCACCTTCTGCAATCAGTACAACAAAACGTGCATGGGTTTCTGCGGTCATACCACGTTCTGC<br>CAGACGACGTGCTGCAAAACCTTCCAGAACACCACGAACTTCAATTGCATCACGAATCTGATCGCTGCTAACACCACGGGCTGCATAACCACGTGCACCCAGACG<br>AACAACCAGACCTTCTTGTTCCAGTGAACGCAGTGCGATACGAACCGGCATACGGCTAACACCCAGTGCTGCTGCGGTCGGAATTTCTGCAATACGTTCACCACT<br>TTTGATTTCACCGCTTGCAATCATTTTACGCAGTGCCATCATAACACGCTGACCCGGTTTAATACGAGGCATGTCCATGTCTATCCTCCTTATTTACGTTCGTTA<br>GTTTTCAACGTTTGAATTTTGCATAACGTTCACGTGCAATTTCCAGGGTGCTATTACGCACACGTTCAAAACGTTCTTTATCTTTCTGCCACAGGCTACGAACTA<br>CCAGACCACGAACGCTGTTAAAAATCAGCCACAGAATATCTTCGGCATCATCACGGCTCAGACCACGGCTCACCAGCACACCCAGCCACATATCTTCAACAACAA<br>AACGATTACGTTCAACGGTACGCTGAATACCTTCACGCAGTGCCGGATCACGATCTGCTGCAACAATCAGATCCAGGCCGATGCTAAAATCATCATCCAGAAAAA<br>ATTCTGCTGCATCATCCAGCATCTGCTGAATAACATCATCTTCCGGTTTCAGTTTTGCCAGACGTGCACGGCTACGTTCGGTAATCTGCTCATACAGCCATTCAA<br>AGGTTGCCAGCAGCAGTTCCAGTTTGGTCGGAAAATGATGGCTCTGTGCACCACGGCTAACACCGGCTGCACCCGGAACATCTGCAATACGAAAACCTGCATAAC<br>CTTTTTCACGCAGAACACCCAGTGCTGCTGCAATCAGTTTACCCTGGGTTTCCATTGCACGTTCTGCCTGGGTACGACGTTTCGGGCTCATAAGAATCTCCGTTT<br>GGTATTTACTATGAATATTCCATTGCTAGCATTATACCTAGGACTGAGCTAGCTGTCAATGGACAAACCTGTCTTGGTGCGATTCAGGCCAAAAAAAACCGGCGC<br>AATGGCCGGTTTCCGTTGTTACAGTGAAAAGAAAAAAGGCCGCAGAGCGGCCTTTTTAGTTAGATCTTATTAGGACCCACTTTCACATTTAAGTTGTTTTTCTAA<br>TCCGCATATGATCAATTCAAGGCCGAATAAGAAGGCTGGCTCTGCACCTTGGTGATCAAATAATTCGATAGCTTGTCGTAATAATGGCGGCATACTATCAGTAGT<br>AGGTGTTTCCCTTTCTTCTTTAGCGACTTGATGCTCTTGATCTTCCAATACGCAACCTAAAGTAAAATGCCCCACAGCGCTGAGTGCATATAATGCATTCTCTAG<br>TGAAAAACCTTGTTGGCATAAAAAGGCTAATTGATTTTCGAGAGTTTCATACTGTTTTTCTGTAGGCCGTGTACCTAAATGTACTTTTGCTCCATCGCGATGACT<br>TAGTAAAGCACATCTAAAACTTTTAGCGTTATTACGTAAAAAATCTTGCCAGCTTTCCCCTTCTAAAGGGCAAAAGTGAGTATGGTGCCTATCTAACATCTCAAT<br>GGCTAAGGCGTCGAGCAAAGCCCGCTTATTTTTTACATGCCAATACAATGTAGGCTGCTCTACACCTAGCTTCTGGGCGAGTTTACGGGTTGTTAAACCTTCGAT<br>TCCGACCTCATTAAGCAGCTCTAATGCGCTGTTAATCACTTTACTTTTATCTAATCTGGACATCTAGTATTTCTCCTCTTTCTCTTCACTGCCCGCTTTCCAGTC<br>GGGAAACCTGTCGTGCCAGCTGCATTAATGAATCGGCCAACGCGCGGGGAGAGGCGGTTTGCGTATTGGGCGCCAGGGTGGTTTTTCTTTTCACCAGTGAGACTG<br>GCAACAGCTGATTGCCCTTCACCGCCTGGCCCTGAGAGAGTTGCAGCAAGCGGTCCACGCTGGTTTGCCCCAGCAGGCGAAAATCCTGTTTGATGGTGGTTAACG<br>GCGGGATATAACATGAGCTATCTTCGGTATCGTCGTATCCCACTACCGAGATATCCGCACCAACGCGCAGCCCGGACTCGGTAATGGCGCGCATTGCGCCCAGCG<br>CCATCTGATCGTTGGCAACCAGCATCGCAGTGGGAACGATGCCCTCATTCAGCATTTGCATGGTTTGTTGAAAACCGGACATGGCACTCCAGTCGCCTTCCCGTT<br>CCGCTATCGGCTGAATTTGATTGCGAGTGAGATATTTATGCCAGCCAGCCAGACGCAGACGCGCCGAGACAGAACTTAATGGGCCCGCTAACAGCGCGATTTGCT<br>GGTGACCCAATGCGACCAGATGCTCCACGCCCAGTCGCGTACCGTCCTCATGGGAGAAAATAATACTGTTGATGGGTGTCTGGTCAGAGACATCAAGAAATAACG<br>CCGGAACATTAGTGCAGGCAGCTTCCACAGCAATGGCATCCTGGTCATCCAGCGGATAGTTAATGATCAGCCCACTGACGCGTTGCGCGAGAAGATTGTGCACCG<br>CCGCTTTACAGGCTTCGACGCCGCTTCGTTCTACCATCGACACCACCACGCTGGCACCCAGTTGATCGGCGCGAGATTTAATCGCCGCGACAATTTGCGACGGCG<br>CGTGCAGGGCCAGACTGGAGGTGGCAACGCCAATCAGCAACGACTGTTTGCCCGCCAGTTGTTGTGCCACGCGGTTGGGAATGTAATTCAGCTCCGCCATCGCCG<br>CTTCCACTTTTTCCCGCGTTTTCGCAGAAACGTGGCTGGCCTGGTTCACCACGCGGGAAACGGTCTGATAAGAGACACCGGCATACTCTGCGACATCGTATAACG<br>TTACTGGTTTCATATTCACCACCCTGAATTGACTCTCTTCCTTAACCGGCACGGAACTCGCTCGGGCTGGCCCCGGTGCATTTTTTAAATACCCGCGAGAAATAG<br>AGTTGATCGTCAAAACCAACATTGCGACCGACGGTGGCGATAGGCATCCGGGTGGTGCTCAAAAGCAGCTTCGCCTGGCTGATACGTTGGTCCTCGCGCCAGCTT<br>AAGACGCTAATCCCTAACTGCTGGCGGAAAAGATGTGACAGACGCGACGGCGACAAGCAAACATGCTGTGCGACGCTGGCGATATCAAAATTGCTGTCTGCCAGG<br>TGATCGCTGATGTACTGACAAGCCTCGCGTACCCGATTATCCATCGGTGGATGGAGCGACTCGTTAATCGCTTCCATGCGCCGCAGTAACAATTGCTCAAGCAGA<br>TTTATCGCCAGCAGCTCCGAATAGCGCCCTTCCCCTTGCCCGGCGTTAATGATTTGCCCAAACAGGTCGCTGAAATGCGGCTGGTGCGCTTCATCCGGGCGAAAG<br>AACCCCGTATTGGCAAATATTGACGGCCAGTTAAGCCATTCATGCCAGTAGGCGCGCGGACGAAAGTAAACCCACTGGTGATACCATTCGCGAGCCTCCGGATGA<br>CGACCGTAGTGATGAATCTCTCCTGGCGGGAACAGCAAAATATCACCCGGTCGGCAAACAAATTCTCGTCCCTGATTTTTCACCACCCCCTGACCGCGAATGGTG<br>AGATTGAGAATATAACCTTTCATTCCCAGCGGTCGGTCGATAAAAAAATCGAGATAACCGTTGGCCTCAATCGGCGTTAAACCCGCCACCAGATGGGCATTAAAC<br>GAGTATCCCGGCAGCAGGGGATCATTTTGCGCTTCAGCCATGTACGTCTCCTCGAATTGCCTGAATTGACTCTCTTCCGGGCGCTATCATGCCATACCGCGAAAG<br>GTTTTGCACCATTCGATGGCGCGCCGCTACCATTTTCTAAACTGGCCTCCCAGGCCTTAATTGGACCTCGAGTGCGGCCGCACTAGTGTCGACGGAGCTCGAATT<br>CGGATCCGAAGTTCCTATACTTTCTAGAGAATAGGAACTTCGGAATAGGAACTTCTCAGAGATTTTATGCTTGTAAACCGTTTTGTGAAAAAATTTTTAAAATAA<br>AAAAGGGGACCTCTAGGGTCCGCAATTAATTAGACAATTGTCTCAGGTCGAGGTGGCCCGGCTCCATGCACCGCGACGCAACGCGGGGAGGCAGACAAGGTATAG<br>GGCGGCGCCTACAATCCATGCCAACCCGTTCCATGTGCTCGCCGAGGCGGCATAAATCGCCGTGACGATCAGCGGTCCAGTGATCGAAGTTAGGCTGGTAAGAGC<br>CGCGAGCGATCCTTGAAGCTGTCCCTGATGGTCGTCATCTACCTGCCTGGACAGCATGGCCTGCAACGCGGGCATCCCGATGCCGCCGGAAGCGAGAAGAATCAT<br>AATGGGGAAGGCCATCCAGCCTCGCGTCGCGAACGCCAGCAAGACGTAGCCCAGCGCGTCGGCCGCCATGCCGGCGATAATGGCCTGCTTCTCGCCGAAACGTTT<br>GGTGGCGGGACCAGTGACGAAGGCTTGAGCGAGGGCGTGCAAGATTCCGAATACCGCAAGCGACAGGCCGATCATCGTCGCGCTCCAGCGAAAGCGGTCCTCGCC<br>GAAAATGACCCAGAGCGCTGCCGGCACCTGTCCTACGAGTTGCATGATAAAGAAGACAGTCATAAGTGCGGCCACAATGGTCATGCCCCGCGCCCACCGGAAGGA<br>GCTGACTGGGTTGAAGGCTCTCAAGGGCATCGGACGGCGCTCTCCCTTATGCGACTCCTGCATTAGGAAGCAGCCCAGTAGTAGGTTGAGGCCGTTGAGCACCGC<br>CGCCGCAAGGAATGGTGCGTGCAGGGAGATGGCGCCCAACAGTCCCCCGGCCACGGGGCCTGCCACCATACCCACGCCGAAACAAGCGCTCATGAGCCCGAAGTG<br>GCGAGCCCGATCTTCCCCATCGGTGATGTCGGCGATATAGGCGCCAGCAACCGCACCTGTGGCGCCGGTGATGCCGGCCACGATGCGTCCGGCGTAGAGAATCCA<br>CAGGACGGGTGTGGTCGCCATGATCGCGTAGTCGATAGTGGCTCCAAGTAGCGAAGCGAGCAGGACTGGGCGGCGGCCAAAGCGGTCGGACAGTGCTCCGAGAAC<br>GGGTGCGCATAGAAATTGCATCAACGCATATAGCGCTAGCAGCACGCCATAGTGACTGGCGATGCTGTCGGAATGGACGATATCCCGCAAGAGGCCCGGCAGTAC<br>CGGCATAACCAAGCCTATGCCTACAGCATCCAGGGTGACGGTGCCGAGGATGACGATGAGCGCATTGTTAGATTTCATACACGGTGCCTGACTGCGTTAGCAATT<br>TAACTGTGATAAACTACCGCATTACAGTTTATCGATGATAAGCTGTCAATCCGATTCGCCAGATGATAAGGAACCATAAAAAAATGGCGCCGATGGGCGCCATTT<br>TTCACTGGTTGATTGCTTTGGTTGAGACGAGGTGTCATACGTAAAAAAGGGCGATCTTGCGACCGCCCTTTTTTTATTAAATGTTGGATTTGTTCAGAACGCTCG<br>GTTGCCGCCGGGCGTTTTTTATTGGTGAGAATCCAAGCACAAGAAGATCCGGCCACGATGCGTCCGGCGTAGAGGATCTGAAGATCAGCAGTTCAACCTGTTGAT<br>AGTACGTACTAAGCTCTCATGTTTCACGTACTAAGCTCTCATGTTTAACGTACTAAGCTCTCATGTTTAACGAACTAAACCCTCATGGCTAACGTACTAAGCTCT<br>CATGGCTAACGTACTAAGCTCTCATGTTTCACGTACTAAGCTCTCATGTTTGAACAATAAAATTAATATAAATCAGCAACTTAAATAGCCTCTAAGGTTTTAAGT<br>TTTATAAGAAAAAAAAGAATATATAAGGCTTTTAAAGCTTTTAAGGTTTAACGGTTGTGGACAACAAGCCAGGGATGTAACGCACTGAGAAGCCCTTAGAGCCTC<br>TCAAAGCAATTTTGAGTGACACAGGAACACTTAACGGCTGACATGGGAATTAGCCATGGGCCCGTGCGAATCATTTTTACGGTTCCTGGCCTTTTGCTGGCCTTT<br>TGCTCACATGTTCTTTCCTGCGTTATCCCCTGATTCTGTGGATAACCGTATTACCGCCTGGACCAAAACGAAAAAACACCCTTTCGGGTGTCTTTTCTGGAATTT<br>GGTACCGAGCCTTTGGTCGAAAAAAAAAGCCCGCACTGTCAGGTGCGGGCTTTTTTCTGTGTTTCCGCACCGACCGCAGCCACAGCGTGTAGCGCTCCCAGGCCG<br>TCCACTCCCTGATCCGGCGCTCGAAGTTCCTATACTTTCTAGAGAATAGGAACTTCTGGACCAAAACGAAAAAGGCCCCCCTTTCGGAGGCCTCTTTTCTGGA<br>ATTTGGTACCGAGTGCAGACGTAAAAAAAGCGGCGTGGTTAGCCGCTTTTTTAATTGCCGGACAAGCTCAGCTAATTAAGCTAGCTTATCGATACCGTCGACCTC<br>GAACCCCACGCCCCTCTTTAATACGACGGGCAATTTGCACTTCAGAAAATGAAGAGTTTGCTTTAGCCATAACAAAAGTCCAGTATGCTTTTTCACAGCATAACT<br>GGACTGATTTCAGTTTACAACTATTCTGTCTAGTTTAAGACTTTATTGTCATAGTTTAGATCTATTTTGTTCAGTTTAAGACTTTATTGTCCGCCCACACCCGC
YJP_MKC108_LP#1	AAAAAAAACCCCGCCCTGTCAGGGGCGGGGTTTTTTTTTTTCTTTTGGGTATAGCGTCGTGGACAGTCATTCATCTTTCTGCCCCTCCAAAAGCAAAAACCCGC<br>CGAAGCGGGTTTTACGTAAATCAGGTGAAACTGACCGATAAGCCGGGACGAGCTGAGCAGTATGTCGACGGTCCGGCGAGAATCTCGCGTAGACACATACATGA<br>GCTTACTTGTACAGCTCGTCCATGCCGAGAGTGATCCCGGCGGCGGTCACGAACTCCAGCAGGACCATGTGATCGCGCTTCTCGTTGGGGTCTTTGCTCAGGGCG<br>GACTGGTAGCTAAGGTAGTGGTTGTCGGGCAGCAGCACGGGGCCGTCGCCGATTGGGGTGTTCTGCTGGTAGTGGTCGGCGAGCTGCACGCTGCCGTCCTCGATG<br>TTGTGGCGGATCTTGAAGTTCACCTTGATGCCGTTCTTCTGCTTGTCGGCCATGATATAGACGTTGTGGCTGTTGTAGTTGTACTCCAGCTTGTGCCCCAGGATG<br>TTGCCGTCCTCCTTGAAGTCGATGCCCTTCAGCTCGATGCGGTTCACCAGGGTGTCGCCCTCGAACTTCACCTCGGCGCGGGTCTTGTAGTTGCCGTCGTCCTTG<br>AAGAAGATGGTGCGCTCCTGGACGTAGCCTTCGGGCATGGCGGACTTGAAGAAGTCGTGCAGCTTCATGTGGTCGGGGTAGCGGGCGAAGCATTGCAGGCCGTAG<br>CCGAAGGTGGTCACGAGGGTGGGCCAGGGCACGGGCAGCTTGCCTGTGGTGCAGATGAACTTCAGGGTCAGCTTGCCGTAGGTGGCATCGCCCTCGCCCTCGCCG<br>GACACGCTGAACTTGTGGCCGTTTACGTCGCCGTCCAGCTCGACCAGGATGGGCACCACCCCGGTGAACAGCTCCTCGCCCTTGCTCACCATCTAGTATTTCTCC<br>TCTTTCTCTAGTATTAAACAAAATTATTTGTAGAGGCTGTTTCGTCCTCACGGACTCATCAGACCGGAAAGCACATCCGGTGACAGCTAATTGTGAGCGCTCACA<br>ATTCCACACATTATACGAGCCGATGATTAATTGTCAACACAGCCAACGATCGTTCTCCTGGTCTTCCAGTACAATCTGCTCTGATGCCCTCGAGTGCGGCCGCAC<br>TAGTGTCGACGGAGCTCGAATTCGGATCCGAAGTTCCTATACTTTCTAGAGAATAGGAACTTCGGAATAGGAACTTCTATTTTTATAGGTTAATGTCATGATAAT<br>AATGGTTTCTTAGACCATGCCTATTTGTTTATTTTTCTAAATACATTCAAATATGTATCCGCTCATGAGACAATAACCCTGATAAATGCTTCAATAATATTGAAA<br>AAGGAAGAGTATGAGCCATATTCAACGGGAAACGTCTTGCTCTAGGCCGCGATTAAATTCCAACATGGATGCTGATTTATATGGGTATAAATGGGCTCGCGATAA<br>TGTCGGGCAATCAGGTGCGACAATCTATCGATTGTATGGGAAGCCCGATGCGCCAGAGTTGTTTCTGAAACATGGCAAAGGTAGCGTTGCCAATGATGTTACAGA<br>TGAGATGGTCAGACTAAACTGGCTGACGGAATTTATGCCTCTTCCGACCATCAAGCATTTTATCCGTACTCCTGATGATGCATGGTTACTCACCACTGCGATCCC<br>CGGGAAAACAGCATTCCAGGTATTAGAAGAATATCCTGATTCAGGTGAAAATATTGTTGATGCGCTGGCAGTGTTCCTGCGCCGGTTGCATTCGATTCCTGTTTG

	TAATTGTCCTTTTAACAGCGACCGCGTATTTCGTCTCGCTCAGGCGCAATCACGAATGAATAACGGTTTGGTTGATGCGAGTGATTTTGATGACGAGCGTAATGG<br>CTGGCCTGTTGAACAAGTCTGGAAAGAAATGCATAAACTTTTGCCATTCTCACCGGATTCAGTCGTCACTCATGGTGATTTCTCACTTGATAACCTTATTTTTGA<br>CGAGGGGAAATTAATAGGTTGTATTGATGTTGGACGAGTCGGAATCGCAGACCGATACCAGGATCTTGCCATCCTATGGAACTGCCTCGGTGAGTTTTCTCCTTC<br>ATTACAGAAACGGCTTTTTCAAAAATATGGTATTGATAATCCTGATATGAATAAATTGCAGTTTCATTTGATGCTCGATGAGTTTTTCTAAAGCTCGCTTGGACT<br>CCTGTTGATAGATCCAGTAATGACCTCAGAACTCCATCTGGATTTGTTCAGAACGCTCGGTTGCCGCCGGGCGTTTTTTATTGGTGAGAATCCAAGCACAAGTTC<br>ACGTACTAAGCTCTCATGTTTAACGTACTAAGCTCTCATGTTTAACGAACTAAACCCTCATGGCTAACGTACTAAGCTCTCATGGCTAACGTACTAAGCTCTCAT<br>GTTTCACGTACTAAGCTCTCATGTTTGAACAATAAAATTAATATAAATCAGCAACTTAAATAGCCTCTAAGGTTTTAAGTTTTATAAGAAAAAAAAGAATATATA<br>AGGCTTTTAAAGCTTTTAAGGTTTAACGGTTGTGGACAACAAGCCAGGGATGTAACGCACTGAGAAGCCCTTAGAGCCTCTCAAAGCAATTTTGAGTGACACAGG<br>AACACTTAACGGCTGACATGGGAATTAGCCATGGGCCCGTGCGAATCATTTTTACGGTTCCTGGCCTTTTGCTGGCCTTTTGCTCACATGTTCTTTCCTGCGTGG<br>ATAACCGTATTACCGCCTGGACCAAAACGAAAAAACACCCTTTCGGGTGTCTTTTCTGGAATTTGGTACCGAGCCTTTGGTCGAAAAAAAAAGCCCGCACTGTCA<br>GGTGCGGGCTTTTTTCTGTGTTTCCAGCCAAGAGCAGTGCCTTGCAGAAGTTCTCGGGCGAAGAGCTACTCCCCTTCTGCGCCGTCCGAAGTTCCTATACTTTCT<br>AGAGAATAGGAACTTCGGACCAAAACGAAAAAACACCCTTTCGGGTGTCTTTTCTGGAATTTGGTACCGAGCCTTTGGTCGAAAAAAAAAGCCCGCACTGTCAGG<br>TGCGGGCTTTTTTCTGTGTTTCCCGTTAACAGCGCAGAACTCAAGCAGGATGTTTGACGCGGGCGGCGTCAAG
YJP_MKC110_LP#1	AAAAAAAACCCCGCCCTGTCAGGGGCGGGGTTTTTTTTTTTTCTTTTGGGTATAGCGTCGTGGACAGTCATTCATCTTTCTGCCCCTCCAAAAGCAAAAACCCGC<br>CGAAGCGGGTTTTTACGTAAATCAGGTGAAACTGACCGATAAGCCGGGACGAGCTGAGCAGTATGTCGACGGTCCGGCGAGAATCTCGCGTAGACACATACATGA<br>GCTTACTTGTACAGCTCGTCCATGCCGAGAGTGATCCCGGCGGCGGTCACGAACTCCAGCAGGACCATGTGATCGCGCTTCTCGTTGGGGTCTTTGCTCAGGGCG<br>GACTGGTAGCTAAGGTAGTGGTTGTCGGGCAGCAGCACGGGGCCGTCGCCGATTGGGGTGTTCTGCTGGTAGTGGTCGGCGAGCTGCACGCTGCCGTCCTCGATG<br>TTGTGGCGGATCTTGAAGTTCACCTTGATGCCGTTCTTCTGCTTGTCGGCCATGATATAGACGTTGTGGCTGTTGTAGTTGTACTCCAGCTTGTGCCCCAGGATG<br>TTGCCGTCCTCCTTGAAGTCGATGCCCTTCAGCTCGATGCGGTTCACCAGGGTGTCGCCCTCGAACTTCACCTCGGCGCGGGTCTTGTAGTTGCCGTCGTCCTTG<br>AAGAAGATGGTGCGCTCCTGGACGTAGCCTTCGGGCATGGCGGACTTGAAGAAGTCGTGCAGCTTCATGTGGTCGGGGTAGCGGGCGAAGCATTGCAGGCCGTAG<br>CCGAAGGTGGTCACGAGGGTGGGCCAGGGCACGGGCAGCTTGCCTGTGGTGCAGATGAACTTCAGGGTCAGCTTGCCGTAGGTGGCATCGCCCTCGCCCTCGCCG<br>GACACGCTGAACTTGTGGCCGTTTACGTCGCCGTCCAGCTCGACCAGGATGGGCACCACCCCGGTGAACAGCTCCTCGCCCTTGCTCACCATCTAGTATTTCTCC<br>TCTTTCTCTAGTATTAAACAAAATTATTTGTAGAGGCTGTTTCGTCCTCACGGACTCATCAGACCGGAAAGCACATCCGGTGACAGCTCGGGTATGGAGAAAAAT<br>ATAGAGAGTTGCGATAAAAAGCGTCAGGTAGGATCCGCTAATCTTATGGATAAAAATGCTACAGCCAACGATCGTTCTCCTGGTCTTCCAGTACAATCTGCTCTG<br>ATGCCCTCGAGTGCGGCCGCACTAGTGTCGACGGAGCTCGAATTCGGATCCGAAGTTCCTATACTTTCTAGAGAATAGGAACTTCGGAATAGGAACTTCTATTTT<br>TATAGGTTAATGTCATGATAATAATGGTTTCTTAGACCATGCCTATTTGTTTATTTTTCTAAATACATTCAAATATGTATCCGCTCATGAGACAATAACCCTGAT<br>AAATGCTTCAATAATATTGAAAAAGGAAGAGTATGAGCCATATTCAACGGGAAACGTCTTGCTCTAGGCCGCGATTAAATTCCAACATGGATGCTGATTTATATG<br>GGTATAAATGGGCTCGCGATAATGTCGGGCAATCAGGTGCGACAATCTATCGATTGTATGGGAAGCCCGATGCGCCAGAGTTGTTTCTGAAACATGGCAAAGGTA<br>GCGTTGCCAATGATGTTACAGATGAGATGGTCAGACTAAACTGGCTGACGGAATTTATGCCTCTTCCGACCATCAAGCATTTTATCCGTACTCCTGATGATGCAT<br>GGTTACTCACCACTGCGATCCCCGGGAAAACAGCATTCCAGGTATTAGAAGAATATCCTGATTCAGGTGAAAATATTGTTGATGCGCTGGCAGTGTTCCTGCGCC<br>GGTTGCATTCGATTCCTGTTTGTAATTGTCCTTTTAACAGCGACCGCGTATTTCGTCTCGCTCAGGCGCAATCACGAATGAATAACGGTTTGGTTGATGCGAGTG<br>ATTTTGATGACGAGCGTAATGGCTGGCCTGTTGAACAAGTCTGGAAAGAAATGCATAAACTTTTGCCATTCTCACCGGATTCAGTCGTCACTCATGGTGATTTCT<br>CACTTGATAACCTTATTTTTGACGAGGGGAAATTAATAGGTTGTATTGATGTTGGACGAGTCGGAATCGCAGACCGATACCAGGATCTTGCCATCCTATGGAACT<br>GCCTCGGTGAGTTTTCTCCTTCATTACAGAAACGGCTTTTTCAAAAATATGGTATTGATAATCCTGATATGAATAAATTGCAGTTTCATTTGATGCTCGATGAGT<br>TTTTCTAAAGCTCGCTTGGACTCCTGTTGATAGATCCAGTAATGACCTCAGAACTCCATCTGGATTTGTTCAGAACGCTCGGTTGCCGCCGGGCGTTTTTTATTG<br>GTGAGAATCCAAGCACAAGTTCACGTACTAAGCTCTCATGTTTAACGTACTAAGCTCTCATGTTTAACGAACTAAACCCTCATGGCTAACGTACTAAGCTCTCAT<br>GGCTAACGTACTAAGCTCTCATGTTTCACGTACTAAGCTCTCATGTTTGAACAATAAAATTAATATAAATCAGCAACTTAAATAGCCTCTAAGGTTTTAAGTTTT<br>ATAAGAAAAAAAAGAATATATAAGGCTTTTAAAGCTTTTAAGGTTTAACGGTTGTGGACAACAAGCCAGGGATGTAACGCACTGAGAAGCCCTTAGAGCCTCTCA<br>AAGCAATTTTGAGTGACACAGGAACACTTAACGGCTGACATGGGAATTAGCCATGGGCCCGTGCGAATCATTTTTACGGTTCCTGGCCTTTTGCTGGCCTTTTGC<br>TCACATGTTCTTTCCTGCGTGGATAACCGTATTACCGCCTGGACCAAAACGAAAAAACACCCTTTCGGGTGTCTTTTCTGGAATTTGGTACCGAGCCTTTGGTCG<br>AAAAAAAAAGCCCGCACTGTCAGGTGCGGGCTTTTTTCTGTGTTTCCAGCCAAGAGCAGTGCCTTGCAGAAGTTCTCGGGCGAAGAGCTACTCCCCTTCTGCGCC<br>GTCCGAAGTTCCTATACTTTCTAGAGAATAGGAACTTCGGACCAAAACGAAAAAACACCCTTTCGGGTGTCTTTTCTGGAATTTGGTACCGAGCCTTTGGTCGAA<br>AAAAAAAGCCCGCACTGTCAGGTGCGGGCTTTTTTCTGTGTTTCCCGTTAACAGCGCAGAACTCAAGCAGGATGTTTGACGCGGGCGGCGTCAAG
YJP_MKC111_LP#1	AAAAAAAACCCCGCCCTGTCAGGGGCGGGGTTTTTTTTTTTTCTTTTGGGTATAGCGTCGTGGACAGTCATTCATCTTTCTGCCCCTCCAAAAGCAAAAACCCGC<br>CGAAGCGGGTTTTTACGTAAATCAGGTGAAACTGACCGATAAGCCGGGACGAGCTGAGCAGTATGTCGACGGTCCGGCGAGAATCTCGCGTAGACACATACATGA<br>GCTTACTTGTACAGCTCGTCCATGCCGAGAGTGATCCCGGCGGCGGTCACGAACTCCAGCAGGACCATGTGATCGCGCTTCTCGTTGGGGTCTTTGCTCAGGGCG<br>GACTGGTAGCTAAGGTAGTGGTTGTCGGGCAGCAGCACGGGGCCGTCGCCGATTGGGGTGTTCTGCTGGTAGTGGTCGGCGAGCTGCACGCTGCCGTCCTCGATG<br>TTGTGGCGGATCTTGAAGTTCACCTTGATGCCGTTCTTCTGCTTGTCGGCCATGATATAGACGTTGTGGCTGTTGTAGTTGTACTCCAGCTTGTGCCCCAGGATG<br>TTGCCGTCCTCCTTGAAGTCGATGCCCTTCAGCTCGATGCGGTTCACCAGGGTGTCGCCCTCGAACTTCACCTCGGCGCGGGTCTTGTAGTTGCCGTCGTCCTTG<br>AAGAAGATGGTGCGCTCCTGGACGTAGCCTTCGGGCATGGCGGACTTGAAGAAGTCGTGCAGCTTCATGTGGTCGGGGTAGCGGGCGAAGCATTGCAGGCCGTAG<br>CCGAAGGTGGTCACGAGGGTGGGCCAGGGCACGGGCAGCTTGCCTGTGGTGCAGATGAACTTCAGGGTCAGCTTGCCGTAGGTGGCATCGCCCTCGCCCTCGCCG<br>GACACGCTGAACTTGTGGCCGTTTACGTCGCCGTCCAGCTCGACCAGGATGGGCACCACCCCGGTGAACAGCTCCTCGCCCTTGCTCACCATCTAGTATTTCTCC<br>TCTTTCTCTAGTATTAAACAAAATTATTTGTAGAGGCTGTTTCGTCCTCACGGACTCATCAGACCGGAAAGCACATCCGGTGACAGCTGTGCTCATTATATCTAT<br>CACTGATAGGGATGTCAATCTCTATCACTGATAGGGACAGCCAACGATCGTTCTCCTGGTCTTCCAGTACAATCTGCTCTGATGCCCTCGAGTGCGGCCGCACTA<br>GTGTCGACGGAGCTCGAATTCGGATCCGAAGTTCCTATACTTTCTAGAGAATAGGAACTTCGGAATAGGAACTTCTATTTTTATAGGTTAATGTCATGATAATAA<br>TGGTTTCTTAGACCATGCCTATTTGTTTATTTTTCTAAATACATTCAAATATGTATCCGCTCATGAGACAATAACCCTGATAAATGCTTCAATAATATTGAAAAA<br>GGAAGAGTATGAGCCATATTCAACGGGAAACGTCTTGCTCTAGGCCGCGATTAAATTCCAACATGGATGCTGATTTATATGGGTATAAATGGGCTCGCGATAATG<br>TCGGGCAATCAGGTGCGACAATCTATCGATTGTATGGGAAGCCCGATGCGCCAGAGTTGTTTCTGAAACATGGCAAAGGTAGCGTTGCCAATGATGTTACAGATG<br>AGATGGTCAGACTAAACTGGCTGACGGAATTTATGCCTCTTCCGACCATCAAGCATTTTATCCGTACTCCTGATGATGCATGGTTACTCACCACTGCGATCCCCG<br>GGAAAACAGCATTCCAGGTATTAGAAGAATATCCTGATTCAGGTGAAAATATTGTTGATGCGCTGGCAGTGTTCCTGCGCCGGTTGCATTCGATTCCTGTTTGTA<br>ATTGTCCTTTTAACAGCGACCGCGTATTTCGTCTCGCTCAGGCGCAATCACGAATGAATAACGGTTTGGTTGATGCGAGTGATTTTGATGACGAGCGTAATGGCT<br>GGCCTGTTGAACAAGTCTGGAAAGAAATGCATAAACTTTTGCCATTCTCACCGGATTCAGTCGTCACTCATGGTGATTTCTCACTTGATAACCTTATTTTTGACG<br>AGGGGAAATTAATAGGTTGTATTGATGTTGGACGAGTCGGAATCGCAGACCGATACCAGGATCTTGCCATCCTATGGAACTGCCTCGGTGAGTTTTCTCCTTCAT<br>TACAGAAACGGCTTTTTCAAAAATATGGTATTGATAATCCTGATATGAATAAATTGCAGTTTCATTTGATGCTCGATGAGTTTTTCTAAAGCTCGCTTGGACTCC<br>TGTTGATAGATCCAGTAATGACCTCAGAACTCCATCTGGATTTGTTCAGAACGCTCGGTTGCCGCCGGGCGTTTTTTATTGGTGAGAATCCAAGCACAAGTTCAC<br>GTACTAAGCTCTCATGTTTAACGTACTAAGCTCTCATGTTTAACGAACTAAACCCTCATGGCTAACGTACTAAGCTCTCATGGCTAACGTACTAAGCTCTCATGT<br>TTCACGTACTAAGCTCTCATGTTTGAACAATAAAATTAATATAAATCAGCAACTTAAATAGCCTCTAAGGTTTTAAGTTTTATAAGAAAAAAAAGAATATATAAG<br>GCTTTTAAAGCTTTTAAGGTTTAACGGTTGTGGACAACAAGCCAGGGATGTAACGCACTGAGAAGCCCTTAGAGCCTCTCAAAGCAATTTTGAGTGACACAGGAA<br>CACTTAACGGCTGACATGGGAATTAGCCATGGGCCCGTGCGAATCATTTTTACGGTTCCTGGCCTTTTGCTGGCCTTTTGCTCACATGTTCTTTCCTGCGTGGAT<br>AACCGTATTACCGCCTGGACCAAAACGAAAAAACACCCTTTCGGGTGTCTTTTCTGGAATTTGGTACCGAGCCTTTGGTCGAAAAAAAAAGCCCGCACTGTCAGG<br>TGCGGGCTTTTTTCTGTGTTTCCAGCCAAGAGCAGTGCCTTGCAGAAGTTCTCGGGCGAAGAGCTACTCCCCTTCTGCGCCGTCCGAAGTTCCTATACTTTCTAG<br>AGAATAGGAACTTCGGACCAAAACGAAAAAACACCCTTTCGGGTGTCTTTTCTGGAATTTGGTACCGAGCCTTTGGTCGAAAAAAAAAGCCCGCACTGTCAGGTG<br>CGGGCTTTTTTCTGTGTTTCCCGTTAACAGCGCAGAACTCAAGCAGGATGTTTGACGCGGGCGGCGTCAAG
YJP_MKC112_LP#1	AAAAAAAACCCCGCCCTGTCAGGGGCGGGGTTTTTTTTTTTTCTTTTGGGTATAGCGTCGTGGACAGTCATTCATCTTTCTGCCCCTCCAAAAGCAAAAACCCGC<br>CGAAGCGGGTTTTTACGTAAATCAGGTGAAACTGACCGATAAGCCGGGACGAGCTGAGCAGTATGTCGACGGTCCGGCGAGAATCTCGCGTAGACACATACATGA<br>GCTTACTTGTACAGCTCGTCCATGCCGAGAGTGATCCCGGCGGCGGTCACGAACTCCAGCAGGACCATGTGATCGCGCTTCTCGTTGGGGTCTTTGCTCAGGGCG<br>GACTGGTAGCTAAGGTAGTGGTTGTCGGGCAGCAGCACGGGGCCGTCGCCGATTGGGGTGTTCTGCTGGTAGTGGTCGGCGAGCTGCACGCTGCCGTCCTCGATG<br>TTGTGGCGGATCTTGAAGTTCACCTTGATGCCGTTCTTCTGCTTGTCGGCCATGATATAGACGTTGTGGCTGTTGTAGTTGTACTCCAGCTTGTGCCCCAGGATG<br>TTGCCGTCCTCCTTGAAGTCGATGCCCTTCAGCTCGATGCGGTTCACCAGGGTGTCGCCCTCGAACTTCACCTCGGCGCGGGTCTTGTAGTTGCCGTCGTCCTTG<br>AAGAAGATGGTGCGCTCCTGGACGTAGCCTTCGGGCATGGCGGACTTGAAGAAGTCGTGCAGCTTCATGTGGTCGGGGTAGCGGGCGAAGCATTGCAGGCCGTAG<br>CCGAAGGTGGTCACGAGGGTGGGCCAGGGCACGGGCAGCTTGCCTGTGGTGCAGATGAACTTCAGGGTCAGCTTGCCGTAGGTGGCATCGCCCTCGCCCTCGCCG<br>GACACGCTGAACTTGTGGCCGTTTACGTCGCCGTCCAGCTCGACCAGGATGGGCACCACCCCGGTGAACAGCTCCTCGCCCTTGCTCACCATCTAGTATTTCTCC<br>TCTTTCTCTAGTATTAAACAAAATTATTTGTAGAGGCTGTTTCGTCCTCACGGACTCATCAGACCGGAAAGCACATCCGGTGACAGCTGAATCTATTATACAGAA<br>AAATTTTCCATAATACAAACAGACCAGATTGTCTGTTTGTTACTCCTACATCGGAACTCCTGGTCTTCCAGTACAATCTGCTCTGATGCCCTCGAGTGCGGCCGC<br>ACTAGTGTCGACGGAGCTCGAATTCGGATCCGAAGTTCCTATACTTTCTAGAGAATAGGAACTTCGGAATAGGAACTTCTATTTTTATAGGTTAATGTCATGATA<br>ATAATGGTTTCTTAGACCATGCCTATTTGTTTATTTTTCTAAATACATTCAAATATGTATCCGCTCATGAGACAATAACCCTGATAAATGCTTCAATAATATTGA<br>AAAAGGAAGAGTATGAGCCATATTCAACGGGAAACGTCTTGCTCTAGGCCGCGATTAAATTCCAACATGGATGCTGATTTATATGGGTATAAATGGGCTCGCGAT<br>AATGTCGGGCAATCAGGTGCGACAATCTATCGATTGTATGGGAAGCCCGATGCGCCAGAGTTGTTTCTGAAACATGGCAAAGGTAGCGTTGCCAATGATGTTACA<br>GATGAGATGGTCAGACTAAACTGGCTGACGGAATTTATGCCTCTTCCGACCATCAAGCATTTTATCCGTACTCCTGATGATGCATGGTTACTCACCACTGCGATC<br>CCCGGGAAAACAGCATTCCAGGTATTAGAAGAATATCCTGATTCAGGTGAAAATATTGTTGATGCGCTGGCAGTGTTCCTGCGCCGGTTGCATTCGATTCCTGTT<br>TGTAATTGTCCTTTTAACAGCGACCGCGTATTTCGTCTCGCTCAGGCGCAATCACGAATGAATAACGGTTTGGTTGATGCGAGTGATTTTGATGACGAGCGTAAT<br>GGCTGGCCTGTTGAACAAGTCTGGAAAGAAATGCATAAACTTTTGCCATTCTCACCGGATTCAGTCGTCACTCATGGTGATTTCTCACTTGATAACCTTATTTTT<br>GACGAGGGGAAATTAATAGGTTGTATTGATGTTGGACGAGTCGGAATCGCAGACCGATACCAGGATCTTGCCATCCTATGGAACTGCCTCGGTGAGTTTTCTCCT<br>TCATTACAGAAACGGCTTTTTCAAAAATATGGTATTGATAATCCTGATATGAATAAATTGCAGTTTCATTTGATGCTCGATGAGTTTTTCTAAAGCTCGCTTGGA

	CTCCTGTTGATAGATCCAGTAATGACCTCAGAACTCCATCTGGATTTGTTCAGAACGCTCGGTTGCCGCCGGGCGTTTTTTATTGGTGAGAATCCAAGCACAAGT<br>TCACGTACTAAGCTCTCATGTTTAACGTACTAAGCTCTCATGTTTAACGAACTAAACCCTCATGGCTAACGTACTAAGCTCTCATGGCTAACGTACTAAGCTCTC<br>ATGTTTCACGTACTAAGCTCTCATGTTTGAACAATAAAATTAATATAAATCAGCAACTTAAATAGCCTCTAAGGTTTTAAGTTTTATAAGAAAAAAAAGAATATA<br>TAAGGCTTTTAAAGCTTTTAAGGTTTAACGGTTGTGGACAACAAGCCAGGGATGTAACGCACTGAGAAGCCCTTAGAGCCTCTCAAAGCAATTTTGAGTGACACA<br>GGAACACTTAACGGCTGACATGGGAATTAGCCATGGGCCCGTGCGAATCATTTTTACGGTTCCTGGCCTTTTGCTGGCCTTTTGCTCACATGTTCTTTCCTGCGT<br>GGATAACCGTATTACCGCCTGGACCAAAACGAAAAAACACCCTTTCGGGTGTCTTTTCTGGAATTTGGTACCGAGCCTTTGGTCGAAAAAAAAAGCCCGCACTGT<br>CAGGTGCGGGCTTTTTTCTGTGTTTCCAGCCAAGAGCAGTGCCTTGCAGAAGTTCTCGGGCGAAGAGCTACTCCCCTTCTGCGCCGTCCGAAGTTCCTATACTTT<br>CTAGAGAATAGGAACTTCGGACCAAAACGAAAAAACACCCTTTCGGGTGTCTTTTCTGGAATTTGGTACCGAGCCTTTGGTCGAAAAAAAAAGCCCGCACTGTCA<br>GGTGCGGGCTTTTTTCTGTGTTTCCCGTTAACAGCGCAGAACTCAAGCAGGATGTTTGACGCGGGCGGCGTCAAG
YJP_MKC113_LP#1	AAAAAAAACCCCGCCCTGTCAGGGGCGGGGTTTTTTTTTTTTCTTTTGGGTATAGCGTCGTGGACAGTCATTCATCTTTCTGCCCCTCCAAAAGCAAAAACCCGC<br>CGAAGCGGGTTTTACGTAAATCAGGTGAAACTGACCGATAAGCCGGGACGAGCTGAGCAGTATGTCGACGGTCCGGCGAGAATCTCGCGTAGACACATACATGA<br>GCTTACTTGTACAGCTCGTCCATGCCGAGAGTGATCCCGGCGGCGGTCACGAACTCCAGCAGGACCATGTGATCGCGCTTCTCGTTGGGGTCTTTGCTCAGGGCG<br>GACTGGTAGCTAAGGTAGTGGTTGTCGGGCAGCAGCACGGGGCCGTCGCCGATTGGGGTGTTCTGCTGGTAGTGGTCGGCGAGCTGCACGCTGCCGTCCTCGATG<br>TTGTGGCGGATCTTGAAGTTCACCTTGATGCCGTTCTTCTGCTTGTCGGCCATGATATAGACGTTGTGGCTGTTGTAGTTGTACTCCAGCTTGTGCCCCAGGATG<br>TTGCCGTCCTCCTTGAAGTCGATGCCCTTCAGCTCGATGCGGTTCACCAGGGTGTCGCCCTCGAACTTCACCTCGGCGCGGGTCTTGTAGTTGCCGTCGTCCTTG<br>AAGAAGATGGTGCGCTCCTGGACGTAGCCTTCGGGCATGGCGGACTTGAAGAAGTCGTGCAGCTTCATGTGGTCGGGGTAGCGGGCGAAGCATTGCAGGCCGTAG<br>CCGAAGGTGGTCACGAGGGTGGGCCAGGGCACGGGCAGCTTGCCTGTGGTGCAGATGAACTTCAGGGTCAGCTTGCCGTAGGTGGCATCGCCCTCGCCCTCGCCG<br>GACACGCTGAACTTGTGGCCGTTTACGTCGCCGTCCAGCTCGACCAGGATGGGCACCACCCCGGTGAACAGCTCCTCGCCCTTGCTCACCATCTAGTATTTCTCC<br>TCTTTCTCTAGTATTAAACAAAATTATTTGTAGAGGCTGTTTCGTCCTCACGGACTCATCAGACCGGAAAGCACATCCGGTGACAGCTATTGGATCCAATGGTAC<br>CTAGGACTGAGCTAGCTGTCAATTGGATCCAATAAGGCAGGTAGGCTCCTCCTGGTCTTCCAGTACAATCTGCTCTGATGCCCTCGAGTGCGGCCGCACTAGTGT<br>CGACGGAGCTCGAATTCGGATCCGAAGTTCCTATACTTTCTAGAGAATAGGAACTTCGGAATAGGAACTTCTATTTTTATAGGTTAATGTCATGATAATAATGGT<br>TTCTTAGACCATGCCTATTTGTTTATTTTTCTAAATACATTCAAATATGTATCCGCTCATGAGACAATAACCCTGATAAATGCTTCAATAATATTGAAAAAGGAA<br>GAGTATGAGCCATATTCAACGGGAAACGTCTTGCTCTAGGCCGCGATTAAATTCCAACATGGATGCTGATTTATATGGGTATAAATGGGCTCGCGATAATGTCGG<br>GCAATCAGGTGCGACAATCTATCGATTGTATGGGAAGCCCGATGCGCCAGAGTTGTTTCTGAAACATGGCAAAGGTAGCGTTGCCAATGATGTTACAGATGAGAT<br>GGTCAGACTAAACTGGCTGACGGAATTTATGCCTCTTCCGACCATCAAGCATTTTATCCGTACTCCTGATGATGCATGGTTACTCACCACTGCGATCCCCGGGAA<br>AACAGCATTCCAGGTATTAGAAGAATATCCTGATTCAGGTGAAAATATTGTTGATGCGCTGGCAGTGTTCCTGCGCCGGTTGCATTCGATTCCTGTTTGTAATTG<br>TCCTTTTAACAGCGACCGCGTATTTCGTCTCGCTCAGGCGCAATCACGAATGAATAACGGTTTGGTTGATGCGAGTGATTTTGATGACGAGCGTAATGGCTGGCC<br>TGTTGAACAAGTCTGGAAAGAAATGCATAAACTTTTGCCATTCTCACCGGATTCAGTCGTCACTCATGGTGATTTCTCACTTGATAACCTTATTTTTGACGAGGG<br>GAAATTAATAGGTTGTATTGATGTTGGACGAGTCGGAATCGCAGACCGATACCAGGATCTTGCCATCCTATGGAACTGCCTCGGTGAGTTTTCTCCTTCATTACA<br>GAAACGGCTTTTTCAAAAATATGGTATTGATAATCCTGATATGAATAAATTGCAGTTTCATTTGATGCTCGATGAGTTTTTCTAAAGCTCGCTTGGACTCCTGTT<br>GATAGATCCAGTAATGACCTCAGAACTCCATCTGGATTTGTTCAGAACGCTCGGTTGCCGCCGGGCGTTTTTTATTGGTGAGAATCCAAGCACAAGTTCACGTAC<br>TAAGCTCTCATGTTTAACGTACTAAGCTCTCATGTTTAACGAACTAAACCCTCATGGCTAACGTACTAAGCTCTCATGGCTAACGTACTAAGCTCTCATGTTTCA<br>CGTACTAAGCTCTCATGTTTGAACAATAAAATTAATATAAATCAGCAACTTAAATAGCCTCTAAGGTTTTAAGTTTTATAAGAAAAAAAAGAATATATAAGGCTT<br>TTAAAGCTTTTAAGGTTTAACGGTTGTGGACAACAAGCCAGGGATGTAACGCACTGAGAAGCCCTTAGAGCCTCTCAAAGCAATTTTGAGTGACACAGGAACACT<br>TAACGGCTGACATGGGAATTAGCCATGGGCCCGTGCGAATCATTTTTACGGTTCCTGGCCTTTTGCTGGCCTTTTGCTCACATGTTCTTTCCTGCGTGGATAACC<br>GTATTACCGCCTGGACCAAAACGAAAAAACACCCTTTCGGGTGTCTTTTCTGGAATTTGGTACCGAGCCTTTGGTCGAAAAAAAAAGCCCGCACTGTCAGGTGCG<br>GGCTTTTTTCTGTGTTTCCAGCCAAGAGCAGTGCCTTGCAGAAGTTCTCGGGCGAAGAGCTACTCCCCTTCTGCGCCGTCCGAAGTTCCTATACTTTCTAGAGAA<br>TAGGAACTTCGGACCAAAACGAAAAAACACCCTTTCGGGTGTCTTTTCTGGAATTTGGTACCGAGCCTTTGGTCGAAAAAAAAAGCCCGCACTGTCAGGTGCGGG<br>CTTTTTTCTGTGTTTCCCGTTAACAGCGCAGAACTCAAGCAGGATGTTTGACGCGGGCGGCGTCAAG
YJP_MKC114_LP#1	AAAAAAAACCCCGCCCTGTCAGGGGCGGGGTTTTTTTTTTTTCTTTTGGGTATAGCGTCGTGGACAGTCATTCATCTTTCTGCCCCTCCAAAAGCAAAAACCCGC<br>CGAAGCGGGTTTTACGTAAATCAGGTGAAACTGACCGATAAGCCGGGACGAGCTGAGCAGTATGTCGACGGTCCGGCGAGAATCTCGCGTAGACACATACATGA<br>GCTTACTTGTACAGCTCGTCCATGCCGAGAGTGATCCCGGCGGCGGTCACGAACTCCAGCAGGACCATGTGATCGCGCTTCTCGTTGGGGTCTTTGCTCAGGGCG<br>GACTGGTAGCTAAGGTAGTGGTTGTCGGGCAGCAGCACGGGGCCGTCGCCGATTGGGGTGTTCTGCTGGTAGTGGTCGGCGAGCTGCACGCTGCCGTCCTCGATG<br>TTGTGGCGGATCTTGAAGTTCACCTTGATGCCGTTCTTCTGCTTGTCGGCCATGATATAGACGTTGTGGCTGTTGTAGTTGTACTCCAGCTTGTGCCCCAGGATG<br>TTGCCGTCCTCCTTGAAGTCGATGCCCTTCAGCTCGATGCGGTTCACCAGGGTGTCGCCCTCGAACTTCACCTCGGCGCGGGTCTTGTAGTTGCCGTCGTCCTTG<br>AAGAAGATGGTGCGCTCCTGGACGTAGCCTTCGGGCATGGCGGACTTGAAGAAGTCGTGCAGCTTCATGTGGTCGGGGTAGCGGGCGAAGCATTGCAGGCCGTAG<br>CCGAAGGTGGTCACGAGGGTGGGCCAGGGCACGGGCAGCTTGCCTGTGGTGCAGATGAACTTCAGGGTCAGCTTGCCGTAGGTGGCATCGCCCTCGCCCTCGCCG<br>GACACGCTGAACTTGTGGCCGTTTACGTCGCCGTCCAGCTCGACCAGGATGGGCACCACCCCGGTGAACAGCTCCTCGCCCTTGCTCACCATCTAGTATTTCTCC<br>TCTTTCTCTAGTATTAAACAAAATTATTTGTAGAGGCTGTTTCGTCCTCACGGACTCATCAGACCGGAAAGCACATCCGGTGACAGCTAGCGTTTTCAAGTTCGT<br>GGAAAGCAGAATGGTTCAGGCGAGCCGGATCGGAAATTCCCTCAGATAGGCCCCCCAGATGAGGGGATCAGAGCATGTCGCGCAAAAGTGTGCAGCGGTTTTG<br>CGATAACGACATGCGCCAAAACAAAGACTGAAAGCGCGAGGAGCGAATCTGAAAGATCGCGACCCGCTTTAGAGCGCCGTGCGTCCGTTTGGACGCACAAAGGGA<br>CTTTTGTGCTACCACTCCTGGTCTTCCAGTACAATCTGCTCTGATGCCCTCGAGTGCGGCCGCACTAGTGTCGACGGAGCTCGAATTCGGATCCGAAGTTCCTAT<br>ACTTTCTAGAGAATAGGAACTTCGGAATAGGAACTTCTATTTTTATAGGTTAATGTCATGATAATAATGGTTTCTTAGACCATGCCTATTTGTTTATTTTTCTAA<br>ATACATTCAAATATGTATCCGCTCATGAGACAATAACCCTGATAAATGCTTCAATAATATTGAAAAAGGAAGAGTATGAGCCATATTCAACGGGAAACGTCTTGC<br>TCTAGGCCGCGATTAAATTCCAACATGGATGCTGATTTATATGGGTATAAATGGGCTCGCGATAATGTCGGGCAATCAGGTGCGACAATCTATCGATTGTATGGG<br>AAGCCCGATGCGCCAGAGTTGTTTCTGAAACATGGCAAAGGTAGCGTTGCCAATGATGTTACAGATGAGATGGTCAGACTAAACTGGCTGACGGAATTTATGCCT<br>CTTCCGACCATCAAGCATTTTATCCGTACTCCTGATGATGCATGGTTACTCACCACTGCGATCCCCGGGAAAACAGCATTCCAGGTATTAGAAGAATATCCTGAT<br>TCAGGTGAAAATATTGTTGATGCGCTGGCAGTGTTCCTGCGCCGGTTGCATTCGATTCCTGTTTGTAATTGTCCTTTTAACAGCGACCGCGTATTTCGTCTCGCT<br>CAGGCGCAATCACGAATGAATAACGGTTTGGTTGATGCGAGTGATTTTGATGACGAGCGTAATGGCTGGCCTGTTGAACAAGTCTGGAAAGAAATGCATAAACTT<br>TTGCCATTCTCACCGGATTCAGTCGTCACTCATGGTGATTTCTCACTTGATAACCTTATTTTTGACGAGGGGAAATTAATAGGTTGTATTGATGTTGGACGAGTC<br>GGAATCGCAGACCGATACCAGGATCTTGCCATCCTATGGAACTGCCTCGGTGAGTTTTCTCCTTCATTACAGAAACGGCTTTTTCAAAAATATGGTATTGATAAT<br>CCTGATATGAATAAATTGCAGTTTCATTTGATGCTCGATGAGTTTTTCTAAAGCTCGCTTGGACTCCTGTTGATAGATCCAGTAATGACCTCAGAACTCCATCTG<br>GATTTGTTCAGAACGCTCGGTTGCCGCCGGGCGTTTTTTATTGGTGAGAATCCAAGCACAAGTTCACGTACTAAGCTCTCATGTTTAACGTACTAAGCTCTCATG<br>TTTAACGAACTAAACCCTCATGGCTAACGTACTAAGCTCTCATGGCTAACGTACTAAGCTCTCATGTTTCACGTACTAAGCTCTCATGTTTGAACAATAAAATTA<br>ATATAAATCAGCAACTTAAATAGCCTCTAAGGTTTTAAGTTTTATAAGAAAAAAAAGAATATATAAGGCTTTTAAAGCTTTTAAGGTTTAACGGTTGTGGACAAC<br>AAGCCAGGGATGTAACGCACTGAGAAGCCCTTAGAGCCTCTCAAAGCAATTTTGAGTGACACAGGAACACTTAACGGCTGACATGGGAATTAGCCATGGGCCCGT<br>GCGAATCATTTTTACGGTTCCTGGCCTTTTGCTGGCCTTTTGCTCACATGTTCTTTCCTGCGTGGATAACCGTATTACCGCCTGGACCAAAACGAAAAAACACCC<br>TTTCGGGTGTCTTTTCTGGAATTTGGTACCGAGCCTTTGGTCGAAAAAAAAAGCCCGCACTGTCAGGTGCGGGCTTTTTTCTGTGTTTCCAGCCAAGAGCAGTGC<br>CTTGCAGAAGTTCTCGGGCGAAGAGCTACTCCCCTTCTGCGCCGTCCGAAGTTCCTATACTTTCTAGAGAATAGGAACTTCGGACCAAAACGAAAAAACACCCTT<br>TCGGGTGTCTTTTCTGGAATTTGGTACCGAGCCTTTGGTCGAAAAAAAAAGCCCGCACTGTCAGGTGCGGGCTTTTTTCTGTGTTTCCCGTTAACAGCGCAGAAC<br>TCAAGCAGGATGTTTGACGCGGGCGGCGTCAAG
YJP_MKC115_LP#1	AAAAAAAACCCCGCCCTGTCAGGGGCGGGGTTTTTTTTTTTTCTTTTGGGTATAGCGTCGTGGACAGTCATTCATCTTTCTGCCCCTCCAAAAGCAAAAACCCGC<br>CGAAGCGGGTTTTACGTAAATCAGGTGAAACTGACCGATAAGCCGGGACGAGCTGAGCAGTATGTCGACGGTCCGGCGAGAATCTCGCGTAGACACATACATGA<br>GCTTACTTGTACAGCTCGTCCATGCCGAGAGTGATCCCGGCGGCGGTCACGAACTCCAGCAGGACCATGTGATCGCGCTTCTCGTTGGGGTCTTTGCTCAGGGCG<br>GACTGGTAGCTAAGGTAGTGGTTGTCGGGCAGCAGCACGGGGCCGTCGCCGATTGGGGTGTTCTGCTGGTAGTGGTCGGCGAGCTGCACGCTGCCGTCCTCGATG<br>TTGTGGCGGATCTTGAAGTTCACCTTGATGCCGTTCTTCTGCTTGTCGGCCATGATATAGACGTTGTGGCTGTTGTAGTTGTACTCCAGCTTGTGCCCCAGGATG<br>TTGCCGTCCTCCTTGAAGTCGATGCCCTTCAGCTCGATGCGGTTCACCAGGGTGTCGCCCTCGAACTTCACCTCGGCGCGGGTCTTGTAGTTGCCGTCGTCCTTG<br>AAGAAGATGGTGCGCTCCTGGACGTAGCCTTCGGGCATGGCGGACTTGAAGAAGTCGTGCAGCTTCATGTGGTCGGGGTAGCGGGCGAAGCATTGCAGGCCGTAG<br>CCGAAGGTGGTCACGAGGGTGGGCCAGGGCACGGGCAGCTTGCCTGTGGTGCAGATGAACTTCAGGGTCAGCTTGCCGTAGGTGGCATCGCCCTCGCCCTCGCCG<br>GACACGCTGAACTTGTGGCCGTTTACGTCGCCGTCCAGCTCGACCAGGATGGGCACCACCCCGGTGAACAGCTCCTCGCCCTTGCTCACCATCTAGTATTTCTCC<br>TCTTTCTCTAGTATTAAACAAAATTATTTGTAGAGGCTGTTTCGTCCTCACGGACTCATCAGACCGGAAAGCACATCCGGTGACAGCTTTGCTAAGGAATATACT<br>TACATTCATGGTTGTTTGTAAATACTGCTGGGTGACACGTGGCAGCGTACTCCTGGTCTTCCAGTACAATCTGCTCTGATGCCCTCGAGTGCGGCCGCACTAGTG<br>TCGACGGAGCTCGAATTCGGATCCGAAGTTCCTATACTTTCTAGAGAATAGGAACTTCGGAATAGGAACTTCTATTTdTTATAGGTTAATGTCATGATAATAATG<br>GTTTCTTAGACCATGCCTATTTGTTTATTTTTCTAAATACATTCAAATATGTATCCGCTCATGAGACAATAACCCTGATAAATGCTTCAATAATATTGAAAAAGG<br>AAGAGTATGAGCCATATTCAACGGGAAACGTCTTGCTCTAGGCCGCGATTAAATTCCAACATGGATGCTGATTTATATGGGTATAAATGGGCTCGCGATAATGTC<br>GGGCAATCAGGTGCGACAATCTATCGATTGTATGGGAAGCCCGATGCGCCAGAGTTGTTTCTGAAACATGGCAAAGGTAGCGTTGCCAATGATGTTACAGATGAG<br>ATGGTCAGACTAAACTGGCTGACGGAATTTATGCCTCTTCCGACCATCAAGCATTTTATCCGTACTCCTGATGATGCATGGTTACTCACCACTGCGATCCCCGGG<br>AAAACAGCATTCCAGGTATTAGAAGAATATCCTGATTCAGGTGAAAATATTGTTGATGCGCTGGCAGTGTTCCTGCGCCGGTTGCATTCGATTCCTGTTTGTAAT<br>TGTCCTTTTAACAGCGACCGCGTATTTCGTCTCGCTCAGGCGCAATCACGAATGAATAACGGTTTGGTTGATGCGAGTGATTTTGATGACGAGCGTAATGGCTGG<br>CCTGTTGAACAAGTCTGGAAAGAAATGCATAAACTTTTGCCATTCTCACCGGATTCAGTCGTCACTCATGGTGATTTCTCACTTGATAACCTTATTTTTGACGAG<br>GGGAAATTAATAGGTTGTATTGATGTTGGACGAGTCGGAATCGCAGACCGATACCAGGATCTTGCCATCCTATGGAACTGCCTCGGTGAGTTTTCTCCTTCATTA<br>CAGAAACGGCTTTTTCAAAAATATGGTATTGATAATCCTGATATGAATAAATTGCAGTTTCATTTGATGCTCGATGAGTTTTTCTAAAGCTCGCTTGGACTCCTG<br>TTGATAGATCCAGTAATGACCTCAGAACTCCATCTGGATTTGTTCAGAACGCTCGGTTGCCGCCGGGCGTTTTTTATTGGTGAGAATCCAAGCACAAGTTCACGT<br>ACTAAGCTCTCATGTTTAACGTACTAAGCTCTCATGTTTAACGAACTAAACCCTCATGGCTAACGTACTAAGCTCTCATGGCTAACGTACTAAGCTCTCATGTTT

	CACGTACTAAGCTCTCATGTTTGAACAATAAAATTAATATAAATCAGCAACTTAAATAGCCTCTAAGGTTTTAAGTTTTATAAGAAAAAAAAGAATATATAAGGC<br>TTTTAAAGCTTTTAAGGTTTAACGGTTGTGGACAACAAGCCAGGGATGTAACGCACTGAGAAGCCCTTAGAGCCTCTCAAAGCAATTTTGAGTGACACAGGAACA<br>CTTAACGGCTGACATGGGAATTAGCCATGGGCCCGTGCGAATCATTTTTACGGTTCCTGGCCTTTTGCTGGCCTTTTGCTCACATGTTCTTTCCTGCGTGGATAA<br>CCGTATTACCGCCTGGACCAAAACGAAAAAACACCCTTTCGGGTGTCTTTTCTGGAATTTGGTACCGAGCCTTTGGTCGAAAAAAAAAGCCCGCACTGTCAGGTG<br>CGGGCTTTTTTCTGTGTTTCCAGCCAAGAGCAGTGCCTTGCAGAAGTTCTCGGGCGAAGAGCTACTCCCCTTCTGCGCCGTCCGAAGTTCCTATACTTTCTAGAG<br>AATAGGAACTTCGGACCAAAACGAAAAAACACCCTTTCGGGTGTCTTTTCTGGAATTTGGTACCGAGCCTTTGGTCGAAAAAAAAAGCCCGCACTGTCAGGTGCG<br>GGCTTTTTTCTGTGTTTCCCGTTAACAGCGCAGAACTCAAGCAGGATGTTTGACGCGGGCGGCGTCAAG
YJP_MKC254_LP#1	AAAAAAAACCCCGCCCTGTCAGGGGCGGGGTTTTTTTTTTTTCTTTTGGGTATAGCGTCGTGGACAGTCATTCATCTTTCTGCCCCTCCAAAAGCAAAAACCCGC<br>CGAAGCGGGTTTTACGTAAATCAGGTGAAACTGACCGATAAGCCGGGACGAGCTGAGCAGTATGTCGACGGTCCGGCGAGAATCTCGCGTAGACACATACATGA<br>GCTTACTTGTACAGCTCGTCCATGCCGAGAGTGATCCCGGCGGCGGTCACGAACTCCAGCAGGACCATGTGATCGCGCTTCTCGTTGGGGTCTTTGCTCAGGGCG<br>GACTGGTAGCTAAGGTAGTGGTTGTCGGGCAGCAGCACGGGGCCGTCGCCGATTGGGGTGTTCTGCTGGTAGTGGTCGGCGAGCTGCACGCTGCCGTCCTCGATG<br>TTGTGGCGGATCTTGAAGTTCACCTTGATGCCGTTCTTCTGCTTGTCGGCCATGATATAGACGTTGTGGCTGTTGTAGTTGTACTCCAGCTTGTGCCCCAGGATG<br>TTGCCGTCCTCCTTGAAGTCGATGCCCTTCAGCTCGATGCGGTTCACCAGGGTGTCGCCCTCGAACTTCACCTCGGCGCGGGTCTTGTAGTTGCCGTCGTCCTTG<br>AAGAAGATGGTGCGCTCCTGGACGTAGCCTTCGGGCATGGCGGACTTGAAGAAGTCGTGCAGCTTCATGTGGTCGGGGTAGCGGGCGAAGCATTGCAGGCCGTAG<br>CCGAAGGTGGTCACGAGGGTGGGCCAGGGCACGGGCAGCTTGCCTGTGGTGCAGATGAACTTCAGGGTCAGCTTGCCGTAGGTGGCATCGCCCTCGCCCTCGCCG<br>GACACGCTGAACTTGTGGCCGTTTACGTCGCCGTCCAGCTCGACCAGGATGGGCACCACCCCGGTGAACAGCTCCTCGCCCTTGCTCACCATCTAGTATTTCTCC<br>TCTTTCTCTAGTATTAAACAAAATTATTTGTAGAGGCTGTTTCGTCCTCACGGACTCATCAGACCGGAAAGCACATCCGGTGACAGCTTCAGGCTAGCATAATAC<br>CTAGGACTGAGCTAGCTGTAAAAGTTAGGGACTTATCAAGCTTCCACCGTACGTCGAGTATGGTCTTCCAGTACAATCTGCTCTGATGCCCTCGAGTGCGGCCGC<br>ACTAGTGTCGACGGAGCTCGAATTCGGATCCGAAGTTCCTATACTTTCTAGAGAATAGGAACTTCGGACCAAAACGAAAAAACACCCTTTCGGGTGTCTTTTCTG<br>GAATTTGGTACCGAGCCTTTGGTCGAAAAAAAAAGCCCGCACTGTCAGGTGCGGGCTTTTTTCTGTGTTTCCCGTTAACAGCGCAGAACTCAAGCAGGATGTTTG<br>ACGCGGGCGGCGTCAAG
YJP_MKC255_LP#1	AAAAAAAACCCCGCCCTGTCAGGGGCGGGGTTTTTTTTTTTTCTTTTGGGTATAGCGTCGTGGACAGTCATTCATCTTTCTGCCCCTCCAAAAGCAAAAACCCGC<br>CGAAGCGGGTTTTACGTAAATCAGGTGAAACTGACCGATAAGCCGGGACGAGCTGAGCAGTATGTCGACGGTCCGGCGAGAATCTCGCGTAGACACATACATGA<br>GCTTACTTGTACAGCTCGTCCATGCCGAGAGTGATCCCGGCGGCGGTCACGAACTCCAGCAGGACCATGTGATCGCGCTTCTCGTTGGGGTCTTTGCTCAGGGCG<br>GACTGGTAGCTAAGGTAGTGGTTGTCGGGCAGCAGCACGGGGCCGTCGCCGATTGGGGTGTTCTGCTGGTAGTGGTCGGCGAGCTGCACGCTGCCGTCCTCGATG<br>TTGTGGCGGATCTTGAAGTTCACCTTGATGCCGTTCTTCTGCTTGTCGGCCATGATATAGACGTTGTGGCTGTTGTAGTTGTACTCCAGCTTGTGCCCCAGGATG<br>TTGCCGTCCTCCTTGAAGTCGATGCCCTTCAGCTCGATGCGGTTCACCAGGGTGTCGCCCTCGAACTTCACCTCGGCGCGGGTCTTGTAGTTGCCGTCGTCCTTG<br>AAGAAGATGGTGCGCTCCTGGACGTAGCCTTCGGGCATGGCGGACTTGAAGAAGTCGTGCAGCTTCATGTGGTCGGGGTAGCGGGCGAAGCATTGCAGGCCGTAG<br>CCGAAGGTGGTCACGAGGGTGGGCCAGGGCACGGGCAGCTTGCCTGTGGTGCAGATGAACTTCAGGGTCAGCTTGCCGTAGGTGGCATCGCCCTCGCCCTCGCCG<br>GACACGCTGAACTTGTGGCCGTTTACGTCGCCGTCCAGCTCGACCAGGATGGGCACCACCCCGGTGAACAGCTCCTCGCCCTTGCTCACCATCTAGTATTTCCCC<br>TCTTTCTCTAGTATTAAACAAAATTATTTGTAGAGGCTGTTTCGTCCTCACGGACTCATCAGACCGGAAAGCACATCCGGTGACAGCTTCAGGCTAGCATAATAC<br>CTAGGACTGAGCTAGCTGTAAAAGTTAGGGACTTATCAAGCTTCCACCGTACGTCGAGTATGGTCTTCCAGTACAATCTGCTCTGATGCCCTCGAGTGCGGCCGC<br>ACTAGTGTCGACGGAGCTCGAATTCGGATCCGAAGTTCCTATACTTTCTAGAGAATAGGAACTTCGGACCAAAACGAAAAAACACCCTTTCGGGTGTCTTTTCTG<br>GAATTTGGTACCGAGCCTTTGGTCGAAAAAAAAAGCCCGCACTGTCAGGTGCGGGCTTTTTTCTGTGTTTCCCGTTAACAGCGCAGAACTCAAGCAGGATGTTTG<br>ACGCGGGCGGCGTCAAG
YJP_MKC140_LP#1	AAAAAAAACCCCGCCCTGTCAGGGGCGGGGTTTTTTTTTTTTCTTTTGGGTATAGCGTCGTGGACAGTCATTCATCTTTCTGCCCCTCCAAAAGCAAAAACCCGC<br>CGAAGCGGGTTTTACGTAAATCAGGTGAAACTGACCGATAAGCCGGGACGAGCTGAGCAGTATGTCGACGGTCCGGCGAGAATCTCGCGTAGACACATACATGA<br>GCAGTAGGACCAAAACGAAAAAGGCCCCCCTTTCGGGAGGCCTCTTTTCTGGAATTTGGTACCGAGTGCAGACGTAAAAAAAGCGGCGTGGTTAGCCGCTTTTT<br>TAATTGCCGGATTAACGCTGTGTACCCGGACAAACACCATTAATCAGCAGGAAGGTAAATTCTTCAATATCCTGTTCAACGGTCAGCTGTTCGGTCAGCAGGCGA<br>TACCAACAAAAACCAAAAATCATATCCAGCAGCAGTTCACGATTGGTATCTTTCGGCAGTTCACCATTGCTAATGGCATTTTCAACCAGTTTTTCGGCATCTCA<br>CGACGACGTTCCATAAACTGATCTTTCAGCTGGGTCAGGGTTGCAGGGTCCAGCTGTGCTTCTGCAATAACACAACGAAATGCTTCACCACAAATGGTTTCACGC<br>CAAACTTTCCACAGATTACGCAGCAGAAAATCCAGATCGGCTTTAAAGCTACCCAGATCCGGAAATTTACGCACCTGTTCGCTTTCATTTTCATACACTTCGGCA<br>ATCAGTGCTGCTTTATTGGTCCACCAACGATAAATGGTCGGTTTGCTTGCACCGGCACGACGTGCAACGCTTTCAATGCTCAGACCGCTATAACCACATTCTTTC<br>AGGATTTCAATGGTGCTGGTCAGAATTGCTTTATGGGTATGCGGACTACGCAGGCTACCAATGCTGCTACGGCTCGGGGTACGTGCCATAGGATCGCCCTCGTAA<br>AGTTAAACAAAATTATTTGTAGAGGCGGTTTCGACATCACTGTCTCATCAGAACGCAAAGCACATGCGTTGACCGCTAATTGTGAGCGCTCACAATTCCACACAT<br>TATACGAGCCGATGATTAATTGTCAACACAGCCAACGATCGTTCTCCTGGTCTTCCAGTACAATCTGCTCTGATGCCCTCGAGTGCGGCCGCACTAGTGTCGACG<br>GAGCTCGAATTCGGATCCGAAGTTCCTATACTTTCTAGAGAATAGGAACTTCGGAATAGGAACTTCTATTTTTATAGGTTAATGTCATGATAATAATGGTTTCTT<br>AGACCATGCCTATTTGTTTATTTTTCTAAATACATTCAAATATGTATCCGCTCATGAGACAATAACCCTGATAAATGCTTCAATAATATTGAAAAAGGAAGAGTA<br>TGAGCCATATTCAACGGGAAACGTCTTGCTCTAGGCCGCGATTAAATTCCAACATGGATGCTGATTTATATGGGTATAAATGGGCTCGCGATAATGTCGGGCAAT<br>CAGGTGCGACAATCTATCGATTGTATGGGAAGCCCGATGCGCCAGAGTTGTTTCTGAAACATGGCAAAGGTAGCGTTGCCAATGATGTTACAGATGAGATGGTCA<br>GACTAAACTGGCTGACGGAATTTATGCCTCTTCCGACCATCAAGCATTTTATCCGTACTCCTGATGATGCATGGTTACTCACCACTGCGATCCCCGGGAAAACAG<br>CATTCCAGGTATTAGAAGAATATCCTGATTCAGGTGAAAATATTGTTGATGCGCTGGCAGTGTTCCTGCGCCGGTTGCATTCGATTCCTGTTTGTAATTGTCCTT<br>TTAACAGCGACCGCGTATTTCGTCTCGCTCAGGCGCAATCACGAATGAATAACGGTTTGGTTGATGCGAGTGATTTTGATGACGAGCGTAATGGCTGGCCTGTTG<br>AACAAGTCTGGAAAGAAATGCATAAACTTTTGCCATTCTCACCGGATTCAGTCGTCACTCATGGTGATTTCTCACTTGATAACCTTATTTTTGACGAGGGGAAAT<br>TAATAGGTTGTATTGATGTTGGACGAGTCGGAATCGCAGACCGATACCAGGATCTTGCCATCCTATGGAACTGCCTCGGTGAGTTTTCTCCTTCATTACAGAAAC<br>GGCTTTTTCAAAAATATGGTATTGATAATCCTGATATGAATAAATTGCAGTTTCATTTGATGCTCGATGAGTTTTTCTAAAGCTCGCTTGGACTCCTGTTGATAG<br>ATCCAGTAATGACCTCAGAACTCCATCTGGATTTGTTCAGAACGCTCGGTTGCCGCCGGGCGTTTTTTATTGGTGAGAATCCAAGCACAAGAAGATCCGGCCACG<br>ATGCGTCCGGCGTAGAGGATCTGAAGATCAGCAGTTCAACCTGTTGATAGTACGTACTAAGCTCTCATGTTTCACGTACTAAGCTCTCATGTTTAACGTACTAAG<br>CTCTCATGTTTAACGAACTAAACCCTCATGGCTAACGTACTAAGCTCTCATGGCTAACGTACTAAGCTCTCATGTTTCACGTACTAAGCTCTCATGTTTGAACAA<br>TAAAATTAATATAAATCAGCAACTTAAATAGCCTCTAAGGTTTTAAGTTTTATAAGAAAAAAAAGAATATATAAGGCTTTTAAAGCTTTTAAGGTTTAACGGTTG<br>TGGACAACAAGCCAGGGATGTAACGCACTGAGAAGCCCTTAGAGCCTCTCAAAGCAATTTTGAGTGACACAGGAACACTTAACGGCTGACATGGGAATTAGCCAT<br>GGGCCCGTGCGAATCATTTTTACGGTTCCTGGCCTTTTGCTGGCCTTTTGCTCACATGTTCTTTCCTGCGTGGATAACCGTATTACCGCCTGGACCAAAACGAAA<br>AAACACCCTTTCGGGTGTCTTTTCTGGAATTTGGTACCGAGCCTTTGGTCGAAAAAAAAAGCCCGCACTGTCAGGTGCGGGCTTTTTTCTGTGTTTCCAGCCAAG<br>AGCAGTGCCTTGCAGAAGTTCTCGGGCGAAGAGCTACTCCCCTTCTGCGCCGTCCGAAGTTCCTATACTTTCTAGAGAATAGGAACTTCGGACCAAAACGAAAAA<br>ACACCCTTTCGGGTGTCTTTTCTGGAATTTGGTACCGAGCCTTTGGTCGAAAAAAAAAGCCCGCACTGTCAGGTGCGGGCTTTTTTCTGTGTTTCCCGTTAACAG<br>CGCAGAACTCAAGCAGGATGTTTGACGCGGGCGGCGTCAAG
YJP_MKC141_LP#1	AAAAAAAACCCCGCCCTGTCAGGGGCGGGGTTTTTTTTTTTTCTTTTGGGTATAGCGTCGTGGACAGTCATTCATCTTTCTGCCCCTCCAAAAGCAAAAACCCGC<br>CGAAGCGGGTTTTACGTAAATCAGGTGAAACTGACCGATAAGCCGGGACGAGCTGAGCAGTATGTCGACGGTCCGGCGAGAATCTCGCGTAGACACATACATGA<br>GCAAGCGTCAGAGGAGCCAAATTCAAAAAAGCCTGCTTTCTAGCAGGCTTTTTGCTTTCTAATGGAAGCATAAAAAAATGGCGCCGATGGGCGCCATTTTTCACT<br>GCGGCAAGAATTACTTCAGACAGAGTATCAAAAGGCGAAACCTCCGCAATGCGGAGGTTTCTTTTTAAAGATTATTTGCTCAGGCCATTCAGAAAGATCTGGCTG<br>AATTTGTTCATCAGTTTAATGCGTTCGTTGATATTCTGCTCATGGGTAAAGGTAACAATGCCATTAACGGCATTTGCTGCAATTTTGCTCACGGCATTAACATCA<br>TTAATGCACCATTCGCCGTTCAGATTACCTTCTTTAAAGATCACGTGGTAGGCATCGATGTATTTGTTTTCCAGTTTGTTCATTTTCTCGTTGATGCTGTTGGTT<br>TTATAGTACTCGGTATAAAACTCGATGATGGCATTCTGCAGCGGATAGTAATATTCGGTGGTCAGGCTCAGTTCATTATACAGATAGAATTTCTCGCGGTTGGTT<br>TTGCATTTGATTTGTTCTTTTTCCACTGCTCTTGCCATTTGCTTTCTTCGATGTTCAGGATTTCCAGAAACAGGTTCTCTTTGGTTTTAAAGTGATAATACAGA<br>TTGCCTTTGCTGCTTTCGCTCAGTTTAACAATTTCACCGGTGGTGGTTGCGTTATAGCCATTTTTGATAAACAGTTCTTTGGCAACGCCCAGAATTTTATCTTTC<br>AGGTTCATCTAGTAGTTGTCCTCTATCAAAAGCCAATGGCATGGCTTAAACAAAATTATTTGTAGAGGTCGTTTCGGCATCACTGCCTCATCAGAAGTGCAAGCA<br>CAGCACTTGACGACTAATTGTGAGCGCTCACAATTCCACACATTATACGAGCCGATGATTAATTGTCAACACAGCCAACGATCGTTACCTTGGTCTTCCAGTACA<br>ATCTGCTCTGATGCCCTCGAGTGCGGCCGCACTAGTGTCGACGGAGCTCGAATTCGGATCCGAAGTTCCTATACTTTCTAGAGAATAGGAACTTCGGAATAGGAA<br>CTTCTATTTTTATAGGTTAATGTCATGATAATAATGGTTTCTTAGACCATGCCTATTTGTTTATTTTTCTAAATACATTCAAATATGTATCCGCTCATGAGACAA<br>TAACCCTGATAAATGCTTCAATAATATTGAAAAAGGAAGAGTATGAGCCATATTCAACGGGAAACGTCTTGCTCTAGGCCGCGATTAAATTCCAACATGGATGCT<br>GATTTATATGGGTATAAATGGGCTCGCGATAATGTCGGGCAATCAGGTGCGACAATCTATCGATTGTATGGGAAGCCCGATGCGCCAGAGTTGTTTCTGAAACAT<br>GGCAAAGGTAGCGTTGCCAATGATGTTACAGATGAGATGGTCAGACTAAACTGGCTGACGGAATTTATGCCTCTTCCGACCATCAAGCATTTTATCCGTACTCCT<br>GATGATGCATGGTTACTCACCACTGCGATCCCCGGGAAAACAGCATTCCAGGTATTAGAAGAATATCCTGATTCAGGTGAAAATATTGTTGATGCGCTGGCAGTG<br>TTCCTGCGCCGGTTGCATTCGATTCCTGTTTGTAATTGTCCTTTTAACAGCGACCGCGTATTTCGTCTCGCTCAGGCGCAATCACGAATGAATAACGGTTTGGTT<br>GATGCGAGTGATTTTGATGACGAGCGTAATGGCTGGCCTGTTGAACAAGTCTGGAAAGAAATGCATAAACTTTTGCCATTCTCACCGGATTCAGTCGTCACTCAT<br>GGTGATTTCTCACTTGATAACCTTATTTTTGACGAGGGGAAATTAATAGGTTGTATTGATGTTGGACGAGTCGGAATCGCAGACCGATACCAGGATCTTGCCATC<br>CTATGGAACTGCCTCGGTGAGTTTTCTCCTTCATTACAGAAACGGCTTTTTCAAAAATATGGTATTGATAATCCTGATATGAATAAATTGCAGTTTCATTTGATG<br>CTCGATGAGTTTTTCTAAAGCTCGCTTGGACTCCTGTTGATAGATCCAGTAATGACCTCAGAACTCCATCTGGATTTGTTCAGAACGCTCGGTTGCCGCCGGGCG<br>TTTTTTATTGGTGAGAATCCAAGCACAAGAAGATCCGGCCACGATGCGTCCGGCGTAGAGGATCTGAAGATCAGCAGTTCAACCTGTTGATAGTACGTACTAAGC<br>TCTCATGTTTCACGTACTAAGCTCTCATGTTTAACGTACTAAGCTCTCATGTTTAACGAACTAAACCCTCATGGCTAACGTACTAAGCTCTCATGGCTAACGTAC<br>TAAGCTCTCATGTTTCACGTACTAAGCTCTCATGTTTGAACAATAAAATTAATATAAATCAGCAACTTAAATAGCCTCTAAGGTTTTAAGTTTTATAAGAAAAAA<br>AAGAATATATAAGGCTTTTAAAGCTTTTAAGGTTTAACGGTTGTGGACAACAAGCCAGGGATGTAACGCACTGAGAAGCCCTTAGAGCCTCTCAAAGCAATTTTG<br>AGTGACACAGGAACACTTAACGGCTGACATGGGAATTAGCCATGGGCCCGTGCGAATCATTTTTACGGTTCCTGGCCTTTTGCTGGCCTTTTGCTCACATGTTCT<br>TTCCTGCGTGGATAACCGTATTACCGCCTGGACCAAAACGAAAAAACACCCTTTCGGGTGTCTTTTCTGGAATTTGGTACCGAGCCTTTGGTCGAAAAAAAAAGC

	CCGCACTGTCAGGTGCGGGCTTTTTCTGTGTTTCCAGCCAAGAGCAGTGCCTTGCAGAAGTTCTCGGGCGAAGAGCTACTCCCCTTCTGCGCCGTCCGAAGTTC<br>CTATACTTTCTAGAGAATAGGAACTTCGGACCAAAACGAAAAAACACCCTTTCGGGTGTCTTTTCTGGAATTTGGTACCGAGCCTTTGGTCGAAAAAAAAAGCCC<br>GCACTGTCAGGTGCGGGCTTTTTCTGTGTTTCCCGTTAACAGCGCAGAACTCAAGCAGGATGTTTGACGCGGGCGGCGTCAAG
YJP_MKC142_LP#1	AAAAAAAACCCCGCCCTGTCAGGGGCGGGGTTTTTTTTTTTCTTTTGGGTATAGCGTCGTGGACAGTCATTCATCTTTCTGCCCCTCCAAAAGCAAAAACCCGC<br>CGAAGCGGGTTTTTACGTAAATCAGGTGAAACTGACCGATAAGCCGGGACGAGCTGAGCAGTATGTCGACGGTCCGGCGAGAATCTCGCGTAGACACATACATGA<br>GCAGCGGCGGAGAAAAGGTCCGAAAATTCGGACCCGATGGAATTGGATAAAAAAAGGCCCCTGTTGAAATTGCAGGGGCCTGGTACGAGCATTATTTTGCATCTG<br>CCTGTTTAATCAGTTCCAGGGTTTTTCAACACGATCTGCAGGCAGCGGTGCACCCAGAACTGCCAGGCTTGCATCTGCCAGCATAATTGCGGTTTCCGGCAGGC<br>TATCTGCGCTCAGCGGACTCGGAATTTTACCATCATTGCGACGCATTTCAATAACGCTCATGGTAATATGAAACGGCAGTTCTGCACGCGGATCATCACCAACAA<br>TTTCGGTTGCCAGATCACGAAAAACATTGGTCAGTGCTTCACGCTGGCTATGATATTCTGCAAATTCTTCGCTACCAACAATCGGCAGCTGATACAGACGACCAA<br>CATTCCATTTGGTGCTCAGCAGCAGACGAACTTCGCTTGCAACAATTGCCCACAGACGCATTTCCGGACCTGCATCCAGGGTGCTCAGATCTTCTGCCAGAACGG<br>TGCTCGGTTCAACGGTGCTTTTCAGCAGGGTCAGAAAGATTTCGGTTTTGCTCGGAAAATGATAATACAGGCTTGCCTGACGAATACCAACTGCATCTGCAATCT<br>GATGGGTACTGGTGGTTGCAAAACCCTGACGGGTAAACAGTTCTGCGCTTGCATCCAGAATTTCTTCACGCGGATTTTTACCTGCACGACGCGGTGCACTACGAC<br>GCGGACGACCAACTGCGCCTGCCATCTAGTAACCTCTTTGCTGATTATTAGGGAACATTTTAAACAAAATTATTTGTAGAGGGGTGTTTCGTCCTTTCGGACTCA<br>TCAGTCAAGGTACGCACCTTGAGACACCCTAATTGTGAGCGCTCACAATTCCACACATTATACGAGCCGATGATTAATTGTCAACACAGCCAACGATCGTTGGCA<br>TGGTCTTCCAGTACAATCTGCTCTGATGCCCTCGAGTGCGGCCGCACTAGTGTCGACGGAGCTCGAATTCGGATCCGAAGTTCCTATACTTTCTAGAGAATAGGA<br>ACTTCGGAATAGGAACTTCTATTTTTATAGGTTAATGTCATGATAATAATGGTTTCTTAGACCATGCCTATTTGTTTATTTTTCTAAATACATTCAAATATGTAT<br>CCGCTCATGAGACAATAACCCTGATAAATGCTTCAATAATATTGAAAAAGGAAGAGTATGAGCCATATTCAACGGGAAACGTCTTGCTCTAGGCCGCGATTAAAT<br>TCCAACATGGATGCTGATTTATATGGGTATAAATGGGCTCGCGATAATGTCGGGCAATCAGGTGCGACAATCTATCGATTGTATGGGAAGCCCGATGCGCCAGAG<br>TTGTTTCTGAAACATGGCAAAGGTAGCGTTGCCAATGATGTTACAGATGAGATGGTCAGACTAAACTGGCTGACGGAATTTATGCCTCTTCCGACCATCAAGCAT<br>TTTATCCGTACTCCTGATGATGCATGGTTACTCACCACTGCGATCCCCGGGAAAACAGCATTCCAGGTATTAGAAGAATATCCTGATTCAGGTGAAAATATTGTT<br>GATGCGCTGGCAGTGTTCCTGCGCCGGTTGCATTCGATTCCTGTTTGTAATTGTCCTTTTAACAGCGACCGCGTATTTCGTCTCGCTCAGGCGCAATCACGAATG<br>AATAACGGTTTGGTTGATGCGAGTGATTTTGATGACGAGCGTAATGGCTGGCCTGTTGAACAAGTCTGGAAAGAAATGCATAAACTTTTGCCATTCTCACCGGAT<br>TCAGTCGTCACTCATGGTGATTTCTCACTTGATAACCTTATTTTTGACGAGGGGAAATTAATAGGTTGTATTGATGTTGGACGAGTCGGAATCGCAGACCGATAC<br>CAGGATCTTGCCATCCTATGGAACTGCCTCGGTGAGTTTTCTCCTTCATTACAGAAACGGCTTTTTCAAAAATATGGTATTGATAATCCTGATATGAATAAATTG<br>CAGTTTCATTTGATGCTCGATGAGTTTTTCTAAAGCTCGCTTGGACTCCTGTTGATAGATCCAGTAATGACCTCAGAACTCCATCTGGATTTGTTCAGAACGCTC<br>GGTTGCCGCCGGGCGTTTTTTATTGGTGAGAATCCAAGCACAAGAAGATCCGGCCACGATGCGTCCGGCGTAGAGGATCTGAAGATCAGCAGTTCAACCTGTTGA<br>TAGTACGTACTAAGCTCTCATGTTTCACGTACTAAGCTCTCATGTTTAACGTACTAAGCTCTCATGTTTAACGAACTAAACCCTCATGGCTAACGTACTAAGCTC<br>TCATGGCTAACGTACTAAGCTCTCATGTTTCACGTACTAAGCTCTCATGTTTGAACAATAAAATTAATATAAATCAGCAACTTAAATAGCCTCTAAGGTTTTAAG<br>TTTTATAAGAAAAAAAAGAATATATAAGGCTTTTAAAGCTTTTAAGGTTTAACGGTTGTGGACAACAAGCCAGGGATGTAACGCACTGAGAAGCCCTTAGAGCCT<br>CTCAAAGCAATTTTGAGTGACACAGGAACACTTAACGGCTGACATGGGAATTAGCCATGGGCCCGTGCGAATCATTTTTACGGTTCCTGGCCTTTTGCTGGCCTT<br>TTGCTCACATGTTCTTTCCTGCGTGGATAACCGTATTACCGCCTGGACCAAAACGAAAAAACACCCTTTCGGGTGTCTTTTCTGGAATTTGGTACCGAGCCTTTG<br>GTCGAAAAAAAAAGCCCGCACTGTCAGGTGCGGGCTTTTTCTGTGTTTCCAGCCAAGAGCAGTGCCTTGCAGAAGTTCTCGGGCGAAGAGCTACTCCCCTTCTG<br>CGCCGTCCGAAGTTCCTATACTTTCTAGAGAATAGGAACTTCGGACCAAAACGAAAAAACACCCTTTCGGGTGTCTTTTCTGGAATTTGGTACCGAGCCTTTGGT<br>CGAAAAAAAAAGCCCGCACTGTCAGGTGCGGGCTTTTTCTGTGTTTCCCGTTAACAGCGCAGAACTCAAGCAGGATGTTTGACGCGGGCGGCGTCAAG
YJP_MKC146_LP#1	AAAAAAAACCCCGCCCTGTCAGGGGCGGGGTTTTTTTTTTTCTTTTGGGTATAGCGTCGTGGACAGTCATTCATCTTTCTGCCCCTCCAAAAGCAAAAACCCGC<br>CGAAGCGGGTTTTTACGTAAATCAGGTGAAACTGACCGATAAGCCGGGACGAGCTGAGCAGTATGTCGACGGTCCGGCGAGAATCTCGCGTAGACACATACATGA<br>GCAGCGCATAAAAAAAATGGCGCCGATGGGCGCCATTTTTCACTGTGTCATACGTAAAAAAGGGCGATCTTGCGACCGCCCTTTTTTTATTAAATGTTTATTTTGC<br>CAGCGGATTACGCAGACCTGCATCAATCAGATCAACCAGCTGATCACAACGGGTTGCCAGATTACCAATATCTTCTTGTGCAATCAGAACCGGATGAATAACGCT<br>GGTCAGTGCATGCAGAACGGTTTCTGCCAGAACCGGAATATCGGTTGCAATATACAGACCTGCTTCAACACCATCACGAATAATTTCGGCCAGCAGTTTTGCAAT<br>CACGCTTTTGTAATAATCGCCACATTTCATATCCTGTTTGGCGGTCATCAGGATCTGAATAAAAACCCGTATGTGTTTGAAATGATCCTGATGATGCTGTTCCAT<br>CAGATTACGTGCCAGATGACGCAGACGATCCAGCGGTGCATGGCTTTTATCCAGCGGACAAATCTGACGTTCAAAAACACCAATCTGACCAAAACCAATTGCATC<br>AACCAGTGCGTTTTTGCTGCTAAAATGTTTAAACACATTTGCCGGACTCATATTCAGTGCGCTTGCAATATCTGCAATGGCAACTGCATTAAAACCACGTTCACG<br>AAACAGTTCTTCGGCTTTTGCCAGAATATCACGACGGGTTTCTTCGGCACTACGACGCGGACGACGACGAACCGGCAGATCGCTTTTACGATCACCTTTACGCAC<br>CTGATCAATGGTTTTGTTCATCTATTAGCTCCTGTTTGAAACATAGTCCATAGCGTATTAAACAAAATTATTTGTAGAGGGGGTTTCGTCATCACTGACTCATCA<br>GAGTTGAAAGCACATCAACTGACCCCTAATTGTGAGCGCTCACAATTCCACACATTATACGAGCCGATGATTAATTGTCAACACAGCCAACGATCGTTCTCCTGG<br>TCTTCCAGTACAATCTGCTCTGATGCCCTCGAGTGCGGCCGCACTAGTGTCGACGGAGCTCGAATTCGGATCCGAAGTTCCTATACTTTCTAGAGAATAGGAACT<br>TCGGAATAGGAACTTCTATTTTTATAGGTTAATGTCATGATAATAATGGTTTCTTAGACCATGCCTATTTGTTTATTTTTCTAAATACATTCAAATATGTATCCG<br>CTCATGAGACAATAACCCTGATAAATGCTTCAATAATATTGAAAAAGGAAGAGTATGAGCCATATTCAACGGGAAACGTCTTGCTCTAGGCCGCGATTAAATTCC<br>AACATGGATGCTGATTTATATGGGTATAAATGGGCTCGCGATAATGTCGGGCAATCAGGTGCGACAATCTATCGATTGTATGGGAAGCCCGATGCGCCAGAGTTG<br>TTTCTGAAACATGGCAAAGGTAGCGTTGCCAATGATGTTACAGATGAGATGGTCAGACTAAACTGGCTGACGGAATTTATGCCTCTTCCGACCATCAAGCATTTT<br>ATCCGTACTCCTGATGATGCATGGTTACTCACCACTGCGATCCCCGGGAAAACAGCATTCCAGGTATTAGAAGAATATCCTGATTCAGGTGAAAATATTGTTGAT<br>GCGCTGGCAGTGTTCCTGCGCCGGTTGCATTCGATTCCTGTTTGTAATTGTCCTTTTAACAGCGACCGCGTATTTCGTCTCGCTCAGGCGCAATCACGAATGAAT<br>AACGGTTTGGTTGATGCGAGTGATTTTGATGACGAGCGTAATGGCTGGCCTGTTGAACAAGTCTGGAAAGAAATGCATAAACTTTTGCCATTCTCACCGGATTCA<br>GTCGTCACTCATGGTGATTTCTCACTTGATAACCTTATTTTTGACGAGGGGAAATTAATAGGTTGTATTGATGTTGGACGAGTCGGAATCGCAGACCGATACCAG<br>GATCTTGCCATCCTATGGAACTGCCTCGGTGAGTTTTCTCCTTCATTACAGAAACGGCTTTTTCAAAAATATGGTATTGATAATCCTGATATGAATAAATTGCAG<br>TTTCATTTGATGCTCGATGAGTTTTTCTAAAGCTCGCTTGGACTCCTGTTGATAGATCCAGTAATGACCTCAGAACTCCATCTGGATTTGTTCAGAACGCTCGGT<br>TGCCGCCGGGCGTTTTTTATTGGTGAGAATCCAAGCACAAGAAGATCCGGCCACGATGCGTCCGGCGTAGAGGATCTGAAGATCAGCAGTTCAACCTGTTGATAG<br>TACGTACTAAGCTCTCATGTTTCACGTACTAAGCTCTCATGTTTAACGTACTAAGCTCTCATGTTTAACGAACTAAACCCTCATGGCTAACGTACTAAGCTCTCA<br>TGGCTAACGTACTAAGCTCTCATGTTTCACGTACTAAGCTCTCATGTTTGAACAATAAAATTAATATAAATCAGCAACTTAAATAGCCTCTAAGGTTTTAAGTTT<br>TATAAGAAAAAAAAGAATATATAAGGCTTTTAAAGCTTTTAAGGTTTAACGGTTGTGGACAACAAGCCAGGGATGTAACGCACTGAGAAGCCCTTAGAGCCTCTC<br>AAAGCAATTTTGAGTGACACAGGAACACTTAACGGCTGACATGGGAATTAGCCATGGGCCCGTGCGAATCATTTTTACGGTTCCTGGCCTTTTGCTGGCCTTTTG<br>CTCACATGTTCTTTCCTGCGTGGATAACCGTATTACCGCCTGGACCAAAACGAAAAAACACCCTTTCGGGTGTCTTTTCTGGAATTTGGTACCGAGCCTTTGGTC<br>GAAAAAAAAAGCCCGCACTGTCAGGTGCGGGCTTTTTCTGTGTTTCCAGCCAAGAGCAGTGCCTTGCAGAAGTTCTCGGGCGAAGAGCTACTCCCCTTCTGCGC<br>CGTCCGAAGTTCCTATACTTTCTAGAGAATAGGAACTTCGGACCAAAACGAAAAAACACCCTTTCGGGTGTCTTTTCTGGAATTTGGTACCGAGCCTTTGGTCGA<br>AAAAAAAAGCCCGCACTGTCAGGTGCGGGCTTTTTCTGTGTTTCCCGTTAACAGCGCAGAACTCAAGCAGGATGTTTGACGCGGGCGGCGTCAAG
YJP_MKC149_LP#1	AAAAAAAACCCCGCCCTGTCAGGGGCGGGGTTTTTTTTTTTCTTTTGGGTATAGCGTCGTGGACAGTCATTCATCTTTCTGCCCCTCCAAAAGCAAAAACCCGC<br>CGAAGCGGGTTTTTACGTAAATCAGGTGAAACTGACCGATAAGCCGGGACGAGCTGAGCAGTATGTCGACGGTCCGGCGAGAATCTCGCGTAGACACATACATGA<br>GCAGCGAAAAGAGGTGGCCAGGGGGATCACCTGGCAGCATGCTGCCTAATAAAAAAGGCCGGAAAATCCGGCCTTTTGACGCTTTAGCTCTGACGGCTCAGTGCT<br>GCCCACAGGCTTTCTTCAACACCGGTCAGCAGTTCATCGGTCAGGCTCAGATAATCGTTCTCGATCATTTCATACACTTCCATAAAGCTGCCAAACAGAATTGCA<br>ATCAGTGCATTTTCCGGCAGATTACGAATCACACCCTGTTTCTGACCTTCACGAAAAAGGTGCACACAAATTCAACCAGTTTCTGATATGCCAGACGGCTTTCT<br>TCGGTCAGAAAGGTGCCCTGGCTATGGGTTTTGATAAAACCCAGTGCACGCGGATGATTTTTGGTAAAGGTCACCATACCTTCAAAAATGTGATGAAAGCCATCA<br>CGATAACCATCACGTTCATTTGCCAGACCGCTTTCAATACACTGCAGAAATTCATTAACATGCTGCTGAAACAGTTCGTTCACCAGGCTTTCTTTGTTTTTGAAA<br>TAGCGATAAATGGTGCCTGCACCAACTTTTGCATTTTCTGCAATCATCGGCATGGTGGTTGCATCAAAACCACGTTCTGCAAACAGCAGCAGGCTTGCGCTAAAA<br>ATTGCTTTCTGTTTGGTCGGGGTGCTTTCCATCCTCCCGGCCTCGTTTGGTTAAACAAAATTATTTGTAGAGGACTGTTTCGGCCCTTTTGGGCCATCGTCAGGT<br>CGGATACACATCCGGCGACAGTCTAATTGTGAGCGCTCACAATTCCACACATTATACGAGCCGATGATTAATTGTCAACACAGCCAACGATCGTTCTCCTGGTCT<br>TCCAGTACAATCTGCTCTGATGCCCTCGAGTGCGGCCGCACTAGTGTCGACGGAGCTCGAATTCGGATCCGAAGTTCCTATACTTTCTAGAGAATAGGAACTTCG<br>GAATAGGAACTTCTATTTTTATAGGTTAATGTCATGATAATAATGGTTTCTTAGACCATGCCTATTTGTTTATTTTTCTAAATACATTCAAATATGTATCCGCTC<br>ATGAGACAATAACCCTGATAAATGCTTCAATAATATTGAAAAAGGAAGAGTATGAGCCATATTCAACGGGAAACGTCTTGCTCTAGGCCGCGATTAAATTCCAAC<br>ATGGATGCTGATTTATATGGGTATAAATGGGCTCGCGATAATGTCGGGCAATCAGGTGCGACAATCTATCGATTGTATGGGAAGCCCGATGCGCCAGAGTTGTTT<br>CTGAAACATGGCAAAGGTAGCGTTGCCAATGATGTTACAGATGAGATGGTCAGACTAAACTGGCTGACGGAATTTATGCCTCTTCCGACCATCAAGCATTTTATC<br>CGTACTCCTGATGATGCATGGTTACTCACCACTGCGATCCCCGGGAAAACAGCATTCCAGGTATTAGAAGAATATCCTGATTCAGGTGAAAATATTGTTGATGCG<br>CTGGCAGTGTTCCTGCGCCGGTTGCATTCGATTCCTGTTTGTAATTGTCCTTTTAACAGCGACCGCGTATTTCGTCTCGCTCAGGCGCAATCACGAATGAATAAC<br>GGTTTGGTTGATGCGAGTGATTTTGATGACGAGCGTAATGGCTGGCCTGTTGAACAAGTCTGGAAAGAAATGCATAAACTTTTGCCATTCTCACCGGATTCAGTC<br>GTCACTCATGGTGATTTCTCACTTGATAACCTTATTTTTGACGAGGGGAAATTAATAGGTTGTATTGATGTTGGACGAGTCGGAATCGCAGACCGATACCAGGAT<br>CTTGCCATCCTATGGAACTGCCTCGGTGAGTTTTCTCCTTCATTACAGAAACGGCTTTTTCAAAAATATGGTATTGATAATCCTGATATGAATAAATTGCAGTTT<br>CATTTGATGCTCGATGAGTTTTTCTAAAGCTCGCTTGGACTCCTGTTGATAGATCCAGTAATGACCTCAGAACTCCATCTGGATTTGTTCAGAACGCTCGGTTGC<br>CGCCGGGCGTTTTTTATTGGTGAGAATCCAAGCACAAGAAGATCCGGCCACGATGCGTCCGGCGTAGAGGATCTGAAGATCAGCAGTTCAACCTGTTGATAGTAC<br>GTACTAAGCTCTCATGTTTCACGTACTAAGCTCTCATGTTTAACGTACTAAGCTCTCATGTTTAACGAACTAAACCCTCATGGCTAACGTACTAAGCTCTCATGG<br>CTAACGTACTAAGCTCTCATGTTTCACGTACTAAGCTCTCATGTTTGAACAATAAAATTAATATAAATCAGCAACTTAAATAGCCTCTAAGGTTTTAAGTTTTAT<br>AAGAAAAAAAAGAATATATAAGGCTTTTAAAGCTTTTAAGGTTTAACGGTTGTGGACAACAAGCCAGGGATGTAACGCACTGAGAAGCCCTTAGAGCCTCTCAAA<br>GCAATTTTGAGTGACACAGGAACACTTAACGGCTGACATGGGAATTAGCCATGGGCCCGTGCGAATCATTTTTACGGTTCCTGGCCTTTTGCTGGCCTTTTGCTC<br>ACATGTTCTTTCCTGCGTGGATAACCGTATTACCGCCTGGACCAAAACGAAAAAACACCCTTTCGGGTGTCTTTTCTGGAATTTGGTACCGAGCCTTTGGTCGAA<br>AAAAAAAGCCCGCACTGTCAGGTGCGGGCTTTTTCTGTGTTTCCAGCCAAGAGCAGTGCCTTGCAGAAGTTCTCGGGCGAAGAGCTACTCCCCTTCTGCGCCGT

	CCGAAGTTCCTATACTTTCTAGAGAATAGGAACTTCGGACCAAAACGAAAAAACACCCTTTCGGGTGTCTTTTCTGGAATTTGGTACCGAGCCTTTGGTCGAAAA<br>AAAAAGCCCGCACTGTCAGGTGCGGGCTTTTTTCTGTGTTTCCCGTTAACAGCGCAGAACTCAAGCAGGATGTTTGACGCGGGCGGCGTCAAG
YJP_MKC154_LP#1	AAAAAAAACCCCGCCCTGTCAGGGGCGGGGTTTTTTTTTTTTCTTTTGGGTATAGCGTCGTGGACAGTCATTCATCTTTCTGCCCCTCCAAAAGCAAAAACCCGC<br>CGAAGCGGGTTTTTACGTAAATCAGGTGAAACTGACCGATAAGCCGGGACGAGCTGAGCAGTATGTCGACGGTCCGGCGAGAATCTCGCGTAGACACATACATGA<br>GCAGCGCATAAAAAAATGGCGCCGATGGGCGCCATTTTTCACTGTGTCATACGTAAAAAAGGGCGATCTTGCGACCGCCCTTTTTTTATTAAATGTTTATTTTGC<br>CAGCGGATTACGCAGACCTGCATCAATCAGATCAACCAGCTGATCACAACGGGTTGCCAGATTACCAATATCTTCTTGTGCAATCAGAACCGGATGAATAACGCT<br>GGTCAGTGCATGCAGAACGGTTTCTGCCAGAACCGGAATATCGGTTGCAATATACAGACCTGCTTCAACACCATCACGAATAATTTCGGCCAGCAGTTTTGCAAT<br>CACGCTTTTGTAATAATCGCCACATTTCATATCCTGTTTGGCGGTCATCAGGATCTGAATAAAAACCCGTATGTGTTTGAAATGATCCTGATGATGCTGTTCCAT<br>CAGATTACGTGCCAGATGACGCAGACGATCCAGCGGTGCATGGCTTTTATCCAGCGGACAAATCTGACGTTCAAAAACACCAATCTGACCAAAACCAATTGCATC<br>AACCAGTGCGTTTTTGCTGCTAAAATGTTTAAACACATTTGCCGGACTCATATTCAGTGCGCTTGCAATATCTGCAATGGCAACTGCATTAAAACCACGTTCACT<br>AAACAGTTCTTCGGCTTTTGCCAGAATATCACGACGGGTTTCTTCGGCACTATGACGCGGACGACGACGAACCGGCAGATCGCTTTTACGATCACCTTTACGCAC<br>CTGATCAATGGTTTTGTTCATCTATTAGCTCCTGTTTGAAACATAGTCCATAGCGTATTAAACAAAATTATTTGTAGAGGGGGTTTCGTCATCACTGACTCATCA<br>GAGTTGAAAGCACATCAACTGACCCCTAATTGTGAGCGCTCACAATTCCACACATTATACGAGCCGATGATTAATTGTCAACACAGCCAACGATCGTTCTCCTGG<br>TCTTCCAGTACAATCTGCTCTGATGCCCTCGAGTGCGGCCGCACTAGTGTCGACGGAGCTCGAATTCGGATCCGAAGTTCCTATACTTTCTAGAGAATAGGAACT<br>TCGGAATAGGAACTTCTATTTTTATAGGTTAATGTCATGATAATAATGGTTTCTTAGACCATGCCTATTTGTTTATTTTTCTAAATACATTCAAATATGTATCCG<br>CTCATGAGACAATAACCCTGATAAATGCTTCAATAATATTGAAAAAGGAAGAGTATGAGCCATATTCAACGGGAAACGTCTTGCTCTAGGCCGCGATTAAATTCC<br>AACATGGATGCTGATTTATATGGGTATAAATGGGCTCGCGATAATGTCGGGCAATCAGGTGCGACAATCTATCGATTGTATGGGAAGCCCGATGCGCCAGAGTTG<br>TTTCTGAAACATGGCAAAGGTAGCGTTGCCAATGATGTTACAGATGAGATGGTCAGACTAAACTGGCTGACGGAATTTATGCCTCTTCCGACCATCAAGCATTTT<br>ATCCGTACTCCTGATGATGCATGGTTACTCACCACTGCGATCCCCGGGAAAACAGCATTCCAGGTATTAGAAGAATATCCTGATTCAGGTGAAAATATTGTTGAT<br>GCGCTGGCAGTGTTCCTGCGCCGGTTGCATTCGATTCCTGTTTGTAATTGTCCTTTTAACAGCGACCGCGTATTTCGTCTCGCTCAGGCGCAATCACGAATGAAT<br>AACGGTTTGGTTGATGCGAGTGATTTTGATGACGAGCGTAATGGCTGGCCTGTTGAACAAGTCTGGAAAGAAATGCATAAACTTTTGCCATTCTCACCGGATTCA<br>GTCGTCACTCATGGTGATTTCTCACTTGATAACCTTATTTTTGACGAGGGGAAATTAATAGGTTGTATTGATGTTGGACGAGTCGGAATCGCAGACCGATACCAG<br>GATCTTGCCATCCTATGGAACTGCCTCGGTGAGTTTTCTCCTTCATTACAGAAACGGCTTTTTCAAAAATATGGTATTGATAATCCTGATATGAATAAATTGCAG<br>TTTCATTTGATGCTCGATGAGTTTTTCTAAAGCTCGCTTGGACTCCTGTTGATAGATCCAGTAATGACCTCAGAACTCCATCTGGATTTGTTCAGAACGCTCGGT<br>TGCCGCCGGGCGTTTTTTATTGGTGAGAATCCAAGCACAAGAAGATCCGGCCACGATGCGTCCGGCGTAGAGGATCTGAAGATCAGCAGTTCAACCTGTTGATAG<br>TACGTACTAAGCTCTCATGTTTCACGTACTAAGCTCTCATGTTTAACGTACTAAGCTCTCATGTTTAACGAACTAAACCCTCATGGCTAACGTACTAAGCTCTCA<br>TGGCTAACGTACTAAGCTCTCATGTTTCACGTACTAAGCTCTCATGTTTGAACAATAAAATTAATATAAATCAGCAACTTAAATAGCCTCTAAGGTTTTAAGTTT<br>TATAAGAAAAAAAAGAATATATAAGGCTTTTAAAGCTTTTAAGGTTTAACGGTTGTGGACAACAAGCCAGGGATGTAACGCACTGAGAAGCCCTTAGAGCCTCTC<br>AAAGCAATTTTGAGTGACACAGGAACACTTAACGGCTGACATGGGAATTAGCCATGGGCCCGTGCGAATCATTTTTACGGTTCCTGGCCTTTTGCTGGCCTTTTG<br>CTCACATGTTCTTTCCTGCGTGGATAACCGTATTACCGCCTGGACCAAAACGAAAAAACACCCTTTCGGGTGTCTTTTCTGGAATTTGGTACCGAGCCTTTGGTC<br>GAAAAAAAAAGCCCGCACTGTCAGGTGCGGGCTTTTTTCTGTGTTTCCAGCCAAGAGCAGTGCCTTGCAGAAGTTCTCGGGCGAAGAGCTACTCCCCTTCTGCGC<br>CGTCCGAAGTTCCTATACTTTCTAGAGAATAGGAACTTCGGACCAAAACGAAAAAACACCCTTTCGGGTGTCTTTTCTGGAATTTGGTACCGAGCCTTTGGTCGA<br>AAAAAAAAGCCCGCACTGTCAGGTGCGGGCTTTTTTCTGTGTTTCCCGTTAACAGCGCAGAACTCAAGCAGGATGTTTGACGCGGGCGGCGTCAAG
YJP_MKC155_LP#1	AAAAAAAACCCCGCCCTGTCAGGGGCGGGGTTTTTTTTTTTTCTTTTGGGTATAGCGTCGTGGACAGTCATTCATCTTTCTGCCCCTCCAAAAGCAAAAACCCGC<br>CGAAGCGGGTTTTTACGTAAATCAGGTGAAACTGACCGATAAGCCGGGACGAGCTGAGCAGTATGTCGACGGTCCGGCGAGAATCTCGCGTAGACACATACATGA<br>GCAGCGAGAAAAGCAGAAAAAACGCCGCTGAACGCGGCGTTTTGGATTGTTTGCTGTTTCTTCAGAAGAGGTAGCGTGACCGACTTCCCGGTCACGCTAAGAATG<br>ATTATTAATCGGTCGGCAGATGCTGGGTGATAAAATGACGGGTCAGGCTATTTGCACGGGTTTTATCCAGCGGTTTACCGCTCAGTGCTGCACGCAGCCACAGAC<br>CATCAATCAGTGCTGCCAGACCATAACCTGCCTCTTGTGCCTGTTCACGAGGCAGTTCACGACGAAATTCGCTAACCAGATTGCTCAGCAGACGACGACTGCTAA<br>CCTGCTGCAGACGATACAGCATCGGCTGATGCATGCTGCTTGCCCAAAATGCCAGCCATGCTTTCATTGCTGCGCTGCTAACCTGGGTTTCATCAAAATTACCAC<br>CAACAATTGCCTGCAGACGCTGTTCTGCGCTACCCTGCGGCAGTGCATGCAGACGATTCAGAACTGCATCACGCAGCTGGCTGGTAATATCACGCATGGTTGCTT<br>CCAGCAGACCGTTTTTATCGCGGAAATAATGGCTAATAATACCGGTGCTAACACCGGCACGACGTGCAATCTGTGCAATGGTTGCATCATGCATACCAACTTCAT<br>TAATTGCTTCCAGGGTTGCATCAATCAGCTGACGACGACGAATGCTCTGCATACCCAGTTTCGGCATGTGCTACTCCTCGGGGGGTTAAACAAAATTATTTGTAG<br>AGGAAGTTTCGTCGTCACCGACTCATCAGAATTAAAAGCACATTAATTGACTTCTAATTGTGAGCGCTCACAATTCCACACATTATACGAGCCGATGATTAATTG<br>TCAACACAGCCAACGATCGTTCTCCTGGTCTTCCAGTACAATCTGCTCTGATGCCCTCGAGTGCGGCCGCACTAGTGTCGACGGAGCTCGAATTCGGATCCGAAG<br>TTCCTATACTTTCTAGAGAATAGGAACTTCGGAATAGGAACTTCTATTTTTATAGGTTAATGTCATGATAATAATGGTTTCTTAGACCATGCCTATTTGTTTATT<br>TTTCTAAATACATTCAAATATGTATCCGCTCATGAGACAATAACCCTGATAAATGCTTCAATAATATTGAAAAAGGAAGAGTATGAGCCATATTCAACGGGAAAC<br>GTCTTGCTCTAGGCCGCGATTAAATTCCAACATGGATGCTGATTTATATGGGTATAAATGGGCTCGCGATAATGTCGGGCAATCAGGTGCGACAATCTATCGATT<br>GTATGGGAAGCCCGATGCGCCAGAGTTGTTTCTGAAACATGGCAAAGGTAGCGTTGCCAATGATGTTACAGATGAGATGGTCAGACTAAACTGGCTGACGGAATT<br>TATGCCTCTTCCGACCATCAAGCATTTTATCCGTACTCCTGATGATGCATGGTTACTCACCACTGCGATCCCCGGGAAAACAGCATTCCAGGTATTAGAAGAATA<br>TCCTGATTCAGGTGAAAATATTGTTGATGCGCTGGCAGTGTTCCTGCGCCGGTTGCATTCGATTCCTGTTTGTAATTGTCCTTTTAACAGCGACCGCGTATTTCG<br>TCTCGCTCAGGCGCAATCACGAATGAATAACGGTTTGGTTGATGCGAGTGATTTTGATGACGAGCGTAATGGCTGGCCTGTTGAACAAGTCTGGAAAGAAATGCA<br>TAAACTTTTGCCATTCTCACCGGATTCAGTCGTCACTCATGGTGATTTCTCACTTGATAACCTTATTTTTGACGAGGGGAAATTAATAGGTTGTATTGATGTTGG<br>ACGAGTCGGAATCGCAGACCGATACCAGGATCTTGCCATCCTATGGAACTGCCTCGGTGAGTTTTCTCCTTCATTACAGAAACGGCTTTTTCAAAAATATGGTAT<br>TGATAATCCTGATATGAATAAATTGCAGTTTCATTTGATGCTCGATGAGTTTTTCTAAAGCTCGCTTGGACTCCTGTTGATAGATCCAGTAATGACCTCAGAACT<br>CCATCTGGATTTGTTCAGAACGCTCGGTTGCCGCCGGGCGTTTTTTATTGGTGAGAATCCAAGCACAAGAAGATCCGGCCACGATGCGTCCGGCGTAGAGGATCT<br>GAAGATCAGCAGTTCAACCTGTTGATAGTACGTACTAAGCTCTCATGTTTCACGTACTAAGCTCTCATGTTTAACGTACTAAGCTCTCATGTTTAACGAACTAAA<br>CCCTCATGGCTAACGTACTAAGCTCTCATGGCTAACGTACTAAGCTCTCATGTTTCACGTACTAAGCTCTCATGTTTGAACAATAAAATTAATATAAATCAGCAA<br>CTTAAATAGCCTCTAAGGTTTTAAGTTTTATAAGAAAAAAAAGAATATATAAGGCTTTTAAAGCTTTTAAGGTTTAACGGTTGTGGACAACAAGCCAGGGATGTA<br>ACGCACTGAGAAGCCCTTAGAGCCTCTCAAAGCAATTTTGAGTGACACAGGAACACTTAACGGCTGACATGGGAATTAGCCATGGGCCCGTGCGAATCATTTTTA<br>CGGTTCCTGGCCTTTTGCTGGCCTTTTGCTCACATGTTCTTTCCTGCGTGGATAACCGTATTACCGCCTGGACCAAAACGAAAAAACACCCTTTCGGGTGTCTTT<br>TCTGGAATTTGGTACCGAGCCTTTGGTCGAAAAAAAAAGCCCGCACTGTCAGGTGCGGGCTTTTTTCTGTGTTTCCAGCCAAGAGCAGTGCCTTGCAGAAGTTCT<br>CGGGCGAAGAGCTACTCCCCTTCTGCGCCGTCCGAAGTTCCTATACTTTCTAGAGAATAGGAACTTCGGACCAAAACGAAAAAACACCCTTTCGGGTGTCTTTTC<br>TGGAATTTGGTACCGAGCCTTTGGTCGAAAAAAAAAGCCCGCACTGTCAGGTGCGGGCTTTTTTCTGTGTTTCCCGTTAACAGCGCAGAACTCAAGCAGGATGTT<br>TGACGCGGGCGGCGTCAAG
YJP_MKC156_LP#1	AAAAAAAACCCCGCCCTGTCAGGGGCGGGGTTTTTTTTTTTTCTTTTGGGTATAGCGTCGTGGACAGTCATTCATCTTTCTGCCCCTCCAAAAGCAAAAACCCGC<br>CGAAGCGGGTTTTTACGTAAATCAGGTGAAACTGACCGATAAGCCGGGACGAGCTGAGCAGTATGTCGACGGTCCGGCGAGAATCTCGCGTAGACACATACATGA<br>GCAGTAGGACCAAAACGAAAAAGGCCCCCCTTTCGGGAGGCCTCTTTTCTGGAATTTGGTACCGAGTGCAGACGTAAAAAAAGCGGCGTGGTTAGCCGCTTTTT<br>TAATTGCCGGATTAACGCTGTGTACCCGGACAAACACCATTAATCAGCAGGAAGGTAAATTCTTCAATATCCTGTTCAACGGTCAGCTGTTCGGTCAGCAGGCGA<br>TACCAACAAAAACCAAAAATCATATCCAGCAGCAGTTCACGATTGGTATCTTTCGGCAGTTCACCATTGCTAATGGCATTTTCAACCAGTTTTTCGGCATCTCA<br>CGACGACGTTCCATAAACTGATCTTTCAGCTGGGTCAGGGTTGCAGGGTCCAGCTGTGCTTCTGCAATAACACAACGAAATGCTTCACCACAAATGGTTTCACGC<br>CAAACTTTCCACAGATTACGCAGCAGAAAATCCAGATCGGCTTTAAAGCTACCCAGATCCGGAAATTTACGCACCTGTTCGCTTTCATTTTCATACACTTCGGCA<br>ATCAGTGCTGCTTTATTGGTCCACCAACGATAAATGGTCGGTTTGCTTGCACCGGCACGACGTGCAACGCTTTCAATGCTCAGACCGCTATAACCACATTCTTTC<br>AGGATTTCAATGGTGCTGGTCAGAATTGCTTTATGGGTATGCGGACTACGCAGGCTACCAATGCTGCTACGGCTCGGGGTACGTGCCATCTAGTATTTCTCCCTT<br>TCAAACATAGTCCATAGTTAAACAAAATTATTTGTAGAGGCGGTTTCGACATCACTGTCTCATCAGAACGCAAAGCACATGCGTTGACCGCTAATTGTGAGCGCT<br>CACAATTCCACACATTATACGAGCCGATGATTAATTGTCAACACAGCCAACGATCGTTCTCCTGGTCTTCCAGTACAATCTGCTCTGATGCCCTCGAGTGCGGCC<br>GCACTAGTGTCGACGGAGCTCGAATTCGGATCCGAAGTTCCTATACTTTCTAGAGAATAGGAACTTCGGAATAGGAACTTCTATTTTTATAGGTTAATGTCATGA<br>TAATAATGGTTTCTTAGACCATGCCTATTTGTTTATTTTTCTAAATACATTCAAATATGTATCCGCTCATGAGACAATAACCCTGATAAATGCTTCAATAATATT<br>GAAAAAGGAAGAGTATGAGCCATATTCAACGGGAAACGTCTTGCTCTAGGCCGCGATTAAATTCCAACATGGATGCTGATTTATATGGGTATAAATGGGCTCGCG<br>ATAATGTCGGGCAATCAGGTGCGACAATCTATCGATTGTATGGGAAGCCCGATGCGCCAGAGTTGTTTCTGAAACATGGCAAAGGTAGCGTTGCCAATGATGTTA<br>CAGATGAGATGGTCAGACTAAACTGGCTGACGGAATTTATGCCTCTTCCGACCATCAAGCATTTTATCCGTACTCCTGATGATGCATGGTTACTCACCACTGCGA<br>TCCCCGGGAAAACAGCATTCCAGGTATTAGAAGAATATCCTGATTCAGGTGAAAATATTGTTGATGCGCTGGCAGTGTTCCTGCGCCGGTTGCATTCGATTCCTG<br>TTTGTAATTGTCCTTTTAACAGCGACCGCGTATTTCGTCTCGCTCAGGCGCAATCACGAATGAATAACGGTTTGGTTGATGCGAGTGATTTTGATGACGAGCGTA<br>ATGGCTGGCCTGTTGAACAAGTCTGGAAAGAAATGCATAAACTTTTGCCATTCTCACCGGATTCAGTCGTCACTCATGGTGATTTCTCACTTGATAACCTTATTT<br>TTGACGAGGGGAAATTAATAGGTTGTATTGATGTTGGACGAGTCGGAATCGCAGACCGATACCAGGATCTTGCCATCCTATGGAACTGCCTCGGTGAGTTTTCTC<br>CTTCATTACAGAAACGGCTTTTTCAAAAATATGGTATTGATAATCCTGATATGAATAAATTGCAGTTTCATTTGATGCTCGATGAGTTTTTCTAAAGCTCGCTTG<br>GACTCCTGTTGATAGATCCAGTAATGACCTCAGAACTCCATCTGGATTTGTTCAGAACGCTCGGTTGCCGCCGGGCGTTTTTTATTGGTGAGAATCCAAGCACAA<br>GAAGATCCGGCCACGATGCGTCCGGCGTAGAGGATCTGAAGATCAGCAGTTCAACCTGTTGATAGTACGTACTAAGCTCTCATGTTTCACGTACTAAGCTCTCAT<br>GTTTAACGTACTAAGCTCTCATGTTTAACGAACTAAACCCTCATGGCTAACGTACTAAGCTCTCATGGCTAACGTACTAAGCTCTCATGTTTCACGTACTAAGCT<br>CTCATGTTTGAACAATAAAATTAATATAAATCAGCAACTTAAATAGCCTCTAAGGTTTTAAGTTTTATAAGAAAAAAAAGAATATATAAGGCTTTTAAAGCTTTT<br>AAGGTTTAACGGTTGTGGACAACAAGCCAGGGATGTAACGCACTGAGAAGCCCTTAGAGCCTCTCAAAGCAATTTTGAGTGACACAGGAACACTTAACGGCTGAC<br>ATGGGAATTAGCCATGGGCCCGTGCGAATCATTTTTACGGTTCCTGGCCTTTTGCTGGCCTTTTGCTCACATGTTCTTTCCTGCGTGGATAACCGTATTACCGCC<br>TGGACCAAAACGAAAAAACACCCTTTCGGGTGTCTTTTCTGGAATTTGGTACCGAGCCTTTGGTCGAAAAAAAAAGCCCGCACTGTCAGGTGCGGGCTTTTTTCT<br>GTGTTTCCAGCCAAGAGCAGTGCCTTGCAGAAGTTCTCGGGCGAAGAGCTACTCCCCTTCTGCGCCGTCCGAAGTTCCTATACTTTCTAGAGAATAGGAACTTCG

	GACCAAAACGAAAAAACACCCTTTCGGGTGTCTTTTCTGGAATTTGGTACCGAGCCTTTGGTCGAAAAAAAAAGCCCGCACTGTCAGGTGCGGGCTTTTTTCTGT<br>GTTTCCCGTTAACAGCGCAGAACTCAAGCAGGATGTTTGACGCGGGCGGCGTCAAG
YJP_MKC158_LP#1	AAAAAAAACCCCGCCCTGTCAGGGGCGGGGTTTTTTTTTTTTCTTTTGGGTATAGCGTCGTGGACAGTCATTCATCTTTCTGCCCCTCCAAAAGCAAAAACCCGC<br>CGAAGCGGGTTTTTACGTAAATCAGGTGAAACTGACCGATAAGCCGGGACGAGCTGAGCAGTATGTCGACGGTCCGGCGAGAATCTCGCGTAGACACATACATGA<br>GCAGCGAGAAAAGCAGAAAAAACGCCGCTGAACGCGGCGTTTTGGATTGTTTGCTGTTTCTTCAGAAGAGGTAGCGTGACCGACTTCCCGGTCACGCTAAGAATG<br>ATTATTAATCGGTCGGCAGATGCTGGGTGATAAAATGACGGGTCAGGCTATTTGCACGGGTTTTATCCAGCGGTTTACCGCTCAGTGCTGCACGCAGCCACAGAC<br>CATCAATCAGTGCTGCCAGACCATAACCTGCCTCTTGTGCCTGTTCACGAGGCAGTTCACGACGAAATTCGCTAACCAGATTGCTCAGCAGACGACGACTGCTAA<br>CCTGCTGCAGACGATACAGCATCGGCTGATGCATGCTGCTTGCCCAAAATGCCAGCCATGCTTTCATTGCTGCGCTGCTAACCTGGGTTTCATCAAAATTACCAC<br>CAACAATTGCCTGCAGACGCTGTTCTGCGCTACCCTGCGGCAGTGCATGCAGACGATTCAGAACTGCATCACGCAGCTGGCTGGTAATATCACGCATGGTTGCTT<br>CCAGCAGACCGTTTTTATCGCGGAAATAATGGCTAATAATACCGGTGCTAACACCGGCACGACGTGCAATCTGTGCAATGGTTGCATCATGCATACCAACTTCAT<br>TAATTGCTTCCAGGGTTGCATCAATCAGCTGACGACGACGAATGCTCTGCATACCCAGTTTCGGCATTGTGCTCCTCCTCGGAAGGTTAAACAAAATTATTTGTA<br>GAGGAAGTTTCGTCGTCACCGACTCATCAGAATTAAAAGCACATTAATTGACTTCTAATTGTGAGCGCTCACAATTCCACACATTATACGAGCCGATGATTAATT<br>GTCAACACAGCCAACGATCGTTCTCCTGGTCTTCCAGTACAATCTGCTCTGATGCCCTCGAGTGCGGCCGCACTAGTGTCGACGGAGCTCGAATTCGGATCCGAA<br>GTTCCTATACTTTCTAGAGAATAGGAACTTCGGAATAGGAACTTCTATTTTTATAGGTTAATGTCATGATAATAATGGTTTCTTAGACCATGCCTATTTGTTTAT<br>TTTTCTAAATACATTCAAATATGTATCCGCTCATGAGACAATAACCCTGATAAATGCTTCAATAATATTGAAAAAGGAAGAGTATGAGCCATATTCAACGGGAAA<br>CGTCTTGCTCTAGGCCGCGATTAAATTCCAACATGGATGCTGATTTATATGGGTATAAATGGGCTCGCGATAATGTCGGGCAATCAGGTGCGACAATCTATCGAT<br>TGTATGGGAAGCCCGATGCGCCAGAGTTGTTTCTGAAACATGGCAAAGGTAGCGTTGCCAATGATGTTACAGATGAGATGGTCAGACTAAACTGGCTGACGGAAT<br>TTATGCCTCTTCCGACCATCAAGCATTTTATCCGTACTCCTGATGATGCATGGTTACTCACCACTGCGATCCCCGGGAAAACAGCATTCCAGGTATTAGAAGAAT<br>ATCCTGATTCAGGTGAAAATATTGTTGATGCGCTGGCAGTGTTCCTGCGCCGGTTGCATTCGATTCCTGTTTGTAATTGTCCTTTTAACAGCGACCGCGTATTTC<br>GTCTCGCTCAGGCGCAATCACGAATGAATAACGGTTTGGTTGATGCGAGTGATTTTGATGACGAGCGTAATGGCTGGCCTGTTGAACAAGTCTGGAAAGAAATGC<br>ATAAACTTTTGCCATTCTCACCGGATTCAGTCGTCACTCATGGTGATTTCTCACTTGATAACCTTATTTTTGACGAGGGGAAATTAATAGGTTGTATTGATGTTG<br>GACGAGTCGGAATCGCAGACCGATACCAGGATCTTGCCATCCTATGGAACTGCCTCGGTGAGTTTTCTCCTTCATTACAGAAACGGCTTTTTCAAAAATATGGTA<br>TTGATAATCCTGATATGAATAAATTGCAGTTTCATTTGATGCTCGATGAGTTTTTCTAAAGCTCGCTTGGACTCCTGTTGATAGATCCAGTAATGACCTCAGAAC<br>TCCATCTGGATTTGTTCAGAACGCTCGGTTGCCGCCGGGCGTTTTTTATTGGTGAGAATCCAAGCACAAGAAGATCCGGCCACGATGCGTCCGGCGTAGAGGATC<br>TGAAGATCAGCAGTTCAACCTGTTGATAGTACGTACTAAGCTCTCATGTTTCACGTACTAAGCTCTCATGTTTAACGTACTAAGCTCTCATGTTTAACGAACTAA<br>ACCCTCATGGCTAACGTACTAAGCTCTCATGGCTAACGTACTAAGCTCTCATGTTTCACGTACTAAGCTCTCATGTTTGAACAATAAAATTAATATAAATCAGCA<br>ACTTAAATAGCCTCTAAGGTTTTAAGTTTTATAAGAAAAAAAAGAATATATAAGGCTTTTAAAGCTTTTAAGGTTTAACGGTTGTGGACAACAAGCCAGGGATGT<br>AACGCACTGAGAAGCCCTTAGAGCCTCTCAAAGCAATTTTGAGTGACACAGGAACACTTAACGGCTGACATGGGAATTAGCCATGGGCCCGTGCGAATCATTTTT<br>ACGGTTCCTGGCCTTTTGCTGGCCTTTTGCTCACATGTTCTTTCCTGCGTGGATAACCGTATTACCGCCTGGACCAAAACGAAAAAACACCCTTTCGGGTGTCTT<br>TTCTGGAATTTGGTACCGAGCCTTTGGTCGAAAAAAAAAGCCCGCACTGTCAGGTGCGGGCTTTTTTCTGTGTTTCCAGCCAAGAGCAGTGCCTTGCAGAAGTTC<br>TCGGGCGAAGAGCTACTCCCCTTCTGCGCCGTCCGAAGTTCCTATACTTTCTAGAGAATAGGAACTTCGGACCAAAACGAAAAAACACCCTTTCGGGTGTCTTTT<br>CTGGAATTTGGTACCGAGCCTTTGGTCGAAAAAAAAAGCCCGCACTGTCAGGTGCGGGCTTTTTTCTGTGTTTCCCGTTAACAGCGCAGAACTCAAGCAGGATGT<br>TTGACGCGGGCGGCGTCAAG
YJP_MKC141_LP#2	ACATTTAATAAAAAAAGGGCGGTCGCAAGATCGCCCTTTTTTACGTATGACACAGTGAAAAATGGCGCCCATCGGCGCCATTTTTTTATGGAAGTTCCTATTCTC<br>TAGAAAGTATAGGAACTTCAGACGAGAAACGTTCCGTCCGTCTGGGTCAGTTGCCTAACCTTAACTTTACGCAGGTTCAGCTTGGAAACACAGAAAAAAGCCCG<br>CACCTGACAGTGCGGGCTTTTTTTTTCGACCAAAGGCTCGGTACCAAATTCCAGAAAAGACACCCGAAAGGGTGTTTTTTCGTTTTGGTCCAGGCGGTAATACGG<br>TTATCCACAGAATCAGGGGATAACGCAGGAAAGAACATGTGAGCAAAAGGCCAGCAAAAGGCCAGGAACCGTAAAAATGATTCGCACGGGCCCATGGCTAATTAC<br>CATGTCGGTAATGACTCCAACTTATTGATAGTGTTTTATGTTCAGATAATGCCCGATGACTTTGTCATGCAGCTCCACCGATTTTGAGAACGACAGCGACTTCCG<br>TCCCAGCCGTGCCAGGTGCTGCCTCAGATTCAGGTTATGCCGCTCAATTCGCTGCGTATATCGCTTGCTGATTACGTGCAGCTTTCCCTTCAGGCGGGATTCATA<br>CAGCGGCCAGCCATCCGTCATCCATATCACCACGTCAAAGGGTGACAGCAGGCTCATAAGACGCCCCAGCGTCGCCATAGTGCGTTCACCGAATACGTGCGCAAC<br>AAACGTCTTCCGGAGACTGTCATACGCGTAAAACAGCCAGCGCTGGCGCGATTTAGCCCCGACATAGCCCCACTGTTCGTCCATTTCCGCGCAGACGATGACGTC<br>ACTGCCCGGCTGTATGCGCGAGGTTACCGACTGCGGCCTGAGTTTTTTAAGTGACGTAAAATCGTGTTGAGGCCAACGCCCATAATGCGGGCTGTTGCCCGGCAT<br>CCAACGCCATTCATGGCCATATCAATGATTTTCTGGTGCGTACCGGGTTGAGAAGCGGTGTAAGTGAACTGCAGTTGCCATGTTTTACGGCAGTGAGAGCAGAGA<br>TAGCGCTGATGTCCGGCGGTGCTTTTGCCGTTACGCACCACACCGTCAGTAGCTGAACAGGAGGGACAGCTGATAGAAACAGAAGCCACTGGAGCACCTCAAAAA<br>CACCATCATACACTAAATCAGTAAGTTGGCAGCATCACCTCCCATGTCAGCCGTTAAGTGTTCCTGTGTCACTCAAAATTGCTTTGAGAGGCTCTAAGGGCTTCT<br>CAGTGCGTTACATCCCTGGCTTGTTGTCCACAACCGTTAAACCTTAAAAGCTTTAAAAGCCTTATATATTCTTTTTTTTCTTATAAAACTTAAAACCTTAGAGGC<br>TATTTAAGTTGCTGATTTATATTAATTTTATTGTTCAAACATGAGAGCTTAGTACGTGAAACATGAGAGCTTAGTACGTTAGCCATGAGAGCTTAGTACGTTAGC<br>CATGAGGGTTTAGTTCGTTAAACATGAGAGCTTAGTACGTTAAACATGAGAGCTTAGTACGTGAAACATGAGAGCTTAGTACGTACTATCAACAGGTTGAACTGC<br>TGATCTTCAGATCCTCTACGCCGGACGCATCGTGGCCGGATCTTCTTGTGCTTGGATTCTCACCAATAAAAAACGCCCGGCGGCAACCGAGCGTTCTGAACAAAT<br>CCAGATGGAGTTCTGAGGTCATTACTGGATCTATCAACAGGAGTCCAAGCGAGCTTTACGCCCCGCCCTGCCACTCATCGCAGTACTGTTGTAATTCATTAAGCA<br>TTCTGCCGACATGGAAGCCATCACAAACGGCATGATGAACCTGAATCGCCAGCGGCATCAGCACCTTGTCGCCTTGCGTATAATATTTGCCCATGGTGAAAACGG<br>GGGCGAAGAAGTTGTCCATATTGGCCACGTTTAAATCAAAACTGGTGAAACTCACCCAGGGATTGGCTGAGACGAAAAACATATTCTCAATAAACCCTTTAGGGA<br>AATAGGCCAGGTTTTCACCGTAACACGCCACATCTTGCGAATATATGTGTAGAAACTGCCGGAAATCGTCGTGGTATTCACTCCAGAGCGATGAAAACGTTTCAG<br>TTTGCTCATGGAAAACGGTGTAACAAGGGTGAACACTATCCCATATCACCAGCTCACCGTCTTTCATTGCCATACGAAATTCCGGATGAGCATTCATCAGGCGGG<br>CAAGAATGTGAATAAAGGCCGGATAAAACTTGTGCTTATTTTTCTTTACGGTCTTTAAAAAGGCCGTAATATCCAGCTGAACGGTCTGGTTATAGGTACATTGAG<br>CAACTGACTGAAATGCCTCAAAATGTTCTTTACGATGCCATTGGGATATATCAACGGTGGTATATCCAGTGATTTTTTTCTCCATTTTAGCTTCCTTAGCTCCTG<br>AAAATCTCGATAACTCAAAAAATACGCCCGGTAGTGATCTTATTTCATTATGGTGAAAGTTGGAACCTCTTACGTGCCGATCAACGTCTCATTTTCGCCAATTTA<br>AATCGTAATTATTGGGGACCCTGTATTTAGAAAAATAAACAAATAGGCATGGTCTAAGAAACCATTATTATCATGACATTAACCTATAAAAATAGAAGTTCCTAT<br>TCCGAAGTTCCTATTCTCTAGAAAGTATAGGAACTTCGGATCCGAATTCGAGCTCCGTCGACACTAGTGCGGCCGCACTCGAGGGCATCAGAGCAGATTGTACTG<br>GAAGACCATACTGGTATGGAAGCTATACGTTACCAATTGACAGCTAGCTCAGTCCTACTTTAGTATATAGACCGTGCGATCGGTCTATAAGCTGTCACCGGATGT<br>GCTTTCCGGTCTGATGAGTCCGTGAGGACGAAACAGCCTCTACAAATAATTTTGTTTAATACTAGAGAAAGAGGAGAAATACTAGATGGTGAGCAAGGGCGAGGA<br>GCTGTTCACCGGGGTGGTGCCCATCCTGGTCGAGCTGGACGGCGACGTAAACGGCCACAAGTTCAGCGTGTCCGGCGAGGGCGAGGGCGATGCCACCTACGGCAA<br>GCTGACCCTGAAGTTCATCTGCACCACAGGCAAGCTGCCCGTGCCCTGGCCCACCCTCGTGACCACCTTCGGCTACGGCCTGCAATGCTTCGCCCGCTACCCCGA<br>CCACATGAAGCTGCACGACTTCTTCAAGTCCGCCATGCCCGAAGGCTACGTCCAGGAGCGCACCATCTTCTTCAAGGACGACGGCAACTACAAGACCCGCGCCGA<br>GGTGAAGTTCGAGGGCGACACCCTGGTGAACCGCATCGAGCTGAAGGGCATCGACTTCAAGGAGGACGGCAACATCCTGGGGCACAAGCTGGAGTACAACTACAA<br>CAGCCACAACGTCTATATCATGGCCGACAAGCAGAAGAACGGCATCAAGGTGAACTTCAAGATCCGCCACAACATCGAGGACGGCAGCGTGCAGCTCGCCGACCA<br>CTACCAGCAGAACACCCCAATCGGCGACGGCCCCGTGCTGCTGCCCGACAACCACTACCTTAGCTACCAGTCCGCCCTGAGCAAAGACCCCAACGAGAAGCGCGA<br>TCACATGGTCCTGCTGGAGTTCGTGACCGCCGCCGGGATCACTCTCGGCATGGACGAGCTGTACAAGTAAGTGTTATAAACCTGTGTGAGAGTTAAGTTTACATG<br>GGCAAAGTTGATGACCGGGTCGTCCGTTGCAGACAAAAAAAATGGCGCACAATGTGCGCCATTTTTCACTTCACAGGTACTATTGTTTTGAATTGAAAAGGGCGC<br>TTCGGCGCCCTTTTTGCATTTGTTGACGGCATATATTTGTATATCGAAGCGCCCTGATGGGCGCTTTTTTTATTTAATCGATAACCAGA
YJP_MKC142_LP#2	ACATTTAATAAAAAAAGGGCGGTCGCAAGATCGCCCTTTTTTACGTATGACACAGTGAAAAATGGCGCCCATCGGCGCCATTTTTTTATGGAAGTTCCTATTCTC<br>TAGAAAGTATAGGAACTTCAGACGAGAAACGTTCCGTCCGTCTGGGTCAGTTGCCTAACCTTAACTTTACGCAGGTTCAGCTTGGAAACACAGAAAAAAGCCCG<br>CACCTGACAGTGCGGGCTTTTTTTTTCGACCAAAGGCTCGGTACCAAATTCCAGAAAAGACACCCGAAAGGGTGTTTTTTCGTTTTGGTCCAGGCGGTAATACGG<br>TTATCCACAGAATCAGGGGATAACGCAGGAAAGAACATGTGAGCAAAAGGCCAGCAAAAGGCCAGGAACCGTAAAAATGATTCGCACGGGCCCATGGCTAATTCC<br>CATGTCAGCCGTTAAGTGTTCCTGTGTCACTCAAAATTGCTTTGAGAGGCTCTAAGGGCTTCTCAGTGCGTTACATCCCTGGCTTGTTGTCCACAACCGTTAAAC<br>CTTAAAAGCTTTAAAAGCCTTATATATTCTTTTTTTTCTTATAAAACTTAAAACCTTAGAGGCTATTTAAGTTGCTGATTTATATTAATTTTATTGTTCAAACAT<br>GAGAGCTTAGTACGTGAAACATGAGAGCTTAGTACGTTAGCCATGAGAGCTTAGTACGTTAGCCATGAGGGTTTAGTTCGTTAAACATGAGAGCTTAGTACGTTA<br>AACATGAGAGCTTAGTACGTGAAACATGAGAGCTTAGTACGTACTATCAACAGGTTGAACTGCTGATCTTCAGATCCTCTACGCCGGACGCATCGTGGCCGGATC<br>TTCTTGTGCTTGGATTCTCACCAATAAAAAACGCCCGGCGGCAACCGAGCGTTCTGAACAAATCCAGATGGAGTTCTGAGGTCATTACTGGATCTATCAACAGGA<br>GTCCAAGCGAGCTTTACGCCCCGCCCTGCCACTCATCGCAGTACTGTTGTAATTCATTAAGCATTCTGCCGACATGGAAGCCATCACAAACGGCATGATGAACCT<br>GAATCGCCAGCGGCATCAGCACCTTGTCGCCTTGCGTATAATATTTGCCCATGGTGAAAACGGGGGCGAAGAAGTTGTCCATATTGGCCACGTTTAAATCAAAAC<br>TGGTGAAACTCACCCAGGGATTGGCTGAGACGAAAAACATATTCTCAATAAACCCTTTAGGGAAATAGGCCAGGTTTTCACCGTAACACGCCACATCTTGCGAAT<br>ATATGTGTAGAAACTGCCGGAAATCGTCGTGGTATTCACTCCAGAGCGATGAAAACGTTTCAGTTTGCTCATGGAAAACGGTGTAACAAGGGTGAACACTATCCC<br>ATATCACCAGCTCACCGTCTTTCATTGCCATACGAAATTCCGGATGAGCATTCATCAGGCGGGCAAGAATGTGAATAAAGGCCGGATAAAACTTGTGCTTATTTT<br>TCTTTACGGTCTTTAAAAAGGCCGTAATATCCAGCTGAACGGTCTGGTTATAGGTACATTGAGCAACTGACTGAAATGCCTCAAAATGTTCTTTACGATGCCATT<br>GGGATATATCAACGGTGGTATATCCAGTGATTTTTTTCTCCATTTTAGCTTCCTTAGCTCCTGAAAATCTCGATAACTCAAAAAATACGCCCGGTAGTGATCTTA<br>TTTCATTATGGTGAAAGTTGGAACCTCTTACGTGCCGATCAACGTCTCATTTTCGCCAATTTAAATCGTAATTATTGGGGACCCTGTATTTAGAAAAATAAACAA<br>ATAGGCATGGTCTAAGAAACCATTATTATCATGACATTAACCTATAAAAATAGAAGTTCCTATTCCGAAGTTCCTATTCTCTAGAAAGTATAGGAACTTCGGATC<br>CGAATTCGAGCTCCGTCGACACTAGTGCGGCCGCACTCGAGGGCATCAGAGCAGATTGTACTGGAAGACCATACTCTTGTCCAACCAAATGATTCGTTACCCTTT<br>GACAGTTTCTATCGATCTATAGATAATGCTAGCAGCTGTCACCGGATGTGCTTTCCGGTCTGATGAGTCCGTGAGGACGAAACAGCCTCTACAAATAATTTTGTT<br>TAATACTAGAGAAAGAGGAGAAATACTAGATGGTGAGCAAGGGCGAGGAGCTGTTCACCGGGGTGGTGCCCATCCTGGTCGAGCTGGACGGCGACGTAAACGGCC<br>ACAAGTTCAGCGTGTCCGGCGAGGGCGAGGGCGATGCCACCTACGGCAAGCTGACCCTGAAGTTCATCTGCACCACAGGCAAGCTGCCCGTGCCCTGGCCCACCC<br>TCGTGACCACCTTCGGCTACGGCCTGCAATGCTTCGCCCGCTACCCCGACCACATGAAGCTGCACGACTTCTTCAAGTCCGCCATGCCCGAAGGCTACGTCCAGG<br>AGCGCACCATCTTCTTCAAGGACGACGGCAACTACAAGACCCGCGCCGAGGTGAAGTTCGAGGGCGACACCCTGGTGAACCGCATCGAGCTGAAGGGCATCGACT

	TCAAGGAGGACGGCAACATCCTGGGGCACAAGCTGGAGTACAACTACAACAGCCACAACGTCTATATCATGGCCGACAAGCAGAAGAACGGCATCAAGGTGAACT<br>TCAAGATCCGCCACAACATCGAGGACGGCAGCGTGCAGCTCGCCGACCACTACCAGCAGAACACCCCAATCGGCGACGGCCCCGTGCTGCTGCCCGACAACCACT<br>ACCTTAGCTACCAGTCCGCCCTGAGCAAAGACCCCAACGAGAAGCGCGATCACATGGTCCTGCTGGAGTTCGTGACCGCCGCCGGGATCACTCTCGGCATGGACG<br>AGCTGTACAAGTAAGTGTTATAAACCTGTGTGAGAGTTAAGTTTACATGGGCAAAGTTGATGACCGGGTCGTCCGTTGCAGACAAAAAAAATGGCGCACAATGTG<br>CGCCATTTTTCACTTCACAGGTACTATTGTTTTGAATTGAAAAGGGCGCTTCGGCGCCCTTTTTGCATTTGTTGACGGCATATATTTGTATATCGAAGCGCCCTG<br>ATGGGCGCTTTTTTTATTTAATCGATAACCAGA
YJP_MKC149_LP#2	ACATTTAATAAAAAAAGGGCGGTCGCAAGATCGCCCTTTTTTACGTATGACACAGTGAAAAATGGCGCCCATCGGCGCCATTTTTTTATGGAAGTTCCTATTCTC<br>TAGAAAGTATAGGAACTTCAGACGAGAAACGTTCCGTCCGTCTGGGTCAGTTGCCTAACCTTAACTTTTACGCAGGTTCAGCTTGGAAACACAGAAAAAAGCCCG<br>CACCTGACAGTGCGGGCTTTTTTTTTCGACCAAAGGCTCGGTACCAAATTCCAGAAAAGACACCCGAAAGGGTGTTTTTTCGTTTTGGTCCAGGCGGTAATACGG<br>TTATCCACAGAATCAGGGGATAACGCAGGAAAGAACATGTGAGCAAAAGGCCAGCAAAAGGCCAGGAACCGTAAAAATGATTCGCACGGGCCCATGGCTAATTAC<br>CATGTCGGTAATGACTCCAACTTATTGATAGTGTTTTATGTTCAGATAATGCCCGATGACTTTGTCATGCAGCTCCACCGATTTTGAGAACGACAGCGACTTCCG<br>TCCCAGCCGTGCCAGGTGCTGCCTCAGATTCAGGTTATGCCGCTCAATTCGCTGCGTATATCGCTTGCTGATTACGTGCAGCTTTCCCTTCAGGCGGGATTCATA<br>CAGCGGCCAGCCATCCGTCATCCATATCACCACGTCAAAGGGTGACAGCAGGCTCATAAGACGCCCCAGCGTCGCCATAGTGCGTTCACCGAATACGTGCGCAAC<br>AAACGTCTTCCGGAGACTGTCATACGCGTAAAACAGCCAGCGCTGGCGCGATTTAGCCCCGACATAGCCCCACTGTTCGTCCATTTCCGCGCAGACGATGACGTC<br>ACTGCCCGGCTGTATGCGCGAGGTTACCGACTGCGGCCTGAGTTTTTTAAGTGACGTAAAATCGTGTTGAGGCCAACGCCCATAATGCGGGCTGTTGCCCGGCAT<br>CCAACGCCATTCATGGCCATATCAATGATTTTCTGGTGCGTACCGGGTTGAGAAGCGGTGTAAGTGAACTGCAGTTGCCATGTTTTACGGCAGTGAGAGCAGAGA<br>TAGCGCTGATGTCCGGCGGTGCTTTTGCCGTTACGCACCACACCGTCAGTAGCTGAACAGGAGGGACAGCTGATAGAAACAGAAGCCACTGGAGCACCTCAAAAA<br>CACCATCATACACTAAATCAGTAAGTTGGCAGCATCACCTCCCATGTCAGCCGTTAAGTGTTCCTGTGTCACTCAAAATTGCTTTGAGAGGCTCTAAGGGCTTCT<br>CAGTGCGTTACATCCCTGGCTTGTTGTCCACAACCGTTAAACCTTAAAAGCTTTAAAAGCCTTATATATTCTTTTTTTTCTTATAAAACTTAAAACCTTAGAGGC<br>TATTTAAGTTGCTGATTTATATTAATTTTATTGTTCAAACATGAGAGCTTAGTACGTGAAACATGAGAGCTTAGTACGTTAGCCATGAGAGCTTAGTACGTTAGC<br>CATGAGGGTTTAGTTCGTTAAACATGAGAGCTTAGTACGTTAAACATGAGAGCTTAGTACGTGAAACATGAGAGCTTAGTACGTACTATCAACAGGTTGAACTGC<br>TGATCTTCAGATCCTCTACGCCGGACGCATCGTGGCCGGATCTTCTTGTGCTTGGATTCTCACCAATAAAAAACGCCCGGCGGCAACCGAGCGTTCTGAACAAAT<br>CCAGATGGAGTTCTGAGGTCATTACTGGATCTATCAACAGGAGTCCAAGCGAGCTTTACGCCCCGCCCTGCCACTCATCGCAGTACTGTTGTAATTCATTAAGCA<br>TTCTGCCGACATGGAAGCCATCACAAACGGCATGATGAACCTGAATCGCCAGCGGCATCAGCACCTTGTCGCCTTGCGTATAATATTTGCCCATGGTGAAAACGG<br>GGGCGAAGAAGTTGTCCATATTGGCCACGTTTAAATCAAAACTGGTGAAACTCACCCAGGGATTGGCTGAGACGAAAAACATATTCTCAATAAACCCTTTAGGGA<br>AATAGGCCAGGTTTTCACCGTAACACGCCACATCTTGCGAATATATGTGTAGAAACTGCCGGAAATCGTCGTGGTATTCACTCCAGAGCGATGAAAACGTTTCAG<br>TTTGCTCATGGAAAACGGTGTAACAAGGGTGAACACTATCCCATATCACCAGCTCACCGTCTTTCATTGCCATACGAAATTCCGGATGAGCATTCATCAGGCGGG<br>CAAGAATGTGAATAAAGGCCGGATAAAACTTGTGCTTATTTTTCTTTACGGTCTTTAAAAAGGCCGTAATATCCAGCTGAACGGTCTGGTTATAGGTACATTGAG<br>CAACTGACTGAAATGCCTCAAAATGTTCTTTACGATGCCATTGGGATATATCAACGGTGGTATATCCAGTGATTTTTTTCTCCATTTTAGCTTCCTTAGCTCCTG<br>AAAATCTCGATAACTCAAAAAATACGCCCGGTAGTGATCTTATTTCATTATGGTGAAAGTTGGAACCTCTTACGTGCCGATCAACGTCTCATTTTCGCCAATTTA<br>AATCGTAATTATTGGGGACCCTGTATTTAGAAAAATAAACAAATAGGCATGGTCTAAGAAACCATTATTATCATGACATTAACCTATAAAAATAGAAGTTCCTAT<br>TCCGAAGTTCCTATTCTCTAGAAAGTATAGGAACTTCGGATCCGAATTCGAGCTCCGTCGACACTAGTGCGGCCGCACTCGAGGGCATCAGAGCAGATTGTACTG<br>GAAGACCATACTAACGAATGGCTCATGTCTGATTCGTTACCAATTGACGGAATGAACGTTCATTCCGATAATGCTAGCAGCTGTCACCGGATGTGCTTTCCGGTC<br>TGATGAGTCCGTGAGGACGAAACAGCCTCTACAAATAATTTTGTTTAATACTAGAGAAAGAGGAGAAATACTAGATGGTGAGCAAGGGCGAGGAGCTGTTCACCG<br>GGGTGGTGCCCATCCTGGTCGAGCTGGACGGCGACGTAAACGGCCACAAGTTCAGCGTGTCCGGCGAGGGCGAGGGCGATGCCACCTACGGCAAGCTGACCCTGA<br>AGTTCATCTGCACCACAGGCAAGCTGCCCGTGCCCTGGCCCACCCTCGTGACCACCTTCGGCTACGGCCTGCAATGCTTCGCCCGCTACCCCGACCACATGAAGC<br>TGCACGACTTCTTCAAGTCCGCCATGCCCGAAGGCTACGTCCAGGAGCGCACCATCTTCTTCAAGGACGACGGCAACTACAAGACCCGCGCCGAGGTGAAGTTCG<br>AGGGCGACACCCTGGTGAACCGCATCGAGCTGAAGGGCATCGACTTCAAGGAGGACGGCAACATCCTGGGGCACAAGCTGGAGTACAACTACAACAGCCACAACG<br>TCTATATCATGGCCGACAAGCAGAAGAACGGCATCAAGGTGAACTTCAAGATCCGCCACAACATCGAGGACGGCAGCGTGCAGCTCGCCGACCACTACCAGCAGA<br>ACACCCCAATCGGCGACGGCCCCGTGCTGCTGCCCGACAACCACTACCTTAGCTACCAGTCCGCCCTGAGCAAAGACCCCAACGAGAAGCGCGATCACATGGTCC<br>TGCTGGAGTTCGTGACCGCCGCCGGGATCACTCTCGGCATGGACGAGCTGTACAAGTAAGTGTTATAAACCTGTGTGAGAGTTAAGTTTACATGGGCAAAGTTGA<br>TGACCGGGTCGTCCGTTGCAGACAAAAAAAATGGCGCACAATGTGCGCCATTTTTCACTTCACAGGTACTATTGTTTTGAATTGAAAAGGGCGCTTCGGCGCCCT<br>TTTTGCATTTGTTGACGGCATATATTTGTATATCGAAGCGCCCTGATGGGCGCTTTTTTTATTTAATCGATAACCAGA
YJP_MKC154_LP#2	ACATTTAATAAAAAAAGGGCGGTCGCAAGATCGCCCTTTTTTACGTATGACACAGTGAAAAATGGCGCCCATCGGCGCCATTTTTTTATGGAAGTTCCTATTCTC<br>TAGAAAGTATAGGAACTTCAGACGAGAAACGTTCCGTCCGTCTGGGTCAGTTGCCTAACCTTAACTTTTACGCAGGTTCAGCTTGGAAACACAGAAAAAAGCCCG<br>CACCTGACAGTGCGGGCTTTTTTTTTCGACCAAAGGCTCGGTACCAAATTCCAGAAAAGACACCCGAAAGGGTGTTTTTTCGTTTTGGTCCAGGCGGTAATACGG<br>TTATCCACAGAATCAGGGGATAACGCAGGAAAGAACATGTGAGCAAAAGGCCAGCAAAAGGCCAGGAACCGTAAAAATGATTCGCACGGGCCCATGGCTAATTCC<br>CATGTCAGCCGTTAAGTGTTCCTGTGTCACTCAAAATTGCTTTGAGAGGCTCTAAGGGCTTCTCAGTGCGTTACATCCCTGGCTTGTTGTCCACAACCGTTAAAC<br>CTTAAAAGCTTTAAAAGCCTTATATATTCTTTTTTTTCTTATAAAACTTAAAACCTTAGAGGCTATTTAAGTTGCTGATTTATATTAATTTTATTGTTCAAACAT<br>GAGAGCTTAGTACGTGAAACATGAGAGCTTAGTACGTTAGCCATGAGAGCTTAGTACGTTAGCCATGAGGGTTTAGTTCGTTAAACATGAGAGCTTAGTACGTTA<br>AACATGAGAGCTTAGTACGTGAAACATGAGAGCTTAGTACGTACTATCAACAGGTTGAACTGCTGATCTTCAGATCCTCTACGCCGGACGCATCGTGGCCGGATC<br>TTCTTGTGCTTGGATTCTCACCAATAAAAAACGCCCGGCGGCAACCGAGCGTTCTGAACAAATCCAGATGGAGTTCTGAGGTCATTACTGGATCTATCAACAGGA<br>GTCCAAGCGAGCTTTACGCCCCGCCCTGCCACTCATCGCAGTACTGTTGTAATTCATTAAGCATTCTGCCGACATGGAAGCCATCACAAACGGCATGATGAACCT<br>GAATCGCCAGCGGCATCAGCACCTTGTCGCCTTGCGTATAATATTTGCCCATGGTGAAAACGGGGGCGAAGAAGTTGTCCATATTGGCCACGTTTAAATCAAAAC<br>TGGTGAAACTCACCCAGGGATTGGCTGAGACGAAAAACATATTCTCAATAAACCCTTTAGGGAAATAGGCCAGGTTTTCACCGTAACACGCCACATCTTGCGAAT<br>ATATGTGTAGAAACTGCCGGAAATCGTCGTGGTATTCACTCCAGAGCGATGAAAACGTTTCAGTTTGCTCATGGAAAACGGTGTAACAAGGGTGAACACTATCCC<br>ATATCACCAGCTCACCGTCTTTCATTGCCATACGAAATTCCGGATGAGCATTCATCAGGCGGGCAAGAATGTGAATAAAGGCCGGATAAAACTTGTGCTTATTTT<br>TCTTTACGGTCTTTAAAAAGGCCGTAATATCCAGCTGAACGGTCTGGTTATAGGTACATTGAGCAACTGACTGAAATGCCTCAAAATGTTCTTTACGATGCCATT<br>GGGATATATCAACGGTGGTATATCCAGTGATTTTTTTCTCCATTTTAGCTTCCTTAGCTCCTGAAAATCTCGATAACTCAAAAAATACGCCCGGTAGTGATCTTA<br>TTTCATTATGGTGAAAGTTGGAACCTCTTACGTGCCGATCAACGTCTCATTTTCGCCAATTTAAATCGTAATTATTGGGGACCCTGTATTTAGAAAAATAAACAA<br>ATAGGCATGGTCTAAGAAACCATTATTATCATGACATTAACCTATAAAAATAGAAGTTCCTATTCCGAAGTTCCTATTCTCTAGAAAGTATAGGAACTTCGGATC<br>CGAATTCGAGCTCCGTCGACACTAGTGCGGCCGCACTCGAGGGCATCAGAGCAGATTGTACTGGAAGACCATACTTCGTCACTAGAGGGCGATAGTGACAAACTT<br>GACAACTCATCACTTCCTACGTAGGCTGCTAGCAGCTGTCACCGGATGTGCTTTCCGGTCTGATGAGTCCGTGAGGACGAAACAGCCTCTACAAATAATTTTGTT<br>TAATACTAGAGAAAGAGGAGAAATACTAGATGGTGAGCAAGGGCGAGGAGCTGTTCACCGGGGTGGTGCCCATCCTGGTCGAGCTGGACGGCGACGTAAACGGCC<br>ACAAGTTCAGCGTGTCCGGCGAGGGCGAGGGCGATGCCACCTACGGCAAGCTGACCCTGAAGTTCATCTGCACCACAGGCAAGCTGCCCGTGCCCTGGCCCACCC<br>TCGTGACCACCTTCGGCTACGGCCTGCAATGCTTCGCCCGCTACCCCGACCACATGAAGCTGCACGACTTCTTCAAGTCCGCCATGCCCGAAGGCTACGTCCAGG<br>AGCGCACCATCTTCTTCAAGGACGACGGCAACTACAAGACCCGCGCCGAGGTGAAGTTCGAGGGCGACACCCTGGTGAACCGCATCGAGCTGAAGGGCATCGACT<br>TCAAGGAGGACGGCAACATCCTGGGGCACAAGCTGGAGTACAACTACAACAGCCACAACGTCTATATCATGGCCGACAAGCAGAAGAACGGCATCAAGGTGAACT<br>TCAAGATCCGCCACAACATCGAGGACGGCAGCGTGCAGCTCGCCGACCACTACCAGCAGAACACCCCAATCGGCGACGGCCCCGTGCTGCTGCCCGACAACCACT<br>ACCTTAGCTACCAGTCCGCCCTGAGCAAAGACCCCAACGAGAAGCGCGATCACATGGTCCTGCTGGAGTTCGTGACCGCCGCCGGGATCACTCTCGGCATGGACG<br>AGCTGTACAAGTAAGTGTTATAAACCTGTGTGAGAGTTAAGTTTACATGGGCAAAGTTGATGACCGGGTCGTCCGTTGCAGACAAAAAAAATGGCGCACAATGTG<br>CGCCATTTTTCACTTCACAGGTACTATTGTTTTGAATTGAAAAGGGCGCTTCGGCGCCCTTTTTGCATTTGTTGACGGCATATATTTGTATATCGAAGCGCCCTG<br>ATGGGCGCTTTTTTTATTTAATCGATAACCAGA
YJP_MKC155_LP#2	ACATTTAATAAAAAAAGGGCGGTCGCAAGATCGCCCTTTTTTACGTATGACACAGTGAAAAATGGCGCCCATCGGCGCCATTTTTTTATGGAAGTTCCTATTCTC<br>TAGAAAGTATAGGAACTTCAGACGAGAAACGTTCCGTCCGTCTGGGTCAGTTGCCTAACCTTAACTTTTACGCAGGTTCAGCTTGGAAACACAGAAAAAAGCCCG<br>CACCTGACAGTGCGGGCTTTTTTTTTCGACCAAAGGCTCGGTACCAAATTCCAGAAAAGACACCCGAAAGGGTGTTTTTTCGTTTTGGTCCAGGCGGTAATACGG<br>TTATCCACAGAATCAGGGGATAACGCAGGAAAGAACATGTGAGCAAAAGGCCAGCAAAAGGCCAGGAACCGTAAAAATGATTCGCACGGGCCCATGGCTAATTCC<br>CATGTCAGCCGTTAAGTGTTCCTGTGTCACTCAAAATTGCTTTGAGAGGCTCTAAGGGCTTCTCAGTGCGTTACATCCCTGGCTTGTTGTCCACAACCGTTAAAC<br>CTTAAAAGCTTTAAAAGCCTTATATATTCTTTTTTTTCTTATAAAACTTAAAACCTTAGAGGCTATTTAAGTTGCTGATTTATATTAATTTTATTGTTCAAACAT<br>GAGAGCTTAGTACGTGAAACATGAGAGCTTAGTACGTTAGCCATGAGAGCTTAGTACGTTAGCCATGAGGGTTTAGTTCGTTAAACATGAGAGCTTAGTACGTTA<br>AACATGAGAGCTTAGTACGTGAAACATGAGAGCTTAGTACGTACTATCAACAGGTTGAACTGCTGATCTTCAGATCCTCTACGCCGGACGCATCGTGGCCGGATC<br>TTCTTGTGCTTGGATTCTCACCAATAAAAAACGCCCGGCGGCAACCGAGCGTTCTGAACAAATCCAGATGGAGTTCTGAGGTCATTACTGGATCTATCAACAGGA<br>GTCCAAGCGAGCTTTACGCCCCGCCCTGCCACTCATCGCAGTACTGTTGTAATTCATTAAGCATTCTGCCGACATGGAAGCCATCACAAACGGCATGATGAACCT<br>GAATCGCCAGCGGCATCAGCACCTTGTCGCCTTGCGTATAATATTTGCCCATGGTGAAAACGGGGGCGAAGAAGTTGTCCATATTGGCCACGTTTAAATCAAAAC<br>TGGTGAAACTCACCCAGGGATTGGCTGAGACGAAAAACATATTCTCAATAAACCCTTTAGGGAAATAGGCCAGGTTTTCACCGTAACACGCCACATCTTGCGAAT<br>ATATGTGTAGAAACTGCCGGAAATCGTCGTGGTATTCACTCCAGAGCGATGAAAACGTTTCAGTTTGCTCATGGAAAACGGTGTAACAAGGGTGAACACTATCCC<br>ATATCACCAGCTCACCGTCTTTCATTGCCATACGAAATTCCGGATGAGCATTCATCAGGCGGGCAAGAATGTGAATAAAGGCCGGATAAAACTTGTGCTTATTTT<br>TCTTTACGGTCTTTAAAAAGGCCGTAATATCCAGCTGAACGGTCTGGTTATAGGTACATTGAGCAACTGACTGAAATGCCTCAAAATGTTCTTTACGATGCCATT<br>GGGATATATCAACGGTGGTATATCCAGTGATTTTTTTCTCCATTTTAGCTTCCTTAGCTCCTGAAAATCTCGATAACTCAAAAAATACGCCCGGTAGTGATCTTA<br>TTTCATTATGGTGAAAGTTGGAACCTCTTACGTGCCGATCAACGTCTCATTTTCGCCAATTTAAATCGTAATTATTGGGGACCCTGTATTTAGAAAAATAAACAA<br>ATAGGCATGGTCTAAGAAACCATTATTATCATGACATTAACCTATAAAAATAGAAGTTCCTATTCCGAAGTTCCTATTCTCTAGAAAGTATAGGAACTTCGGATC<br>CGAATTCGAGCTCCGTCGACACTAGTGCGGCCGCACTCGAGGGCATCAGAGCAGATTGTACTGGAAGACCATACTAGCGCGGTGAGAGGGATTCGTTACCAATA<br>GACAATTGATTGGACGTTCAATATAATGCTAGCAGCTGTCACCGGATGTGCTTTCCGGTCTGATGAGTCCGTGAGGACGAAACAGCCTCTACAAATAATTTTGTT<br>TAATACTAGAGAAAGAGGAGAAATACTAGATGGTGAGCAAGGGCGAGGAGCTGTTCACCGGGGTGGTGCCCATCCTGGTCGAGCTGGACGGCGACGTAAACGGCC

	ACAAGTTCAGCGTGTCCGGCGAGGGCGAGGGCGATGCCACCTACGGCAAGCTGACCCTGAAGTTCATCTGCACCACAGGCAAGCTGCCCGTGCCCTGGCCCACCC<br>TCGTGACCACCTTCGGCTACGGCCTGCAATGCTTCGCCCGCTACCCCGACCACATGAAGCTGCACGACTTCTTCAAGTCCGCCATGCCCGAAGGCTACGTCCAGG<br>AGCGCACCATCTTCTTCAAGGACGACGGCAACTACAAGACCCGCGCCGAGGTGAAGTTCGAGGGCGACACCCTGGTGAACCGCATCGAGCTGAAGGGCATCGACT<br>TCAAGGAGGACGGCAACATCCTGGGGCACAAGCTGGAGTACAACTACAACAGCCACAACGTCTATATCATGGCCGACAAGCAGAAGAACGGCATCAAGGTGAACT<br>TCAAGATCCGCCACAACATCGAGGACGGCAGCGTGCAGCTCGCCGACCACTACCAGCAGAACACCCCAATCGGCGACGGCCCCGTGCTGCTGCCCGACAACCACT<br>ACCTTAGCTACCAGTCCGCCCTGAGCAAAGACCCCAACGAGAAGCGCGATCACATGGTCCTGCTGGAGTTCGTGACCGCCGCCGGGATCACTCTCGGCATGGACG<br>AGCTGTACAAGTAAGTGTTATAAACCTGTGTGAGAGTTAAGTTTACATGGGCAAAGTTGATGACCGGGTCGTCCGTTGCAGACAAAAAAAATGGCGCACAATGTG<br>CGCCATTTTTCACTTCACAGGTACTATTGTTTTGAATTGAAAAGGGCGCTTCGGCGCCCTTTTTGCATTTGTTGACGGCATATATTTGTATATCGAAGCGCCCTG<br>ATGGGCGCTTTTTTTATTTAATCGATAACCAGA
YJP_MKC156_LP#2	ACATTTAATAAAAAAAGGGCGGTCGCAAGATCGCCCTTTTTTACGTATGACACAGTGAAAAATGGCGCCCATCGGCGCCATTTTTTTATGGAAGTTCCTATTCTC<br>TAGAAAGTATAGGAACTTCAGACGAGAAACGTTCCGTCCGTCTGGGTCAGTTGCCTAACCTTAACTTTTACGCAGGTTCAGCTTGGAAACACAGAAAAAAGCCCG<br>CACCTGACAGTGCGGGCTTTTTTTTTCGACCAAAGGCTCGGTACCAAATTCCAGAAAAGACACCCGAAAGGGTGTTTTTTCGTTTTGGTCCAGGCGGTAATACGG<br>TTATCCACAGAATCAGGGGATAACGCAGGAAAGAACATGTGAGCAAAAGGCCAGCAAAAGGCCAGGAACCGTAAAAATGATTCGCACGGGCCCATGGCTAATTAC<br>CATGTCGGTAATGACTCCAACTTATTGATAGTGTTTTATGTTCAGATAATGCCCGATGACTTTGTCATGCAGCTCCACCGATTTTGAGAACGACAGCGACTTCCG<br>TCCCAGCCGTGCCAGGTGCTGCCTCAGATTCAGGTTATGCCGCTCAATTCGCTGCGTATATCGCTTGCTGATTACGTGCAGCTTTCCCTTCAGGCGGGATTCATA<br>CAGCGGCCAGCCATCCGTCATCCATATCACCACGTCAAAGGGTGACAGCAGGCTCATAAGACGCCCCAGCGTCGCCATAGTGCGTTCACCGAATACGTGCGCAAC<br>AAACGTCTTCCGGAGACTGTCATACGCGTAAAACAGCCAGCGCTGGCGCGATTTAGCCCCGACATAGCCCCACTGTTCGTCCATTTCCGCGCAGACGATGACGTC<br>ACTGCCCGGCTGTATGCGCGAGGTTACCGACTGCGGCCTGAGTTTTTTAAGTGACGTAAAATCGTGTTGAGGCCAACGCCCATAATGCGGGCTGTTGCCCGGCAT<br>CCAACGCCATTCATGGCCATATCAATGATTTTCTGGTGCGTACCGGGTTGAGAAGCGGTGTAAGTGAACTGCAGTTGCCATGTTTTACGGCAGTGAGAGCAGAGA<br>TAGCGCTGATGTCCGGCGGTGCTTTTGCCGTTACGCACCACCCGTCAGTAGCTGAACAGGAGGGACAGCTGATAGAAACAGAAGCCACTGGAGCACCTCAAAAA<br>CACCATCATACACTAAATCAGTAAGTTGGCAGCATCACCTCCCATGTCAGCCGTTAAGTGTTCCTGTGTCACTCAAAATTGCTTTGAGAGGCTCTAAGGGCTTCT<br>CAGTGCGTTACATCCCTGGCTTGTTGTCCACAACCGTTAAACCTTAAAAGCTTTAAAAGCCTTATATATTCTTTTTTTTCTTATAAAACTTAAAACCTTAGAGGC<br>TATTTAAGTTGCTGATTTATATTAATTTTATTGTTCAAACATGAGAGCTTAGTACGTGAAACATGAGAGCTTAGTACGTTAGCCATGAGAGCTTAGTACGTTAGC<br>CATGAGGGTTTAGTTCGTTAAACATGAGAGCTTAGTACGTTAAACATGAGAGCTTAGTACGTGAAACATGAGAGCTTAGTACGTACTATCAACAGGTTGAACTGC<br>TGATCTTCAGATCCTCTACGCCGGACGCATCGTGGCCGGATCTTCTTGTGCTTGGATTCTCACCAATAAAAAACGCCCGGCGGCAACCGAGCGTTCTGAACAAAT<br>CCAGATGGAGTTCTGAGGTCATTACTGGATCTATCAACAGGAGTCCAAGCGAGCTTTACGCCCCGCCCTGCCACTCATCGCAGTACTGTTGTAATTCATTAAGCA<br>TTCTGCCGACATGGAAGCCATCACAAACGGCATGATGAACCTGAATCGCCAGCGGCATCAGCACCTTGTCGCCTTGCGTATAATATTTGCCCATGGTGAAAACGG<br>GGGCGAAGAAGTTGTCCATATTGGCCACGTTTAAATCAAAACTGGTGAAACTCACCCAGGGATTGGCTGAGACGAAAAACATATTCTCAATAAACCCTTTAGGGA<br>AATAGGCCAGGTTTTCACCGTAACACGCCACATCTTGCGAATATATGTGTAGAAACTGCCGGAAATCGTCGTGGTATTCACTCCAGAGCGATGAAAACGTTTCAG<br>TTTGCTCATGGAAAACGGTGTAACAAGGGTGAACACTATCCCATATCACCAGCTCACCGTCTTTCATTGCCATACGAAATTCCGGATGAGCATTCATCAGGCGGG<br>CAAGAATGTGAATAAAGGCCGGATAAAACTTGTGCTTATTTTTCTTTACGGTCTTTAAAAAGGCCGTAATATCCAGCTGAACGGTCTGGTTATAGGTACATTGAG<br>CAACTGACTGAAATGCCTCAAAATGTTCTTTACGATGCCATTGGGATATATCAACGGTGGTATATCCAGTGATTTTTTTCTCCATTTTAGCTTCCTTAGCTCCTG<br>AAAATCTCGATAACTCAAAAAATACGCCCGGTAGTGATCTTATTTCATTATGGTGAAAGTTGGAACCTCTTACGTGCCGATCAACGTCTCATTTTCGCCAATTTA<br>AATCGTAATTATTGGGGACCCTGTATTTAGAAAAATAAACAAATAGGCATGGTCTAAGAAACCATTATTATCATGACATTAACCTATAAAAATAGAAGTTCCTAT<br>TCCGAAGTTCCTATTCTCTAGAAAGTATAGGAACTTCGGATCCGAATTCGAGCTCCGTCGACACTAGTGCGGCCGCACTCGAGGGCATCAGAGCAGATTGTACTG<br>GAAGACCATACTCGACGTACGGTGGAATCTGATTCGTTACCAATTGACATGATACGAAACGTACCGTATCGTTAAGGTAGCTGTCACCGGATGTGCTTTCCGGTC<br>TGATGAGTCCGTGAGGACGAAACAGCCTCTACAAATAATTTTGTTTAATACTAGAGAAAGAGGAGAAATACTAGATGGTGAGCAAGGGCGAGGAGCTGTTCACCG<br>GGGTGGTGCCCATCCTGGTCGAGCTGGACGGCGACGTAAACGGCCACAAGTTCAGCGTGTCCGGCGAGGGCGAGGGCGATGCCACCTACGGCAAGCTGACCCTGA<br>AGTTCATCTGCACCACAGGCAAGCTGCCCGTGCCCTGGCCCACCCTCGTGACCACCTTCGGCTACGGCCTGCAATGCTTCGCCCGCTACCCCGACCACATGAAGC<br>TGCACGACTTCTTCAAGTCCGCCATGCCCGAAGGCTACGTCCAGGAGCGCACCATCTTCTTCAAGGACGACGGCAACTACAAGACCCGCGCCGAGGTGAAGTTCG<br>AGGGCGACACCCTGGTGAACCGCATCGAGCTGAAGGGCATCGACTTCAAGGAGGACGGCAACATCCTGGGGCACAAGCTGGAGTACAACTACAACAGCCACAACG<br>TCTATATCATGGCCGACAAGCAGAAGAACGGCATCAAGGTGAACTTCAAGATCCGCCACAACATCGAGGACGGCAGCGTGCAGCTCGCCGACCACTACCAGCAGA<br>ACACCCCAATCGGCGACGGCCCCGTGCTGCTGCCCGACAACCACTACCTTAGCTACCAGTCCGCCCTGAGCAAAGACCCCAACGAGAAGCGCGATCACATGGTCC<br>TGCTGGAGTTCGTGACCGCCGCCGGGATCACTCTCGGCATGGACGAGCTGTACAAGTAAGTGTTATAAACCTGTGTGAGAGTTAAGTTTACATGGGCAAAGTTGA<br>TGACCGGGTCGTCCGTTGCAGACAAAAAAAATGGCGCACAATGTGCGCCATTTTTCACTTCACAGGTACTATTGTTTTGAATTGAAAAGGGCGCTTCGGCGCCCT<br>TTTTGCATTTGTTGACGGCATATATTTGTATATCGAAGCGCCCTGATGGGCGCTTTTTTTATTTAATCGATAACCAGA
YJP_MKC258_LP#1	AAAAAAAACCCCGCCCTGTCAGGGGCGGGGTTTTTTTTTTTCTTTTGGGTATAGCGTCGTGGACAGTCATTCATCTTTCTGCCCCTCCAAAAGCAAAAACCCGC<br>CGAAGCGGGTTTTTACGTAAATCAGGTGAAACTGACCGATAAGCCGGGACGAGCTGAGCAGTATGTCGACGGTCCGGCGAGAATCTCGCGTAGACACATACATGA<br>GCAGTAGGACCAAAACGAAAAAGGCCCCCCTTTCGGGAGGCCTCTTTTCTGGAATTTGGTACCGAGTGCAGACGTAAAAAAAGCGGCGTGGTTAGCCGCTTTTT<br>TAATTGCCGGATTAACGCTGTGTACCCGGACAAACACCATTAATCAGCAGGAAGGTAAATTCTTCAATATCCTGTTCAACGGTCAGCTGTTCGGTCAGCAGGCGA<br>TACCAACAAAAACCAAAAATCATATCCAGCAGCAGTTCACGATTGGTATCTTTCGGCAGTTCACCATTGCTAATGGCATTTTCAACCAGTTTTTCGGCATCTCA<br>CGACGACGTTCCATAAACTGATCTTTCAGCTGGGTCAGGGTTGCAGGGTCCAGCTGTGCTTCTGCAATAACACAACGAAATGCTTCACCACAAATGGTTTCACGC<br>CAAACTTTCCACAGATTACGCAGCAGAAAATCCAGATCGGCTTTAAAGCTACCCAGATCCGGAAATTTACGCACCTGTTCGCTTTCATTTTCATACACTTCGGCA<br>ATCAGTGCTGCTTTATTGGTCCACCAACGATAAATGGTCGGTTTGCTTGCACCGGCACGACGTGCAACGCTTTCAATGCTCAGACCGCTATAACCACATTCTTTC<br>AGGATTTCAATGGTGCTGGTCAGAATTGCTTTATGGGTATGCGGACTACGCAGGCTACCAATGCTGCTACGGCTCGGGGTACGTGCCATCTAGTATTTCTCCCTT<br>TCAAACATAGTCCATAGTTAAACAAAATTATTTGTAGAGGCGGTTTCGACATCACTGTCTCATCAGAACGCAAAGCACATGCGTTGACCGCTGTGCTCATTATAT<br>CTATCACTGATAGGGATGTCAATCTCTATCACTGATAGGGACAGCCAACGATCGTTCGGGTATGGAGAAAAATATAGAGAGTTGCGATAAAAAGCGTCAGGTAGG<br>ATCCGCTAATCTTATGGATAAAAATGCTACAGCCAACGATCGTTCTCCCAGTACAATCTGCTCTGATGCCCTCGAGTGCGGCCGCACTAGTGTCGACGGAGCTCG<br>AATTCGGATCCGAAGTTCCTATACTTTCTAGAGAATAGGAACTTCGGACCAAAACGAAAAAACACCCTTTCGGGTGTCTTTTCTGGAATTTGGTACCGAGCCTTT<br>GGTCGAAAAAAAAAGCCCGCACTGTCAGGTGCGGGCTTTTTTCTGTGTTTCC
YJP_MKC258_LP#2<br>(Same as<br>YJP_MKC260_LP#2,<br>YJP_MKC261_LP#2)	ACATTTAATAAAAAAAGGGCGGTCGCAAGATCGCCCTTTTTTACGTATGACACAGTGAAAAATGGCGCCCATCGGCGCCATTTTTTTATGGAAGTTCCTATTCTC<br>TAGAAAGTATAGGAACTTCGGATCCGAATTCGAGCTCCGTCGACACTAGTGCGGCCGCACTCGAGGGCATCAGAGCAGATTGTACTGGAAGACCATACTCGACGT<br>ACGGTGGAATCTGATTCGTTACCAATTGACATGATACGAAACGTACCGTATCGTTAAGGTAGCTGTCACCGGATGTGCTTTCCGGTCTGATGAGTCCGTGAGGAC<br>GAAACAGCCTCTACAAATAATTTTGTTTAATACTAGAGAAAGAGGAGAAATACTAGATGGTGAGCAAGGGCGAGGAGCTGTTCACCGGGGTGGTGCCCATCCTGG<br>TCGAGCTGGACGGCGACGTAAACGGCCACAAGTTCAGCGTGTCCGGCGAGGGCGAGGGCGATGCCACCTACGGCAAGCTGACCCTGAAGTTCATCTGCACCACAG<br>GCAAGCTGCCCGTGCCCTGGCCCACCCTCGTGACCACCTTCGGCTACGGCCTGCAATGCTTCGCCCGCTACCCCGACCACATGAAGCTGCACGACTTCTTCAAGT<br>CCGCCATGCCCGAAGGCTACGTCCAGGAGCGCACCATCTTCTTCAAGGACGACGGCAACTACAAGACCCGCGCCGAGGTGAAGTTCGAGGGCGACACCCTGGTGA<br>ACCGCATCGAGCTGAAGGGCATCGACTTCAAGGAGGACGGCAACATCCTGGGGCACAAGCTGGAGTACAACTACAACAGCCACAACGTCTATATCATGGCCGACA<br>AGCAGAAGAACGGCATCAAGGTGAACTTCAAGATCCGCCACAACATCGAGGACGGCAGCGTGCAGCTCGCCGACCACTACCAGCAGAACACCCCAATCGGCGACG<br>GCCCCGTGCTGCTGCCCGACAACCACTACCTTAGCTACCAGTCCGCCCTGAGCAAAGACCCCAACGAGAAGCGCGATCACATGGTCCTGCTGGAGTTCGTGACCG<br>CCGCCGGGATCACTCTCGGCATGGACGAGCTGTACAAGTAAGTGTTATAAACCTGTGTGAGAGTTAAGTTTACATGGGCAAAGTTGATGACCGGGTCGTCCGTTG<br>CAGACAAAAAAAATGGCGCACAATGTGCGCCATTTTTCACTTCACAGGTACTATTGTTTTGAATTGAAAAGGGCGCTTCGGCGCCCTTTTTGCATTTGTTGACGG<br>CATATATTTGTATATCGAAGCGCCCTGATGGGCGCTTTTTTTATTTAATCGATAACCAGA
YJP_MKC259_LP#1	AAAAAAAACCCCGCCCTGTCAGGGGCGGGGTTTTTTTTTTTCTTTTGGGTATAGCGTCGTGGACAGTCATTCATCTTTCTGCCCCTCCAAAAGCAAAAACCCGC<br>CGAAGCGGGTTTTTACGTAAATCAGGTGAAACTGACCGATAAGCCGGGACGAGCTGAGCAGTATGTCGACGGTCCGGCGAGAATCTCGCGTAGACACATACATGA<br>GCAGTAGGACCAAAACGAAAAAGGCCCCCCTTTCGGGAGGCCTCTTTTCTGGAATTTGGTACCGAGTGCAGACGTAAAAAAAGCGGCGTGGTTAGCCGCTTTTT<br>TAATTGCCGGATTAACGCTGTGTACCCGGACAAACACCATTAATCAGCAGGAAGGTAAATTCTTCAATATCCTGTTCAACGGTCAGCTGTTCGGTCAGCAGGCGA<br>TACCAACAAAAACCAAAAATCATATCCAGCAGCAGTTCACGATTGGTATCTTTCGGCAGTTCACCATTGCTAATGGCATTTTCAACCAGTTTTTCGGCATCTCA<br>CGACGACGTTCCATAAACTGATCTTTCAGCTGGGTCAGGGTTGCAGGGTCCAGCTGTGCTTCTGCAATAACACAACGAAATGCTTCACCACAAATGGTTTCACGC<br>CAAACTTTCCACAGATTACGCAGCAGAAAATCCAGATCGGCTTTAAAGCTACCCAGATCCGGAAATTTACGCACCTGTTCGCTTTCATTTTCATACACTTCGGCA<br>ATCAGTGCTGCTTTATTGGTCCACCAACGATAAATGGTCGGTTTGCTTGCACCGGCACGACGTGCAACGCTTTCAATGCTCAGACCGCTATAACCACATTCTTTC<br>AGGATTTCAATGGTGCTGGTCAGAATTGCTTTATGGGTATGCGGACTACGCAGGCTACCAATGCTGCTACGGCTCGGGGTACGTGCCATCTAGTATTTCTCCCTT<br>TCAAACATAGTCCATAGTTAAACAAAATTATTTGTAGAGGCGGTTTCGACATCACTGTCTCATCAGAACGCAAAGCACATGCGTTGACCGCTCGGGTATGGAGAA<br>AAATATAGAGAGTTGCGATAAAAAGCGTCAGGTAGGATCCGCTAATCTTATGGATAAAAATGCTACAGCCAACGATCGTTCTCCCAGTACAATCTGCTCTGATGC<br>CCTCGAGTGCGGCCGCACTAGTGTCGACGGAGCTCGAATTCGGATCCGAAGTTCCTATACTTTCTAGAGAATAGGAACTTCGGACCAAAACGAAAAAACACCCTT<br>TCGGGTGTCTTTTCTGGAATTTGGTACCGAGCCTTTGGTCGAAAAAAAAAGCCCGCACTGTCAGGTGCGGGCTTTTTTCTGTGTTTCC
YJP_MKC259_LP#2	ACATTTAATAAAAAAAGGGCGGTCGCAAGATCGCCCTTTTTTACGTATGACACAGTGAAAAATGGCGCCCATCGGCGCCATTTTTTTATGGAAGTTCCTATTCTC<br>TAGAAAGTATAGGAACTTCGGATCCGAATTCGAGCTCCGTCGACACTAGTGCGGCCGCACTCGAGGGCATCAGAGCAGATTGGGAGCGCAACCACTCTCGCTCCC<br>TATCAGTGATAGAGATTGACATCCCTATCAGTGATAGATATAATGAGCACAGCGGTCAACGCATGTGCTTTGCGTTCTGATGAGACAGTGATGTCGAAACCGCCT<br>CTACAAATAATTTTGTTTAACTATGGACTATGTTTGAAAGGGAGAAATACTAGATGGCACGTACCCCGAGCCGTAGCAGCATTGGTAGCCTGCGTAGTCCGCATA<br>CCCATAAAGCAATTCTGACCAGCACCATTGAAATCCTGAAAGAATGTGGTTATAGCGGTCTGAGCATTGAAAGCGTTGCACGTCGTGCCGGTGCAAGCAAACCGA<br>CCATTTATCGTTGGTGGACCAATAAAGCAGCACTGATTGCCGAAGTGTATGAAAATGAAAGCGAACAGGTGCGTAAATTTCCGGATCTGGGTAGCTTTAAAGCCG<br>ATCTGGATTTTCTGCTGCGTAATCTGTGGAAAGTTTGGCGTGAAACCATTTGTGGTGAAGCATTTCGTTGTGTTATTGCAGAAGCACAGCTGGACCCTGCAACCC<br>TGACCCAGCTGAAAGATCAGTTTATGGAACGTCGTCGTGAGATGCCGAAAAAACTGGTTGAAAATGCCATTAGCAATGGTGAACTGCCGAAAGATACCAATCGTG

	AACTGCTGCTGGATATGATTTTTGGTTTTTGTTGGTATCGCCTGCTGACCGAACAGCTGACCGTTGAACAGGATATTGAAGAATTTACCTTCCTGCTGATTAATG<br>GTGTTTGTCCGGGTACACAGCGTTAATCCGGCAATTAAAAAAGCGGCTAACCACGCCGCTTTTTTTACGTCTGCACTCGGTACCAAATTCCAGAAAAGAGGCCTC<br>CCGAAAGGGGGGCCTTTTTTCGTTTTGGTCCTACTCGACGTACGGTGGAATCTGATTCGTTACCAATTGACATGATACGAAACGTACCGTATCGTTAAGGTAGCT<br>GTCACCGGATGTGCTTTCCGGTCTGATGAGTCCGTGAGGACGAAACAGCCTCTACAAATAATTTTGTTTAATACTAGAGAAAGAGGAGAAATACTAGATGGTGAG<br>CAAGGGCGAGGAGCTGTTCACCGGGGTGGTGCCCATCCTGGTCGAGCTGGACGGCGACGTAAACGGCCACAAGTTCAGCGTGTCCGGCGAGGGCGAGGGCGATGC<br>CACCTACGGCAAGCTGACCCTGAAGTTCATCTGCACCACAGGCAAGCTGCCCGTGCCCTGGCCCACCCTCGTGACCACCTTCGGCTACGGCCTGCAATGCTTCGC<br>CCGCTACCCCGACCACATGAAGCTGCACGACTTCTTCAAGTCCGCCATGCCCGAAGGCTACGTCCAGGAGCGCACCATCTTCTTCAAGGACGACGGCAACTACAA<br>GACCCGCGCCGAGGTGAAGTTCGAGGGCGACACCCTGGTGAACCGCATCGAGCTGAAGGGCATCGACTTCAAGGAGGACGGCAACATCCTGGGGCACAAGCTGGA<br>GTACAACTACAACAGCCACAACGTCTATATCATGGCCGACAAGCAGAAGAACGGCATCAAGGTGAACTTCAAGATCCGCCACAACATCGAGGACGGCAGCGTGCA<br>GCTCGCCGACCACTACCAGCAGAACACCCCAATCGGCGACGGCCCCGTGCTGCTGCCCGACAACCACTACCTTAGCTACCAGTCCGCCCTGAGCAAAGACCCCAA<br>CGAGAAGCGCGATCACATGGTCCTGCTGGAGTTCGTGACCGCCGCCGGGATCACTCTCGGCATGGACGAGCTGTACAAGTAAAATGGTGTTATAAACCTGTGTGA<br>GAGTTAAGTTTACATGGCAAAGTTGATGACCGGGTCGTCCGTTGCAGACAAAAAAAATGGCGCACAATGTGCGCCATTTTTCACTTCACAGGTACTATTGTTTT<br>GAATTGAAAAGGGCGCTTCGGCGCCCTTTTTGCATTTGTTGACGGCATATATTTGTATATCGAAGCGCCCTGATGGGCGCTTTTTTTATTTAATCGATAACCAGA
YJP_MKC260_LP#1	AAAAAAAACCCCGCCCTGTCAGGGGCGGGGTTTTTTTTTTTTCTTTTGGGTATAGCGTCGTGGACAGTCATTCATCTTTCTGCCCCTCCAAAAGCAAAAACCCGC<br>CGAAGCGGGTTTTTACGTAAATCAGGTGAAACTGACCGATAAGCCGGGACGAGCTGAGCAGTATGTCGACGGTCCGGCGAGAATCTCGCGTAGACACATACATGA<br>GCACCTGTCAGAGGAGCCAAATTCAAAAAAGCCTGCTTTCTAGCAGGCTTTTTGCTTTCTAATGGAAGCATAAAAAAATGGCGCCGATGGGCGCCATTTTTCACT<br>GCGGCAAGAATTACTTCAGACAGAGTATCAAAAGGCGAAACCTCCGCAATGCGGAGGTTTCTTTTTAAAGATTATTTGCTCAGGCCATTCAGAAAGATCTGGCTG<br>AATTTGTTCATCAGTTTAATGCGTTCGTTGATATTCTGCTCATGGGTAAAGGTAACAATGCCATTAACGGCATTTGCTGCAATTTTGCTCACGGCATTAACATCA<br>TTAATGCACCATTCGCCGTTCAGATTACCTTCTTTAAAGATCACGTGGTAGGCATCGATGTATTTGTTTTCCAGTTTGTTCATTTTCTCGTTGATGCTGTTGGTT<br>TTATAGTACTCGGTATAAAACTCGATGATGGCATTCTGCAGCGGATAGTAATATTCGGTGGTCAGGCTCAGTTCATTATACAGATAGAATTTCTCGCGGTTGGTT<br>TTGCATTTGATTTGTTCTTTTTTCCACTGCTCTTGCCATTTGCTTTCTTCGATGTTCAGGATTTCCAGAAACAGGTTCTCTTTGGTTTTAAAGTGATAATACAGA<br>TTGCCTTTGCTGCTTTCGCTCAGTTTAACAATTTCACCGGTGGTGGTTGCGTTATAGCCATTTTTGATAAACAGTTCTTTGGCAACGCCCAGAATTTTATCTTTC<br>AGGTTCATCTAGTAGTTGTCCTCTATCAAAAGCCAATGGCATGGCTTAAACAAAATTATTTGTAGAGGTCGTTTCGGCATCACTGCCTCATCAGAAGTGCAAGCA<br>CAGCACTTGACGACTAGCGTTTTCAAGTTCGTGGAAAGCAGAATGGTTCAGGCGAGCCGGATCGGAAATTCCCTCAGATAGGCCCCCCCAGATGAGGGGGATCAG<br>AGCATGTCGCGCAAAAGTGTGCAGCGGTTTTGCGATAACGACATGCGCCAAAACAAAGACTGAAAGCGCGAGGAGCGAATCTGAAAGATCGCGACCCGCTTTAGA<br>GCGCCGTGCGTCCGTTTGGACGCACAAAGGGACTTTTGTGCTACCACATTGCGGAGAAAAGGTCCGAAAATTCGGACCCGATGGAATTGGATAAAAAAAGGCCCC<br>TGTTGAAATTGCAGGGGCCTGGTACGAGCATTATTTTGCATCTGCCTGTTTAATCAGTTCCAGGGTTTTTTCAACACGATCTGCAGGCAGCGGTGCACCCAGAAC<br>TGCCAGGCTTGCATCTGCCAGCATAATTGCGGTTTCCGGCAGGCTATCTGCGCTCAGCGGACTCGGAATTTTACCATCATTGCGACGCATTTCAATAACGCTCAT<br>GGTAATATGAAACGGCAGTTCTGCACGCGGATCATCACCAACAATTTCGGTTGCCAGATCACGAAAAACATTGGTCAGTGCTTCACGCTGGCTATGATATTCTGC<br>AAATTCTTCGCTACCAACAATCGGCAGCTGATACAGACGACCAACATTCCATTTGGTGCTCAGCAGCAGACGAACTTCGCTTGCAACAATTGCCCACAGACGCAT<br>TTCCGGACCTGCATCCAGGGTGCTCAGATCTTCTGCCAGAACGGTGCTCGGTTCAACGGTGCTTTTCAGCAGGGTCAGAAAGATTTCGGTTTTGCTCGGAAAATG<br>ATAATACAGGCTTGCCTGACGAATACCAACTGCATCTGCAATCTGATGGGTACTGGTGGTTGCAAAACCCTGACGGGTAAACAGTTCTGCGCTTGCATCCAGAAT<br>TTCTTCACGCGGATTTTTACCTGCACGACGCGGTGCACTACGACGCGGACGACCAACTGCGCCTGCCATCTAGTAACCTCTTTGCTGATTATTAGGGAACATTTT<br>AAACAAAATTATTTGTAGAGGGGTGTTTCGTCCTTTCGGACTCATCAGTCAAGGTACGCACCTTGAGACACCCTGTGCTCATTATATCTATCACTGATAGGGATG<br>TCAATCTCTATCACTGATAGGGAGCGAGAGTGGTTGCGAGTAGGACCAAAACGAAAAAAGGCCCCCCTTTCGGGAGGCCTCTTTTCTGGAATTTGGTACCGAGTG<br>CAGACGTAAAAAAAGCGGCGTGGTTAGCCGCTTTTTTAATTGCCGGATTAACGCTGTGTACCCGGACAAACACCATTAATCAGCAGGAAGGTAAATTCTTCAATA<br>TCCTGTTCAACGGTCAGCTGTTCGGTCAGCAGGCGATACCAACAAAAACCAAAAATCATATCCAGCAGCAGTTCACGATTGGTATCTTTCGGCAGTTCACCATTG<br>CTAATGGCATTTTCAACCAGTTTTTTCGGCATCTCACGACGACGTTCCATAAACTGATCTTTCAGCTGGGTCAGGGTTGCAGGGTCCAGCTGTGCTTCTGCAATA<br>ACACAACGAAATGCTTCACCACAAATGGTTTCACGCCAAACTTTCCACAGATTACGCAGCAGAAAATCCAGATCGGCTTTAAAGCTACCCAGATCCGGAAATTTA<br>CGCACCTGTTCGCTTTCATTTTCATACACTTCGGCAATCAGTGCTGCTTTATTGGTCCACCAACGATAAATGGTCGGTTTGCTTGCACCGGCACGACGTGCAACG<br>CTTTCAATGCTCAGACCGCTATAACCACATTCTTTCAGGATTTCAATGGTGCTGGTCAGAATTGCTTTATGGGTATGCGGACTACGCAGGCTACCAATGCTGCTA<br>CGGCTCGGGGTACGTGCCATCTAGTATTTCTCCCTTTCAAACATAGTCCATAGTTAAACAAAATTATTTGTAGAGGCGGTTTCGACATCACTGTCTCATCAGAAC<br>GCAAAGCACATGCGTTGACCGCTGCTAGCATTATCTATAGATCGATAGAAACTGTCAAAGGGTAACGAATCATTTGGTTGGACAAGTATAGACCGATCGCACGGT<br>CTATATACTAAAGTAGGACTGAGCTAGCTGTCAATTGGTAACGTATAGCTTCCATACCCTCCCAGTACAATCTGCTCTGATGCCCTCGAGTGCGGCCGCACTAGT<br>GTCGACGGAGCTCGAATTCGGATCCGAAGTTCCTATACTTTCTAGAGAATAGGAACTTCGGACCAAAACGAAAAAACACCCTTTCGGGTGTCTTTTCTGGAATTT<br>GGTACCGAGCCTTTGGTCGAAAAAAAAAGCCCGCACTGTCAGGTGCGGGCTTTTTTCTGTGTTTCC
YJP_MKC261_LP#1	AAAAAAAACCCCGCCCTGTCAGGGGCGGGGTTTTTTTTTTTTCTTTTGGGTATAGCGTCGTGGACAGTCATTCATCTTTCTGCCCCTCCAAAAGCAAAAACCCGC<br>CGAAGCGGGTTTTTACGTAAATCAGGTGAAACTGACCGATAAGCCGGGACGAGCTGAGCAGTATGTCGACGGTCCGGCGAGAATCTCGCGTAGACACATACATGA<br>GCAAGCGTCAGAGGAGCCAAATTCAAAAAAGCCTGCTTTCTAGCAGGCTTTTTGCTTTCTAATGGAAGCATAAAAAAATGGCGCCGATGGGCGCCATTTTTCACT<br>GCGGCAAGAATTACTTCAGACAGAGTATCAAAAGGCGAAACCTCCGCAATGCGGAGGTTTCTTTTTAAAGATTATTTGCTCAGGCCATTCAGAAAGATCTGGCTG<br>AATTTGTTCATCAGTTTAATGCGTTCGTTGATATTCTGCTCATGGGTAAAGGTAACAATGCCATTAACGGCATTTGCTGCAATTTTGCTCACGGCATTAACATCA<br>TTAATGCACCATTCGCCGTTCAGATTACCTTCTTTAAAGATCACGTGGTAGGCATCGATGTATTTGTTTTCCAGTTTGTTCATTTTCTCGTTGATGCTGTTGGTT<br>TTATAGTACTCGGTATAAAACTCGATGATGGCATTCTGCAGCGGATAGTAATATTCGGTGGTCAGGCTCAGTTCATTATACAGATAGAATTTCTCGCGGTTGGTT<br>TTGCATTTGATTTGTTCTTTTTTCCACTGCTCTTGCCATTTGCTTTCTTCGATGTTCAGGATTTCCAGAAACAGGTTCTCTTTGGTTTTAAAGTGATAATACAGA<br>TTGCCTTTGCTGCTTTCGCTCAGTTTAACAATTTCACCGGTGGTGGTTGCGTTATAGCCATTTTTGATAAACAGTTCTTTGGCAACGCCCAGAATTTTATCTTTC<br>AGGTTCATCTAGTAGTTGTCCTCTATCAAAAGCCAATGGCATGGCTTAAACAAAATTATTTGTAGAGGTCGTTTCGGCATCACTGCCTCATCAGAAGTGCAAGCA<br>CAGCACTTGACGACTAATTGTGAGCGCTCACAATTCCACACATTATACGAGCCGATGATTAATTGTCAACACAGCCAACGATCGTTACCTGCGGAGAAAAGGTCC<br>GAAAATTCGGACCCGATGGAATTGGATAAAAAAAGGCCCCTGTTGAAATTGCAGGGGCCTGGTACGAGCATTATTTTGCATCTGCCTGTTTAATCAGTTCCAGGG<br>TTTTTTCAACACGATCTGCAGGCAGCGGTGCACCCAGAACTGCCAGGCTTGCATCTGCCAGCATAATTGCGGTTTCCGGCAGGCTATCTGCGCTCAGCGGACTCG<br>GAATTTTACCATCATTGCGACGCATTTCAATAACGCTCATGGTAATATGAAACGGCAGTTCTGCACGCGGATCATCACCAACAATTTCGGTTGCCAGATCACGAA<br>AAACATTGGTCAGTGCTTCACGCTGGCTATGATATTCTGCAAATTCTTCGCTACCAACAATCGGCAGCTGATACAGACGACCAACATTCCATTTGGTGCTCAGCA<br>GCAGACGAACTTCGCTTGCAACAATTGCCCACAGACGCATTTCCGGACCTGCATCCAGGGTGCTCAGATCTTCTGCCAGAACGGTGCTCGGTTCAACGGTGCTTT<br>TCAGCAGGGTCAGAAAGATTTCGGTTTTGCTCGGAAAATGATAATACAGGCTTGCCTGACGAATACCAACTGCATCTGCAATCTGATGGGTACTGGTGGTTGCAA<br>AACCCTGACGGGTAAACAGTTCTGCGCTTGCATCCAGAATTTCTTCACGCGGATTTTTACCTGCACGACGCGGTGCACTACGACGCGGACGACCAACTGCGCCTG<br>CCATCTAGTAACCTCTTTGCTGATTATTAGGGAACATTTTAAACAAAATTATTTGTAGAGGGGTGTTTCGTCCTTTCGGACTCATCAGTCAAGGTACGCACCTTG<br>AGACACCCTGCTAGCAGCCTACGTAGGAAGTGATGAGTTGTCAAGTTTGTCACTATCGCCCTCTAGTGACGACATTCATAAAAAAATGGCGCCGATGGGCGCCAT<br>TTTTCACTGTGTCATACGTAAAAAAGGGCGATCTTGCGACCGCCCTTTTTTTATTAAATGTTTATTTTGCCAGCGGATTACGCAGACCTGCATCAATCAGATCAA<br>CCAGCTGATCACAACGGGTTGCCAGATTACCAATATCTTCTTGTGCAATCAGAACCGGATGAATAACGCTGGTCAGTGCATGCAGAACGGTTTCTGCCAGAACCG<br>GAATATCGGTTGCAATATACAGACCTGCTTCAACACCATCACGAATAATTTCGGCCAGCAGTTTTGCAATCACGCTTTTGTAATAATCGCCACATTTCATATCCT<br>GTTTGGCGGTCATCAGGATCTGAATAAAAACCCGTATGTGTTTGAAATGATCCTGATGATGCTGTTCCATCAGATTACGTGCCAGATGACGCAGACGATCCAGCG<br>GTGCATGGCTTTTATCCAGCGGACAAATCTGACGTTCAAAAACACCAATCTGACCAAAACCAATTGCATCAACCAGTGCGTTTTTGCTGCTAAAATGTTTAAACA<br>CATTTGCCGGACTCATATTCAGTGCGCTTGCAATATCTGCAATGGCAACTGCATTAAAACCACGTTCACTAAACAGTTCTTCGGCTTTTGCCAGAATATCACGAC<br>GGGTTTCTTCGGCACTATGACGCGGACGACGACGAACCGGCAGATCGCTTTTACGATCACCTTTACGCACCTGATCAATGGTTTTGTTCATCTATTAGCTCCTGT<br>TTGAAACATAGTCCATAGCGTATTAAACAAAATTATTTGTAGAGGGGGTTTCGTCATCACTGACTCATCAGAGTTGAAAGCACATCAACTGACCCCTGTGCTCAT<br>TATATCTATCACTGATAGGGATGTCAATCTCTATCACTGATAGGGACAGCCAACGATCGTTTATAGACCGATCGCACGGTCTATATACTAAAGTAGGACTGAGCT<br>AGCTGTCAATTGGTAACGTATAGCTTCCATACCAGTAGGACCAAAACGAAAAAAGGCCCCCCTTTCGGGAGGCCTCTTTTCTGGAATTTGGTACCGAGTGCAGAC<br>GTAAAAAAAGCGGCGTGGTTAGCCGCTTTTTTAATTGCCGGATTAACGCTGTGTACCCGGACAAACACCATTAATCAGCAGGAAGGTAAATTCTTCAATATCCTG<br>TTCAACGGTCAGCTGTTCGGTCAGCAGGCGATACCAACAAAAACCAAAAATCATATCCAGCAGCAGTTCACGATTGGTATCTTTCGGCAGTTCACCATTGCTAAT<br>GGCATTTTCAACCAGTTTTTTCGGCATCTCACGACGACGTTCCATAAACTGATCTTTCAGCTGGGTCAGGGTTGCAGGGTCCAGCTGTGCTTCTGCAATAACACA<br>ACGAAATGCTTCACCACAAATGGTTTCACGCCAAACTTTCCACAGATTACGCAGCAGAAAATCCAGATCGGCTTTAAAGCTACCCAGATCCGGAAATTTACGCAC<br>CTGTTCGCTTTCATTTTCATACACTTCGGCAATCAGTGCTGCTTTATTGGTCCACCAACGATAAATGGTCGGTTTGCTTGCACCGGCACGACGTGCAACGCTTTC<br>AATGCTCAGACCGCTATAACCACATTCTTTCAGGATTTCAATGGTGCTGGTCAGAATTGCTTTATGGGTATGCGGACTACGCAGGCTACCAATGCTGCTACGGCT<br>CGGGGTACGTGCCATCTAGTATTTCTCCCTTTCAAACATAGTCCATAGTTAAACAAAATTATTTGTAGAGGCGGTTTCGACATCACTGTCTCATCAGAACGCAAA<br>GCACATGCGTTGACCGCTGCTAGCATTATCTATAGATCGATAGAAACTGTCAAAGGGTAACGAATCATTTGGTTGGACAAGAGCGTTTTCAAGTTCGTGGAAAGC<br>AGAATGGTTCAGGCGAGCCGGATCGGAAATTCCCTCAGATAGGCCCCCCCAGATGAGGGGGATCAGAGCATGTCGCGCAAAAGTGTGCAGCGGTTTTGCGATAAC<br>GACATGCGCCAAAACAAAGACTGAAAGCGCGAGGAGCGAATCTGAAAGATCGCGACCCGCTTTAGAGCGCCGTGCGTCCGTTTGGACGCACAAAGGGACTTTTGT<br>GCTACCACTCCCAGTACAATCTGCTCTGATGCCCTCGAGTGCGGCCGCACTAGTGTCGACGGAGCTCGAATTCGGATCCGAAGTTCCTATACTTTCTAGAGAATA<br>GGAACTTCGGACCAAAACGAAAAAACACCCTTTCGGGTGTCTTTTCTGGAATTTGGTACCGAGCCTTTGGTCGAAAAAAAAAGCCCGCACTGTCAGGTGCGGGCT<br>TTTTTCTGTGTTTCC
YJP_MKC262_LP#1	AAAAAAAACCCCGCCCTGTCAGGGGCGGGGTTTTTTTTTTTTCTTTTGGGTATAGCGTCGTGGACAGTCATTCATCTTTCTGCCCCTCCAAAAGCAAAAACCCGC<br>CGAAGCGGGTTTTTACGTAAATCAGGTGAAACTGACCGATAAGCCGGGACGAGCTGAGCAGTATGTCGACGGTCCGGCGAGAATCTCGCGTAGACACATACATGA<br>GCACCTGTCAGAGGAGCCAAATTCAAAAAAGCCTGCTTTCTAGCAGGCTTTTTGCTTTCTAATGGAAGCATAAAAAAATGGCGCCGATGGGCGCCATTTTTCACT<br>GCGGCAAGAATTACTTCAGACAGAGTATCAAAAGGCGAAACCTCCGCAATGCGGAGGTTTCTTTTTAAAGATTATTTGCTCAGGCCATTCAGAAAGATCTGGCTG<br>AATTTGTTCATCAGTTTAATGCGTTCGTTGATATTCTGCTCATGGGTAAAGGTAACAATGCCATTAACGGCATTTGCTGCAATTTTGCTCACGGCATTAACATCA

	TTAATGCACCATTCGCCGTTCAGATTACCTTCTTTAAAGATCACGTGGTAGGCATCGATGTATTTGTTTTCCAGTTTGTTCATTTTCTCGTTGATGCTGTTGGTT<br>TTATAGTACTCGGTATAAAACTCGATGATGGCATTCTGCAGCGGATAGTAATATTCGGTGGTCAGGCTCAGTTCATTATACAGATAGAATTTCTCGCGGTTGGTT<br>TTGCATTTGATTTGTTCTTTTTTCCACTGCTCTTGCCATTTGCTTTCTTCGATGTTCAGGATTTCCAGAAACAGGTTCTCTTTGGTTTTAAAGTGATAATACAGA<br>TTGCCTTTGCTGCTTTCGCTCAGTTTAACAATTTCACCGGTGGTGGTTGCGTTATAGCCATTTTTGATAAACAGTTCTTTGGCAACGCCCAGAATTTTATCTTTC<br>AGGTTCATCTAGTAGTTGTCCTCTATCAAAAGCCAATGGCATGGCTTAAACAAAATTATTTGTAGAGGTCGTTTCGGCATCACTGCCTCATCAGAAGTGCAAGCA<br>CAGCACTTGACGACTGTGCTCATTATATCTATCACTGATAGGGATGTCAATCTCTATCACTGATAGGGAGCGAGAGTGGTTGCGCATTCATAAAAAAATGGCGCC<br>GATGGGCGCCATTTTTCACTGTGTCATACGTAAAAAAGGGCGATCTTGCGACCGCCCTTTTTTTATTAAATGTTTATTTTGCCAGCGGATTACGCAGACCTGCAT<br>CAATCAGATCAACCAGCTGATCACAACGGGTTGCCAGATTACCAATATCTTCTTGTGCAATCAGAACCGGATGAATAACGCTGGTCAGTGCATGCAGAACGGTTT<br>CTGCCAGAACCGGAATATCGGTTGCAATATACAGACCTGCTTCAACACCATCACGAATAATTTCGGCCAGCAGTTTTGCAATCACGCTTTTGTAATAATCGCCAC<br>ATTTCATATCCTGTTTGGCGGTCATCAGGATCTGAATAAAAACCCGTATGTGTTTGAAATGATCCTGATGATGCTGTTCCATCAGATTACGTGCCAGATGACGCA<br>GACGATCCAGCGGTGCATGGCTTTTATCCAGCGGACAAATCTGACGTTCAAAAACACCAATCTGACCAAAACCAATTGCATCAACCAGTGCGTTTTTGCTGCTAA<br>AATGTTTAAACACATTTGCCGGACTCATATTCAGTGCGCTTGCAATATCTGCAATGGCAACTGCATTAAAACCACGTTCACTAAACAGTTCTTCGGCTTTTGCCA<br>GAATATCACGACGGGTTTCTTCGGCACTATGACGCGGACGACGACGAACCGGCAGATCGCTTTTACGATCACCTTTACGCACCTGATCAATGGTTTTGTTCATCT<br>ATTAGCTCCTGTTTGAAACATAGTCCATAGCGTATTAAACAAAATTATTTGTAGAGGGGGTTTCGTCATCACTGACTCATCAGAGTTGAAAGCACATCAACTGAC<br>CCCTAGCGTTTTCAAGTTCGTGGAAAGCAGAATGGTTCAGGCGAGCCGGATCGGAAATTCCCTCAGATAGGCCCCCCCAGATGAGGGGGATCAGAGCATGTCGCG<br>CAAAAGTGTGCAGCGGTTTTGCGATAACGACATGCGCCAAAACAAAGACTGAAAGCGCGAGGAGCGAATCTGAAAGATCGCGACCCGCTTTAGAGCGCCGTGCGT<br>CCGTTTGGACGCACAAAGGGACTTTTGTGCTACCAAGTAGGACCAAAACGAAAAAAGGCCCCCCTTTCGGGAGGCCTCTTTTCTGGAATTTGGTACCGAGTGCAG<br>ACGTAAAAAAAGCGGCGTGGTTAGCCGCTTTTTTAATTGCCGGATTAACGCTGTGTACCCGGACAAACACCATTAATCAGCAGGAAGGTAAATTCTTCAATATCC<br>TGTTCAACGGTCAGCTGTTCGGTCAGCAGGCGATACCAACAAAAACCAAAAATCATATCCAGCAGCAGTTCACGATTGGTATCTTTCGGCAGTTCACCATTGCTA<br>ATGGCATTTTCAACCAGTTTTTTCGGCATCTCACGACGACGTTCCATAAACTGATCTTTCAGCTGGGTCAGGGTTGCAGGGTCCAGCTGTGCTTCTGCAATAACA<br>CAACGAAATGCTTCACCACAAATGGTTTCACGCCAAACTTTCCACAGATTACGCAGCAGAAAATCCAGATCGGCTTTAAAGCTACCCAGATCCGGAAATTTACGC<br>ACCTGTTCGCTTTCATTTTCATACACTTCGGCAATCAGTGCTGCTTTATTGGTCCACCAACGATAAATGGTCGGTTTGCTTGCACCGGCACGACGTGCAACGCTT<br>TCAATGCTCAGACCGCTATAACCACATTCTTTCAGGATTTCAATGGTGCTGGTCAGAATTGCTTTATGGGTATGCGGACTACGCAGGCTACCAATGCTGCTACGG<br>CTCGGGGTACGTGCCATCTAGTATTTCTCCCTTTCAAACATAGTCCATAGTTAAACAAAATTATTTGTAGAGGCGGTTTCGACATCACTGTCTCATCAGAACGCA<br>AAGCACATGCGTTGACCGCTGCTAGCAGCCTACGTAGGAAGTGATGAGTTGTCAAGTTTGTCACTATCGCCCTCTAGTGACGACTCCCAGTACAATCTGCTCTGA<br>TGCCCTCGAGTGCGGCCGCACTAGTGTCGACGGAGCTCGAATTCGGATCCGAAGTTCCTATACTTTCTAGAGAATAGGAACTTCGGACCAAAACGAAAAAACACC<br>CTTTCGGGTGTCTTTTCTGGAATTTGGTACCGAGCCTTTGGTCGAAAAAAAAAGCCCGCACTGTCAGGTGCGGGCTTTTTTCTGTGTTTCC
YJP_MKC262_LP#2	ACATTTAATAAAAAAAGGGCGGTCGCAAGATCGCCCTTTTTTACGTATGACACAGTGAAAAATGGCGCCCATCGGCGCCATTTTTTTATGGAAGTTCCTATTCTC<br>TAGAAAGTATAGGAACTTCGGATCCGAATTCGAGCTCCGTCGACACTAGTGCGGCCGCACTCGAGGGCATCAGAGCAGATTGGGAGCGCAACCACTCTCGCTCCC<br>TATCAGTGATAGAGATTGACATCCCTATCAGTGATAGATATAATGAGCACAGCGGTCAACGCATGTGCTTTGCGTTCTGATGAGACAGTGATGTCGAAACCGCCT<br>CTACAAATAATTTTGTTTAACTATGGACTATGTTTGAAAGGGAGAAATACTAGATGGCACGTACCCCGAGCCGTAGCAGCATTGGTAGCCTGCGTAGTCCGCATA<br>CCCATAAAGCAATTCTGACCAGCACCATTGAAATCCTGAAAGAATGTGGTTATAGCGGTCTGAGCATTGAAAGCGTTGCACGTCGTGCCGGTGCAAGCAAACCGA<br>CCATTTATCGTTGGTGGACCAATAAAGCAGCACTGATTGCCGAAGTGTATGAAAATGAAAGCGAACAGGTGCGTAAATTTCCGGATCTGGGTAGCTTTAAAGCCG<br>ATCTGGATTTTCTGCTGCGTAATCTGTGGAAAGTTTGGCGTGAAACCATTTGTGGTGAAGCATTTCGTTGTGTTATTGCAGAAGCACAGCTGGACCCTGCAACCC<br>TGACCCAGCTGAAAGATCAGTTTATGGAACGTCGTCGTGAGATGCCGAAAAAACTGGTTGAAAATGCCATTAGCAATGGTGAACTGCCGAAAGATACCAATCGTG<br>AACTGCTGCTGGATATGATTTTTGGTTTTTGTTGGTATCGCCTGCTGACCGAACAGCTGACCGTTGAACAGGATATTGAAGAATTTACCTTCCTGCTGATTAATG<br>GTGTTTGTCCGGGTACACAGCGTTAATCCGGCAATTAAAAAAGCGGCTAACCACGCCGCTTTTTTTACGTCTGCACTCGGTACCAAATTCCAGAAAAGAGGCCTC<br>CCGAAAGGGGGGCCTTTTTTCGTTTTGGTCCTACTCGACGTACGGTGGAATCTGATTCGTTACCAATTGACATGATACGAAACGTACCGTATCGTTAAGGTAGCT<br>GTCACCGGATGTGCTTTCCGGTCTGATGAGTCCGTGAGGACGAAACAGCCTCTACAAATAATTTTGTTTAATACTAGAGAAAGAGGAGAAATACTAGATGGTGAG<br>CAAGGGCGAGGAGCTGTTCACCGGGGTGGTGCCCATCCTGGTCGAGCTGGACGGCGACGTAAACGGCCACAAGTTCAGCGTGTCCGGCGAGGGCGAGGGCGATGC<br>CACCTACGGCAAGCTGACCCTGAAGTTCATCTGCACCACAGGCAAGCTGCCCGTGCCCTGGCCCACCCTCGTGACCACCTTCGGCTACGGCCTGCAATGCTTCGC<br>CCGCTACCCCGACCACATGAAGCTGCACGACTTCTTCAAGTCCGCCATGCCCGAAGGCTACGTCCAGGAGCGCACCATCTTCTTCAAGGACGACGGCAACTACAA<br>GACCCGCGCCGAGGTGAAGTTCGAGGGCGACACCCTGGTGAACCGCATCGAGCTGAAGGGCATCGACTTCAAGGAGGACGGCAACATCCTGGGGCACAAGCTGGA<br>GTACAACTACAACAGCCACAACGTCTATATCATGGCCGACAAGCAGAAGAACGGCATCAAGGTGAACTTCAAGATCCGCCACAACATCGAGGACGGCAGCGTGCA<br>GCTCGCCGACCACTACCAGCAGAACACCCCAATCGGCGACGGCCCCGTGCTGCTGCCCGACAACCACTACCTTAGCTACCAGTCCGCCCTGAGCAAAGACCCCAA<br>CGAGAAGCGCGATCACATGGTCCTGCTGGAGTTCGTGACCGCCGCCGGGATCACTCTCGGCATGGACGAGCTGTACAAGTAAAATGGTGTTATAAACCTGTGTGA<br>GAGTTAAGTTTACATGGGCAAAGTTGATGACCGGGTCGTCCGTTGCAGACAAAAAAAATGGCGCACAATGTGCGCCATTTTTCACTTCACAGGTACTATTGTTTT<br>GAATTGAAAAGGGCGCTTCGGCGCCCTTTTTGCATTTGTTGACGGCATATATTTGTATATCGAAGCGCCCTGATGGGCGCTTTTTTTATTTAATCGATAACCAGA
YJP_MKC263_LP#1	AAAAAAAACCCCGCCCTGTCAGGGGCGGGGTTTTTTTTTTTTCTTTTGGGTATAGCGTCGTGGACAGTCATTCATCTTTCTGCCCCTCCAAAAGCAAAAACCCGC<br>CGAAGCGGGTTTTTACGTAAATCAGGTGAAACTGACCGATAAGCCGGGACGAGCTGAGCAGTATGTCGACGGTCCGGCGAGAATCTCGCGTAGACACATACATGA<br>GCAAGCTCAGAGGAGCCAAATTCAAAAAAGCCTGCTTTCTAGCAGGCTTTTTGCTTTCTAATGGAAGCATAAAAAAATGGCGCCGATGGGCGCCATTTTTCACTG<br>CGGCAAGAATTACTTCAGACAGAGTATCAAAAGGCGAAACCTCCGCAATGCGGAGGTTTCTTTTTAAAGATTATTTGCTCAGGCCATTCAGAAAGATCTGGCTGA<br>ATTTGTTCATCAGTTTAATGCGTTCGTTGATATTCTGCTCATGGGTAAAGGTAACAATGCCATTAACGGCATTTGCTGCAATTTTGCTCACGGCATTAACATCAT<br>TAATGCACCATTCGCCGTTCAGATTACCTTCTTTAAAGATCACGTGGTAGGCATCGATGTATTTGTTTTCCAGTTTGTTCATTTTCTCGTTGATGCTGTTGGTTT<br>TATAGTACTCGGTATAAAACTCGATGATGGCATTCTGCAGCGGATAGTAATATTCGGTGGTCAGGCTCAGTTCATTATACAGATAGAATTTCTCGCGGTTGGTTT<br>TGCATTTGATTTGTTCTTTTTTCCACTGCTCTTGCCATTTGCTTTCTTCGATGTTCAGGATTTCCAGAAACAGGTTCTCTTTGGTTTTAAAGTGATAATACAGAT<br>TGCCTTTGCTGCTTTCGCTCAGTTTAACAATTTCACCGGTGGTGGTTGCGTTATAGCCATTTTTGATAAACAGTTCTTTGGCAACGCCCAGAATTTTATCTTTCA<br>GGTTCATCTAGTAGTTGTCCTCTATCAAAAGCCAATGGCATGGCTTAAACAAAATTATTTGTAGAGGTCGTTTCGGCATCACTGCCTCATCAGAAGTGCAAGCAC<br>AGCACTTGACGACTGTGCTCATTATATCTATCACTGATAGGGATGTCAATCTCTATCACTGATAGGGAGCGAGAGTGGTTGCGACCTCATAAAAAAATGGCGCCG<br>ATGGGCGCCATTTTTCACTGTGTCATACGTAAAAAAGGGCGATCTTGCGACCGCCCTTTTTTTATTAAATGTTTATTTTGCCAGCGGATTACGCAGACCTGCATC<br>AATCAGATCAACCAGCTGATCACAACGGGTTGCCAGATTACCAATATCTTCTTGTGCAATCAGAACCGGATGAATAACGCTGGTCAGTGCATGCAGAACGGTTTC<br>TGCCAGAACCGGAATATCGGTTGCAATATACAGACCTGCTTCAACACCATCACGAATAATTTCGGCCAGCAGTTTTGCAATCACGCTTTTGTAATAATCGCCACA<br>TTTCATATCCTGTTTGGCGGTCATCAGGATCTGAATAAAAACCCGTATGTGTTTGAAATGATCCTGATGATGCTGTTCCATCAGATTACGTGCCAGATGACGCAG<br>ACGATCCAGCGGTGCATGGCTTTTATCCAGCGGACAAATCTGACGTTCAAAAACACCAATCTGACCAAAACCAATTGCATCAACCAGTGCGTTTTTGCTGCTAAA<br>ATGTTTAAACACATTTGCCGGACTCATATTCAGTGCGCTTGCAATATCTGCAATGGCAACTGCATTAAAACCACGTTCACTAAACAGTTCTTCGGCTTTTGCCAG<br>AATATCACGACGGGTTTCTTCGGCACTATGACGCGGACGACGACGAACCGGCAGATCGCTTTTACGATCACCTTTACGCACCTGATCAATGGTTTTGTTCATCTA<br>TTAGCTCCTGTTTGAAACATAGTCCATAGCGTATTAAACAAAATTATTTGTAGAGGGGGTTTCGTCATCACTGACTCATCAGAGTTGAAAGCACATCAACTGACC<br>CCTAGCGTTTTCAAGTTCGTGGAAAGCAGAATGGTTCAGGCGAGCCGGATCGGAAATTCCCTCAGATAGGCCCCCCCAGATGAGGGGGATCAGAGCATGTCGCGC<br>AAAAGTGTGCAGCGGTTTTGCGATAACGACATGCGCCAAAACAAAGACTGAAAGCGCGAGGAGCGAATCTGAAAGATCGCGACCCGCTTTAGAGCGCCGTGCGTC<br>CGTTTGGACGCACAAAGGGACTTTTGTGCTACCACATTAAAAGAGGTGGCCAGGGGGATCACCTGGCAGCATGCTGCCTAATAAAAAGGCCGGAAAATCCGGCC<br>TTTTGACGCTTTAGCTCTGACGGCTCAGTGCTGCCCACAGGCTTTCTTCAACACCGGTCAGCAGTTCATCGGTCAGGCTCAGATAATCGTTCTCGATCATTTCAT<br>ACACTTCCATAAAGCTGCCAAACAGAATTGCAATCAGTGCATTTTCCGGCAGATTACGAATCACACCCTGTTTCTGACCTTCACGAAAAAGGTGCACACAAATT<br>CAACCAGTTTCTGATATGCCAGACGGCTTTCTTCGGTCAGAAAGGTGCCCTGGCTATGGGTTTTGATAAAACCCAGTGCACGCGGATGATTTTTGGTAAAGGTCA<br>CCATACCTTCAAAAATGTGATGAAAGCCATCACGATAACCATCACGTTCATTTGCCAGACCGCTTTCAATACACTGCAGAAATTCATTAACATGCTGCTGAAACA<br>GTTCGTTCACCAGGCTTTCTTTGTTTTTGAAATAGCGATAAATGGTGCCTGCACCAACTTTTGCATTTTCTGCAATCATCGGCATGGTGGTTGCATCAAAACCAC<br>GTTCTGCAAACAGCAGCAGGCTTGCGCTAAAAATTGCTTTCTGTTTGGTCGGGGTGCTTTCCATCCTCCCGGCCTCGTTTGGTTAAACAAAATTATTTGTAGAGG<br>ACTGTTTCGGCCCTTTTGGGCCATCGTCAGGTCGGATACACATCCGGCGACAGTCTGCTAGCAGCCTACGTAGGAAGTGATGAGTTGTCAAGTTTGTCACTATCG<br>CCCTCTAGTGACGAAGTAGGACCAAAACGAAAAAAGGCCCCCCTTTCGGGAGGCCTCTTTTCTGGAATTTGGTACCGAGTGCAGACGTAAAAAAAGCGGCGTGGT<br>TAGCCGCTTTTTTAATTGCCGGATTAACGCTGTGTACCCGGACAAACACCATTAATCAGCAGGAAGGTAAATTCTTCAATATCCTGTTCAACGGTCAGCTGTTCG<br>GTCAGCAGGCGATACCAACAAAAACCAAAAATCATATCCAGCAGCAGTTCACGATTGGTATCTTTCGGCAGTTCACCATTGCTAATGGCATTTTCAACCAGTTTT<br>TTCGGCATCTCACGACGACGTTCCATAAACTGATCTTTCAGCTGGGTCAGGGTTGCAGGGTCCAGCTGTGCTTCTGCAATAACACAACGAAATGCTTCACCACAA<br>ATGGTTTCACGCCAAACTTTCCACAGATTACGCAGCAGAAAATCCAGATCGGCTTTAAAGCTACCCAGATCCGGAAATTTACGCACCTGTTCGCTTTCATTTTCA<br>TACACTTCGGCAATCAGTGCTGCTTTATTGGTCCACCAACGATAAATGGTCGGTTTGCTTGCACCGGCACGACGTGCAACGCTTTCAATGCTCAGACCGCTATAA<br>CCACATTCTTTCAGGATTTCAATGGTGCTGGTCAGAATTGCTTTATGGGTATGCGGACTACGCAGGCTACCAATGCTGCTACGGCTCGGGGTACGTGCCATCTAG<br>TATTTCTCCCTTTCAAACATAGTCCATAGTTAAACAAAATTATTTGTAGAGGCGGTTTCGACATCACTGTCTCATCAGAACGCAAAGCACATGCGTTGACCGCTG<br>TGCTCATTATATCTATCACTGATAGGGATGTCAATCTCTATCACTGATAGGGAGCGAGAGTGGTTGCGCTCCCAGTACAATCTGCTCTGATGCCCTCGAGTGCGG<br>CCGCACTAGTGTCGACGGAGCTCGAATTCGGATCCGAAGTTCCTATACTTTCTAGAGAATAGGAACTTCGGACCAAAACGAAAAAACACCCTTTCGGGTGTCTTT<br>TCTGGAATTTGGTACCGAGCCTTTGGTCGAAAAAAAAAGCCCGCACTGTCAGGTGCGGGCTTTTTTCTGTGTTTCC
YJP_MKC263_LP#2	ACATTTAATAAAAAAAGGGCGGTCGCAAGATCGCCCTTTTTTACGTATGACACAGTGAAAAATGGCGCCCATCGGCGCCATTTTTTTATGGAAGTTCCTATTCTC<br>TAGAAAGTATAGGAACTTCGGATCCGAATTCGAGCTCCGTCGACACTAGTGCGGCCGCACTCGAGGGCATCAGAGCAGATTGGGAGGGTATGGAAGCTATACGTT<br>ACCAATTGACAGCTAGCTCAGTCCTACTTTAGTATATAGACCGTGCGATCGGTCTATAAGACTGTCGCCGGATGTGTATCCGACCTGACGATGGCCCAAAAGGGC<br>CGAAACAGTCCTCTACAAATAATTTTGTTTAACCAAACGAGGCCGGGAGGATGGAAAGCACCCCGACCAAACAGAAAGCAATTTTTAGCGCAAGCCTGCTGCTGT<br>TTGCAGAACGTGGTTTTGATGCAACCACCATGCCGATGATTGCAGAAAATGCAAAAGTTGGTGCAGGCACCATTTATCGCTATTTCAAAAACAAAGAAAGCCTGG<br>TGAACGAACTGTTTCAGCAGCATGTTAATGAATTTCTGCAGTGTATTGAAAGCGGTCTGGCAAATGAACGTGATGGTTATCGTGATGGCTTTCATCACATTTTG

	AAGGTATGGTGACCTTTACCAAAAATCATCCGCGTGCACTGGGTTTTATCAAAACCCATAGCCAGGGCACCTTTCTGACCGAAGAAAGCCGTCTGGCATATCAGA<br>AACTGGTTGAATTTGTGTGCACCTTTTTTCGTGAAGGTCAGAAACAGGGTGTGATTCGTAATCTGCCGGAAAATGCACTGATTGCAATTCTGTTTGGCAGCTTTA<br>TGGAAGTGTATGAAATGATCGAGAACGATTATCTGAGCCTGACCGATGAACTGCTGACCGGTGTTGAAGAAAGCCTGTGGGCAGCACTGAGCCGTCAGAGCTAAA<br>GCGTCAAAAGGCCGGATTTTCCGGCCTTTTTTATTAGGCAGCATGCTGCCAGGTGATCCCCCTGGCCACCTCTTTTTACTCGACGTACGGTGGAATCTGATTCGT<br>TACCAATTGACATGATACGAAACGTACCGTATCGTTAAGGTAGTCGTCAAGTGCTGTGCTTGCACTTCTGATGAGGCAGTGATGCCGAAACGACCTCTACAAATA<br>ATTTTGTTTAAGCCATGCCATTGGCTTTTGATAGAGGACAACTACTAGATGAACCTGAAAGATAAAATTCTGGGCGTTGCCAAAGAACTGTTTATCAAAAATGGC<br>TATAACGCAACCACCACCGGTGAAATTGTTAAACTGAGCGAAAGCAGCAAAGGCAATCTGTATTATCACTTTAAAACCAAAGAGAACCTGTTTCTGGAAATCCTG<br>AACATCGAAGAAAGCAAATGGCAAGAGCAGTGGAAAAAAGAACAAATCAAATGCAAAACCAACCGCGAGAAATTCTATCTGTATAATGAACTGAGCCTGACCACC<br>GAATATTACTATCCGCTGCAGAATGCCATCATCGAGTTTTATACCGAGTACTATAAAACCAACAGCATCAACGAGAAAATGAACAAACTGGAAAACAAATACATC<br>GATGCCTACCACGTGATCTTTAAAGAAGGTAATCTGAACGGCGAATGGTGCATTAATGATGTTAATGCCGTGAGCAAAATTGCAGCAAATGCCGTTAATGGCATT<br>GTTACCTTTACCCATGAGCAGAATATCAACGAACGCATTAAACTGATGAACAAATTCAGCCAGATCTTTCTGAATGGCCTGAGCAAATAATCTTTAAAAAGAAAC<br>CTCCGCATTGCGGAGGTTTCGCCTTTTGATACTCTGTCTGAAGTAATTCTTGCCGCAGTGAAAAATGGCGCCCATCGGCGCCATTTTTTTATGCTTCCATTAGAA<br>AGCAAAAAGCCTGCTAGAAAGCAGGCTTTTTTGAATTTGGCTCCTCTGACAATGCGACGTACGGTGGAATCTGATTCGTTACCAATTGACATGATACGAAACGTA<br>CCGTATCGTTAAGGTAGGGGTCAGTTGATGTGCTTTCAACTCTGATGAGTCAGTGATGACGAAACCCCCTCTACAAATAATTTTGTTTAATACGCTATGGACTAT<br>GTTTCAAACAGGAGCTAATAGATGAACAAAACCATTGATCAGGTGCGTAAAGGTGATCGTAAAAGCGATCTGCCGGTTCGTCGTCGTCCGCGTCATAGTGCCGAA<br>GAAACCCGTCGTGATATTCTGGCAAAAGCCGAAGAACTGTTTAGTGAACGTGGTTTTAATGCAGTTGCCATTGCAGATATTGCAAGCGCACTGAATATGAGTCCG<br>GCAAATGTGTTTAAACATTTTAGCAGCAAAAACGCACTGGTTGATGCAATTGGTTTTGGTCAGATTGGTGTTTTTGAACGTCAGATTTGTCCGCTGGATAAAAGC<br>CATGCACCGCTGGATCGTCTGCGTCATCTGGCACGTAATCTGATGGAACAGCATCATCAGGATCATTTCAAACACATACGGGTTTTTATTCAGATCCTGATGACC<br>GCCAAACAGGATATGAAATGTGGCGATTATTACAAAAGCGTGATTGCAAAACTGCTGGCCGAAATTATTCGTGATGGTGTTGAAGCAGGTCTGTATATTGCAACC<br>GATATTCCGGTTCTGGCAGAAACCGTTCTGCATGCACTGACCAGCGTTATTCATCCGGTTCTGATTGCACAAGAAGATATTGGTAATCTGGCAACCCGTTGTGAT<br>CAGCTGGTTGATCTGATTGATGCAGGTCTGCGTAATCCGCTGGCAAAATAAACATTTAATAAAAAAAGGGCGGTCGCAAGATCGCCCTTTTTTACGTATGACACA<br>GTGAAAAATGGCGCCCATCGGCGCCATTTTTTTATGAGGTAACGAATGGCTCATGTCTGATTCGTTACCAATTGACGGAATGAACGTTCATTCCGATAATGCTAG<br>CAGCTGTCACCGGATGTGCTTTCCGGTCTGATGAGTCCGTGAGGACGAAACAGCCTCTACAAATAATTTTGTTTAATACTAGAGAAAGAGGAGAAATACTAGATG<br>GTGAGCAAGGGCGAGGAGCTGTTCACCGGGGTGGTGCCCATCCTGGTCGAGCTGGACGGCGACGTAAACGGCCACAAGTTCAGCGTGTCCGGCGAGGGCGAGGGC<br>GATGCCACCTACGGCAAGCTGACCCTGAAGTTCATCTGCACCACAGGCAAGCTGCCCGTGCCCTGGCCCACCCTCGTGACCACCTTCGGCTACGGCCTGCAATGC<br>TTCGCCCGCTACCCCGACCACATGAAGCTGCACGACTTCTTCAAGTCCGCCATGCCCGAAGGCTACGTCCAGGAGCGCACCATCTTCTTCAAGGACGACGGCAAC<br>TACAAGACCCGCGCCGAGGTGAAGTTCGAGGGCGACACCCTGGTGAACCGCATCGAGCTGAAGGGCATCGACTTCAAGGAGGACGGCAACATCCTGGGGCACAAG<br>CTGGAGTACAACTACAACAGCCACAACGTCTATATCATGGCCGACAAGCAGAAGAACGGCATCAAGGTGAACTTCAAGATCCGCCACAACATCGAGGACGGCAGC<br>GTGCAGCTCGCCGACCACTACCAGCAGAACACCCCAATCGGCGACGGCCCCGTGCTGCTGCCCGACAACCACTACCTTAGCTACCAGTCCGCCCTGAGCAAAGAC<br>CCCAACGAGAAGCGCGATCACATGGTCCTGCTGGAGTTCGTGACCGCCGCCGGGATCACTCTCGGCATGGACGAGCTGTACAAGTAAGCCTGTGTTATAAACCTG<br>TGTGAGAGTTAAGTTTACATGGCAAAGTTGATGACCGGGTCGTCCGTTGCAGACAAAAAAAATGGCGCACAATGTGCGCCATTTTTCACTTCACAGGTACTATT<br>GTTTTGAATTGAAAAGGGCGCTTCGGCGCCCTTTTTGCATTTGTTGACGGCATATATTTGTATATCGAAGCGCCCTGATGGGCGCTTTTTTTATTTAATCGATAA<br>CCAGA
YJP_MKC264_LP#1	AAAAAAAACCCCGCCCTGTCAGGGGCGGGGTTTTTTTTTTTCTTTTGGGTATAGCGTCGTGGACAGTCATTCATCTTTCTGCCCCTCCAAAAGCAAAAACCCGC<br>CGAAGCGGGTTTTTACGTAAATCAGGTGAAACTGACCGATAAGCCGGGACGAGCTGAGCAGTATGTCGACGGTCCGGCGAGAATCTCGCGTAGACACATACATGA<br>GCAAGCGTCAGAGGAGCCAAATTCAAAAAAGCCTGCTTTCTAGCAGGCTTTTGCTTTCTAATGGAAGCATAAAAAAATGGCGCCGATGGGCGCCATTTTTCACT<br>GCGGCAAGAATTACTTCAGACAGAGTATCAAAAGGCGAAACCTCCGCAATGCGGAGGTTTCTTTTTAAAGATTATTTGCTCAGGCCATTCAGAAAGATCTGGCTG<br>AATTTGTTCATCAGTTTAATGCGTTCGTTGATATTCTGCTCATGGGTAAAGGTAACAATGCCATTAACGGCATTTGCTGCAATTTTGCTCACGGCATTAACATCA<br>TTAATGCACCATTCGCCGTTCAGATTACCTTCTTTAAAGATCACGTGGTAGGCATCGATGTATTTGTTTTCCAGTTTGTTCATTTTCTCGTTGATGCTGTTGGTT<br>TTATAGTACTCGGTATAAAACTCGATGATGGCATTCTGCAGCGGATAGTAATATTCGGTGGTCAGGCTCAGTTCATTATACAGATAGAATTTCTCGCGGTTGGTT<br>TTGCATTTGATTTGTTCTTTTTTCCACTGCTCTTGCCATTTGCTTTCTTCGATGTTCAGGATTTCCAGAAACAGGTTCTCTTTGGTTTTAAAGTGATAATACAGA<br>TTGCCTTTGCTGCTTTCGCTCAGTTTAACAATTTCACCGGTGGTGGTTGCGTTATAGCCATTTTTGATAAACAGTTCTTTGGCAACGCCCAGAATTTTATCTTTC<br>AGGTTCATCTAGTAGTTGTCCTCTATCAAAAGCCAATGGCATGGCTTAAACAAAATTATTTGTAGAGGTCGTTTCGGCATCACTGCCTCATCAGAAGTGCAAGCA<br>CAGCACTTGACGACTGCTAGCAGCCTACGTAGGAAGTGATGAGTTGTCAAGTTTGTCACTATCGCCCTCTAGTGACGAACCTCATAAAAAAATGGCGCCGATGGG<br>CGCCATTTTTCACTGTGTCATACGTAAAAAAGGGCGATCTTGCGACCGCCCTTTTTTTATTAAATGTTTATTTTGCCAGCGGATTACGCAGACCTGCATCAATCA<br>GATCAACCAGCTGATCACAACGGGTTGCCAGATTACCAATATCTTCTTGTGCAATCAGAACCGGATGAATAACGCTGGTCAGTGCATGCAGAACGGTTTCTGCCA<br>GAACCGGAATATCGGTTGCAATATACAGACCTGCTTCAACACCATCACGAATAATTTCGGCCAGCAGTTTTGCAATCACGCTTTTGTAATAATCGCCACATTTCA<br>TATCCTGTTTGGCGGTCATCAGGATCTGAATAAAAACCCGTATGTGTTTGAAATGATCCTGATGATGCTGTTCCATCAGATTACGTGCCAGATGACGCAGACGAT<br>CCAGCGGTGCATGGCTTTTATCCAGCGGACAAATCTGACGTTCAAAAACACCAATCTGACCAAAACCAATTGCATCAACCAGTGCGTTTTTGCTGCTAAAATGTT<br>TAAACACATTTGCCGGACTCATATTCAGTGCGCTTGCAATATCTGCAATGGCAACTGCATTAAAACCACGTTCACTAAACAGTTCTTCGGCTTTTGCCAGAATAT<br>CACGACGGGTTTCTTCGGCACTATGACGCGGACGACGACGAACCGGCAGATCGCTTTTACGATCACCTTTACGCACCTGATCAATGGTTTTGTTCATCTATTAGC<br>TCCTGTTTGAAACATAGTCCATAGCGTATTAAACAAAATTATTTGTAGAGGGGGTTTCGTCATCACTGACTCATCAGAGTTGAAAGCACATCAACTGACCCCTAG<br>CGTTTTCAAGTTCGTGGAAAGCAGAATGGTTCAGGCGAGCCGGATCGGAAATTCCCTCAGATAGGCCCCCCAGATGAGGGGGATCAGAGCATGTCGCGCAAAAG<br>TGTGCAGCGGTTTTGCGATAACGACATGCGCCAAAACAAAGACTGAAAGCGCGAGGAGCGAATCTGAAAGATCGCGACCCGCTTTAGAGCGCCGTGCGTCCGTTT<br>GGACGCACAAAGGGACTTTTGTGCTACCACATTAAAAGAGGTGGCCAGGGGGATCACCTGGCAGCATGCTGCCTAATAAAAAGGCCGGAAAATCCGGCCTTTTG<br>ACGCTTTAGCTCTGACGGCTCAGTGCTGCCCACAGGCTTTCTTCAACACCGGTCAGCAGTTCATCGGTCAGGCTCAGATAATCGTTCTCGATCATTTCATACACT<br>TCCATAAAGCTGCCAAACAGAATTGCAATCAGTGCATTTTCCGGCAGATTACGAATCACACCCTGTTTCTGACCTTCACGAAAAAAGGTGCACACAAATTCAACC<br>AGTTTCTGATATGCCAGACGGCTTTCTTCGGTCAGAAAGGTGCCCTGGCTATGGGTTTTGATAAAACCCAGTGCACGCGGATGATTTTTGGTAAAGGTCACCATA<br>CCTTCAAAAATGTGATGAAAGCCATCACGATAACCATCACGTTCATTTGCCAGACCGCTTTCAATACACTGCAGAAATTCATTAACATGCTGCTGAAACAGTTCG<br>TTCACCAGGCTTTCTTTGTTTTTGAAATAGCGATAAATGGTGCCTGCACCAACTTTTGCATTTTCTGCAATCATCGGCATGGTGGTTGCATCAAAACCACGTTCT<br>GCAAACAGCAGCAGGCTTGCGCTAAAAATTGCTTTCTGTTTGGTCGGGGTGCTTTCCATCCTCCCGGCCTCGTTTGGTTAAACAAAATTATTTGTAGAGGACTGT<br>TTCGGCCCTTTTGGGCCATCGTCAGGTCGGATACACATCCGGCGACAGTCTGTGCTCATTATATCTATCACTGATAGGGATGTCAATCTCTATCACTGATAGGGA<br>GCGAGAGTGGTTGCGAGTAGGACCAAAACGAAAAAAGGCCCCCCTTTCGGGAGGCCTCTTTTCTGGAATTTGGTACCGAGTGCAGACGTAAAAAAAGCGGCGTGG<br>TTAGCCGCTTTTTTAATTGCCGGATTAACGCTGTGTACCCGGACAAACACCATTAATCAGCAGGAAGGTAAATTCTTCAATATCCTGTTCAACGGTCAGCTGTTC<br>GGTCAGCAGGCGATACCAACAAAAACCAAAAATCATATCCAGCAGCAGTTCACGATTGGTATCTTTCGGCAGTTCACCATTGCTAATGGCATTTTCAACCAGTTT<br>TTTCGGCATCTCACGACGACGTTCCATAAACTGATCTTTCAGCTGGGTCAGGGTTGCAGGGTCCAGCTGTGCTTCTGCAATAACACAACGAAATGCTTCACCACA<br>AATGGTTTCACGCCAAACTTTCCACAGATTACGCAGCAGAAAATCCAGATCGGCTTTAAAGCTACCCAGATCCGGAAATTTACGCACCTGTTCGCTTTCATTTTC<br>ATACACTTCGGCAATCAGTGCTGCTTTATTGGTCCACCAACGATAAATGGTCGGTTTGCTTGCACCGGCACGACGTGCAACGCTTTCAATGCTCAGACCGCTATA<br>ACCACATTCTTTCAGGATTTCAATGGTGCTGGTCAGAATTGCTTTATGGGTATGCGGACTACGCAGGCTACCAATGCTGCTACGGCTCGGGGTACGTGCCATCTA<br>GTATTTCTCCCTTTCAAACATAGTCCATAGTTAAACAAAATTATTTGTAGAGGCGGTTTCGACATCACTGTCTCATCAGAACGCAAAGCACATGCGTTGACCGCT<br>TATAGACCGATCGCACGGTCTATATACTAAAGTAGGACTGAGCTAGCTGTCAATTGGTAACGTATAGCTTCCATACCCTCCCAGTACAATCTGCTCTGATGCCCT<br>CGAGTGCGGCCGCACTAGTGTCGACGGAGCTCGAATTCGGATCCGAAGTTCCTATACTTTCTAGAGAATAGGAACTTCGGACCAAAACGAAAAAACACCCTTTCG<br>GGTGTCTTTTCTGGAATTTGGTACCGAGCCTTTGGTCGAAAAAAAAAGCCCGCACTGTCAGGTGCGGGCTTTTTTCTGTGTTTCC
YJP_MKC264_LP#2	ACATTTAATAAAAAAAGGGCGGTCGCAAGATCGCCCTTTTTTACGTATGACACAGTGAAAAATGGCGCCCATCGGCGCCATTTTTTTATGGAAGTTCCTATTCTC<br>TAGAAAGTATAGGAACTTCGGATCCGAATTCGAGCTCCGTCGACACTAGTGCGGCCGCACTCGAGGGCATCAGAGCAGATTGGGAGCGACGTACGGTGGAATCTG<br>ATTCGTTACCAATTGACATGATACGAAACGTACCGTATCGTTAAGGTAGACTGTCGCCGGATGTGTATCCGACCTGACGATGGCCCAAAAGGGCCGAAACAGTCC<br>TCTACAAATAATTTTGTTTAACCAAACGAGGCCGGGAGGATGGAAAGCACCCCGACCAAACAGAAAGCAATTTTTAGCGCAAGCCTGCTGCTGTTTGCAGAACGT<br>GGTTTTGATGCAACCACCATGCCGATGATTGCAGAAAATGCAAAAGTTGGTGCAGGCACCATTTATCGCTATTTCAAAAACAAAGAAAGCCTGGTGAACGAACTG<br>TTTCAGCAGCATGTTAATGAATTTCTGCAGTGTATTGAAAGCGGTCTGGCAAATGAACGTGATGGTTATCGTGATGGCTTTCATCACATTTTTGAAGGTATGGTG<br>ACCTTTACCAAAAATCATCCGCGTGCACTGGGTTTTATCAAAACCCATAGCCAGGGCACCTTTCTGACCGAAGAAAGCCGTCTGGCATATCAGAAACTGGTTGAA<br>TTTGTGTGCACCTTTTTTCGTGAAGGTCAGAAACAGGGTGTGATTCGTAATCTGCCGGAAAATGCACTGATTGCAATTCTGTTTGGCAGCTTTATGGAAGTGTAT<br>GAAATGATCGAGAACGATTATCTGAGCCTGACCGATGAACTGCTGACCGGTGTTGAAGAAAGCCTGTGGGCAGCACTGAGCCGTCAGAGCTAAAGCGTCAAAAGG<br>CCGGATTTTCCGGCCTTTTTTATTAGGCAGCATGCTGCCAGGTGATCCCCCTGGCCACCTCTTTTTACTAACGATCGTTGGCTGTGTTGACAATTAATCATCGGC<br>TCGTATAATGTGTGGAATTGTGAGCGCTCACAATTAGTCGTCAAGTGCTGTGCTTGCACTTCTGATGAGGCAGTGATGCCGAAACGACCTCTACAAATAATTTTG<br>TTTAAGCCATGCCATTGGCTTTTGATAGAGGACAACTACTAGATGAACCTGAAAGATAAAATTCTGGGCGTTGCCAAAGAACTGTTTATCAAAAATGGCTATAAC<br>GCAACCACCACCGGTGAAATTGTTAAACTGAGCGAAAGCAGCAAAGGCAATCTGTATTATCACTTTAAAACCAAAGAGAACCTGTTTCTGGAAATCCTGAACATC<br>GAAGAAAGCAAATGGCAAGAGCAGTGGAAAAAAGAACAAATCAAATGCAAAACCAACCGCGAGAAATTCTATCTGTATAATGAACTGAGCCTGACCACCGAATAT<br>TACTATCCGCTGCAGAATGCCATCATCGAGTTTTATACCGAGTACTATAAAACCAACAGCATCAACGAGAAAATGAACAAACTGGAAAACAAATACATCGATGCC<br>TACCACGTGATCTTTAAAGAAGGTAATCTGAACGGCGAATGGTGCATTAATGATGTTAATGCCGTGAGCAAAATTGCAGCAAATGCCGTTAATGGCATTGTTACC<br>TTTACCCATGAGCAGAATATCAACGAACGCATTAAACTGATGAACAAATTCAGCCAGATCTTTCTGAATGGCCTGAGCAAATAATCTTTAAAAAGAAACCTCCGC<br>ATTGCGGAGGTTTCGCCTTTTGATACTCTGTCTGAAGTAATTCTTGCCGCAGTGAAAAATGGCGCCCATCGGCGCCATTTTTTTATGCTTCCATTAGAAAGCAAA<br>AAGCCTGCTAGAAAGCAGGCTTTTTTGAATTTGGCTCCTCTGACAATGAACGAATGGCTCATGTCTGATTCGTTACCAATTGACGGAATGAACGTTCATTCCGAT<br>AATGCTAGCAGCTGTCACCGGATGTGCTTTCCGGTCTGATGAGTCCGTGAGGACGAAACAGCCTCTACAAATAATTTTGTTTAATACTAGAGAAAGAGGAGAAAT<br>ACTAGATGGTGAGCAAGGGCGAGGAGCTGTTCACCGGGGTGGTGCCCATCCTGGTCGAGCTGGACGGCGACGTAAACGGCCACAAGTTCAGCGTGTCCGGCGAGG<br>GCGAGGGCGATGCCACCTACGGCAAGCTGACCCTGAAGTTCATCTGCACCACAGGCAAGCTGCCCGTGCCCTGGCCCACCCTCGTGACCACCTTCGGCTACGGCC

	TGCAATGCTTCGCCCGCTACCCCGACCACATGAAGCTGCACGACTTCTTCAAGTCCGCCATGCCCGAAGGCTACGTCCAGGAGCGCACCATCTTCTTCAAGGACG<br>ACGGCAACTACAAGACCCGCGCCGAGGTGAAGTTCGAGGGCGACACCCTGGTGAACCGCATCGAGCTGAAGGGCATCGACTTCAAGGAGGACGGCAACATCCTGG<br>GGCACAAGCTGGAGTACAACTACAACAGCCACAACGTCTATATCATGGCCGACAAGCAGAAGAACGGCATCAAGGTGAACTTCAAGATCCGCCACAACATCGAGG<br>ACGGCAGCGTGCAGCTCGCCGACCACTACCAGCAGAACACCCCCAATCGGCGACGGCCCCGTGCTGCTGCCCGACAACCACTACCTTAGCTACCAGTCCGCCCTGA<br>GCAAAGACCCCAACGAGAAGCGCGATCACATGGTCCTGCTGGAGTTCGTGACCGCCGCCGGGATCACTCTCGGCATGGACGAGCTGTACAAGTAAAGGTGTGTTA<br>TAAACCTGTGTGAGAGTTAAGTTTACATGGGCAAAGTTGATGACCGGGTCGTCCGTTGCAGACAAAAAAAATGGCGCACAATGTGCGCCATTTTTCACTTCACAG<br>GTACTATTGTTTTGAATTGAAAAGGGCGCTTCGGCGCCCTTTTTGCATTTGTTGACGGCATATATTTGTATATCGAAGCGCCCTGATGGGCGCTTTTTTTATTTA<br>ATCGATAACCAGA
YJP_MKC265_LP#1	AAAAAAAACCCCGCCCTGTCAGGGGCGGGGTTTTTTTTTTTTCTTTTGGGTATAGCGTCGTGGACAGTCATTCATCTTTCTGCCCCTCCAAAAGCAAAAACCCGC<br>CGAAGCGGGTTTTTACGTAAATCAGGTGAAACTGACCGATAAGCCGGGACGAGCTGAGCAGTATGTCGACGGTCCGGCGAGAATCTCGCGTAGACACATACATGA<br>GCAAGCGTCAGAGGAGCCAAATTCAAAAAAGCCTGCTTTCTAGCAGGCTTTTTGCTTTCTAATGGAAGCATAAAAAAATGGCGCCGATGGGCGCCATTTTTCACT<br>GCGGCAAGAATTACTTCAGACAGAGTATCAAAAGGCGAAACCTCCGCAATGCGGAGGTTTCTTTTTAAAGATTATTTGCTCAGGCCATTCAGAAAGATCTGGCTG<br>AATTTGTTCATCAGTTTAATGCGTTCGTTGATATTCTGCTCATGGGTAAAGGTAACAATGCCATTAACGGCATTTGCTGCAATTTTGCTCACGGCATTAACATCA<br>TTAATGCACCATTCGCCGTTCAGATTACCTTCTTTAAAGATCACGTGGTAGGCATCGATGTATTTGTTTTCCAGTTTGTTCATTTTCTCGTTGATGCTGTTGGTT<br>TTATAGTACTCGGTATAAAACTCGATGATGGCATTCTGCAGCGGATAGTAATATTCGGTGGTCAGGCTCAGTTCATTATACAGATAGAATTTCTCGCGGTTGGTT<br>TTGCATTTGATTTGTTCTTTTTTCCACTGCTCTTGCCATTTGCTTTCTTCGATGTTCAGGATTTCCAGAAACAGGTTCTCTTTGGTTTTAAAGTGATAATACAGA<br>TTGCCTTTGCTGCTTTCGCTCAGTTTAACAATTTCACCGGTGGTGGTTGCGTTATAGCCATTTTTGATAAACAGTTCTTTGGCAACGCCCAGAATTTTATCTTTC<br>AGGTTCATCTAGTAGTTGTCCTCTATCAAAAGCCAATGGCATGGCTTAAACAAAATTATTTGTAGAGGTCGTTTCGGCATCACTGCCTCATCAGAAGTGCAAGCA<br>CAGCACTTGACGACTGTGCTCATTATATCTATCACTGATAGGGATGTCAATCTCTATCACTGATAGGGAGCGAGAGTGGTTGCGACCTCATAAAAAAAATGGCGCC<br>GATGGGCGCCATTTTTCACTGTGTCATACGTAAAAAAGGGCGATCTTGCGACCGCCCTTTTTTTATTAAATGTTTATTTGCCAGCGGATTACGCAGACCTGCAT<br>CAATCAGATCAACCAGCTGATCACAACGGGTTGCCAGATTACCAATATCTTCTTGTGCAATCAGAACCGGATGAATAACGCTGGTCAGTGCATGCAGAACGGTTT<br>CTGCCAGAACCGGAATATCGGTTGCAATATACAGACCTGCTTCAACACCATCACGAATAATTTCGGCCAGCAGTTTTGCAATCACGCTTTTGTAATAATCGCCAC<br>ATTTCATATCCTGTTTGGCGGTCATCAGGATCTGAATAAAAACCCGTATGTGTTTGAAATGATCCTGATGATGCTGTTCCATCAGATTACGTGCCAGATGACGCA<br>GACGATCCAGCGGTGCATGGCTTTTATCCAGCGGACAAATCTGACGTTCAAAAACACCAATCTGACCAAAACCAATTGCATCAACCAGTGCGTTTTTGCTGCTAA<br>AATGTTTAAACACATTTGCCGGACTCATATTCAGTGCGCTTGCAATATCTGCAATGGCAACTGCATTAAAACCACGTTCACTAAACAGTTCTTCGGCTTTTGCCA<br>GAATATCACGACGGGTTTCTTCGGCACTATGACGCGGACGACGAACCGGCAGATCGCTTTTACGATCACCTTTACGCACCTGATCAATGGTTTTGTTCATCT<br>ATTAGCTCCTGTTTGAAACATAGTCCATAGCGTATTAAACAAAATTATTTGTAGAGGGGGTTTCGTCATCACTGACTCATCAGAGTTGAAAGCACATCAACTGAC<br>CCCTAGCGTTTTCAAGTTCGTGGAAAGCAGAATGGTTCAGGCGAGCCGGATCGGAAATTCCCTCAGATAGGCCCCCCCAGATGAGGGGGATCAGAGCATGTCGCG<br>CAAAAGTGTGCAGCGGTTTTGCGATAACGACATGCGCCAAAACAAAGACTGAAAGCGCGAGGAGCGAATCTGAAAGATCGCGACCCGCTTTAGAGCGCCGTGCGT<br>CCGTTTGGACGCACAAAGGGACTTTTGTGCTACCACATTAAAAGAGGTGGCCAGGGGGATCACCTGGCAGCATGCTGCCTAATAAAAAAGGCCGGAAAATCCGGC<br>CTTTTGACGCTTTAGCTCTGACGGCTCAGTGCTGCCCACAGGCTTTCTTCAACACCGGTCAGCAGTTCATCGGTCAGGCTCAGATAATCGTTCTCGATCATTTCA<br>TACACTTCCATAAAGCTGCCAAACAGAATTGCAATCAGTGCATTTTCCGGCAGATTACGAATCACACCCTGTTTCTGACCTTCACGAAAAAAGGTGCACACAAAT<br>TCAACCAGTTTCTGATATGCCAGACGGCTTTCTTCGGTCAGAAAGGTGCCCTGGCTATGGGTTTGATAAAACCCAGTGCACGCGGATGATTTTTGGTAAAGGTC<br>ACCATACCTTCAAAAATGTGATGAAAGCCATCACGATAACCATCACGTTCATTTGCCAGACCGCTTTCAATACACTGCAGAAATTCATTAACATGCTGCTGAAAC<br>AGTTCGTTCACCAGGCTTTCTTTGTTTTTGAAATAGCGATAAATGGTGCCTGCACCAACTTTTGCATTTTCTGCAATCATCGGCATGGTGGTTGCATCAAAACCA<br>CGTTCTGCAAACAGCAGCAGGCTTGCGCTAAAAATTGCTTTCTGTTTGGTCGGGGTGCTTTCCATCCTCCCGGCCTCGTTTGGTTAAACAAAATTATTTGTAGAG<br>GACTGTTTCGGCCCTTTTGGGCCATCGTCAGGTCGGATACACATCCGGCGACAGTCTTATAGACCGATCGCACGGTCTATATACTAAAGTAGGACTGAGCTAGCT<br>GTCAATTGGTAACGTATAGCTTCCATACCAGTAGGACCAAAACGAAAAAAGGCCCCCCTTTCGGGAGGCCTCTTTTCTGGAATTTGGTACCGAGTGCAGACGTAA<br>AAAAAGCGGCGTGGTTAGCCGCTTTTTTAATTGCCGGATTAACGCTGTGTACCCGGACAAACACCATTAATCAGCAGGAAGGTAAATTCTTCAATATCCTGTTCA<br>ACGGTCAGCTGTTCGGTCAGCAGGCGATACCAACAAAAACCAAAAATCATATCCAGCAGCAGTTCACGATTGGTATCTTTCGGCAGTTCACCATTGCTAATGGCA<br>TTTTCAACCAGTTTTTTCGGCATCTCACGACGACGTTCCATAAACTGATCTTTCAGCTGGGTCAGGGTTGCAGGGTCCAGCTGTGCTTCTGCAATAACACAACGA<br>AATGCTTCACCACAAATGGTTTCACGCCAAACTTTCCACAGATTACGCAGCAGAAAATCCAGATCGGCTTTAAAGCTACCCAGATCCGGAAATTTACGCACCTGT<br>TCGCTTTCATTTTCATACACTTCGGCAATCAGTGCTGCTTTATTGGTCCACCAACGATAAATGGTCGGTTTGCTTGCACCGGCACGACGTGCAACGCTTTCAATG<br>CTCAGACCGCTATAACCACATTCTTTCAGGATTTCAATGGTGCTGGTCAGAATTGCTTTATGGGTATGCGGACTACGCAGGCTACCAATGCTGCTACGGCTCGGG<br>GTACGTGCCATCTAGTATTTCTCCCTTTCAAACATAGTCCATAGTTAAACAAAATTATTTGTAGAGGCGGTTTCGACATCACTGTCTCATCAGAACGCAAAGCAC<br>ATGCGTTGACCGCTGCTAGCAGCCTACGTAGGAAGTGATGAGTTGTCAAGTTTGTCACTATCGCCCTCTAGTGACGACTCCCAGTACAATCTGCTCTGATGCCCT<br>CGAGTGCGGCCGCACTAGTGTCGACGGAGCTCGAATTCGGATCCGAAGTTCCTATACTTTCTAGAGAATAGGAACTTCGGACCAAAACGAAAAAACACCCTTTCG<br>GGTGTCTTTTCTGGAATTTGGTACCGAGCCTTTGGTCGAAAAAAAAAGCCCGCACTGTCAGGTGCGGGCTTTTTTCTGTGTTTCC
YJP_MKC265_LP#2	ACATTTAATAAAAAAAGGGCGGTCGCAAGATCGCCCTTTTTTACGTATGACACAGTGAAAAATGGCGCCCATCGGCGCCATTTTTTTATGGAAGTTCCTATTCTC<br>TAGAAAGTATAGGAACTTCGGATCCGAATTCGAGCTCCGTCGACACTAGTGCGGCCGCACTCGAGGGCATCAGAGCAGATTGGGAGGAGCCTACCTGCCTTATTG<br>GATCCAATTGACAGCTAGCTCAGTCCTAGGTACCATTGGATCCAATAGCGGTCAACGCATGTGCTTTGCGTTCTGATGAGACAGTGATGTCGAAACCGCCTCTAC<br>AAATAATTTTGTTTAACTATGGACTATGTTTGAAAGGGAGAAATACTAGATGGCACGTACCCCGAGCCGTAGCAGCATTGGTAGCCTGCGTAGTCCGCATACCCA<br>TAAAGCAATTCTGACCAGCACCATTGAAATCCTGAAAGAATGTGGTTATAGCGGTCTGAGCATTGAAAGCGTTGCACGTCGTGCCGGTGCAAGCAAACCGACCAT<br>TTATCGTTGGTGGACCAATAAAGCAGCACTGATTGCCGAAGTGTATGAAAATGAAAGCGAACAGGTGCGTAAATTTCCGGATCTGGGTAGCTTTAAAGCCGATCT<br>GGATTTTCTGCTGCGTAATCTGTGGAAAGTTTGGCGTGAAACCATTTGTGGTGAAGCATTTCGTTGTGTTATTGCAGAAGCACAGCTGGACCCTGCAACCCTGAC<br>CCAGCTGAAAGATCAGTTTATGGAACGTCGTCGTGAGATGCCGAAAAAACTGGTTGAAAATGCCATTAGCAATGGTGAACTGCCGAAAGATACCAATCGTGAACT<br>GCTGCTGGATATGATTTTTGGTTTTTGTTGGTATCGCCTGCTGACCGAACAGCTGACCGTTGAACAGGATATTGAAGAATTTACCTTCCTGCTGATTAATGGTGT<br>TTGTCCGGGTACACAGCGTTAATCCGGCAATTAAAAAAGCGGCTAACCACGCCGCTTTTTTTACGTCTGCACTCGGTACCAAATTCCAGAAAAGAGGCCTCCCGA<br>AAGGGGGGCCTTTTTTCGTTTTGGTCCTACTCGACGTACGGTGGAATCTGATTCGTTACCAATTGACATGATACGAAACGTACCGTATCGTTAAGGTAGACTGTC<br>GCCGGATGTGTATCCGACCTGACGATGGCCCAAAAGGGCCGAAACAGTCCTCTACAAATAATTTTGTTTAACCAAACGAGGCCGGGAGGATGGAAAGCACCCCGA<br>CCAAACAGAAAGCAATTTTTAGCGCAAGCCTGCTGCTGTTTGCAGAACGTGGTTTTGATGCAACCACCATGCCGATGATTGCAGAAAATGCAAAAGTTGGTGCAG<br>GCACCATTTATCGCTATTTCAAAAACAAAGAAAGCCTGGTGAACGAACTGTTTCAGCAGCATGTTAATGAATTTCTGCAGTGTATTGAAAGCGGTCTGGCAAATG<br>AACGTGATGGTTATCGTGATGGCTTTCATCACATTTTTGAAGGTATGGTGACCTTTACCAAAAATCATCCGCGTGCACTGGGTTTTATCAAAACCCATAGCCAGG<br>GCACCTTTCTGACCGAAGAAAGCCGTCTGGCATATCAGAAACTGGTTGAATTTGTGTGCACCTTTTTTCGTGAAGGTCAGAAACAGGGTGTGATTCGTAATCTGC<br>CGGAAAATGCACTGATTGCAATTCTGTTTGGCAGCTTTATGGAAGTGTATGAAATGATCGAGAACGATTATCTGAGCCTGACCGATGAACTGCTGACCGGTGTTG<br>AAGAAAGCCTGTGGGCAGCACTGAGCCGTCAGAGCTAAAGCGTCAAAAGGCCGGATTTTCCGGCCTTTTTTATTAGGCAGCATGCTGCCAGGTGATCCCCCTGGC<br>CACCTCTTTTAATGAACGAATGGCTCATGTCTGATTCGTTACCAATTGACGGAATGAACGTTCATTCCGATAATGCTAGCAGCTGTCACCGGATGTGCTTTCCGG<br>TCTGATGAGTCCGTGAGGACGAAACAGCCTCTACAAATAATTTTGTTTAATACTAGAGAAAGAGGAGAAATACTAGATGGTGAGCAAGGGCGAGGAGCTGTTCAC<br>CGGGGTGGTGCCCATCCTGGTCGAGCTGGACGGCGACGTAAACGGCCACAAGTTCAGCGTGTCCGGCGAGGGCGAGGGCGATGCCACCTACGGCAAGCTGACCCT<br>GAAGTTCATCTGCACCACAGGCAAGCTGCCCGTGCCCTGGCCCACCCTCGTGACCACCTTCGGCTACGGCCTGCAATGCTTCGCCCGCTACCCCGACCACATGAA<br>GCTGCACGACTTCTTCAAGTCCGCCATGCCCGAAGGCTACGTCCAGGAGCGCACCATCTTCTTCAAGGACGACGGCAACTACAAGACCCGCGCCGAGGTGAAGTT<br>CGAGGGCGACACCCTGGTGAACCGCATCGAGCTGAAGGGCATCGACTTCAAGGAGGACGGCAACATCCTGGGGCACAAGCTGGAGTACAACTACAACAGCCACAA<br>CGTCTATATCATGGCCGACAAGCAGAAGAACGGCATCAAGGTGAACTTCAAGATCCGCCACAACATCGAGGACGGCAGCGTGCAGCTCGCCGACCACTACCAGCA<br>GAACACCCCCAATCGGCGACGGCCCCGTGCTGCTGCCCGACAACCACTACCTTAGCTACCAGTCCGCCCTGAGCAAAGACCCCAACGAGAAGCGCGATCACATGGT<br>CCTGCTGGAGTTCGTGACCGCCGCCGGGATCACTCTCGGCATGGACGAGCTGTACAAGTAAAGGTGTGTTATAAACCTGTGTGAGAGTTAAGTTTACATGGGCAA<br>AGTTGATGACCGGGTCGTCCGTTGCAGACAAAAAAAATGGCGCACAATGTGCGCCATTTTTCACTTCACAGGTACTATTGTTTTGAATTGAAAAGGGCGCTTCGG<br>CGCCCTTTTTGCATTTGTTGACGGCATATATTTGTATATCGAAGCGCCCTGATGGGCGCTTTTTTTATTTAATCGATAACCAGA
YJP_MKC266_LP#1	AAAAAAAACCCCGCCCTGTCAGGGGCGGGGTTTTTTTTTTTTCTTTTGGGTATAGCGTCGTGGACAGTCATTCATCTTTCTGCCCCTCCAAAAGCAAAAACCCGC<br>CGAAGCGGGTTTTTACGTAAATCAGGTGAAACTGACCGATAAGCCGGGACGAGCTGAGCAGTATGTCGACGGTCCGGCGAGAATCTCGCGTAGACACATACATGA<br>GCTAGTTTACTTGTACAGCTCGTCCATGCCGAGAGTGATCCCGGCGGCGGTCACGAACTCCAGCAGGACCATGTGATCGCGCTTCTCGTTGGGGTCTTTGCTCAG<br>GGCGGACTGGTAGCTAAGGTAGTGGTTGTCGGGCAGCAGCACGGGGCCGTCGCCGATTGGGGTGTTCTGCTGGTAGTGGTCGGCGAGCTGCACGCTGCCGTCCTC<br>GATGTTGTGGCGGATCTTGAAGTTCACCTTGATGCCGTTCTTCTGCTTGTCGGCCATGATATAGACGTTGTGGCTGTTGTAGTTGTACTCCAGCTTGTGCCCCAG<br>GATGTTGCCGTCCTCCTTGAAGTCGATGCCCTTCAGCTCGATGCGGTTCACCAGGGTGTCGCCCTCGAACTTCACCTCGGCGCGGGTCTTGTAGTTGCCGTCGTC<br>CTTGAAGAAGATGGTGCGCTCCTGGACGTAGCCTTCGGGCATGGCGGACTTGAAGAAGTCGTGCAGCTTCATGTGGTCGGGGTAGCGGGCGAAGCATTGCAGGCC<br>GTAGCCGAAGGTGGTCACGAGGGTGGGCCAGGGCACGGGCAGCTTGCCTGTGGTGCAGATGAACTTCAGGGTCAGCTTGCCGTAGGTGGCATCGCCCTCGCCCTC<br>GCCGGACACGCTGAACTTGTGGCCGTTTACGTCGCCGTCCAGCTCGACCAGGATGGGCACCACCCCGGTGAACAGCTCCTCGCCCTTGCTCACCATCTAGTATTT<br>CTCCTCTTTCTCTAGTATTAAACAAAATTATTTGTAGAGGCTGTTTCGTCCTCACGGACTCATCAGACCGGAAAGCACATCCGGTGACAGCTACCTTAACGATAC<br>GGTACGTTTCGTATCATGTCAATTGGTAACGAATCAGATTCCACCGTACGTCGAAGCGTCAGAGGAGCCAAATTCAAAAAAGCCTGCTTTCTAGCAGGCTTTTTG<br>CTTTCTAATGGAAGCATAAAAAAATGGCGCCGATGGGCGCCATTTTTCACTGCGGCAAGAATTACTTCAGACAGAGTATCAAAAGGCGAAACCTCCGCAATGCGG<br>AGGTTTCTTTTTAAAGATTATTTGCTCAGGCCATTCAGAAAGATCTGGCTGAATTTGTTCATCAGTTTAATGCGTTCGTTGATATTCTGCTCATGGGTAAAGGTA<br>ACAATGCCATTAACGGCATTTGCTGCAATTTTGCTCACGGCATTAACATCATTAATGCACCATTCGCCGTTCAGATTACCTTCTTTAAAGATCACGTGGTAGGCA<br>TCGATGTATTTGTTTTCCAGTTTGTTCATTTTCTCGTTGATGCTGTTGGTTTTATAGTACTCGGTATAAAACTCGATGATGGCATTCTGCAGCGGATAGTAATAT<br>TCGGTGGTCAGGCTCAGTTCATTATACAGATAGAATTTCTCGCGGTTGGTTTTGCATTTGATTTGTTCTTTTTTCCACTGCTCTTGCCATTTGCTTTCTTCGATG<br>TTCAGGATTTCCAGAAACAGGTTCTCTTTGGTTTTAAAGTGATAATACAGATTGCCTTTGCTGCTTTCGCTCAGTTTAACAATTTCACCGGTGGTGGTTGCGTTA

	TAGCCATTTTTGATAAACAGTTCTTTGGCAACGCCCAGAATTTTATCTTTCAGGTTCATCTAGTAGTTGTCCTCTATCAAAAGCCAATGGCATGGCTTAAACAAA<br>ATTATTTGTAGAGGTCGTTTCGGCATCACTGCCTCATCAGAAGTGCAAGCACAGCACTTGACGACTATTGGATCCAATGGTACCTAGGACTGAGCTAGCTGTCAA<br>TTGGATCCAATAAGGCAGGTAGGCTCACCTGCGGAGAAAAGGTCCGAAAATTCGGACCCGATGGAATTGGATAAAAAAAGGCCCCTGTTGAAATTGCAGGGGCCT<br>GGTACGAGCATTATTTTGCATCTGCCTGTTTAATCAGTTCCAGGGTTTTTTCAACACGATCTGCAGGCAGCGGTGCACCCAGAACTGCCAGGCTTGCATCTGCCA<br>GCATAATTGCGGTTTCCGGCAGGCTATCTGCGCTCAGCGGACTCGGAATTTTACCATCATTGCGACGCATTTCAATAACGCTCATGGTAATATGAAACGGCAGTT<br>CTGCACGCGGATCATCACCAACAATTTCGGTTGCCAGATCACGAAAAACATTGGTCAGTGCTTCACGCTGGCTATGATATTCTGCAAATTCTTCGCTACCAACAA<br>TCGGCAGCTGATACAGACGACCAACATTCCATTTGGTGCTCAGCAGCAGACGAACTTCGCTTGCAACAATTGCCCACAGACGCATTTCCGGACCTGCATCCAGGG<br>TGCTCAGATCTTCTGCCAGAACGGTGCTCGGTTCAACGGTGCTTTTCAGCAGGGTCAGAAAGATTTCGGTTTTGCTCGGAAAATGATAATACAGGCTTGCCTGAC<br>GAATACCAACTGCATCTGCAATCTGATGGGTACTGGTGGTTGCAAAACCCTGACGGGTAAACAGTTCTGCGCTTGCATCCAGAATTTCTTCACGCGGATTTTTAC<br>CTGCACGACGCGGTGCACTACGACGCGGACGACCAACTGCGCCTGCCATCTAGTAACCTCTTTGCTGATTATTAGGGAACATTTTAAACAAAATTATTTGTAGAG<br>GGGTGTTTCGTCCTTTCGGACTCATCAGTCAAGGTACGCACCTTGAGACACCCTGTGCTCATTATATCTATCACTGATAGGGATGTCAATCTCTATCACTGATAG<br>GGAGCGAGAGTGGTTGCGCATTCATAAAAAAATGGCGCCGATGGGCGCCATTTTTCACTGTGTCATACGTAAAAAAGGGCGATCTTGCGACCGCCCTTTTTTTAT<br>TAAATGTTTATTTTGCCAGCGGATTACGCAGACCTGCATCAATCAGATCAACCAGCTGATCACAACGGGTTGCCAGATTACCAATATCTTCTTGTGCAATCAGAA<br>CCGGATGAATAACGCTGGTCAGTGCATGCAGAACGGTTTCTGCCAGAACCGGAATATCGGTTGCAATATACAGACCTGCTTCAACACCATCACGAATAATTTCGG<br>CCAGCAGTTTTGCAATCACGCTTTTGTAATAATCGCCACATTTCATATCCTGTTTGGCGGTCATCAGGATCTGAATAAAAACCCGTATGTGTTTGAAATGATCCT<br>GATGATGCTGTTCCATCAGATTACGTGCCAGATGACGCAGACGATCCAGCGGTGCATGGCTTTTATCCAGCGGACAAATCTGACGTTCAAAAACACCAATCTGAC<br>CAAAACCAATTGCATCAACCAGTGCGTTTTTGCTGCTAAAATGTTTAAACACATTTGCCGGACTCATATTCAGTGCGCTTGCAATATCTGCAATGGCAACTGCAT<br>TAAAACCACGTTCACTAAACAGTTCTTCGGCTTTTGCCAGAATATCACGACGGGTTTCTTCGGCACTATGACGCGGACGACGAACCGGCAGATCGCTTTTAC<br>GATCACCTTTACGCACCTGATCAATGGTTTTGTTCATCTATTAGCTCCTGTTTGAAACATAGTCCATAGCGTATTAAACAAAATTATTTGTAGAGGGGGTTTCGT<br>CATCACTGACTCATCAGAGTTGAAAGCACATCAACTGACCCCTAGCGTTTTCAAGTTCGTGGAAAGCAGAATGGTTCAGGCGAGCCGGATCGGAAATTCCCTCAG<br>ATAGGCCCCCCCAGATGAGGGGGATCAGAGCATGTCGCGCAAAAGTGTGCAGCGGTTTTGCGATAACGACATGCGCCAAAACAAAGACTGAAAGCGCGAGGAGCG<br>AATCTGAAAGATCGCGACCCGCTTTAGAGCGCCGTGCGTCCGTTTGGACGCACAAAGGGACTTTTGTGCTACCAAGTAGGACCAAAACGAAAAAAGGCCCCCCTT<br>TCGGGAGGCCTCTTTTCTGGAATTTGGTACCGAGTGCAGACGTAAAAAAAGCGGCGTGGTTAGCCGCTTTTTTAATTGCCGGATTAACGCTGTGTACCCGGACAA<br>ACACCATTAATCAGCAGGAAGGTAAATTCTTCAATATCCTGTTCAACGGTCAGCTGTTCGGTCAGCAGGCGATACCAACAAAAACCAAAAATCATATCCAGCAGC<br>AGTTCACGATTGGTATCTTTCGGCAGTTCACCATTGCTAATGGCATTTTCAACCAGTTTTTTCGGCATCTCACGACGACGTTCCATAAACTGATCTTTCAGCTGG<br>GTCAGGGTTGCAGGGTCCAGCTGTGCTTCTGCAATAACACAACGAAATGCTTCACCACAAATGGTTTCACGCCAAACTTTCCACAGATTACGCAGCAGAAAATCC<br>AGATCGGCTTTAAAGCTACCCAGATCCGGAAATTTACGCACCTGTTCGCTTTCATTTTCATACACTTCGGCAATCAGTGCTGCTTTATTGGTCCACCAACGATAA<br>ATGGTCGGTTTGCTTGCACCGGCACGACGTGCAACGCTTTCAATGCTCAGACCGCTATAACCACATTCTTTCAGGATTTCAATGGTGCTGGTCAGAATTGCTTTA<br>TGGGTATGCGGACTACGCAGGCTACCAATGCTGCTACGGCTCGGGGTACGTGCCATCTAGTATTTCTCCCTTTCAAACATAGTCCATAGTTAAACAAAATTATTT<br>GTAGAGGCGGTTTCGACATCACTGTCTCATCAGAACGCAAAGCACATGCGTTGACCGCTGCTAGCAGCCTACGTAGGAAGTGATGAGTTGTCAAGTTTGTCACTA<br>TCGCCCTCTAGTGACGACTCCCAGTACAATCTGCTCTGATGCCCTCGAGTGCGGCCGCACTAGTGTCGACGGAGCTCGAATTCGGATCCGAAGTTCCTATACTTT<br>CTAGAGAATAGGAACTTCGGACCAAAACGAAAAAACACCCTTTCGGGTGTCTTTTCTGGAATTTGGTACCGAGCCTTTGGTCGAAAAAAAAAGCCCGCACTGTCA<br>GGTGCGGGCTTTTTTCTGTGTTTCC
YJP_MKC266_LP#2	ACATTTAATAAAAAAAGGGCGGTCGCAAGATCGCCCTTTTTTACGTATGACACAGTGAAAAATGGCGCCCATCGGCGCCATTTTTTTATGGAAGTTCCTATTCTC<br>TAGAAAGTATAGGAACTTCGGATCCGAATTCGAGCTCCGTCGACACTAGTGCGGCCGCACTCGAGGGCATCAGAGCAGATTGGGAGGGTATGGAAGCTATACGTT<br>ACCAATTGACAGCTAGCTCAGTCCTACTTTAGTATATAGACCGTGCGATCGGTCTATAAGCGGTCAACGCATGTGCTTTGCGTTCTGATGAGACAGTGATGTCGA<br>AACCGCCTCTACAAATAATTTTGTTTAACTATGGACTATGTTTGAAAGGGAGAAATACTAGATGGCACGTACCCCGAGCCGTAGCAGCATTGGTAGCCTGCGTAG<br>TCCGCATACCCATAAAGCAATTCTGACCAGCACCATTGAAATCCTGAAAGAATGTGGTTATAGCGGTCTGAGCATTGAAAGCGTTGCACGTCGTGCCGGTGCAAG<br>CAAACCGACCATTTATCGTTGGTGGACCAATAAAGCAGCACTGATTGCCGAAGTGTATGAAAATGAAAGCGAACAGGTGCGTAAATTTCCGGATCTGGGTAGCTT<br>TAAAGCCGATCTGGATTTTCTGCTGCGTAATCTGTGGAAAGTTTGGCGTGAAACCATTTGTGGTGAAGCATTTCGTTGTGTTATTGCAGAAGCACAGCTGGACCC<br>TGCAACCCTGACCCAGCTGAAAGATCAGTTTATGGAACGTCGTCGTGAGATGCCGAAAAAACTGGTTGAAAATGCCATTAGCAATGGTGAACTGCCGAAAGATAC<br>CAATCGTGAACTGCTGCTGGATATGATTTTTGGTTTTTGTTGGTATCGCCTGCTGACCGAACAGCTGACCGTTGAACAGGATATTGAAGAATTTACCTTCCTGCT<br>GATTAATGGTGTTTGTCCGGGTACACAGCGTTAATCCGGCAATTAAAAAAGCGGCTAACCACGCCGCTTTTTTTACGTCTGCACTCGGTACCAAATTCCAGAAAA<br>GAGGCCTCCCGAAAGGGGGGCCTTTTTTCGTTTTGGTCCTACTCTTGTCCAACCAAATGATTCGTTACCCTTTGACAGTTTCTATCGATCTATAGATAATGCTAG<br>CAGCTGTCACCGGATGTGCTTTCCGGTCTGATGAGTCCGTGAGGACGAAACAGCCTCTACAAATAATTTTGTTTAATACTAGAGAAAGAGGAGAAATACTAGATG<br>GTGAGCAAGGGCGAGGAGCTGTTCACCGGGGTGGTGCCCATCCTGGTCGAGCTGGACGGCGACGTAAACGGCCACAAGTTCAGCGTGTCCGGCGAGGGCGAGGGC<br>GATGCCACCTACGGCAAGCTGACCCTGAAGTTCATCTGCACCACAGGCAAGCTGCCCGTGCCCTGGCCCACCCTCGTGACCACCTTCGGCTACGGCCTGCAATGC<br>TTCGCCCGCTACCCCGACCACATGAAGCTGCACGACTTCTTCAAGTCCGCCATGCCCGAAGGCTACGTCCAGGAGCGCACCATCTTCTTCAAGGACGACGGCAAC<br>TACAAGACCCGCGCCGAGGTGAAGTTCGAGGGCGACACCCTGGTGAACCGCATCGAGCTGAAGGGCATCGACTTCAAGGAGGACGGCAACATCCTGGGGCACAAG<br>CTGGAGTACAACTACAACAGCCACAACGTCTATATCATGGCCGACAAGCAGAAGAACGGCATCAAGGTGAACTTCAAGATCCGCCACAACATCGAGGACGGCAGC<br>GTGCAGCTCGCCGACCACTACCAGCAGAACACCCCAATCGGCGACGGCCCCGTGCTGCTGCCCGACAACCACTACCTTAGCTACCAGTCCGCCCTGAGCAAAGAC<br>CCCAACGAGAAGCGCGATCACATGGTCCTGCTGGAGTTCGTGACCGCCGCCGGGATCACTCTCGGCATGGACGAGCTGTACAAGTAAAATGGTGTTATAAACCTG<br>TGTGAGAGTTAAGTTTACATGGGCAAAGTTGATGACCGGGTCGTCCGTTGCAGACAAAAAAAATGGCGCACAATGTGCGCCATTTTTCACTTCACAGGTACTATT<br>GTTTTGAATTGAAAAGGGCGCTTCGGCGCCCTTTTTGCATTTGTTGACGGCATATATTTGTATATCGAAGCGCCCTGATGGGCGCTTTTTTTATTTAATCGATAA<br>CCAGA
YJP_MKC267_LP#1	AAAAAAAACCCCGCCCTGTCAGGGGCGGGGTTTTTTTTTTTCTTTTGGGTATAGCGTCGTGGACAGTCATTCATCTTTCTGCCCCTCCAAAAGCAAAAACCCGC<br>CGAAGCGGGTTTTTACGTAAATCAGGTGAAACTGACCGATAAGCCGGGACGAGCTGAGCAGTATGTCGACGGTCCGGCGAGAATCTCGCGTAGACACATACATGA<br>GCTAGTTTACTTGTACAGCTCGTCCATGCCGAGAGTGATCCCGGCGGCGGTCACGAACTCCAGCAGGACCATGTGATCGCGCTTCTCGTTGGGGTCTTTGCTCAG<br>GGCGGACTGGTAGCTAAGGTAGTGGTTGTCGGGCAGCAGCACGGGGCCGTCGCCGATTGGGGTGTTCTGCTGGTAGTGGTCGGCGAGCTGCACGCTGCCGTCCTC<br>GATGTTGTGGCGGATCTTGAAGTTCACCTTGATGCCGTTCTTCTGCTTGTCGGCCATGATATAGACGTTGTGGCTGTTGTAGTTGTACTCCAGCTTGTGCCCCAG<br>GATGTTGCCGTCCTCCTTGAAGTCGATGCCCTTCAGCTCGATGCGGTTCACCAGGGTGTCGCCCTCGAACTTCACCTCGGCGCGGGTCTTGTAGTTGCCGTCGTC<br>CTTGAAGAAGATGGTGCGCTCCTGGACGTAGCCTTCGGGCATGGCGGACTTGAAGAAGTCGTGCAGCTTCATGTGGTCGGGGTAGCGGGCGAAGCATTGCAGGCC<br>GTAGCCGAAGGTGGTCACGAGGGTGGGCCAGGGCACGGGCAGCTTGCCTGTGGTGCAGATGAACTTCAGGGTCAGCTTGCCGTAGGTGGCATCGCCCTCGCCCTC<br>GCCGGACACGCTGAACTTGTGGCCGTTTACGTCGCCGTCCAGCTCGACCAGGATGGGCACCACCCCGGTGAACAGCTCCTCGCCCTTGCTCACCATCTAGTATTT<br>CTCCTCTTTCTCTAGTATTAAACAAAATTATTTGTAGAGGCTGTTTCGTCCTCACGGACTCATCAGACCGGAAAGCACATCCGGTGACAGCTACCTTAACGATAC<br>GGTACGTTTCGTATCATGTCAATTGGTAACGAATCAGATTCCACCGTACGTCGAAGCGTCAGAGGAGCCAAATTCAAAAAAGCCTGCTTTCTAGCAGGCTTTTTG<br>CTTTCTAATGGAAGCATAAAAAAATGGCGCCGATGGGCGCCATTTTTCACTGCGGCAAGAATTACTTCAGACAGAGTATCAAAAGGCGAAACCTCCGCAATGCGG<br>AGGTTTCTTTTTAAAGATTATTTGCTCAGGCCATTCAGAAAGATCTGGCTGAATTTGTTCATCAGTTTAATGCGTTCGTTGATATTCTGCTCATGGGTAAAGGTA<br>ACAATGCCATTAACGGCATTTGCTGCAATTTTGCTCACGGCATTAACATCATTAATGCACCATTCGCCGTTCAGATTACCTTCTTTAAAGATCACGTGGTAGGCA<br>TCGATGTATTTGTTTTCCAGTTTGTTCATTTTCTCGTTGATGCTGTTGGTTTTATAGTACTCGGTATAAAACTCGATGATGGCATTCTGCAGCGGATAGTAATAT<br>TCGGTGGTCAGGCTCAGTTCATTATACAGATAGAATTTCTCGCGGTTGGTTTTGCATTTGATTTGTTCTTTTTTCCACTGCTCTTGCCATTTGCTTTCTTCGATG<br>TTCAGGATTTCCAGAAACAGGTTCTCTTTGGTTTTAAAGTGATAATACAGATTGCCTTTGCTGCTTTCGCTCAGTTTAACAATTTCACCGGTGGTGGTTGCGTTA<br>TAGCCATTTTTGATAAACAGTTCTTTGGCAACGCCCAGAATTTTATCTTTCAGGTTCATCTAGTAGTTGTCCTCTATCAAAAGCCAATGGCATGGCTTAAACAAA<br>ATTATTTGTAGAGGTCGTTTCGGCATCACTGCCTCATCAGAAGTGCAAGCACAGCACTTGACGACTATTGGATCCAATGGTACCTAGGACTGAGCTAGCTGTCAA<br>TTGGATCCAATAAGGCAGGTAGGCTCACCTGCGGAGAAAAGGTCCGAAAATTCGGACCCGATGGAATTGGATAAAAAAAGGCCCCTGTTGAAATTGCAGGGGCCT<br>GGTACGAGCATTATTTTGCATCTGCCTGTTTAATCAGTTCCAGGGTTTTTTCAACACGATCTGCAGGCAGCGGTGCACCCAGAACTGCCAGGCTTGCATCTGCCA<br>GCATAATTGCGGTTTCCGGCAGGCTATCTGCGCTCAGCGGACTCGGAATTTTACCATCATTGCGACGCATTTCAATAACGCTCATGGTAATATGAAACGGCAGTT<br>CTGCACGCGGATCATCACCAACAATTTCGGTTGCCAGATCACGAAAAACATTGGTCAGTGCTTCACGCTGGCTATGATATTCTGCAAATTCTTCGCTACCAACAA<br>TCGGCAGCTGATACAGACGACCAACATTCCATTTGGTGCTCAGCAGCAGACGAACTTCGCTTGCAACAATTGCCCACAGACGCATTTCCGGACCTGCATCCAGGG<br>TGCTCAGATCTTCTGCCAGAACGGTGCTCGGTTCAACGGTGCTTTTCAGCAGGGTCAGAAAGATTTCGGTTTTGCTCGGAAAATGATAATACAGGCTTGCCTGAC<br>GAATACCAACTGCATCTGCAATCTGATGGGTACTGGTGGTTGCAAAACCCTGACGGGTAAACAGTTCTGCGCTTGCATCCAGAATTTCTTCACGCGGATTTTTAC<br>CTGCACGACGCGGTGCACTACGACGCGGACGACCAACTGCGCCTGCCATCTAGTAACCTCTTTGCTGATTATTAGGGAACATTTTAAACAAAATTATTTGTAGAG<br>GGGTGTTTCGTCCTTTCGGACTCATCAGTCAAGGTACGCACCTTGAGACACCCTGTGCTCATTATATCTATCACTGATAGGGATGTCAATCTCTATCACTGATAG<br>GGAGCGAGAGTGGTTGCGCATTCATAAAAAAATGGCGCCGATGGGCGCCATTTTTCACTGTGTCATACGTAAAAAAGGGCGATCTTGCGACCGCCCTTTTTTTAT<br>TAAATGTTTATTTTGCCAGCGGATTACGCAGACCTGCATCAATCAGATCAACCAGCTGATCACAACGGGTTGCCAGATTACCAATATCTTCTTGTGCAATCAGAA<br>CCGGATGAATAACGCTGGTCAGTGCATGCAGAACGGTTTCTGCCAGAACCGGAATATCGGTTGCAATATACAGACCTGCTTCAACACCATCACGAATAATTTCGG<br>CCAGCAGTTTTGCAATCACGCTTTTGTAATAATCGCCACATTTCATATCCTGTTTGGCGGTCATCAGGATCTGAATAAAAACCCGTATGTGTTTGAAATGATCCT<br>GATGATGCTGTTCCATCAGATTACGTGCCAGATGACGCAGACGATCCAGCGGTGCATGGCTTTTATCCAGCGGACAAATCTGACGTTCAAAAACACCAATCTGAC<br>CAAAACCAATTGCATCAACCAGTGCGTTTTTGCTGCTAAAATGTTTAAACACATTTGCCGGACTCATATTCAGTGCGCTTGCAATATCTGCAATGGCAACTGCAT<br>TAAAACCACGTTCACTAAACAGTTCTTCGGCTTTTGCCAGAATATCACGACGGGTTTCTTCGGCACTATGACGCGGACGACGAACCGGCAGATCGCTTTTAC<br>GATCACCTTTACGCACCTGATCAATGGTTTTGTTCATCTATTAGCTCCTGTTTGAAACATAGTCCATAGCGTATTAAACAAAATTATTTGTAGAGGGGGTTTCGT<br>CATCACTGACTCATCAGAGTTGAAAGCACATCAACTGACCCCTAGCGTTTTCAAGTTCGTGGAAAGCAGAATGGTTCAGGCGAGCCGGATCGGAAATTCCCTCAG<br>ATAGGCCCCCCCAGATGAGGGGGATCAGAGCATGTCGCGCAAAAGTGTGCAGCGGTTTTGCGATAACGACATGCGCCAAAACAAAGACTGAAAGCGCGAGGAGCG<br>AATCTGAAAGATCGCGACCCGCTTTAGAGCGCCGTGCGTCCGTTTGGACGCACAAAGGGACTTTTGTGCTACCAAGTAGGACCAAAACGAAAAAAGGCCCCCCTT<br>TCGGGAGGCCTCTTTTCTGGAATTTGGTACCGAGTGCAGACGTAAAAAAAGCGGCGTGGTTAGCCGCTTTTTTAATTGCCGGATTAACGCTGTGTACCCGGACAA

	ACACCATTAATCAGCAGGAAGGTAAATTCTTCAATATCCTGTTCAACGGTCAGCTGTTCGGTCAGCAGGCGATACCAACAAAAACCAAAAATCATATCCAGCAGC<br>AGTTCACGATTGGTATCTTTCGGCAGTTCACCATTGCTAATGGCATTTTCAACCAGTTTTTTCGGCATCTCACGACGACGTTCCATAAACTGATCTTTCAGCTGG<br>GTCAGGGTTGCAGGGTCCAGCTGTGCTTCTGCAATAACACAACGAAATGCTTCACCACAAATGGTTTCACGCCAAACTTTCCACAGATTACGCAGCAGAAAATCC<br>AGATCGGCTTTAAAGCTACCCAGATCCGGAAATTTACGCACCTGTTCGCTTTCATTTTCATACACTTCGGCAATCAGTGCTGCTTTATTGGTCCACCAACGATAA<br>ATGGTCGGTTTGCTTGCACCGGCACGACGTGCAACGCTTTCAATGCTCAGACCGCTATAACCACATTCTTTCAGGATTTCAATGGTGCTGGTCAGAATTGCTTTA<br>TGGGTATGCGGACTACGCAGGCTACCAATGCTGCTACGGCTCGGGGTACGTGCCATCTAGTATTTCTCCCTTTCAAACATAGTCCATAGTTAAACAAAATTATTT<br>GTAGAGGCGGTTTCGACATCACTGTCTCATCAGAACGCAAAGCACATGCGTTGACCGCTGCTAGCAGCCTACGTAGGAAGTGATGAGTTGTCAAGTTTGTCACTA<br>TCGCCCTCTAGTGACGACTCCCAGTACAATCTGCTCTGATGCCCTCGAGTGCGGCCGCACTAGTGTCGACGGAGCTCGAATTCGGATCCGAAGTTCCTATACTTT<br>CTAGAGAATAGGAACTTCGGAATAGGAACTTCTATTTTTATAGGTTAATGTCATGATAATAATGGTTTCTTAGACCATGCCTATTTGTTTATTTTTCTAAATACA<br>TTCAAATATGTATCCGCTCATGAGACAATAACCCTGATAAATGCTTCAATAATATTGAAAAAGGAAGAGTATGAGCCATATTCAACGGGAAACGTCTTGCTCTAG<br>GCCGCGATTAAATTCCAACATGGATGCTGATTTATATGGGTATAAATGGGCTCGCGATAATGTCGGGCAATCAGGTGCGACAATCTATCGATTGTATGGGAAGCC<br>CGATGCGCCAGAGTTGTTTCTGAAACATGGCAAAGGTAGCGTTGCCAATGATGTTACAGATGAGATGGTCAGACTAAACTGGCTGACGGAATTTATGCCTCTTCC<br>GACCATCAAGCATTTTATCCGTACTCCTGATGATGCATGGTTACTCACCACTGCGATCCCCGGGAAAACAGCATTCCAGGTATTAGAAGAATATCCTGATTCAGG<br>TGAAAATATTGTTGATGCGCTGGCAGTGTTCCTGCGCCGGTTGCATTCGATTCCTGTTTGTAATTGTCCTTTTAACAGCGACCGCGTATTTCGTCTCGCTCAGGC<br>GCAATCACGAATGAATAACGGTTTGGTTGATGCGAGTGATTTTGATGACGAGCGTAATGGCTGGCCTGTTGAACAAGTCTGGAAAGAAATGCATAAACTTTTGCC<br>ATTCTCACCGGATTCAGTCGTCACTCATGGTGATTTCTCACTTGATAACCTTATTTTTGACGAGGGGAAATTAATAGGTTGTATTGATGTTGGACGAGTCGGAAT<br>CGCAGACCGATACCAGGATCTTGCCATCCTATGGAACTGCCTCGGTGAGTTTTCTCCTTCATTACAGAAACGGCTTTTTCAAAAATATGGTATTGATAATCCTGA<br>TATGAATAAATTGCAGTTTCATTTGATGCTCGATGAGTTTTTCTAAAGCTCGCTTGGACTCCTGTTGATAGATCCAGTAATGACCTCAGAACTCCATCTGGATTT<br>GTTCAGAACGCTCGGTTGCCGCCGGGCGTTTTTTATTGGTGAGAATCCAAGCACAAGAAGATCCGGCCACGATGCGTCCGGCGTAGAGGATCTGAAGATCAGCAG<br>TTCAACCTGTTGATAGTACGTACTAAGCTCTCATGTTTCACGTACTAAGCTCTCATGTTTAACGTACTAAGCTCTCATGTTTAACGAACTAAACCCTCATGGCTA<br>ACGTACTAAGCTCTCATGGCTAACGTACTAAGCTCTCATGTTTCACGTACTAAGCTCTCATGTTTGAACAATAAAATTAATATAAATCAGCAACTTAAATAGCCT<br>CTAAGGTTTTAAGTTTTATAAGAAAAAAAAGAATATATAAGGCTTTTAAAGCTTTTAAGGTTTAACGGTTGTGGACAACAAGCCAGGGATGTAACGCACTGAGAA<br>GCCCTTAGAGCCTCTCAAAGCAATTTTGAGTGACACAGGAACACTTAACGGCTGACATGGGAATTAGCCATGGGCCCGTGCGAATCATTTTTACGGTTCCTGGCC<br>TTTTGCTGGCCTTTTGCTCACATGTTCTTTCCTGCGTGGATAACCGTATTACCGCCTGGACCAAAACGAAAAAACACCCTTTCGGGTGTCTTTTCTGGAATTTGG<br>TACCGAGCCTTTGGTCGAAAAAAAAAGCCCGCACTGTCAGGTGCGGGCTTTTTTCTGTGTTTCCAGCCAAGAGCAGTGCCTTGCAGAAGTTCTCGGGCGAAGAGC<br>TACTCCCCTTCTGCGCCGTCCGAAGTTCCTATACTTTCTAGAGAATAGGAACTTCGGACCAAAACGAAAAAACACCCTTTCGGGTGTCTTTTCTGGAATTTGGTA<br>CCGAGCCTTTGGTCGAAAAAAAAAGCCCGCACTGTCAGGTGCGGGCTTTTTTCTGTGTTTCC
YJP_MKC266_LP#2	ACATTTAATAAAAAAAGGGCGGTCGCAAGATCGCCCTTTTTTACGTATGACACAGTGAAAAATGGCGCCCATCGGCGCCATTTTTTTATGGAAGTTCCTATTCTC<br>TAGAAAGTATAGGAACTTCAGACGAGAAACGTTCCGTCCGTCTGGGTCAGTTGCCTAACCTTAACTTTACGCAGGTTCAGCTTGGAAACACAGAAAAAAGCCCG<br>CACCTGACAGTGCGGGCTTTTTTTTTCGACCAAAGGCTCGGTACCAAATTCCAGAAAAGACACCCGAAAGGGTGTTTTTTCGTTTTGGTCCAGGCGGTAATACGG<br>TTATCCACAGAATCAGGGGATAACGCAGGAAAGAACATGTGAGCAAAAGGCCAGCAAAAGGCCAGGAACCGTAAAAATGATTCGCACGGGCCCATGGCTAATTAC<br>CATGTCGGTAATGACTCCAACTTATTGATAGTGTTTTATGTTCAGATAATGCCCGATGACTTTGTCATGCAGCTCCACCGATTTTGAGAACGACAGCGACTTCCG<br>TCCCAGCCGTGCCAGGTGCTGCCTCAGATTCAGGTTATGCCGCTCAATTCGCTGCGTATATCGCTTGCTGATTACGTGCAGCTTTCCCTTCAGGCGGGATTCATA<br>CAGCGGCCAGCCATCCGTCATCCATATCACCACGTCAAAGGGTGACAGCAGGCTCATAAGACGCCCCAGCGTCGCCATAGTGCGTTCACCGAATACGTGCGCAAC<br>AAACGTCTTCCGGAGACTGTCATACGCGTAAAACAGCCAGCGCTGGCGCGATTTAGCCCCGACATAGCCCCACTGTTCGTCCATTTCCGCGCAGACGATGACGTC<br>ACTGCCCGGCTGTATGCGCGAGGTTACCGACTGCGGCCTGAGTTTTTTAAGTGACGTAAAATCGTGTTGAGGCCAACGCCCATAATGCGGGCTGTTGCCCGGCAT<br>CCAACGCCATTCATGGCCATATCAATGATTTTCTGGTGCGTACCGGGTTGAGAAGCGGTGTAAGTGAACTGCAGTTGCCATGTTTTACGGCAGTGAGAGCAGAGA<br>TAGCGCTGATGTCCGGCGGTGCTTTTGCCGTTACGCACCACACCGTCAGTAGCTGAACAGGAGGGACAGCTGATAGAAACAGAAGCCACTGGAGCACCTCAAAAA<br>CACCATCATACACTAAATCAGTAAGTTGGCAGCATCACCTCCCATGTCAGCCGTTAAGTGTTCCTGTGTCACTCAAAATTGCTTTGAGAGGCTCTAAGGGCTTCT<br>CAGTGCGTTACATCCCTGGCTTGTTGTCCACAACCGTTAAACCTTAAAAGCTTTAAAAGCCTTATATATTCTTTTTTTTCTTATAAAACTTAAAACCTTAGAGGC<br>TATTTAAGTTGCTGATTTATATTAATTTTATTGTTCAAACATGAGAGCTTAGTACGTGAAACATGAGAGCTTAGTACGTTAGCCATGAGAGCTTAGTACGTTAGC<br>CATGAGGGTTTAGTTCGTTAAACATGAGAGCTTAGTACGTTAAACATGAGAGCTTAGTACGTGAAACATGAGAGCTTAGTACGTACTATCAACAGGTTGAACTGC<br>TGATCTTCAGATCCTCTACGCCGGACGCATCGTGGCCGGATCTTCTTGTGCTTGGATTCTCACCAATAAAAAACGCCCGGCGGCAACCGAGCGTTCTGAACAAAT<br>CCAGATGGAGTTCTGAGGTCATTACTGGATCTATCAACAGGAGTCCAAGCGAGCTTTACGCCCCGCCCTGCCACTCATCGCAGTACTGTTGTAATTCATTAAGCA<br>TTCTGCCGACATGGAAGCCATCACAAACGGCATGATGAACCTGAATCGCCAGCGGCATCAGCACCTTGTCGCCTTGCGTATAATATTTGCCCATGGTGAAAACGG<br>GGGCGAAGAAGTTGTCCATATTGGCCACGTTTAAATCAAAACTGGTGAAACTCACCCAGGGATTGGCTGAGACGAAAAACATATTCTCAATAAACCCTTTAGGGA<br>AATAGGCCAGGTTTTCACCGTAACACGCCACATCTTGCGAATATATGTGTAGAAACTGCCGGAAATCGTCGTGGTATTCACTCCAGAGCGATGAAAACGTTTCAG<br>TTTGCTCATGGAAAACGGTGTAACAAGGGTGAACACTATCCCATATCACCAGCTCACCGTCTTTCATTGCCATACGAAATTCCGGATGAGCATTCATCAGGCGGG<br>CAAGAATGTGAATAAAGGCCGGATAAAACTTGTGCTTATTTTTCTTTACGGTCTTTAAAAAGGCCGTAATATCCAGCTGAACGGTCTGGTTATAGGTACATTGAG<br>CAACTGACTGAAATGCCTCAAAATGTTCTTTACGATGCCATTGGGATATATCAACGGTGGTATATCCAGTGATTTTTTTCTCCATTTTAGCTTCCTTAGCTCCTG<br>AAAATCTCGATAACTCAAAAAATACGCCCGGTAGTGATCTTATTTCATTATGGTGAAAGTTGGAACCTCTTACGTGCCGATCAACGTCTCATTTTCGCCAATTTA<br>AATCGTAATTATTGGGGACCCTGTATTTAGAAAAATAAACAAATAGGCATGGTCTAAGAAACCATTATTATCATGACATTAACCTATAAAAATAGAAGTTCCTAT<br>TCCGAAGTTCCTATTCTCTAGAAAGTATAGGAACTTCGGATCCGAATTCGAGCTCCGTCGACACTAGTGCGGCCGCACTCGAGGGCATCAGAGCAGATTGGGAGG<br>GTATGGAAGCTATACGTTACCAATTGACAGCTAGCTCAGTCCTACTTTAGTATATAGACCGTGCGATCGGTCTATAAGCGGTCAACGCATGTGCTTTGCGTTCTG<br>ATGAGACAGTGATGTCGAAACCGCCTCTACAAATAATTTTGTTTAACTATGGACTATGTTTGAAAGGGAGAAATACTAGATGGCACGTACCCCGAGCCGTAGCAG<br>CATTGGTAGCCTGCGTAGTCCGCATACCCATAAAGCAATTCTGACCAGCACCATTGAAATCCTGAAAGAATGTGGTTATAGCGGTCTGAGCATTGAAAGCGTTGC<br>ACGTCGTGCCGGTGCAAGCAAACCGACCATTTATCGTTGGTGGACCAATAAAGCAGCACTGATTGCCGAAGTGTATGAAAATGAAAGCGAACAGGTGCGTAAATT<br>TCCGGATCTGGGTAGCTTTAAAGCCGATCTGGATTTTCTGCTGCGTAATCTGTGGAAAGTTTGGCGTGAAACCATTTGTGGTGAAGCATTTCGTTGTGTTATTGC<br>AGAAGCACAGCTGGACCCTGCAACCCTGACCCAGCTGAAAGATCAGTTTATGGAACGTCGTCGTGAGATGCCGAAAAAACTGGTTGAAAATGCCATTAGCAATGG<br>TGAACTGCCGAAAGATACCAATCGTGAACTGCTGCTGGATATGATTTTTGGTTTTTGTTGGTATCGCCTGCTGACCGAACAGCTGACCGTTGAACAGGATATTGA<br>AGAATTTACCTTCCTGCTGATTAATGGTGTTTGTCCGGGTACACAGCGTTAATCCGGCAATTAAAAAAGCGGCTAACCACGCCGCTTTTTTTACGTCTGCACTCG<br>GTACCAAATTCCAGAAAAGAGGCCTCCCGAAAGGGGGGCCTTTTTTCGTTTTGGTCCTACTCTTGTCCAACCAAATGATTCGTTACCCTTTGACAGTTTCTATCG<br>ATCTATAGATAATGCTAGCAGCTGTCACCGGATGTGCTTTCCGGTCTGATGAGTCCGTGAGGACGAAACAGCCTCTACAAATAATTTTGTTTAATACTAGAGAAA<br>GAGGAGAAATACTAGATGGTGAGCAAGGGCGAGGAGCTGTTCACCGGGGTGGTGCCCATCCTGGTCGAGCTGGACGGCGACGTAAACGGCCACAAGTTCAGCGTG<br>TCCGGCGAGGGCGAGGGCGATGCCACCTACGGCAAGCTGACCCTGAAGTTCATCTGCACCACAGGCAAGCTGCCCGTGCCCTGGCCCACCCTCGTGACCACCTTC<br>GGCTACGGCCTGCAATGCTTCGCCCGCTACCCCGACCACATGAAGCTGCACGACTTCTTCAAGTCCGCCATGCCCGAAGGCTACGTCCAGGAGCGCACCATCTTC<br>TTCAAGGACGACGGCAACTACAAGACCCGCGCCGAGGTGAAGTTCGAGGGCGACACCCTGGTGAACCGCATCGAGCTGAAGGGCATCGACTTCAAGGAGGACGGC<br>AACATCCTGGGGCACAAGCTGGAGTACAACTACAACAGCCACAACGTCTATATCATGGCCGACAAGCAGAAGAACGGCATCAAGGTGAACTTCAAGATCCGCCAC<br>AACATCGAGGACGGCAGCGTGCAGCTCGCCGACCACTACCAGCAGAACACCCCCAATCGGCGACGGCCCCGTGCTGCTGCCCGACAACCACTACCTTAGCTACCAG<br>TCCGCCCTGAGCAAAGACCCCAACGAGAAGCGCGATCACATGGTCCTGCTGGAGTTCGTGACCGCCGCCGGGATCACTCTCGGCATGGACGAGCTGTACAAGTAA<br>AATGGTGTTATAAACCTGTGTGAGAGTTAAGTTTACATGGGCAAAGTTGATGACCGGGTCGTCCGTTGCAGACAAAAAAAATGGCGCACAATGTGCGCCATTTTT<br>CACTTCACAGGTACTATTGTTTTGAATTGAAAAGGGCGCTTCGGCGCCCTTTTTGCATTTGTTGACGGCATATATTTGTATATCGAAGCGCCCTGATGGGCGCTT<br>TTTTTATTTAATCGATAACCAGA

a. *Annotation colors. Blue: Promoters; Purple: Ribozymes; Green: Repressors, YFP and Sensor ORFs; Red: Terminators; Orange: FRT sites and att sites; yellow highlight: RBSs; Gray highlight: antibiotic resistance gene and origin of rep; Green highlight: Transposase (Tn5, Tn7) recognition sites*

Appendix Table 8: Strains used in this study

Strain Name	Plasmid Integrated	Related figure	Marker removal
YJP_DHC404	plYJP019	Fig 1b	N
YJP_MKC172	Landing pad DNA, plYJP072	Fig 1c	N
YJP_MKC173	Landing pad DNA, plYJP072	Fig 1c, Fig 5	Y
YJP_MKC174	plYJP064-Sensor	Fig 2c, Appendix Fig 5	N
YJP_MKC108	plYJP064-Sensor, plYJP067-PTac	Fig 2c, Appendix Fig 5	N
YJP_MKC110	plYJP064-Sensor, plYJP067-PBadmc	Fig 2c, Appendix Fig 5	N
YJP_MKC111	plYJP064-Sensor, plYJP067-PTet	Fig 2c, Appendix Fig 5	N
YJP_MKC112	plYJP064-Sensor, plYJP067-PCymRC	Fig 2c, Appendix Fig 5	N
YJP_MKC113	plYJP064-Sensor, plYJP067-PVanCC	Fig 2c, Appendix Fig 5	N
YJP_MKC114	plYJP064-Sensor, plYJP067-PCinR	Fig 2c, Appendix Fig 5	N
YJP_MKC115	plYJP064-Sensor, plYJP067-PTtg	Fig 2c, Appendix Fig 5	N
YJP_MKC254	plYJP067-PJ23101H	Fig 2a, 2b	Y
YJP_MKC255	plYJP067-PJ23101L	Appendix Fig 4	Y
YJP_MKC140	plYJP064-Sensor, plYJP066-P3-PhlF, plYJP070-PPhlF	Appendix Fig 7	N
YJP_MKC141	plYJP064-Sensor, plYJP066-Q1-QacR, plYJP070-PQacR	Fig 2d	N
YJP_MKC142	plYJP064-Sensor, plYJP066-A1-AmtR, plYJP070-PAmtR	Fig 2d	N
YJP_MKC146	plYJP064-Sensor, plYJP066-F1-AmeR, plYJP070-PAmeR	Appendix Fig 7	N
YJP_MKC149	plYJP064-Sensor, plYJP066-B3-BM3R1, plYJP070-PBM3R1	Fig 2d	N
YJP_MKC154	plYJP064-Sensor, plYJP066-F1-AmeRs, plYJP070-PAmeR	Fig 2d	N
YJP_MKC155	plYJP064-Sensor, plYJP066-E0-BetI, plYJP070-PBetI	Appendix Fig 7	N
YJP_MKC156	plYJP064-Sensor, plYJP066-P1-PhlF, plYJP070-PPhlF	Fig 2d	N
YJP_MKC158	plYJP064-Sensor, plYJP066-E1-BetI, plYJP070-PBetI	Fig 2d	N
YJP_MKC258	plYJP064-Sensor, plYJP066-NOR-Tandem, plYJP070-PPhlF	Fig 3a, 3c	N
YJP_MKC259	plYJP064-Sensor, plYJP066-NOR-Split, plYJP070-NOR-Split	Fig 3b, 3c	N
YJP_MKC260	plYJP064-Sensor, plYJP066-AND-Tandem, plYJP070-PPhlF	Fig 3d, 3f	Y
YJP_MKC261	plYJP064-Sensor, plYJP066-0x08-Tandem, plYJP070-PPhlF	Fig 3f, Appendix Fig 9	Y
YJP_MKC262	plYJP064-Sensor, plYJP066-AND-Split, plYJP070-AND-Split	Fig 3e, 4	Y
YJP_MKC263	plYJP064-Sensor, plYJP066-XNOR-Split, plYJP070-XNOR-Split	Fig 4	Y
YJP_MKC264	plYJP064-Sensor, plYJP066-0x08-Split, plYJP070-0x08-Split	Fig 4	Y
YJP_MKC265	plYJP064-Sensor, plYJP066-0x0B-Split, plYJP070-0x0B-Split	Fig 4	Y
YJP_MKC266	plYJP064-Sensor, plYJP066-0xF1-Split, plYJP070-0xF1-Split	Fig 4	Y
YJP_MKC267	plYJP064-Sensor, plYJP066-0xF1-Split, plYJP070-0xF1-Split	Fig 5	N

References

1 Shin, J., Zhang, S., Der, B. S., Nielsen, A. A. & Voigt, C. A. Programming Escherichia coli to function as a digital display. *Mol Syst Biol* **16**, e9401, doi:10.15252/msb.20199401 (2020).
