# Supplementary material for: Precision design of stable genetic circuits carried in highly‐insulated E. coli genomic landing pads
Source: Mol Syst Biol. 2020 Aug 19;16(8):e9584. doi: 10.15252/msb.20209584 (PMC7436927; doi:10.15252/msb.20209584)
Supplement: Supplementary file 2 — Dataset EV1 [file MSB-16-e9584-s002.zip › Dataset EV1/Dataset EV1 Legend.docx]

Dataset EV1:

The datasets and computer code produced in this study are available in the following databases:

UCF information (Eco2C1G3T.UCF, Eco2C1G3T.input, and Eco2C1G3T.output) is available in Dataset EV1.

Codes used to process the data are available on github (github.com/CIDARLAB/Cello-v2).
